# Supplementary material for: Key factors for effective implementation of healthcare workers support interventions after patient safety incidents in health organisations: a scoping review
Source: BMJ Open. 2023 Dec 27;13(12):e078118. doi: 10.1136/bmjopen-2023-078118 (PMC10753749; doi:10.1136/bmjopen-2023-078118)
Supplement: Supplementary data [file bmjopen-2023-078118supp002.pdf]

Supplementary Table 1 - Search strategies applied to the different electronic databases

**Applied search strategies :**			**Results**
Context	#1	“Health Services” OR “Health Facilities” OR “Healthcare” OR “Primary health care” OR “General Practice” OR “Family practice” OR “Ambulatory Care” OR “Nursing Care” OR “Family unit” OR "Hospitals"	
Content	#2	“Program evaluation” OR "Support program” OR “Peer support” OR “Support strategies” OR “Organizational factors” OR “Organizational culture”	
Population	#3	(“Health personnel” OR “Physicians” OR “Nurses” OR “Doctor” OR “Practitioner” OR “Medical students” OR “Medical residents” OR “Healthcare providers” OR “Healthcare worker” OR “Healthcare staff") AND (“Error” OR “Near miss” OR “Adverse Event” OR “Clinical Error” OR “Medical error” OR “Second victim” OR “Wounded caregiver” OR “Wounded healer” OR “Secondary trauma”)	
Web of Science Core collection<br>*All databases*<br><br>Last updated 2022/10/31	#1 AND #2 AND #3	Error OR Adverse Event OR Clinical Error OR Medical error OR Second victim OR Wounded caregiver OR Wounded healer OR Secondary trauma (Topic) and Health Services OR Health Facilities OR Healthcare OR Primary health care OR General Practice OR Family practice OR Ambulatory Care OR Nursing Care OR Family unit OR Hospitals (Topic) and Program evaluation OR Support program OR Peer support OR Support strategies OR Organizational factors OR Organizational culture (Topic) and Health personnel OR Physicians OR Nurses OR Doctor OR Practitioner OR Medical students OR Medical residents OR Healthcare providers OR Healthcare worker OR Healthcare staff (Topic) and Article or Review Article or Early Access or Editorial Material (Document Types)	1005 results
Complete search on Pubmed (Medline**)**<br><br>Last updated 2022/10/31	#1 AND #2 AND #3	((((("Health Services"[MeSH Terms]) OR ("Health Services"[Title/Abstract])) OR (("Health Facilities"[MeSH Terms]))) OR ("Health Facilities"[Title/Abstract])) OR ("healthcare"[Title/Abstract])) OR ("Primary health care"[MeSH Terms])) OR ("Primary health care"[Title/Abstract])) OR ("General Practice"[MeSH Terms])) OR ("General Practice"[Title/Abstract])) OR ("Family practice"[MeSH Terms])) OR ("Family practice"[Title/Abstract])) OR ("Ambulatory Care"[MeSH Terms])) OR ("Ambulatory Care"[Title/Abstract])) OR ("Nursing Care"[Title/Abstract])) OR ("Family unit"[Title/Abstract])) OR ("Hospitals"[Title/Abstract]))) AND (("Program evaluation"[Title/Abstract]) OR ("Support program"[Title/Abstract]) OR ("Peer support"[Title/Abstract]) OR ("Support strategies"[Title/Abstract]) OR ("Organizational factors"[Title/Abstract]) OR ("Organizational culture"[MeSH Terms]) OR ("Organizational culture"[Title/Abstract])))) AND (("Health personnel"[MeSH Terms]) OR ("Health personnel"[Title/Abstract]) OR ("Physicians"[Title/Abstract]) OR ("Nurses"[Title/Abstract]) OR ("Doctor"[Title/Abstract]) OR ("Practitioner"[Title/Abstract]) OR ("Medical students"[Title/Abstract]) OR ("Medical residents"[Title/Abstract]) OR ("Healthcare providers"[Title/Abstract]) OR ("Healthcare worker"[Title/Abstract]) OR ("Healthcare staff"[Title/Abstract])))) AND ((Error[Title/Abstract]) OR ((“Near miss”[Title/Abstract]) OR (“Adverse Event”[Title/Abstract]) OR (“Clinical Error”[Title/Abstract]) OR (“Medical error”[MeSH Terms]) OR (“Medical error”[Title/Abstract]) OR (“Second victim”[Title/Abstract]) OR (“Wounded caregiver”[Title/Abstract]) OR (“Wounded healer”[Title/Abstract]) OR (“Secondary trauma”[Title/Abstract])))	743 results

Complete search on PsycInfo<br><br>Last updated 2022/11/3	#1 AND #2 AND #3	(((health services) OR (health facilities) OR (healthcare) OR (primary health care) OR (general practice) OR (family practice) OR (ambulatory care) OR (nursing care) OR (family unit) OR (hospital)) AND ((program evaluation) OR (support programs) OR (peer support) OR (support strategies) OR (organizational factors) OR (organizational culture)) AND ((health personnel) OR (physician) OR (nurse) OR (doctors) OR (practitioner) OR (medical students) OR (medical residents) OR (healthcare providers) OR (healthcare workers) OR (healthcare staff)) AND ((errors) OR (near miss) OR (adverse events) OR (clinical errors) OR (medical errors) OR (second victim) OR (wounded healer) OR (secondary trauma))	787 results
SCOPUS<br><br>Last updated 2022/11/2	#1 AND #2 AND #3	(TITLE-ABS-KEY (health AND services) OR TITLE-ABS-KEY (health AND facilities) OR TITLE-ABS-KEY (healthcare) OR TITLE-ABS-KEY (primary AND health AND care) OR TITLE-ABS-KEY (general AND practice) OR TITLE-ABS-KEY (family AND practice) OR TITLE-ABS-KEY (ambulatory AND care) OR TITLE-ABS-KEY (nursing AND care) OR TITLE-ABS-KEY (family AND unit) OR TITLE-ABS-KEY (hospitals)) AND (TITLE-ABS-KEY (program AND evaluation) OR TITLE-ABS-KEY (support AND program) OR TITLE-ABS-KEY (peer AND support) OR TITLE-ABS-KEY (support AND strategies) OR TITLE-ABS-KEY (organizational AND factors) OR TITLE-ABS-KEY (organizational AND culture)) AND (TITLE-ABS-KEY (health AND personnel) OR TITLE-ABS-KEY (physicians) OR TITLE-ABS-KEY (nurses) OR TITLE-ABS-KEY (doctor) OR TITLE-ABS-KEY (practitioner) OR TITLE-ABS-KEY (medical AND students) OR TITLE-ABS- KEY (medical AND residents) OR TITLE-ABS-KEY (healthcare AND providers) OR TITLE-ABS-KEY (healthcare AND worker) OR TITLE- ABS-KEY (healthcare AND staff)) AND (TITLE-ABS-KEY (error) OR TITLE-ABS-KEY (near AND miss) OR TITLE-ABS-KEY (adverse AND event) OR TITLE-ABS-KEY (clinical AND error) OR TITLE-ABS-KEY (medical AND error) OR TITLE-ABS-KEY (second AND victim) OR TITLE-ABS-KEY (wounded AND caregiver) OR TITLE-ABS-KEY (wounded AND healer) OR TITLE-ABS-KEY (secondary AND trauma)) AND (LIMIT-TO (DOCTYPE , "ar") OR LIMIT-TO (DOCTYPE , "re") OR LIMIT-TO (DOCTYPE , "cp") OR LIMIT-TO (DOCTYPE , "cr"))	5074 results
Complete search on CINAHL<br><br>Last updated 2022/10/31	#1 AND #2 AND #3	TX ("health services" OR "health facilities" OR healthcare OR "primary health care" OR "general practice" OR "family practice" OR "ambulatory care" OR "nursing care" OR "family unit" OR hospital) AND TX ("program evaluation" OR "support programs" OR "peer support" OR "support strategies" OR "organizational factors" OR "organizational culture") AND TX ("health personnel" OR physician OR nurse OR doctors OR practitioner OR "medical students" OR "medical residents" OR "healthcare providers" OR "healthcare workers" OR "healthcare staff") AND TX (errors OR "near miss" OR "adverse events" OR "clinical errors" OR "medical errors" OR "second victim" OR "wounded healer" OR "secondary trauma")	1163 results
Complete search on Embase<br><br>Last updated 2022/10/31	#1 AND #2 AND #3	('health services':ab,ti OR 'health facilities':ab,ti OR 'healthcare':ab,ti OR 'primary health care':ab,ti OR 'general practice':ab,ti OR 'family practice':ab,ti OR 'ambulatory care':ab,ti OR 'nursing care':ab,ti OR 'family unit':ab,ti OR 'hospitals':ab,ti) AND ('program evaluation':ab,ti OR 'support program':ab,ti OR 'peer support':ab,ti OR 'support strategies':ab,ti OR 'organizational factors':ab,ti OR 'organizational culture':ab,ti) AND ('health personnel':ab,ti OR 'physicians':ab,ti OR 'nurses':ab,ti OR 'doctor':ab,ti OR 'practitioner':ab,ti OR 'medical students':ab,ti OR 'medical residents':ab,ti OR 'healthcare providers':ab,ti OR 'healthcare worker':ab,ti OR 'healthcare staff':ab,ti) AND (error:ab,ti OR 'adverse event':ab,ti OR 'clinical error':ab,ti OR 'medical error':ab,ti OR 'second victim':ab,ti OR 'wounded caregiver':ab,ti OR 'wounded healer':ab,ti OR 'secondary traumatic stress':ab,ti)	684 results
Scielo citation index through. Web of Science<br><br>Last updated: 2022/11/4	#1 AND #2 AND #3	("Health Services" OR "Health Facilities" OR "Healthcare" OR "Primary health care" OR "General Practice" OR "Family practice" OR "Ambulatory Care" OR "Nursing Care" OR "Family unit" OR "Hospitals") AND ("Program evaluation" OR "Support program" OR "Peer support" OR "Support strategies" OR "Organizational factors" OR "Organizational culture") AND ("Health personnel" OR "Physicians" OR "Nurses" OR "Doctor" OR "Practitioner" OR "Medical students" OR "Medical residents" OR "Healthcare providers" OR "Healthcare worker" OR "Healthcare staff" and "Error" OR "Near miss" OR "Adverse Event" OR "Clinical Error" OR "Medical error" OR "Second victim" OR "Wounded caregiver" OR "Wounded healer" OR "Secondary trauma")	146 results

Complete search on Epistemonikos<br><br>Last updated 2022/11/2	#1 AND #2 AND #3	(title ((((health services) OR (health facilities) OR (healthcare) OR (primary health care) OR (general practice) OR (family practice) OR (ambulatory care) OR (nursing care) OR (family unit) OR (hospital)) AND ((program evaluation) OR (support programs) OR (peer support) OR (support strategies) OR (organizational factors) OR (organizational culture)) AND ((health personnel) OR (physician) OR (nurse) OR (doctors) OR (practitioner) OR (medical students) OR (medical residents) OR (healthcare providers) OR (healthcare workers) OR (healthcare staff)) AND ((errors) OR (near miss) OR (adverse events) OR (clinical errors) OR (medical errors) OR (second victim) OR (wounded healer) OR (secondary trauma))) OR abstract:(((health services) OR (health facilities) OR (healthcare) OR (primary health care) OR (general practice) OR (family practice) OR (ambulatory care) OR (nursing care) OR (family unit) OR (hospital)) AND ((program evaluation) OR (support programs) OR (peer support) OR (support strategies) OR (organizational factors) OR (organizational culture)) AND ((health personnel) OR (physician) OR (nurse) OR (doctors) OR (practitioner) OR (medical students) OR (medical residents) OR (healthcare providers) OR (healthcare workers) OR (healthcare staff)) AND ((errors) OR (near miss) OR (adverse events) OR (clinical errors) OR (medical errors) OR (second victim) OR (wounded healer) OR (secondary trauma))))	106 results
Language		No language filter/restraint will be applied	
Period		No period filter/restraint will be applied	
Exclusion criteria		Article types not included: editorial , letter to the editor, cases series, case reports, narrative review, commentary	

Supplementary Table 2- Characteristics of the included studies

Authors (year of publication)	Type of study and level of evidence	Main aim of the study	Study design	Methods	Key findings	Quality assessment
Allen,Spencer, McEwan,Catarino , Evans, Crooks et al (2020)	Scientific article – Level VI[1]	To evaluate the experience of HCWs working in a mental health service after attending the Schwartz Rounds.	Mixed Method	Application of a quantitative evaluation form after the rounds and focus group and 6 years follow up.	Rounds were helpful, insightful and relevant to support HCWs in a non-blaming environment. The six-year follow-up revealed that the Rounds were still rated positively.	4*/5 of the MMAT criteria *there are no inconsistencies between results
Bryant (2022)	Graduate Theses, Dissertation s - Level VI[1]	To raise staff awareness on resilience, SVP and SupportingYOU intervention in a large academic children's hospital.	Quantitative descriptive	Application of preintervention and postintervention quantitative surveys,	Intervention increased the staff awareness on SVP, their perception on resilience and contribute for a proactive culture to manage critical incidents, by increasing the sense of feeling cared by the institution.	5*/5 of the MMAT criteria
Civil, Hoskins (2022)	Scientific article- Level VI[1]	To describe the design and implementation of a critical incident peer response team program at Waikato Hospital.	Quantitative descriptive	Application of postintervention quantitative survey.	Positive feedback from the attendants was given after the intervention. The program contributed to a supportive culture and enhanced teamwork.	2*/5 of the MMAT * 3 criteria were evaluated as "can't tell" : the sample is not representative of the target population, there is a risk of nonresponse bias; absence of statistical analysis
Edrees, Connors, Paine, Norvell, Taylor, Wu (2016)	Scientific article- Level VI[1]	To describe the development of RISE, initial evaluation of pilot programme and hospital-wide implementation at Johns Hopkins Hospital.	Mixed Method	Application of pre and post implementation quantitative surveys and focus group.	Although there were few calls in the first year of implementation, the rate of calls increased during the next years. Evaluation indicates the success of most encounters with callers and effectiveness of training to prepared the peer supporters to support second victim.	4*/5 of the MMAT criteria *there are no inconsistencies between results
Finney, Jacob, Johnson, Messner, Pulos, Sviggum (2021)	Scientific article Level VI[1]	To describe the implementation and evaluation of a SV program – Healing Emotional lives of Peers (HELP) in a Department of Anaesthesiology.	Mixed method	Application of post implementation quantitative surveys with open questions.	The program developed during 3 years was successfully implemented in the first 2 years of the program inception and is now a resource for other institutions in the region.	4*/5 of the MMAT criteria *no confounders accounted for in the design and analysis
Foreman (2014)	Paper Level VI[1]	To describe the development and implementation of a plan to help perinatal nurses to cope with stress after critical incidents and deaths in a family birth centre.	Qualitative	Qualitative description of the programme testing and the learning from its implementation.	Feedback on the use of the critical event plan has been positive. Nurses found the plan very useful after a tragedy on the unit related with the death of a new-born.	3*/ 5 of the MMAT criteria * 2 criteria were evaluated as "can't tell": qualitative data collection methods are not described; coherence between qualitative data sources, collection, analysis and interpretation is not clear;
Graham, Zerbi, Norcross, Montross-	Scientific article	To describe the Caregiver Support Team programme implementation in	Mixed-method	Application of baseline and post implementation (3 months follow up)	As an addition to the previous implemented Code Lavender intervention, the program was accepted and positively evaluated. Efforts will	4*/5 of the MMAT criteria *no confounders accounted for in the design and analysis

Thomas, Lobbestael , Davidson (2019)	Level VI[1]	an academic medical centre and evaluate its feasibility.		quantitative surveys . Self reported experiences of attendants.	be made to disseminate the program system-wide.	
Johnson, Simms-Ellis, Janes, Mills, Budworth, Atkinson ,Harrison (2020)	Scientific article<br><br>Level VI[1]	To evaluate a psychological resilience coaching intervention for National Health Services (NHS) trust sites and university premises.	Mixed-method	Application of pre and post implementation, follow up after the coaching phone call (10–20 days after the workshop) and 4–6 weeks after the workshop. Interviews application.	The program seems to be feasible and effective on improving general resilience of clinicians, by improving their knowledge and confidence in coping after adverse events.	5/5 of the MMAT criteria
Krzan, Merandi, Morvay, Mirtallo (2015)	Scientific article<br><br>Level VI[1]	To describe YOU Matter Support program implementation at Nationwide Children's Hospital.	Mixed-method	Application of pre and post (after 5 months) online surveys. Collection of peer encounters documentation of the Second Victim SharePoint site.	Most of the surveyed staff reported that the department benefited from implementation of the SV program. After the success of the pilot program, the hospital has decided to expand the YOU Matter program hospital- wide.	4*/5 of the MMAT criteria *no confounders accounted for in the design and analysis
Merandi, Liao , Lewe, Morvay, Stewart, Catt, Scott (2017)	Scientific article<br><br>Level VI[1]	To describe the replication of the ForYOU Matter program and expansion of the program to a large pediatric institution.	Quantitative descriptive	Description of the program implementation and of the collected data from electronic tracking system via SharePoint.	Application of the MUHC support model in the Nationwide Children's Hospital was validated and demonstrates that it's suitable and transferrable to other healthcare facilities and contexts.	3*/5 of the MMAT criteria<br>2 criteria was evaluated as "can't tell": no clear information if the risk of nonresponse bias was low: absence of statistical analysis
Lane, Newman, Taylor, O'Neill, Ghetti, Woltman, Waterman (2018)	Scientific article<br><br>Level VI[1]	To describe the Washington University School of Medicine Clinician Peer Support Program.	Quantitative descriptive	Description of the program development and its implementation.	Program was successfully implemented, however difficulties were found in sustaining the program over time, since peer supporters don't have protected time to be part of the program and therefore the programme activities have conflict with their operative schedules.	3*/5 of the MMAT criteria –<br>*2 criteria was evaluated as "can't tell": nonresponse bias was not applicable to this study; absence of statistical analysis
Mellins, Mayer, Glasofer, Devlin, Albano, Nash et al (2020)	Scientific article<br><br>Level VI[1]	To describe the application of CopeColombia programme in a Department of Psychiatry of a large urban medical centre's.	Mixed method	Description of the key issues/themes and facilitator responses emerged in the sessions (facilitator interventions);<br>post peer supporter group online survey (perceived impact of the program).	HCW emotional distress decreased after the program implementation. Peer Support Groups were the most used, valued and recommended. Sustainability of the program is critical due to financial constrains.	3*/5 of the MMAT criteria<br>*there are no inconsistencies between results;<br>1 criteria was evaluated as "can't tell": it's not clear if the study follows the traditional quality criteria of the qualitative methods involved
Merandi, Winning , Liao, Rogers, Lewe, Gerhardt (2018)	Scientific article<br><br>Level VI[1]	To assess healthcare providers satisfaction in the early implementation of a SV program in a group of neonatal intensive care units.	Mixed method	Application of pre and post implementation surveys with closed and open – ended questions. Thematic content analysis of the qualitative data from the survey.	This study suggest that peer support programs are likely to be viewed as positive and helpful for frontline HCW and managers. However, additional work should be done to assure the accessibility and effectiveness of the program.	4*/5 of the MMAT criteria *no confounders accounted for in the design and analysis

Rivera-Chiauzzi,Smith,Moore-Murray, Lee, Goffman, Bernstein, Chazotte (2022)	Scientific article<br><br>Level II[1]	To describe the development and evaluation a structured peer support program to address the needs of providers involved in obstetric adverse outcomes and to compare it with a routine support for HCW after the same type of events.	Pilot Randomized controlled trial	Application of needs assessment survey before program implementation. Surveys were applied at baseline, 3 months, and 6 months follow up.	Structured peer support program was successfully implemented with limited resources. The enhanced support group was significantly more likely to consider departmental leadership as one of the most helpful resources for support after adverse event. All participants refer were thriving at 6-month follow-up.	4*/5 of the MMAT criteria<br><br>*outcome assessors are not blinded to the intervention provided
Roesler, Ward, Short (2009)	Scientific article<br><br>Level VI[1]	To describe the recovery and reintegration of Neonatal Intensive Care Unit staff after a critical incident based on the Just Culture philosophy.	Qualitative: Single Case	Description of the protocol activation and application after the involvement of a severe adverse event.	The protocol was successfully applied and helped the affected HCW to thrive and return back to work after their involvement in the severe adverse event.	3*/5 of the MMAT criteria<br>*2 criteria were evaluated as “can’t tell”: qualitative data collection methods are not described ; it’s not clear if there is coherence between qualitative data sources, collection, analysis and interpretation
Schrøder, Bovil,, Jørgensen, Abrahamsen (2022)	Scientific article<br><br>Level VI[1]	To evaluate the Buddy Study program by assessing HCW experiences with having the program in the department, attending the compulsory seminar, and using a buddy or being activated as a buddy.	Mixed method	Application of quantitative questionnaires to assess HCW experiences of attending the Buddy Study seminar and participating in the Buddy Study program using a buddy or being activated as a buddy, along with two open-ended questions and three individual interviews for more elaborate answers.	The buddy study program was evaluated positively. It allowed to acknowledge the SVP and strengthen a supportive organizational culture by creating buddy relationships to support HCW after stressful events during care.	4*/5 of the MMAT criteria *no confounders accounted for in the design and analysis
Shapiro, Galowitz (2016)	Scientific article<br><br>Level VI[1]	To describe the development and implementation of Brigham and Women’s Hospital peer support program to support HCW after the impact of an emotional stressful event.	Quantitative descriptive	Description of the program and quantitative data collection of outreached calls and peer support sessions.	The peer support program has been implemented for 4 years and it’s expected to be expanded hospital-wide. The program does not yet reach many clinicians that might be in need of support after stressful events.	3*/5 of the MMAT criteria<br>*3 criteria were evaluated as “can’t tell”: it’s not clear if the sample is representative of the target population; risk of nonresponse bias low and statistical analysis are not applicable
Thompson, Hunnicutt, Broadhead, Vining, Aroke (2022)	Scientific article<br><br>Level VI[1]	To describe the implementation of SV support program in a large academic medical centre based on a quality improvement project.	Quantitative descriptive	Application the SVEST survey pre and post peer support program implementation.	Although the study didn’t find statistically significant differences in pre and post implementation the program has received a positive feedback among leadership members and peer supporters.	4*/5 of the MMAT criteria<br>*2 criteria were evaluated as “can’t tell”: low response rate in post-implementation survey may have decreased generalizability of results which can affect the sample representative ; potential for response bias was identified in the study

El Hechi , Bohnen, Westfal, Han, Cauley, Wright, Schulz et al (2020)	Scientific article<br><br>Level VI[1]	To describe the design and implementation of a SV surgical peer support program and its 1 year impact.	Mixed method	Programme design: literature review and multidisciplinary expert group discussion; Evaluation of the impact of the programme: quantitative and qualitative surveys application – evaluation of the peer supporters and of the program.	The first surgery-specific peer support program in US was successfully implemented. After 1 year experience, the program is highly used and well received.	4/5 of the MMAT criteria<br>*inconsistencies between quantitative and qualitative are not addressed in the study
Mira, Carrillo, Guilabert, Lorenzo, Pérez-Pérez, Silvestre et al (2017)	Scientific article<br><br>Level VI[1]	To describe the development and assessment of Mitigating Impact in Second Victims (MISE) programme on the awareness and information focused on the SVP.	Quantitative descriptive	Description of accreditation process; quantitative survey applied to 26 experts to assess structure and content; Quantitative survey applied to frontline HCW; evaluation of knowledge gained from the online program after each online module.	The online program was positively assessed by the accreditation agency, by the experts and the HCWs. The programme increases knowledge about patient safety, SVP and how to act after a severe adverse event. The time demand is reasonable for complete the course.	5/5 of the MMAT criteria
Scott, Hirschinger, Cox, McCoig, Hahn-Cover, Epperly et al. (2010)	Scientific article<br><br>Level VI[1]	To describe the deployment of ForYOU Team programme , an institutional rapid response system (RRS) for second victims at University of Missouri Health Care (MUHC).	Mixed method	Interviews and quantitative surveys application by the MUHC faculty and staff to support the development of the programme.	After identifying the need for support the SV in MUHC , the programme was designed, developed and successfully implemented. The programme has been integrated in the healthcare in the scheduled activities of the team leaders and clinical teams.	4*/5 of the MMAT criteria<br>*inconsistencies between quantitative and qualitative are not addressed in the study
Schuster (2021)	Scientific article<br><br>Level VI[1]	To describe the implementation of the HART programme and assess its impact on the Hematology/Oncology/Stem Cell Transplant Unit.	Mixed method	Preintervention and midpoint survey (after 3 months of pilot program), and qualitative subjective information collection from daily coach documentation entries.	HART programme was successfully implemented in the department and with high level of utilization from the staff. After implementation of HART, mental, emotional and physical wellbeing of all staff members improved.	4*/5 of the MMAT criteria<br>*inconsistencies between quantitative and qualitative are not addressed in the study
Calder-Sprackman; Kumar; Gerin-Lajoie; Kilvert; Sampsel (2018)	Scientific article<br><br>Level VI[1]	To describe the implementation, adaptation and evaluation of the ice cream rounds intervention in an emergency medicine training programme.	Quantitative descriptive	Application of a pre-implementation survey for needs assessment and a post implementation for feedback after intervention.	This Canadian initiative increased the overall perception of support and companionship, decreased feelings of stress, anxiety and burnout and can have a positive impact on the clinical practice in emergency medicine residents.	3*/5 of the MMAT criteria<br>*2 criteria were evaluated as “can’t tell”: it’s not clear if the sample is representative of the target population; absence of statistical analysis

Peterson, Bergström, Samuelsson, Åsberg, Nygren (2008)	Scientific article<br><br>Level II[1]	To evaluate the effect a reflecting peer-support group on self-reported health, burnout and on perceived changes in work conditions of multidisciplinary group of HCW in 3 hospitals of a county council in Sweden.	Randomized controlled trial.	Application of a baseline survey (7 months before the interview started), pre implementation questionnaire , immediately after the intervention ended (post intervention), 7 and 12 months after the intervention follow up.	The study showed positive intervention effects (after 12 months of the programme implementation) on overall health and decreased perceived work demands. A decrease in exhaustion, depression and anxiety symptoms was observed , as well as an increase in vitality.	5/5 of the MMAT criteria
Rubin, Rassman (2021)	Scientific article<br><br>Level VI[1]	To describe the social work led peer support model, COVID-19 Am I Resilient (cAIR), developed and implemented during the first wave of the COVID-19 pandemic in a large urban healthcare system.	Mixed method	Application of pre and post intervention surveys with quantitative and open ended questions.	The pilot programme cAIR was successfully implemented and provided support to normalize frontline HCW experience during Covid 19 pandemic, encouraged coworkers connections and developed coping skills . Participant engagement in cAIR were strong, however overall utilization of programme activities was low.	4*/5 of the MMAT criteria<br>*inconsistencies between quantitative and qualitative are not addressed in the study
Bernburg, Groneberg , Mache (2019)	Scientific article<br><br>Level II[1]	To develop, implement and evaluate the effectiveness of a mental health intervention programme on nurses' perceived job stress, perceptions of individual coping skills and the quality of patient relations in a group of psychiatric hospital departments.	Randomized controlled trial	Pre and post intervention surveys were applied using Perceived Stress Questionnaire; the Brief Resilient Coping Scale; the Self- Efficacy, Optimism and Pessimism Questionnaire; Emotion Regulation Skills Questionnaire and the German Quality of Relationship Inventory – at baseline , follow up after 3 months, 6 months and after 12 months.	After the intervention being successfully implemented, psychiatric nurses significantly increased their perception about job stress, emotion regulation skills, resilience, and self-efficacy; quality of patient-relationship were significantly higher ($p < 0.05$). The results indicate that self- care skills training may improve such as resilience and self-efficacy.	4*/5 of the MMAT criteria<br>*outcome assessors are not blinded to the intervention provided
Morales, Brown (2019)	Scientific article<br><br>Level VI[1]	To describe the development of the Care for the Caregiver Program in a 10 hospital system.	Qualitative	Description of the "Care for the Caregiver" programme and of practical scenarios after the programme was been activated.	Both described scenarios of support after stressful events indicate that the programme has been useful and well received by the clinicians.	4*/5 of the MMAT criteria<br>* 1 criteria was rated as can't tell : coherence between qualitative data sources, collection, analysis and interpretation is not applicable in this study
Cobos-Vargas, Pérez-Pérez, Núñez-Núñez, Casado-Fernández, Bueno-Cavanillas (2022)	Scientific article<br><br>Level VI[1]	To describe the Procedure for Serious Adverse Events (PSAE), integrated into a sentinel event operational procedure and to describe its accumulated results over two years of implementation in a university hospital.	Mixed method	Collection of quantitative data related with programme application, characteristics of involved HCWs and adverse events; HCWs qualitative feedback on programme improvement solutions and	The programme had a positive impact on patient safety culture and it increased the adverse event reporting in the hospital. The level of programme acceptance was high and HCWs valued to be individually interview after their involvement in the event and to contribute for future improvement actions.	4*/5 of the MMAT criteria<br>*inconsistencies between quantitative and qualitative are not addressed in the study

				their experience after attending the programme.		
Hinzmann, Forster, Koll-Krüsmann, Schießl , Schneider, Sigl-Erkel et al (2022)	Scientific article<br><br>Level VI[1]	To evaluate the burden, benefits, and mechanisms of action of a telephone support service for HCWs (PSU-HELPLINE).	Mixed methods	Application of two surveys: i) quantitative survey during the call(socio-demographic data, current stress ,relief from the counselling interview); ii) meta-questionnaire (to document the call and for quality assurance proposes) . Memory transcripts for thematical analysis were also collected.	The PSU-HELPLINE was mainly used for processing serious events and in phases of overload. The programme was well received by a specific region of Germany and callers consider it useful and supportive service.	4*/5 of the MMAT criteria<br>*inconsistencies between quantitative and qualitative are not addressed in the study

Supplementary Table 3 - Characteristics of the included interventions

Note: We have contacted the authors to complete the missing information, however some of the authors didn't reply to our contact.

**Author (Year of publication)**	**Starting year of the intervention**	**Duration of the intervention**	**Country and Setting**	**Target population**	**Aim of the intervention**	**Main outcomes related with the intervention**
Allen,Spencer, McEwan,Catarino, Evans, Crooks et al (2020)	Missing data	6 months	UK - Mental health service, inpatient acute psychiatric unit	Nursing care, healthcare assistance, occupational therapy, psychiatry, psychological assistance, domestic support building states, administration services, crisis resolution , home treatment team and perinatal mental health team	To understand experiences of emotional distress and reflecting on its impact and ways of coping	**i) Evaluation of Schwartz Rounds*:** From an average evaluation form response was 3.42 out of 4;<br><br>**ii) Experience of the participants after attending Schwartz Rounds:** In the Rounds, HCWs could express their emotions in an non-judgemental and empathic environment. Rounds were considered helpful, insightful and relevant;<br><br>**iii) Long-term follow-up of 6 years**: Rounds were still rated positively.<br><br>*Based on the following criteria: plan to attend the Rounds again, gained insight into thoughts and feelings, facilitator helped the discussion, the overview and presentation of the Rounds were helpful, it helped to improve the relation with colleagues in the work, the knowledge was useful for caring patients, the case was relevant to the clinical work
Bryant (2022)	2021	13 months	US - Intensive care unit, lactation services, child-life services, paediatrics medical surgical units	Staff members from lactation services and paediatric medical-surgical units. child life specialists, physicians, nurses, respiratory therapists, social workers, clinical associates, pharmacist and chaplains	To raise awareness of SVP and SupportingYOU. To increase resiliency by establishing a proactive culture to manage critical incidents prior they occur	**i) Staff perception on resiliency**: 100% of the surveyed staff reported an enhanced perception of resilience (p<0,001, CI 95%);<br><br>**ii) Staff awareness of SVP** significantly increased (p<0,001, CI 95%);<br><br>**iii) Staff awareness of SVP resources and of SupportingYOU**: it was limited since that most of participants were already aware of it;<br><br>**iv) Sense of preparedness in handling critical incidents**: there was an increased sense of managing emotional response after critical incidents among the participants (p<0,01, CI 95%);<br>**v) Feeling cared by the institution –** participants felt care for in the workplace (p<0,01, CI 95%).
Civil, Hoskins (2022)	2018	36 months	New Zealand – Department of Anaesthesia and Pain Medicine	Anaesthetists, surgeons, anaesthetist technicians, theatre nurse coordinator/charge nurse; theatre and recovery room nurses and midwifes	To provide peer-led group psychological first aid to full theatre team stressful events?	**i) Number of participants that attended the defuse:** over 200 members;<br><br>**ii) Number of interventions** : 28 defuse interventions have been delivered;<br><br>**iii) Participants feedback of the defuse** : Defuses were rated through neutral to very helpful, all surveyed participants would attend a defuse again in the future and would recommend to colleague or a friend.

Edrees, Connors, Paine, Norvell, Taylor, Wu (2016)	2011	52 months (from november 2011 to march 2016)	US- Johns Hopkins Hospital	All hospital staff	To provide timely psychological first aid and emotional support after critical incidents based on a call system	**i) Frequency of encounters:** 119 encounters from November 2011 and march 2016;<br>**ii) Caller interaction:** rated as excellent 66,7% or neutral (22,8%).
Finney, Jacob, Johnson, Messner, Pulos, Sviggum (2021)	2018	23 months (from July 2018 to June 2020)	US- Department of anaesthesiology at a large academic institution in the Midwest	Anaesthesiologist, certified nurse anaesthetics , residents, students registered nurse anaesthetics	To provide three-tiered support psychological first aid : first level given by peer and leadership at the local, second level provided by trained peer supporters; third level was provided by external services (from the institution or outside the instituition)	**i) Experience after receiving peer support:** From 31 surveyed participants, 25 (80.0%) evaluated the received support as "extremely" or "very beneficial", and 28 (90.3%) referred that were "extremely" or "very satisfied" with their experience; 30 (96.8%) would recommend HELP programme to a colleague;<br>**ii) HELP Programme Activations for Peer Support:** 91 electronic activations were utilized to assist 179 HCWs (the most common events leading to HELP activation were intraoperative patient demise, cardiac arrest, and pediatric care);<br>**iii) Trained peer support self-assessments:** From 85 surveyed peer supporters, 81 (95.3%) felt satisfied with how the peer support encounter turned out; 19 (22.4%) felt that they needed additional training and experience, 80 (94.1%) felt comfortable with their knowledge and skills as a peer supporter.
Foreman (2014)	Missing data	Info missing	US- Family birth centre Wilcox Women's Pavillon at Legacy Good Samaritan Hospital	All perinatal nurses of the family birth caring for women and newborns in all phases of childbearing	To create a critical event plan and implement it in the centre for helping staff to organize and manage critical events	**i) Experience on the use of the Critical Event Plan:** Nurses found it very useful and helpful**.**
Graham, Zerbi, Norcross, Montross-Thomas, Lobbestael , Davidson (2019)	Missing data	3 months	US- 4 target units in an academic medical centre: telemetry unit, emergency department, neonatal ICU and medical and neurologic ICU	Staff and physicians employed in the units	To provide emotional first aid in the workplace after critical events	**i) Programme activation :** 38 activations of the programme;<br>**ii) Experience after the programme:** all surveyed staff found it helpful and would recommend it to other colleagues; HCWs referred an improvement in feeling cared-for and also noted the sense of safety at the workplace;<br>**iii) Quality of life assessment and job satisfaction**: No significant changes were demonstrated before and after the intervention in Quality ofLife Scores or job satisfaction. One suicide was prevented;<br>**iv) Organizational changes :** Debriefings started to be requested after significant events affecting the entire department.
Johnson, Simms-Ellis, Janes, Mills, Budworth, Atkinson	2018	7 months	UK- National Health Services (NHS) trust sites and University of Leeds	HCWs or students that complete an education programme: midwives, paramedics , obstetrics and gynaecology trainee	To strengthen resilience and preparedness in dealing with adverse events : more flexible thinking, higher self-	**i) Experience after the programme:** workshop was useful, relevant and adequate in length**.** The participants highly valued the peer learning, the level of engagement and the format of the workshop delivery. The coaching call was critical to the consolidation of the knowledge and to

,Harrison (2020)				doctors , paediatric trainee doctors, paediatric consultant, physician associate students and sonography and mammography students.	esteem, better explanatory style	understand how to apply the acquired skills in practice. Knowledge about coping strategies led to a significant increase in self-perceived resilience, as well as confidence in coping with adverse events.
Krzan, Merandi, Morvay, Mirtallo (2015)	2013	5 months	US- pharmacy department at Nationwide Children's Hospital (NCH): one main inpatient pharmacy, inpatient pharmacy satellites, two outpatient pharmacies and home care pharmacy	Staff from all areas of pharmacy services	To provide three-tiered support psychological first aid (based on Susan's Scott model) to support HCWs involved in adverse drug events, patient-related injuries, and other traumatic work experiences	**i) Activation of the programme:** 3 respondents personally used the programme and 11 had recommend it;<br><br>**ii) Experience after the programme :** 85% of the pharmacy (95 of 112 respondents) refer that the department had benefit from the programme's implementation.
Merandi, Liao, Lewe, Morvay, Stewart, Catt, Scott (2017)	2012	60 months (5 years)	US- Nationwide Children's Hospital (NCH)	Staff from Nationwide Children's Hospital (NCH): all inpatient units as well as urgent cares, outpatient primary care clinics, and ambulatory specialty clinics	To provide three-tiered support psychological first aid (based on Susan's Scott model) to support HCWs involved in adverse drug events, patient-related injuries, and other traumatic work experiences	**i) Activation of the programme:** 21 group encounters were documented since November 2013;<br><br>**ii) Quantitative description of peer encounters:** 62% of peer encounters occurred in the emergency department, and 8% in pediatric intensive care unit and cardiothoracic intensive care unit ; nurses have the highest number of peer support encounters. Patient death and emotional stress were the most common reasons for peer encounter.
Lane, Newman, Taylor, O'Neill, Ghetti, Woltman, Waterman (2018)	2014	33 months	US- Two Hospitals affiliates of the Washington School of Medicine:<br>Barnes -Jewish Hospital and St. Louis Children's Hospital	Doctors, junior doctors, physician associates, nurse practitioners, and registered nurse anaesthetists, trainees providing care in the inpatient and outpatient settings	To facilitate the support of clinicians who have been involved in an adverse event or another adverse outcome during medical care	**i) Programme activation:** 165 clinicians were referred to the programme;<br><br>**ii) Median number of interactions per month:** 4.8 referrals per month (Range 0-12).
Mellins, Mayer, Glasofer, Devlin, Albano, Nash et al (2020)	2020	3 months	US-Large urban , academic, tertiary care - Columbia University Irving Medical Centre (CUIMC)	All CUIMC clinical and non-clinical staff, residents and faculty students	To provide peer support after stressful situations and enhance resilience of HCWs	**i) Emotional distress assessment:** The average emotional distress had significantly decreased (*p*<0.05)**;**<br><br>**ii) Perceived helpfulness of the peer support group:** Perceived helpfulness was high (76% rating helpfulness as "quite a bit" or "extremely."); all respondents (with the exception of two) recommended the support group to a colleague.

Merandi, Winning , Liao, Rogers, Lewe, Gerhardt (2018)	2015	12 months	US-7 neonatal intensive care units (NICUs) from quaternary-care, paediatrics hospital	All staff from the 7 NICUs	To provide three-tiered support psychological first aid (based on Susan's Scott model) to support HCWs involved in adverse drug events, patient-related injuries, and other traumatic work experiences	**i) Attendance to the programme:** A small number of healthcare providers (n=6) used the program. Some HCWs (not quantified) did not find the programme accessible.<br>**ii) Satisfaction with the programme:** The majority of the participants (56.3%, n=9) reported moderate benefit of the programme. From 250 surveyed HCWs in the units, 73.2% indicated that the NICUs benefited at least a little from the SV peer support programme. The manager reported that the programme enhanced the team leadership.
Rivera-Chiauzzi,Smith,Moore-Murray, Lee, Goffman, Bernstein, Chazotte (2022)	2015	8 months	US- Department of obstetrics & Gynaecology and Women Health	HCWs and residents (physicians and nurses) who experienced an obstetric adverse outcome.	To provide peer support to HCWs and residents, who have experienced an obstetric adverse outcome, through phone calls	**i) Attendance to the programme :** Out of 34 programme activations, 23 participants were placed in the enhanced group and 19 were assigned in the routine group;<br>**ii) Use of support resources and perception of its helpfulness:** Peer support was the most common source of assistance (p<0.05); departmental leadership was considered one of the most helpful resources for the enhanced support group;<br>**iii) Identified barriers:** Time (P = 0.26) was reported as a constraint by participants in the enhanced group, and routine group reported privacy concerns (P = 0.39) and stigma (P = 0.12)**;**<br>**iv) Effect of the programme on the stage of recovery:** At 6-month follow-up, all participants in the enhanced group reported that they were thriving after the event;<br>**v) Duration of peer support interventions:** most participants required less than 3 months of support to recover from the event.
Roesler, Ward, Short (2009)	2006	No clear information (estimated time : approx. 6 months)	US- Neonatal Intensive Care Unit (NICU), Methodist Hospital of Indianapolis	Staff of the NICU	To provide an institutional incident response after a paediatric serious adverse event	**i) Staff and unit recovery:** The pharmacist technician and 5 out of 6 nurses affected by the incident were back to work after the adverse event (1 nurse didn't return to work for familiar reasons). None of the affected HCWs experienced job changes. Debriefing and healing sessions were essential to the staff unit overcome the situation;<br>**ii) Impact on work culture**- Reinforcement of the culture of disclosure after applying the protocol.
Schrøder, Bovil,, Jørgensen, Abrahamsen (2022)	2018	18 months	Denmark-2 departments at Odense University Hospital: Department of Obstetrics and Gynaecology (OB-GYN) and Svendborg	All midwives in the OB-GYN and all physicians at the IME	To provide one-on-one peer support to HCW following a stressful event, including adverse events	**i) Attendance to the programme:** Out of 156 respondents, 26 of them reported using one of their buddies during the study period**;**<br>**ii) Evaluation of the Buddy Study programme in the department:** The programme encouraged more attentiveness among participants towards one another following adverse events (n=82, 52.6%) ; have

			and Internal Medicine and Emergency Department (IME)			contributed to more inter-collegial talks about adverse events (n=57,36.5%) ; sense of more openness to talk with colleagues about feelings in the aftermath of adverse events (n=65, 41.7%) ; increased willingness to ask for leadership support (n=53, 34.0%);<br><br>**iii) Overall experience with the programme:** an open and compassionate culture was encouraged; increased attentiveness to the staff wellbeing ; increased sense of safety (n=91, 58.3%).
Shapiro, Galowitz (2016)	2012	47 months (January 2012 and December 2015)	US- Brigham and Women's Hospital (BWH)	HCWs of Brigham and Women's Hospital	To provide one-on-one peer support or group peer support (if a team is likely to be affected), after a stressful event, including adverse events	**i) Activation of the programme:** 220 outreach calls to one-on-one peer support;<br><br>**ii) Attendance to the group sessions:** 240 clinicians participated in multidisciplinary group peer support sessions.
Thompson, Hunnicutt, Broadhead, Vining, Aroke (2022)	May, 2020 through October, 2020	1 month (trial period)	US – Anaesthesia department of the large academic centre	Certified registered nurses of the anaesthesia department.	To provide a peer support program to decrease SV distress after stressful events	**i) Frequency of the encounters:** Over the course of one month, a total of 8 peer support encounters were reported;<br><br>**ii) HCWs' distress**: It wasn't possible to observe a significant change in distress level after one month period, however the study found a statistically significant relationship between increased distress and insufficient colleagues support.
El Hechi , Bohnen, Westfal, Han, Cauley, Wright, Schulz et al (2020)	Missing data	12 months	US-Department of surgery at a tertiary academic medical centre	Surgeons and surgical trainees from general surgery, transplantation, pediatric surgery, thoracic surgery, cardiac surgery, vascular surgery, and trauma/acute care surgery.	To provide a peer support - based on three-tiered support psychological first aid - to surgeons and surgical trainees dealing with intraoperative adverse events, catastrophic patient outcomes, and/or long- term litigation cases.	**i) Programme activation:** 47 outreach calls;<br><br>**ii) Evaluation of the programme impact:** The majority of surgical staff was satisfied with the following domains – programme's confidentiality (89%), safe/trusting environment (73%,), timeliness of the intervention (83%). It was also perceived a positive impact on the department's culture, as it raised awareness about the importance of supporting colleagues facing difficult situations at work, and fostered a general sense of "safety and support" in the workplace.
Mira, Carrillo, Guilabert, Lorenzo, Pérez-Pérez, Silvestre et al (2017)	2015	15 months	Spain – without a specific location (online programme)	HCWs from hospitals and primary care that access the webpage of the online programme	To provide an online preventive programme to mitigate the impact of severe adverse events in HCWs and raise awareness about the SV phenomenon.	**i) Knowledge improvement (main outcome):** After completing the informative and demonstrative package, users significantly improved their knowledge about patient safety terminology, impact and prevalence of adverse events, SV support strategies and recommended actions following a severe adverse event (*P*<0.001).

Scott, Hirschinger, Cox, McCoig, Hahn-Cover, Epperly et al. (2010)	2009	10 months	US-University of Missouri Healthcare	University of Missouri Health Care faculty and healthcare staff	To provide a peer support programme to facilitate the second victim's transition through the six stages of emotional recovery	**i) Frequency of the encounters**: 49 encounters with forYOU Team members ,13 of which involved referrals to external support; there were 6 team debriefings with an average of 15 HCWs each. The average duration of the encounter with forYOU Team members lasted 30 minutes, while debriefings extended to 77 minutes.
Schuster (2021)	End of 2019	6 months	US- Department of Hematology/Oncology /Stem Cell Transplant Unit- Boston Children's Hospital – 2 separate units: a 30-bed hematology/oncology unit and a 14-bed stem cell transplant unit. Both units are under one hematology/oncology/ bone marrow transplant department within the inpatient pediatric free-standing children's hospital.	Multidisciplinary staff caring for hematology/oncology patients and their families (registered nurses, advanced practice nurses, clinical assistants, physicians, dieticians, child life specialists, environmental services, food tray distributors, patient experience representatives, social workers, resource room staff, chaplains, pain service staff, supply restockers, and students on the floor).	To provide peer to peer support program to promote a safer, supportive and resilient workplace culture, improve staff wellbeing and decrease the frequency of contacts among colleagues for work-related support outside of working hours to cope with stressful situations	**i) Frequency of the programme interventions:** 98 HART shifts;<br><br>**ii) Outcomes related with HART application:** improvement of HCWs' well-being, the number of HCWs reaching out coworkers for support outside working hours decreased after implementing HART; number of breaks increased during the work shifts, the use of hospital resources increased after HART coaches recommend them;<br><br>**iii) Satisfaction with the work-related support after HART implementation:** 25.6% of participants reported to be extremely satisfied with the received support; 49.4% of HCWs felt more supported by leadership.<br><br>**iv) Interactions resolutions:** The majority of interactions were solved in real time (83.2%);<br><br>**v) Subjective Feedback:** The programme fostered a safe, supportive and open work environment. HCWs felt more connected to their colleagues in the workplace.
Calder-Sprackman; Kumar; Gerin-Lajoie; Kilvert; Sampsel (2018)	2014	24 months	Canada – Department of emergency medicine , university of Ottawa	Residents from the emergency department at the university of Ottawa	To provide a peer-support sessions to improve residents wellbeing and create a supportive and resilient workplace	**i) Perception of change after the support programme**: From a total of 20 surveyed HCWs, 95% referred that support and companionship among residents increased after the support the sessions; 58,8% gained an increased awareness of coping strategies to deal with challenges during residency ;<br><br>**ii) Impact of the Rounds on clinical practice:** Half of HCWs referred that the rounds helped them to reflect about their clinical practice, a quarter of HCWs expressed that the rounds had not significantly impacted their clinical practice. Nevertheless, they acknowledged the value of having this type of support available to them;

						**iii) Perception of stress, anxiety and burnout**: 20% of HCWs decreased sensation of burnout and 10% decrease their perceived stress and anxiety;<br><br>**iv) Programme recommendation:** 89% of respondents would recommend ice cream rounds to other colleagues
Peterson, Bergström, Samuelsson, Åsberg, Nygren (2008)	2002	2 months	Sweden- 3 hospitals of the county council	Physicians, registered nurses, nursing assistants, social workers, occupational therapists, physiotherapists, psychologists, dental nurses and hygienists, dentists, service staff, administrators, teachers and technician. participants scored above the 75th percentile on the exhaustion dimension of the Oldenburg Burnout Inventory	To provide a reflecting peer-support group to discuss and reflect on work-related stress and burnout; to help to find out alternative ways to handle perceived stressful situations on an individual level; to provide an opportunity for mutual support between colleagues, to share and compare experiences and learn from each other.	**i) Frequency of the encounters:** 8 peer-support groups with 5–8 participants in each group**;**<br><br>**ii) Perceived impact of the peer support group:** The space for reflection on ‘real problems’ was valued by the participants; increased knowledge about stress and coping strategies, increased sense of belonging/community; Increased self-confidence;the existence of structured group was appreciated, decreased stress symptoms and anger; behavioural change; sleep improvement;<br><br>**iii) Work-related symptoms and burnout:** Both groups showed an overall decrease from baseline to follow up after 12 months in exhaustion, disengagement, depression and anxiety measures;<br><br>**iv) General health and vitality:** both increased after the intervention;<br><br>**v) Change in work conditions**: statistical significant difference was found in participation at work and support at work after 12 month follow-up.
Rubin, Rassman (2021)	2020	“During spring”	US-large urban healthcare system: Clinical education and practice department in Swedish Health Services	Clinical and non-clinical staff (nurse educator, professional development specialist, programme manager, student intern) and disciplines (nursing, social work) within the Clinical Education and Practice department.	To provide informational and emotional support to help frontline staff thrive in the first wave of the Covid 19 Pandemic	**i) Programme utilization:** 13 out 71 HCWs participated in the programme;<br><br>**ii) Evaluation of programme’s resources:** Approximately 85% of surveyed HCWs agreed or strongly agreed that there were adequate resources to support the HCWs during and after stressful events in the healthcare organization. All respondents agreed that assisting the cAIR video presentation was a valuable use of their time. The nature of the content and dedicated time for staff support stood out as the most helpful aspects of the video presentation. Most respondents improved their knowledge and/or skills with the cAIR resources.

Bernburg, Groneberg , Mache (2019)	Missing data	3 months	Germany - psychiatric hospital departments	Nurses full time working in psychiatric hospital department	To provide a mental health promotion intervention to develop self-care skills in psychiatric nurses	**i) Programme's attendance:** 44 nurses were part of the intervention group and 42 nurses of the control group;<br><br>**ii) Perceived job stress:** intervention group perceived lower levels of stress after attending the programme;<br><br>**iii) Relation with patients**: After the programme, significant improvements in nurses' relationship with their patients and lower levels of perceived conflicts were observed ($p < 0.05$);<br><br>**iv) Emotion regulation:** Large effects on emotion regulation skills were found in the first follow up (3 months) and medium effects sizes were found after 6 and 12 months;<br><br>**v) Resilience and self-efficacy:** statistically difference was found in these measures within groups (p<0.05)**;**<br><br>**vi) Final course evaluation:** Overall satisfaction with the training (1.39; 1-best score, 5-worse score); nurses displayed a strong level of motivation and interest in learning self-care techniques and applying them in their work. All participants verified that the training was worth attending (learning was meaningful and motivating for selfcare).
Morales, Brown (2019)	Missing data	No info	US- 10-hospital health system	Clinicians working in the 10-hospital system	To provide immediate emotional first aid after a serious adverse event and coach to HCWs on how to respond to patients and their families in a timely, empathetic, consistent, and patient-centred way.	**i) Process and outcome's results of two different scenarios have been described:**<br><br>**Scenario 1 (death after health condition deterioration of a patient affected all ICU nursing team) -** Process outcomes: programme activation was done by the nursing director; group sessions were applied to all nurses of the ICU , Employee Assistant Programme was provided to several nurses; Outcome results: after nurses had receive support from the programme, they described "feeling grateful for the opportunity to talk openly without judgment" about the event. Sessions also enabled for peer support among the nursing team members;<br><br>**Scenario 2 (intensivist involved in a stressful situation during intubation of a patient) -** Process outcomes: critical care unit director activated the programme; "Thinking of You" bag was delivered to the HCW in need. **Outcome results**: the intensivist expressed gratitude for both unit staff and members of the programme.
Cobos-Vargas, Pérez-Pérez, Núñez-Núñez, Casado-	2020	24 months	Spain- Clinico San Cecilio University Hospital	Hospital staff involved in any type of serious clinical incidents that caused or could have been caused (death or	To provide support to patients and their families, to HCWs and the healthcare institution after serious events, and to investigate and develop improvement	**i) Programme activations:** From 25 activations, 23 severe adverse events were investigated;<br><br>**ii) Frequency of the peer support encounters:** 1 to 17 HCWs per adverse event received trained peer support;

Fernández, Bueno-Cavanillas (2022)				serious harm of a patient)	actions, based on Susan's Scott model.	**iii) Time from the event until the activation of the programme:** ranged from 12h to one week;<br>**iv) Number of second victims identified:** 34.8% (n=47) were identified as second victims;<br>**v) Referral for third level of support** : 7 cases (14.9%) were referred, however 4 refused it;<br>**vi) Participants experience after participating in the programme:**increased sense of safety in the workplace; participants valued the interview provided in the second level of support and being able to contribute for the improvement actions after the adverse event occurrence;<br>**vii)Feedback for programme improvement:** The programme must be disseminated for all HCWs to have access to it; leaders should be trained to be more aware of the problem; first level of approach is identified has a problem in the units.
Hinzmann, Forster, Koll-Krüsmann, Schießl , Schneider, Sigl-Erkel et al (2022)	2020	24 months	Germany- health and emergency services	Clinicians and managers from the health and emergency services	To stabilize and restore the ability to act in highly stressful situation including adverse events, preserving clinical teams health and work capacity – in case of need , to transfer HCWs and managers with for specific treatment , initial structures of standard psychotherapeutic care in a low-threshold and timely manner.	**i) Evaluation of the programme:** In 81.4% of the cases the programme provided strong and very strong support to the callers in coping with the burden after a stressful event;<br>**ii) Follow up call after the first call :** 52.9% of callers needed a follow-up appointment with the same supporter after the first call;<br>**iii) Qualitative feedback after the programme** : there was an increased understanding of one's own reactions; openness to listen and understand that emotions are welcomed in a safe environment; knowing where to turn for help; enhanced mutual understanding and peer validation, as well as self-awareness and self-reflexion; leadership involvement increased.

Supplementary Table 4 – Main findings of the study according with the acronym OPERA

Main outcomes of interest	Main findings of the study
**O**rganisational factors<br>**P**eople<br>**E**nvironment<br>**R**ecommendations from previous studies on an organisational level<br>**A**ttributes of the interventions	Dissemination of the programme should be prioritised.
	Communication processes should be facilitated between HCWs and supporters.
	Specific training to peer supporters should be provided.
	Communication between the support team members should be regularly maintained.
	It's essential to create a multidisciplinary team with empathic skills.
	Leadership members should be actively engaged in both implementation and development of the programmes.
	A supportive and open organisational culture will benefit the programme implementation.
	To provide protected time and relief in staffing to participate in the programme (either for the programme's implementation team and for HCWs/SV that seek support) it's important for programme's sustainability.
	Evaluation of the support programme should be regular and overt time.
	Funding is important for programme sustainability.
	Programmes should be formally recognised in the instituition and have designated structures.
	Programmes should be easily accessible and on voluntary basis.
	Confidentiality should be ensured to facilitate HCWs' adherence and overcome potential barriers to participate in the programme.
	Programme's characteristics should be align with HCWs' needs.
