# Supplementary figures and images for: Key factors for effective implementation of healthcare workers support interventions after patient safety incidents in health organisations: a scoping review
Source: BMJ Open. 2023 Dec 27;13(12):e078118. doi: 10.1136/bmjopen-2023-078118 (PMC10753749; doi:10.1136/bmjopen-2023-078118)

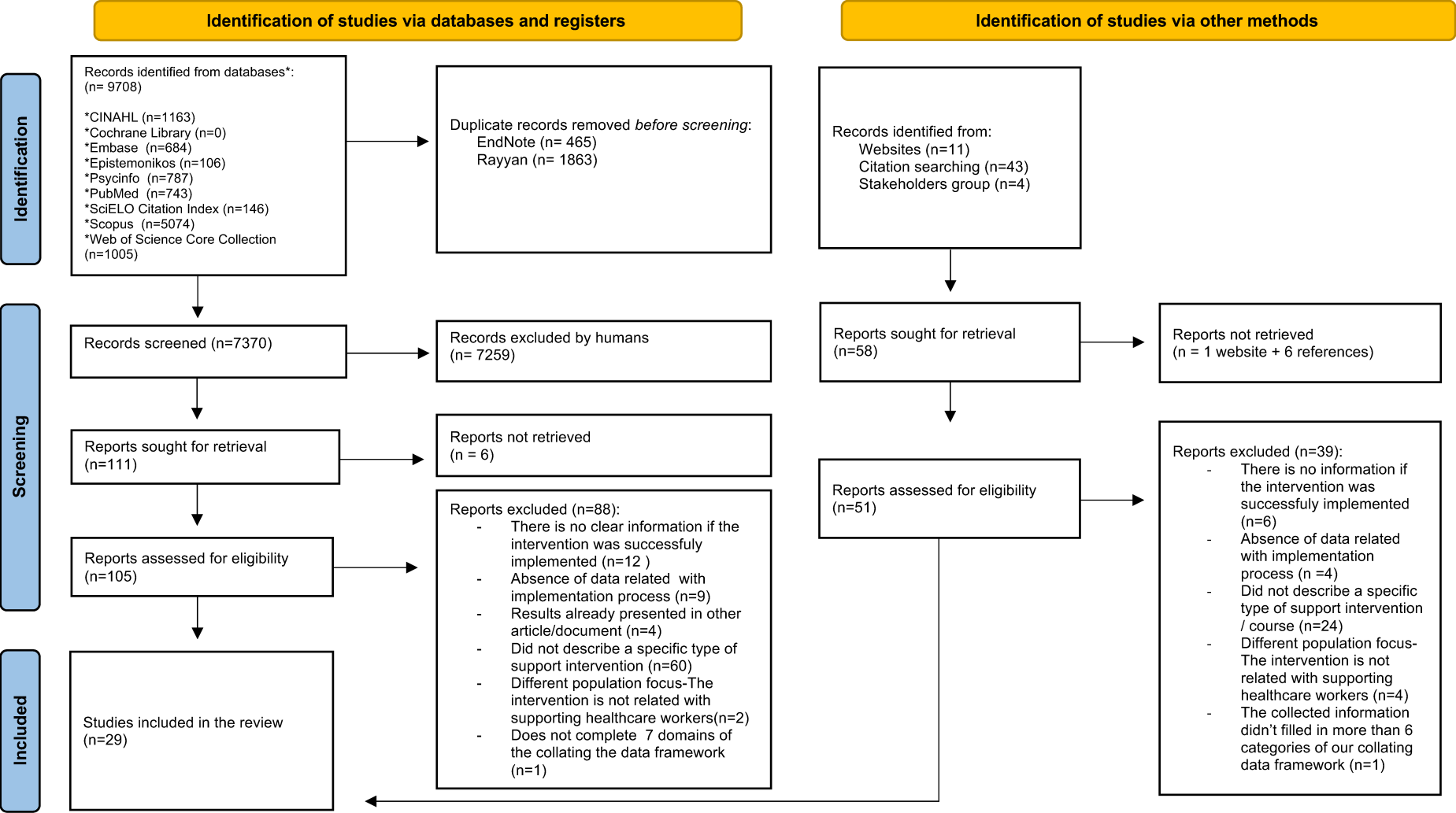

Supplement: Supplementary data [file bmjopen-2023-078118supp001.pdf]
