# Supplementary material for: Key factors for effective implementation of healthcare workers support interventions after patient safety incidents in health organisations: a scoping review
Source: BMJ Open. 2023 Dec 27;13(12):e078118. doi: 10.1136/bmjopen-2023-078118 (PMC10753749; doi:10.1136/bmjopen-2023-078118)
Supplement: Author's manuscript [file bmjopen-2023-078118.draft_revisions.pdf]

BMJ Open is committed to open peer review. As part of this commitment we make the peer review history of every article we publish publicly available.

When an article is published we post the peer reviewers' comments and the authors' responses online. We also post the versions of the paper that were used during peer review. These are the versions that the peer review comments apply to.

The versions of the paper that follow are the versions that were submitted during the peer review process. They are not the versions of record or the final published versions. They should not be cited or distributed as the published version of this manuscript.

BMJ Open is an open access journal and the full, final, typeset and author-corrected version of record of the manuscript is available on our site with no access controls, subscription charges or pay-per-view fees (http://bmjopen.bmj.com).

If you have any questions on BMJ Open's open peer review process please email info.bmjopen@bmj.com

BMJ Open

Key factors for effective implementation of healthcare workers support interventions after patient safety incidents in health organisations-a scoping review

Journal:	*BMJ Open*
Manuscript ID	bmjopen-2023-078118
Article Type:	Original research
Date Submitted by the Author:	27-Jul-2023
Complete List of Authors:	Guerra-Paiva, Sofia; National School of Public Health, NOVA University of Lisbon<br>Lobão, Maria João; National School of Public Health, NOVA University of Lisbon; NOVA Medical School<br>Simões , Diogo ; National School of Public Health, NOVA University of Lisbon<br>Fernandes, Joana; National School of Public Health, NOVA University of Lisbon<br>Donato, Helena; Centro Hospitalar e Universitário de Coimbra, Coimbra – Portugal, Documentation and Scientific Information Service<br>Carrillo, Irene; Miguel Hernandez University of Elche, Health Psychology; FISABIO,<br>Mira, José ; Miguel Hernandez University of Elche, Health Psychology; Salud Alicante-Sant Joan Health District,<br>Sousa, Paulo; National School of Public Health, NOVA University of Lisbon
Keywords:	PUBLIC HEALTH, Quality in health care < HEALTH SERVICES ADMINISTRATION & MANAGEMENT, Health & safety < HEALTH SERVICES ADMINISTRATION & MANAGEMENT, HEALTH SERVICES ADMINISTRATION & MANAGEMENT

SCHOLARONE™
Manuscripts

I, the Submitting Author has the right to grant and does grant on behalf of all authors of the Work (as defined in the below author licence), an exclusive licence and/or a non-exclusive licence for contributions from authors who are: i) UK Crown employees; ii) where BMJ has agreed a CC-BY licence shall apply, and/or iii) in accordance with the terms applicable for US Federal Government officers or employees acting as part of their official duties; on a worldwide, perpetual, irrevocable, royalty-free basis to BMJ Publishing Group Ltd ("BMJ") its licensees and where the relevant Journal is co-owned by BMJ to the co-owners of the Journal, to publish the Work in this journal and any other BMJ products and to exploit all rights, as set out in our licence.

The Submitting Author accepts and understands that any supply made under these terms is made by BMJ to the Submitting Author unless you are acting as an employee on behalf of your employer or a postgraduate student of an affiliated institution which is paying any applicable article publishing charge ("APC") for Open Access articles. Where the Submitting Author wishes to make the Work available on an Open Access basis (and intends to pay the relevant APC), the terms of reuse of such Open Access shall be governed by a Creative Commons licence – details of these licences and which Creative Commons licence will apply to this Work are set out in our licence referred to above.

Other than as permitted in any relevant BMJ Author's Self Archiving Policies, I confirm this Work has not been accepted for publication elsewhere, is not being considered for publication elsewhere and does not duplicate material already published. I confirm all authors consent to publication of this Work and authorise the granting of this licence.

For peer review only

Key factors for effective implementation of healthcare workers support interventions after patient safety incidents in health organisations-a scoping review

Sofia Guerra-Paiva[a,b], Maria João Lobão[a,b,c], Diogo Godinho Simões[a,d], Joana Fernandes[a], Helena Donato[e], Irene Carrillo[f], José Joaquin Mira[f,g], Paulo Sousa[a,b]

Corresponding author:

Sofia Guerra-Paiva[a,b],

https://orcid.org/0000-0002-4311-3799

Avenida Padre Cruz 1600-560 Lisboa

sg.paiva@ensp.unl.pt

a. Public Health Research Centre, National School of Public Health, NOVA University of Lisbon, Lisboa Portugal
b. Comprehensive Health Research Center, NOVA Medical School, Lisboa, Portugal
c. Internal Medicine Department, Hospital de Cascais, Cascais, Portugal
d. Public Health Unit of ACES USP Almada-Seixal, Almada, Portugal
e. Documentation and Scientific Information Service, Centro Hospitalar e Universitário de Coimbra, Coimbra, Portugal
f. Department of Health Psychology, Universidad Miguel Hernández de Elche
g. Alicante-Sant Joan Healthcare District, 03013 Alacant, Spain

Word count: 5443

For peer review only

ABSTRACT:

Background: Supporting healthcare workers (HCWs) and helping them to thrive after stressful situations can have an important impact on patient safety. In this study we set out to map and frame the key elements that underlie an effective implementation of HCW support interventions after patient safety incidents (PSIs), based on five key dimensions (organisational factors, relevant actors, environment, recommendations, operational attributes of the interventions).

Methods: This scoping review is guided by the six-stage approach proposed by Arksey and O'Malley and by PRISMA-ScR. We used the Mixed Methods Appraisal Tool (MMAT) Version 2018 to conduct a quality assessment of the eligible studies. The search strategy applied in this study was last updated in November 2022. The screening was performed between April 2022 and February 2023.

Results and discussion: A total of 9,708 records were retrieved from nine electronic databases, 43 articles were collected from the reference lists of the eligible articles, 11 from websites and four articles were included by the consultation group. We assessed 169 articles for eligibility in the full-text screening. Of these, 29 articles met the eligibility criteria. In this study we found that the existence of an organisational culture based on principles of trust and non-judgement, active leadership involvement, simplified processes and strong dissemination were crucial factors for the effectiveness of the programmes. Training should be provided for peer supporters and leadership to facilitate the response to HCW needs. Immediate and adequate multidisciplinary action after PSIs, ensuring the confidentiality of the process, was found to be important for facilitating HCWs' adherence . Funding and continuous monitoring are useful for the sustainability of the programmes.

Conclusions: HCWs' well-being depends on a strong organisational commitment in ensuring a complex group of interrelated factors to support them after PSIs, based on principles of trust and non-criticism.

Ethics and dissemination This study will not require ethical approval.

Trial registration number: 10.17605/OSF.IO/RQAT6.

Keywords: support programs; health personnel; second victim; clinical error; patient safety

view only

Strengths and limitations of this study:

- To the best of our knowledge, this is the first study focused on understanding the set of characteristics and elements necessary for a successful HCW support programme after a stressful event, such as a PSI;
- We used the MMAT assessment tool to evaluate the quality of the included studies, however some of the criteria couldn't be fully applied in some specific cases;
- We included five experts from five different countries to complement the literature search with additional sources of information;
- The inclusion and exclusion criteria were defined in accordance with a preliminary search strategy, guided by the population, concept and context (PCC), as recommended by the JBI for scoping reviews;
- Regulations focused on professional liability after a PSI were not considered in this study, however they might affect the effectiveness of the programmes.

For peer review only

For peer review only - http://bmjopen.bmj.com/site/about/guidelines.xhtml

INTRODUCTION

It is estimated that half of professionals will experience at least once in their career the second victim (SV) phenomenon defined as "any healthcare worker (HCW), directly or indirectly involved in an unanticipated adverse patient event, unintentional healthcare error, or patient injury, and who becomes victimized in the sense that they are also negatively impacted" (1).

The impact of a patient safety incident (PSI) on HCWs can seriously affect their quality of life (2–4), work satisfaction, confidence in their abilities (4) and work performance (3,5). It can result in turnover intentions and absenteeism (6) and in the most severe cases can lead to suicide (7). All of these consequences can seriously increase the burden on healthcare organisations. These types of healthcare incidents can either result in direct harm to the patient or, in the case of near misses, it may not cause direct harm but it still poses a potential risk to the patient's health (8).

In recent years there has been a growing number of publications describing the implementation of these types of programmes and practices all over the world with the overall aim of decreasing emotional and psychological distress in healthcare workers. A systematic review found that HCWs seek this type of support not only after being involved in a PSI, but also when facing other distressing situations (e.g. emotional distress, torpid evolution of a patient, personal crises, intraoperative mishaps) (9). Several studies mention that poor well-being in HCWs is associated with the occurrence of medical errors (10). Therefore, prioritizing interventions focused on improving HCWs' quality of life, mental and physical well-being, helping them to thrive after stressful situations can have a significant impact on patient safety (PS) and quality of care (10–12).

Although support interventions have demonstrated their benefits and utility, there is still limited research on finding what the common elements present in the development and implementation process of successful interventions are. A toolkit was introduced in 2010 to provide guidance on the implementation of programmes to support HCWs that have been negatively impacted by PSIs. The toolkit was organised into modules and specific actions focused on leadership, risk management, political and practical operational stages, communication and learning opportunities, safety culture and SVP awareness. The development of this toolkit was an important step in assisting with the implementation of healthcare support programmes and it can be adjusted to any type of healthcare organisation (13).

Evidence shows that establishing a set of elements for implementing interventions does not ensure that it will be introduced effectively in daily usage (14,15). Understanding what the factors are that might affect its effectiveness and sustainability and what the necessary implementation process is that will produce the expected effects is one of the main goals of implementation science (14). In this sense, learning from previous experience is an important aspect to consider and can facilitate practical application and contribute to more effective interventions (16).

Objectives:

In this study we set out to map and frame the key elements that underlie an effective implementation of HCW support interventions. We aim to understand what existing structures, organisational factors, relevant actors and operational attributes are present in healthcare support interventions, as well as to collect recommendations from previous studies, in order to identify what kinds of strategies are being applied in healthcare organisations and to guide future implementations. This set of interrelated dimensions were based on health policy implementation, implementation science principles and Donabedian's structure–process–outcome quality-of-care model, adapted by Yano (17).

METHODS

This scoping review is conducted using the six-stage approach proposed by Arksey and O'Malley (18) and is guided by the Preferred Reporting Items for Systematic Reviews and Meta-Analysis Extension for Scoping Reviews to ensure the transparency of the results obtained (19).

All the methodological steps are described in further detail in the scoping review protocol published in a previous publication (20).

For peer review only - http://bmjopen.bmj.com/site/about/guidelines.xhtml

Stage 1 : Identifying the research question(s)

The main goal of this study is to answer the following question:

- What are the key factors for an effective implementation of HCWs support interventions in health organisations after the occurrence of PSIs?

We have defined the following secondary questions to answer our main objective:

- What are the organisational factors that contribute for effective support programmes/other types of support interventions in health organisations when HCWs are physically and/or emotionally affected by PSIs?

- What are the operational attributes that contribute for effective support programmes/other types of support interventions in health organisations when HCWs are physically and/or emotionally affected by PSIs?

- Who are the most relevant actors that contribute for effective support programmes/other types of support intervention in health organisations when HCWs are physically and/or emotionally affected by PSIs?

- What are the contextual factors that contribute for effective support programmes/other types of support intervention in health organisations when HCWs are physically and/or emotionally affected by PSIs?

Stage 2: Search strategy

A comprehensive -search strategy using relevant electronic databases was developed with the support of a qualified research librarian. The search comprised Medical Subject Headings terms along with free-text keywords. We applied the search strategy in nine electronic databases and the last update was done in November 2022 (CINAHL, Cochrane Library, Embase, Epistemonikos, PsycINFO, PubMed, SciELO Citation Index, Scopus, Web of Science Core Collection). The applied search strategies in the electronic databases can be consulted in Complementary table 1. In addition to the database search, relevant websites were consulted and reference lists of the studies included in the full-text screening were screened to identify any other potential articles to include.

Stage 3: Study selection

The titles and abstracts of all retrieved documents were independently screened by two authors between April 2022 and February 2023. In the case of disagreement on article inclusion, a third author was involved to evaluate the paper independently and contribute to making a final decision.

In this study we didn't restrict the period of time or language of the included studies. Empirical studies (original articles) were prioritized along with systematic reviews and meta-analyses for collecting potential eligible studies. Grey literature (theses and other documents) were also considered eligible for the study.

The inclusion and exclusion criteria were defined in accordance with a preliminary search strategy, guided by the population, concept and context (PCC) (recommended by the JBI for scoping reviews (21)) and are further described in the published protocol of this study (20).

In this study we included support interventions in health organisations in which HCWs are physically and/or emotionally affected by PSIs and other distressing situations based on the fact that most support interventions end up being directed toward these types of programmes. We considered support interventions that were fully completed and executed in healthcare organisations, including measurable results that focused on assessing the attainment of an end state.

We focused on five key domains, guided by five main questions to study the implementation of these types of interventions and we organized them in the acronym OPERA (Figure 1).

Exclusion criteria

Editorials, letters to the editor, case series, case reports, narrative reviews and commentaries were excluded.

Stage 4: charting the data

A data extraction template was created to show the characteristics of the eligible studies (detailed information can be consulted in Complementary table 2).

Quality assessment

We used the Mixed Methods Appraisal Tool (MMAT) Version 2018 to conduct a quality assessment of the eligible studies (22). We believe that this appraisal will be important to enhance the quality and rigor of our study, ensuring greater transparency and validity of the data. The eligible studies were evaluated by two independent reviewers. A third reviewer was involved in cases of disagreement in the quality assessment.

Stage 5: collating, summarizing and reporting the results

The information from the eligible studies was collected and organised into different conceptual categories:

<u>- Organisational factors:</u>

a) *organisational structures* (e.g. infrastructures, resources, tools, equipment, units and staffing levels functional for managing and delivering services, leadership structure/authority and organisational culture);

b) *organisational processes* (e.g. organisational actions, procedures, recruitment criteria, training, programme implementation, communication processes, quality of interactions and coordination during programme implementation and dissemination as well as the sustainability of the practice);

c) *organisational outcomes* (e.g. implementation measures, process quality measures, utilization measures, effectiveness measures that assess the attainment of an end state);

<u>- Operational attributes of the interventions</u> (format/type of programme, accessibility, usability and confidentiality of the programme/intervention).

<u>- Relevant actors</u> (the individuals and organisations that actively participate in the development and implementation of the programme).

<u>- Contextual factors</u> (type of healthcare setting and cultural context).

<u>- Preferences and recommendations for the support programmes (</u>recommendations to improve or maintain the support interventions stated in the included studies).

Stage 6: consultation exercise and stakeholder involvement

We invited a group of five experts working on SVP research from five different countries (Finland, Germany, Italy, Portugal and Spain) to complement the literature search with additional sources of information. All of them are members of the European Network Working on Second Victims.

RESULTS:

A total of 9,708 records were retrieved from nine electronic databases, 43 articles were retrieved from the reference lists of the included articles, 11 from websites and four were collected from stakeholders' group inputs.

Based on the screening of titles and abstracts, 7,262 articles were excluded and 13 articles could not be retrieved after trying to contact the authors. A total of 169 articles were assessed for eligibility. Of these,

we excluded 140, and 29 articles ultimately met the eligibility criteria. Detailed information about the data collection, screening process, duplicates removed and reasons for exclusion is exhibited in the flow chart (complementary figure 1), in line with the original PRISMA statement (23)

Studies with levels of evidence IV and II (24) met the eligibility criteria. We have included the following types of studies: mixed methods (n = 15); quantitative descriptive (n = 8); qualitative (n = 3) ; randomized controlled trial (n = 2) (for further details about the included studies please consult Complementary table 2). Bearing in mind that we only included empirical studies, we didn’t include the first two screening questions in the MMAT evaluation (optional for MMAT) (22).

The characteristics of the included studies are outlined in Table 1.

Table 1- Characteristics of the included studies

**Categories**	**Subcategories**	**Number of articles**	**Total**
**Type of scientific article [1]**	Level VI - Evidence from a single descriptive or qualitative study	26	29
	Level II - Evidence obtained from Randomized Controlled Trial	3	
**Type of Study design**	Mixed Method	15	29
	Quantitative descriptive	8	
	Qualitative	3	
	Randomized Controlled Trial	3	
**Countries**	Denmark	1	29
	Germany	3	
	New Zealand	1	
	Spain	2	
	Sweden	1	
	UK	2	
	United States	19	

1.This rating scale is based on Ackley, B. J., Swan, B. A., Ladwig, G., & Tucker, S. (2008). *Evidence-based nursing care guidelines: Medical-surgical interventions.* (p. 7). St. Louis, MO: Mosby Elsevier (24).

Most of the programmes included in this study have a multidisciplinary application and were focused on supporting HCWs after traumatic work experiences directly associated with PSIs. Several programmes were particularly focused on responding to severe adverse events (25–27).

Although one-to-one sessions were the most commonly provided support, some programmes also included group sessions. In our study we also included interventions focused on raising awareness of SVP and creating a supportive and proactive culture to manage critical incidents and enhance HCWs’ well-being.

The included interventions are described in Complementary table 3.

In the following section we will describe the operational attributes of the programmes, organisational factors, relevant actors, contextual factors and other types of recommendations from the included studies.

Operational attributes of the programmes :

Accessibility:

The access of HCWs to the programmes was done by different channels to find the most convenient format for the users: phone call (28–32), email (30,31,33,34) or direct contact with peer supporters or with core team members (30,31,33).

For peer review

Programmes can be activated by the following people:

- anyone that was involved in the stressful event (26,35–39);

- safety and risk management staff (39,40);

- peer supporters (40);

- nurse in charge (41);

- leadership members (26,27);

- programme directors (36,40,42).

In some cases, the entire clinical team is contacted by the implementation team or by leadership members with a view to integrating in the support programme after a PSI, however acceptance only depends on the individual choice of the HCWs (26,42–44).

In the case of programmes that have online resources, they could be accessed through a website (25,45,46).

Although most of the programmes were provided on a voluntary basis, some of them have mandatory activities for all staff in the departments, such as a seminar to promote a shared understanding of SVP and the need for peer support (35), or attendance at debriefing sessions to enhance the recovery of all staff in the unit (27).

Programmes based on the Scott Three-Tier Model establish the access to the programme according to different levels of HCW needs (26,29,38). The first level of support should be available immediately after the incident has happened and team members should be prepared to provide it (local-level support). For accessing the second level of support, anyone can activate peer support with a trained peer. For the third level of support, the HCW is referred to specialized support (38).

In some cases, programmes can be accessed seven days a week (30–32,37,38,46,47). In New Zealand, the phone number to reach the support programme was entered into the staff contact list and on-call phones within the unit to facilitate the activation process (29). The dissemination of schedules, timelines and contacts was a useful strategy employed to facilitate access to the programme (37,46).

The identified barriers to accessing the programmes included the lack of staff awareness about the programme (28,48) and getting the time to attend the interventions (49). Moreover, some HCWs resisted accessing support since they did not recognize the need for it or preferred to avoid dealing with the situation again by talking about it (27).

Usability :

To ensure the maximal attendance of staff, in several programmes the communication process was facilitated by peer supporters or the programme director after the activation of the programme. These actors have the responsibility of contacting the HCWs immediately after the incident to schedule a one-to-one peer support encounter (35,36,40,42,43,45). In several programmes, the schedule was negotiated with HCWs according to their needs (29,39,40,42,43,45,50), and in some cases the location (45,50) and format (in person, phone call or email) of the sessions were also negotiated (39,42).

Staffing relief at the workplace and protected time were provided to allow HCWs to attend the support programmes during their working time (34,41,44,45,50,51).

To facilitate initiation of the programme, a leadership member or the programme director matched the peer supporter profile with the HCWs' needs according to the different criteria, such as geographic proximity and the same medical speciality (39,42,45). In other situations, like in the Buddy Study Programme, the HCWs selected the peer supporter according to their preferences so they could contact if they needed support (35).

To facilitate the HCWs' participation in the programme, some programmes prioritized the virtual format (34,40,45,47) and limited the duration of sessions to 60 minutes (29,47,49–51).

A peer support intervention in Sweden defined the topics to approach in the sessions based on participants' suggestions and beliefs (44). This made it possible to attend to the needs of the participants and to promote their adherence to the interventions (44).

In online programmes like MISE we found that browsing, amount of time required to complete the activities and comprehension of the programme content were valued attributes to facilitate the usability of the programme (25).

Organizational factors – Structures

Resources:

We found four main types of useful resources utilized in the interventions according to different applications. This resources are described in detailed in Table 2.

Table 2: Resources identified in the included HCW support programmes

**Marketing and dissemination materials**	Print marketing materials: posters (29,49); handouts such as brochures and flyers (31,45,46) identification badge of peer support for easy recognition and quick reference cards (31) .<br><br>Digital marketing material: promotional videos (28) website (28,45,47) ; emailbox (34,37,47).
**Selfcare and wellbeing related resources**	Packets with aromatherapy (50,52); chocolate (27,39,52) and snacks (48,51); kind messages (27,48,52) self-care pockets with essential support resources and guidance for coping with normal grief responses (41), others contain a journal, a stress ball, and tissues (39); general mental and emotional wellness advices (35), and use existing resources (35).
**Functional resources for programme implementation**	Electronic mailbox (30,31,34,36,37,40,42,45,47,52) access to virtual zoom (34,47) and webEx platform (47); dedicated mobile phone/pager/ hotline/phone call system for peer supporter sessions (27,29,32,37,38,42,43); web-based collaborative administrative platform for sharing information and managing the program (45) such as Sharepoint (30,31); checklist of responsibilities for the development team (41); list of peer support schedules (41,46); peer encounter forms (30,37) secure database of outreach attempts (42)
**Educational resources (most of them are related with peer support training)**	Online training of psychological first aid (25,37) powerpoint presentations with voice narrations (25) training scenarios (33,38,39); videos (25,45,53); "Do's" and "Don'ts" list, self-affirmations resources (42,53) and specific facilitator's guide (47) made available to peer supporters for guidance during the encounters with SV/HCWs; tutorial for peer support facilitation (51);

Posters, brochures and flyers were the most widely used marketing resources. The most recent studies have an investment in administrative support resources such as the use of SharePoint, a collaborative platform for programme management (30,31,45).

Infrastructures:

The acquisition of materials and human resources was, in most of the cases, on a voluntary basis. However, some studies mentioned that the intervention received specific funding for acquiring resources (50,52).

The existence of a specific room for sharing information and emotions in privacy was referred to by two studies (27,41).

Organizational culture

We identified the following factors associated with the organisational culture that are facilitators of the implementation of HCW support programmes after PSIs:

- openness of the health organisation to innovation (46);

- implementation of previous initiatives that have contributed to the creation of a proactive organisational culture to manage PSIs (29) and to support HCWs after critical incidents/stressful situations and promote their well-being (34,36,50,52);
- the existence of formalized structures directed at fostering a PS culture, based on a just culture approach (27,36,52), and at supporting HCWs and enhancing their well-being (27,30,33,36);
- active involvement of leadership members in initiatives that support SVs and HCWs' well-being (27,36,37,42,45–47,50);
- existence of established policies promoting a supportive organisational culture (such as the application of paid time off after a critical incident occurs) (27) and organisational accountability for employees' support and well-being after PSIs (35).

We also identified some potential organisational barriers to the implementation of programmes:

- a lack of staff and leadership awareness regarding the support programmes for HCWs (28);
- the organisational culture doesn't prioritize PS and HCW support and doesn't disclose wellness problems (40,43,48).

Organizational factors – Process

We found that most of the implemented programmes had developed a needs assessment (30,35,37,43,45,51) and/or conducted a literature review (25,42,45) prior to the design and creation of the programme. The needs assessment makes it possible to adapt the interventions to the needs of the clinical teams and adjust them to the institutional context and culture in accordance with the most recent literature.
The team was recruited using three different methods: direct nomination of the team members based on their ability to provide support in an empathic way (29,36,37,42,50); votes from the clinical team (42,52); and on a voluntary basis (37,43,45).
An advertising campaign for the attention of all the staff that would benefit the programme and show how to activate the service was carried out in a large number of interventions (28,29,31,35,45–47).

We describe below some of the implemented communication strategies described in the included studies:

- Digital marketing: dissemination of the programme on computer screensavers (28) and digital communication through the institutional website.

- Internal communication: hospital magazine, newsletters or email (28,34,37,37).

- Networking: presentation of programme in divisional meetings (28,36,37,40,41,45) or in hospital-wide events and conferences (39,41).

- Involvement of the leadership members in the dissemination process (28,48) and some programmes have included unit-level champions (28).

- Previous staff training on the topic of SVP (28,45) including training for staff provides the first level of support after a PSI in the local (38).

Dissemination was also carried out for recruiting peer supporters to join the peer support programmes (30,36,42,45). Most of the peer supporters received specific training to prepare them for providing assistance to others (28–31,36–38,40,42,44–46,48,50–52).

Organizational factors - Outcomes

Most of the included studies collected outcomes related to evaluation of the programme. The frequency of programme activation was the most common outcome collected in the included intervention.

In the Table 3 we describe the outcomes evaluated in the included studies.

Table 3: Collected outcomes from the included interventions

**Outcomes related to support services utilization**	Frequency of the HCWs who attended the programme (29,32,36,52,53); frequency of programme activation (26,29–31,35–40,42,45,48,50) , average duration of the encounters (38) number of programme dropouts (25); median number of interactions per month

For peer review only - http://bmjopen.bmj.com/site/about/guidelines.xhtml

	(38,40,43); frequency of peer support encounters (28,43); number of HCWs that need external support (38).
**Evaluation of the programme by the peer supporters perspective**	Overall peer support satisfaction with the training (25,26,35,37,53); perception of acquired knowledge, meaningfulness, motivation and interest to learn more and apply the learning (33); satisfaction about how encounter end out (45), need for additional training and experience (45), feeling able to provide support and being comfortable with their knowledge and skills as a peer supporter (45).
**Evaluation of the programme by the user perspective (HCWs involved in PSI/SV)**	Overall satisfaction with the programme (25,48,50) knowledge/skills acquisition (25,34), usefulness of the contents (25), timeliness of the program (42) perceived helpfulness of the program (43,47); HCWs awareness of SVP phenomenon (46). qualitative experience after attending the programme (how much HCWs benefit from the programme) (25,29,30,35,41,44,45,48–53).
**Health-related outcomes**	Psychological and physical distress (37); emotional distress (47); perceived stress (32,33) anxiety and burnout (44,51) assessment of quality of life (52), perceptions of individual coping skills such us emotion regulation, self-efficacy and resilience (33,37,46,53).
**Work related outcomes**	Job satisfaction (52) turnover intention and absenteeism (37) return to work (27) confidence in coping with adverse events (53).

Sustainability of the programmes:

After implementing the pilot intervention, several projects have effectively expanded the pilot intervention to other departments (30), other healthcare facilities (45,49) or hospital wide (31,35). The full integration of the programme in the departments underlies the inclusion of the programme in the scheduled activities and in the available services of the institution (38).

Leadership support was an important factor for the implementation of the programme and its sustainability (28,29,37). Other programmes nominated unit champions to ensure the implementation of the programme and its sustainability by promoting a more supportive culture within the unit (31,40,43).

Regular meetings were found to be important to maintain the cohesion of the team over time (29,38,39,50). Annual courses and the implementation of an interactive virtual platform were important for the expansion of the workforce working in these programmes (29,45). The high level of motivation and interest of the team (42) and retention of peer supporters were given particular consideration for programme sustainability and this was associated with work meaningfulness, staff satisfaction, commitment, a high level of resilience and a high level of confidence as a peer supporter (28).

Some of the programmes were implemented in healthcare organisations where some PS initiatives and “culture-shifting interventions” had already taken place (28,31,46,50). In other cases they were integrated in major projects developed by the organisations (32,36,39). Both situations were considered potential facilitators for maintaining the programmes over time.

Funding was also an important aspect to consider for the sustainability of several programmes (36,40,47,49,50).

Relevant actors

The establishment of a multidisciplinary team for the development and implementation of the support programmes was common to all the programmes. This team was predominantly composed of leadership members (hospital administrators and unit leaders), front-line workers, academics and experts in quality and safety (25,26,28,30,40,41). In some cases, it also included chaplains (41), social workers (30,41) and legal department members (40).

Most of the programmes’ development and implementation were dependent on volunteer efforts. However, some programmes hired specific elements of the team, such as the programme directors and coordination members (32,36,38,46). Several studies highlighted the importance of these members in the programme activation process, particularly in matching the profile of peer supporters with HCWs’ needs and in the contact with peer supporters and outreach people in need of support (36,39,40,42,43). One programme

For peer review only - http://bmjopen.bmj.com/site/about/guidelines.xhtml

included contract freelancer work by psychotherapists to provide more specialized support (32) and other programmes remunerated peer supporters for their work (32,35,50).

Trained peer supporters are crucial for providing effective support for HCWs involved in PSIs. Specialized trainers from different types of backgrounds (such as psychology, nursing, quality improvement and PS, workplace wellness, legal services, executive sponsors, department representatives) provided workshops and seminars for peer supporters (30,42,52). In some cases, there was a specialist to facilitate monthly debriefing meetings for peer supporters to process their experiences and to provide support for coaches (30,50,52).

Most of the programmes also provided access to specialized external support that represents the third level of support in the case of programmes that follow the Scott Three-Tier Model (26,38,45,48,52). In other types of programmes, complementary support was provided by chaplains, social workers or Employee Assistance Programme (EAP) counsellors (27,30,41,45).

On a department level, unit leaders performed different types of essential functions by contributing to the development of the programme (42,50), participating in the recruitment of peer supporters (25,28,30,48), providing first-level support for HCWs in need (26), coordinating programme components and mentoring peer support team members within the facility (38).

Environment – contextual factors

On an internal level, we found that most of the programmes were implemented in large and academic hospitals, characterized by an environment with multiple and complex divisions (26,27,29,31,36–38,40,45–47), and with a high level of specialization (tertiary and quaternary care) (34,35,42,48,50). Some programmes were specifically implemented in stressful and busy environments, such as emergency medicine departments, intensive care units and psychiatric departments (31,33,46,48,51).

We found that in some cases the organisational environment was beneficial to the implementation of the programme, particularly when healthcare organisations were already working towards creating a more supportive environment for their staff and strengthening the safety culture (30,32,36,39,50,52).

We also found that previous occurrence of a very serious adverse event helped in recognizing the need to implement a programme to support staff in supporting them to cope after PSIs (27,28).

On an external level, several studies have mentioned that programme implementation was affected by the Covid-19 pandemic, such as the possibility of handling face-to-face encounters, and it also affected the data collection/monitorization process of the interventions (6,8,9,11,22,24,27).

Recommendations

Different types of recommendations for improving the programmes were mentioned in the included studies with the ultimate goal of achieving a more effective intervention. They were identified from the user perspective (HCWs in need of support after being involved in a PSI) and also from the perspective of the implementation process (described in Table 4).

Table 4: Main recommendations referred to in the included studies from both the user and the implementation process perspectives

Recommendations related with the implementation process	Recommendations directly related with users experience (Healthcare workers involved in PSIs/SV)
To allocate protected time for teams to implement the programme and actively participate in the tasks and training (26,45);<br><br>To simplify the processes (29);<br><br>To promote active involvement of leadership members facilitates the implementation of the programmes and contributes for staff engagement (25,27,29,35,36,38,39,45,48,51);<br><br>To actively involve the target group in the development of the programme and to conduct a needs assessment helps fostering	To allocate protected time for HCWs attend the programme activities and the support sessions (49,51,53);<br>Smaller groups are preferred for sharing experiences and support (49);<br><br>Appropriate timing for programme activation, ideally it should be immediately available to the HCWs after a stressful event (38) and adequate duration of the support (29,53);<br><br>To ensure a supportive management to help staff to cope with stressful situations and to direct them to support resources in

interest and adapt to the specific needs of the target population (44,51);	case of need (26,39,50,52,53), to enhance the level of education on staff resiliency in the services (46);
To promote training sessions and resources to increase managers awareness about the SVP and about the existing support programmes (39,48);	To establish an interdisciplinary support team to open the scope of support according with the different staff needs and background (28,30,31);
To set regular debriefings (in person or virtual meetings) to exchange experiences and to foster a culture of mutual support among the members of the programme's team (29,39);	To create a safe place for sessions (41,50–52);
To create a multidisciplinary support team to facilitate a comprehensive programme's development and address different areas for support while leveraging a range of expertise (40);	Participation in the process should be entirely voluntary and confidential (26,29,38,40,43,45,51,53);
Training should be provided to peer support training based on role play (26,28,39);	To increase program awareness for front-line staff and prepare them how to give first level of support (30,37,48);
To develop a list of key phrases that peer supporters can use in their interactions with SVs (28);	To make resources available and close to the staff, to make them easy to reach (36,41,48);
To invest in programme's dissemination and marketing for increasing HCWs' adherence to the programme (26,31,32,35,37,38,42,47,48);	Active surveillance in the units to identify potential SV (particularly in high risk environments) (38);
To evaluate the impact of the programme and monitor its longer term effects and drive continuous improvement (28,30,33,35,37,39,47,50).	To identify barriers and facilitators for HCW to seek mental health support should be identified (e.g. stigma, career concerns, protected time)(47);
Administrative framework should be ensured to support programme implementation (38);	To invest in creating an organizational culture that addresses and acknowledges clinicians' vulnerability, while promoting a supportive environment after stressful incidents (36,38);
Funding was an important facilitator for program development and implementation (31,47,50);	To involve legal and risk management departments to address concerns about confidentiality or related issues (26,27,40);
To use pre-existing structures , resources and adapt existing programmes to facilitate the development and implementation of the programme(31,35,39,45);	To ensure that SV have an adequate access to the programme, feel safe and not stigmatized when accessing resources (48);
To be formally recognized as an institutional program (26);	All HCWs involved in a critical incident should be contacted to receive support (29,40);
Having an electronic dashboard for sharing documentation and data collection (31);	To provide opportunities to reach the programme even when the HCW was not involved in an adverse event (42);
To invest in telehealth solutions to support HCWs in the workplace (47);	To provide a holistic support (e.g. incorporating integrative therapy techniques; cognitive based therapy) (33,50);
To develop an institutional policy to guide the management of the critical event and support the affected HCWs and patients (27);	
To analyze the economic impact of the programme_ (26,39).	
To integrate staff working in the unit in the programme's team, helps to understand the needs of the unit (50).	

DISCUSSION

The majority of the programmes included in this study are based on peer support. These types of programmes are founded on social support with the ultimate goals of increasing professional quality of life, decreasing emotional stress and ensuring patients' safety (53–55).

We found that these types of programmes should be voluntary, with easy access and widely disseminated in healthcare organisations. This will make it possible to provide immediate psychological first aid after a distressful event and to overcome obstacles related to a lack of awareness of SVP and stigmatization associated with HCW vulnerability (28,31,55–57).

For peer review only - http://bmjopen.bmj.com/site/about/guidelines.xhtml

An emphatic and clear communication between peer supporters and HCWs suffering from stressful situations has been identified as an important factor to consider when implementing this type of programme. A scoping review focused on peer support interventions after traumatic incidents identified several characteristics that should be considered when providing support (54). Willingness to give support, leadership skills, reliability, not being judgmental, empathy, and communicative and relational abilities are essential elements for effective peer support (54). Another study pointed out that the ability to understand others' feelings and experiences after a PSI can be beneficial to the support process, by improving emotional regulation and reinforcing the cooperation between HCWs and peer supporters (58).

The association between just culture and empathic and non-judgemental responses to error has been clear (28,59–61). This is identified as a core condition for an open communication, and to establish positive relationships between peer supporters and HCWs (61,62) to better identify potential SV and to increase programme adherence. In this regard, the legal framework of each country focused on professional liability is often not cited in articles describing interventions, despite its influence on programmes and its impact on transitioning from a reactive safety culture to a generative safety culture (63).

We found that specific training for peer supporters was provided in most of the programmes to prepare them to adequately provide psychological support according to HCWs' needs. Training is believed to be one of the key components to consider when implementing a support programme (28,31,55).

Regular multidisciplinary meetings are relevant procedures for sharing important learning and experiences and they help in keeping the team motivated over time. Rosak-Szyrocka (2015) points out that having a motivated team is very important to ensure their commitment to, and engagement in, work (64). This study also points out that in hospitals, HCWs are particularly motivated by strong interpersonal relations and a positive atmosphere, as they foster cooperation and mutual support among the team members (65). Another study indicates that multidisciplinary teamwork is an essential element for improving outcomes at an organisational level (66).

In our study, we found that leadership engagement in HCWs/SV support initiatives is very important for strengthening the organisational culture towards a non-punitive response to error. According to Boguslavsky, Gutierrez- and Holschneider (67), leaders should have good empathic and communication skills, and should be able to listen, empower and encourage others, in line with non-blame culture principles. Leadership members are essential actors in helping to create stimulating and supportive environments in healthcare among teams (65,68). The Joint Commission (2017) states that leaders' impact is very closely related to their daily actions, attitude and decisions rather than what they recommend (68).

We found that active participation by leaders in the initiatives can influence the effectiveness of programmes and their sustainability. Helping to create a safe and resilient environment can increase the programmes' acceptability among HCWs and their engagement in the activities (66,67,69,70). Leaders' participation was not only important in the implementation process, but also in the development of the programmes. In particular, it can contribute to making the programmes adequate in the healthcare context, facilitating the acquisition of resources and being an important actor in the recruitment of peer supporters.

Moreover, we also found that the effectiveness of these types of support programmes is also dependent on how closely aligned they are with HCWs' needs. Therefore, HCWs should be consulted and involved in the programme development process. Our results corroborate Søvold et al.'s findings, which highlight the importance of HCWs participating more in the decision-making process as well as in the development, implementation, testing and evaluation of the interventions, with the ultimate aim of improving their health, well-being and job satisfaction (71). Interventions that include the target population's perspectives from the first steps of development exhibit a higher level of adherence and adoption and are more sustainable over time (72). In summary, HCWs should feel that these types of programmes have a purpose in their lives (72).

For peer review only - http://bmjopen.bmj.com/site/about/guidelines.xhtml

However, HCWs are very often overloaded with duties that could be undertaken by other staff, and prevented from performing other necessary tasks that need their qualifications, with few opportunities to apply for training, develop their professionals skills and be available to participate in workplace initiatives (66). This contributes to less work satisfaction and more costs for the system (66). In our study, we found that working conditions are one of the main pillars for ensuring effective implementation and its sustainability. Providing protected time and staffing relief were identified as two of the main priorities for ensuring that, on the one hand, qualified HCWs could participate in the development and implementation of support interventions, and on the other, HCWs/SV would be able to attend the support programmes if they needed support.

According to implementation research, participants' responsiveness along with the degree of complexity of the intervention, the quality of delivery and training are key elements of an effective programme (72).

Evidence shows thar financial incentives play an important role in retaining HCWs and also in embracing HCWs' responsibilities in the long term (66,72). In some cases, are also given to clinical teams implementing the support programmes.

Finally, it is agreed that monitoring programme outcomes over time is essential for assessing programme effectiveness, and evaluating its progress and impact on HCWs/SVs and in healthcare organisations (73). This process allows the programmes to be adjusted to facilitate their ongoing improvement.

Although there is growing investment in monitorization programmes in hospital settings, the study of the impact of HCWs' support programmes is still very limited in non-hospital settings.

In hospital settings, we have found that programmes frequently monitor before and after interventions, by collecting both qualitative and quantitative measures. There are a limited number of studies that follow up interventions over time. According to Wade et al. (2022), it is still not clear how much time would be necessary to monitor the impact of programmes on HCWs' skills and knowledge (74). Thus, we recommend that future programmes invest in monitoring their results over time and for longer periods.

Wade et al. (2022) recommend that outcomes related to staff retention, staff sick time and the occurrence of serious safety events should be considered in monitoring the programmes. Our findings corroborate some of Wade et al.'s recommendations, and although the impact evaluation of support programmes is still limited, evidence shows that this has been an important aspect to consider in programme implementation.

In some programmes, we have identified confidentiality concerns during the evaluation of the programmes. Wade et al. (2022) highlighted the need to establish a consensus and build a body of evidence to evaluate these types of programmes in an ethical and confidential way that involves protecting the privacy and confidentiality of the attendees of these types of programmes (74).

LIMITATIONS

In this study, we have found that long-term evaluation of programmes is still limited for the most part, and in some cases, evaluating the outcomes is not recognized as a priority for reasons of confidentiality and ethics. We recognize the need to strengthen the organisational culture towards a non-punitive response to error in order to overcome potential barriers to programme adherence and evaluation. It is very important to increase the follow-up time to understand the impact of these types of programmes and their effectiveness in the long term.

We evaluate all the included studies using the MMAT quality assessment tool since this tool is suitable for different types of methodologies, however some of the criteria couldn't be applied in the descriptive studies. We suggest that this tool could be adapted in the future to these types of studies.

CONCLUSION

This is the first time that a study has focused on understanding the set of characteristics and elements necessary for a successful HCW support programme after stressful events, such as PSIs.

In summary, we concluded that programmes should be easily accessible and voluntary for all HCWs in healthcare organisations. The effectiveness of the programmes is highly dependent on the organisational culture, the active involvement of leadership, and the preparedness and quick response of the multidisciplinary teams that provide peer support. Training should be provided for both peer supporters and leadership, to make it possible to respond to HCWs' needs in a more prepared and satisfactory way. Regular communication among support teams should be maintained over time to keep teams motivated and increase their retention. Finally, programmes should be monitored for their continuous improvement without compromising the confidentially of the data.

We believe that our findings can make an important contribution to evidence-based practice and can guide future research and the implementation of HCWs support programmes in health organizations.

AKNOWLEGMENTS

This work was supported by ERNST – Cost Action 19113 (COST – European Cooperation in Science and Technology, www.cost.eu). We would like to particularly thank the members of stakeholder groups, namely Prof. Reinhard Strametz, Dr Susanna Tella and Prof. Massimiliano Panella, for their contribution to this study as members of the consultation group.

FUNDING

The present publication was funded by Fundação Ciência e Tecnologia, IP national support through CHRC (UIDP/04923/2020) and under the grant (UI/BD/150875/2021).

ETHICS AND DISSEMINATION

This scoping review does not require ethical approval. Results and conclusions of the study will be presented in scientific conferences as well as in international forums and through other relevant dissemination channels.

AUTHOR CONTRIBUTIONS

SGP was involved in the writing of the article. SGP, MJL, PS and JJM contributed to the conceptualization of the study. SGP, MJL, DGS participated in the pilot testing stage and defined the inclusion criteria. SGP, MJL, JDS, JF and IC were involved in the screening process of the eligible studies. HD contributed to the definition of a robust search strategy and participated in the preliminary search in relevant bibliographic databases, particularly in peer-reviewed journals and grey literature databases. PS, JJM and IC contributed to the literature review. PS and JJM also participated in the consultation group. All authors read and approved the manuscript.

COMPETING INTERESTS

This protocol is part of the PhD project of the corresponding author (SGP), which has been previously approved by the National School of Public Health (ENSP- NOVA). Some of the research group members are part of the European Researchers Network Working on Second Victims (ERNST) - COST Action 19113. All the authors have submitted the disclosure of interest form.

REFERENCES

1. Vanhaecht K, Seys D, Russotto S, Strametz R, Mira J, Sigurgeirsdóttir S, et al. An Evidence and Consensus-Based Definition of Second Victim: A Strategic Topic in Healthcare Quality, Patient Safety, Person-Centeredness and Human Resource Management. Int J Environ Res Public Health. 19(24):16869.

For peer review only

2. Rinaldi C, Leigheb F, Vanhaecht K, Donnarumma C, Panella M. Becoming a “second victim” in health care: Pathway of recovery after adverse event. Rev Calid Asist. 31:11–9.

3. Vanhaecht K, Seys D, Schouten L, Bruyneel L, Coeckelberghs E, Panella M, et al. Duration of second victim symptoms in the aftermath of a patient safety incident and association with the level of patient harm: a cross-sectional study in the Netherlands. BMJ Open. 9(7):e029923.

4. Waterman AD, Garbutt J, Hazel E, Dunagan WC, Levinson W, Fraser VJ, et al. The Emotional Impact of Medical Errors on Practicing Physicians in the United States and Canada. Jt Comm J Qual Patient Saf. 33(8):467–76.

5. Carrillo I, Tella S, Strametz R, Vanhaecht K, Panella M, Guerra-Paiva S, et al. Studies on the second victim phenomenon and other related topics in the pan-European environment: the experiences of ERNST Consortium members. Journal of Patient Safety and Risk Management. 2022;

6. Burlison JD, Quillivan RR, Scott SD, Johnson S, Hoffman JM. The Effects of the Second Victim Phenomenon on Work-Related Outcomes: Connecting Self-Reported Caregiver Distress to Turnover Intentions and Absenteeism. J Patient Saf. 17(3):195–9.

7. Scott SD, McCoig MM. Care at the point of impact: Insights into the second-victim experience. J Healthc Risk Manag. 35(4):6–13.

8. World Health Organization. Patient safety incident reporting and learning systems: technical report and guidance. Geneva: World Health Organization; 2020.

9. Busch IM, Moretti F, Purgato M, Barbui C, Wu AW, Rimondini M. Psychological and Psychosomatic Symptoms of Second Victims of Adverse Events: a Systematic Review and Meta-Analysis. J Patient Saf. 16(2):e61–74.

10. Hall LH, Johnson J, Watt I, Tsipa A, O’Connor DB. Healthcare Staff Wellbeing, Burnout, and Patient Safety: A Systematic Review. Harris F, editor. PLOS ONE. 11(7):e0159015.

11. Schiess C, Schwappach D, Schwendimann R, Vanhaecht K, Burgstaller M, Senn B. A Transactional «Second-Victim» Model-Experiences of Affected Healthcare Professionals in Acute-Somatic Inpatient Settings: A Qualitative Metasynthesis. J Patient Saf. 2021;17(8):e1001–18.

12. Schwappach D, Boluarte T. The emotional impact of medical error involvement on physicians: A call for leadership and organizational accountability. Swiss Med Wkly. 2009;

13. Pratt S, Kenny L, Scott SD, Wu AW. How to develop a second victim support program: a toolkit for health care organizations. Jt Comm J Qual Patient Saf. 2012;38(5):235–40.

14. Bauer MS, Kirchner J. Implementation science: What is it and why should I care? Psychiatry Res. 2020;283:112376.

15. Thoele K, Ferren M, Moffat L, Keen A, Newhouse R. Development and use of a toolkit to facilitate implementation of an evidence-based intervention: a descriptive case study. Implement Sci Commun. 2020;1(1):86.

16. Cook S, Mayers S, Goggins K, Schlundt D, Bonnet K, Williams N, et al. Assessing research participant preferences for receiving study results. J Clin Transl Sci. 2019;4(3):243–9.

For peer review only

17. Yano EM. The role of organizational research in implementing evidence-based practice: QUERI Series. Implement Sci. 2008;3(1):29.

18. Arksey H, O'Malley L. Scoping studies: towards a methodological framework. Int J Soc Res Methodol. 2005;8(1):19–32.

19. Tricco AC, Lillie E, Zarin W, O'Brien KK, Colquhoun H, Levac D, et al. PRISMA Extension for Scoping Reviews (PRISMA-ScR): Checklist and Explanation. Ann Intern Med. 2018;169(7):467–73.

20. Guerra-Paiva S, Lobão MJ, Simões JD, Donato H, Carrillo I, Mira JJ, et al. Key factors for effective implementation of healthcare worker support interventions after patient safety incidents in health organisations: a protocol for a scoping review. BMJ Open. 2022;12(8):e061543.

21. The Joanna Briggs Institute. The Joanna Briggs Institute Reviewers' Manual 2015 Methodology for JBI Scoping Reviews. Australia: The Joanna Briggs Institute; 2015.

22. Hong Q, Pluye P, Fàbregues S, Bartlett G, Boardman F, Cargo M, et al. Mixed Methods Appraisal Tool (MMAT), version 2018.

23. Moher D, Liberati A, Tetzlaff J, Altman DG, for the PRISMA Group. Preferred reporting items for systematic reviews and meta-analyses: the PRISMA statement. BMJ. 2009;339:b2535–b2535.

24. Ackley B, Swan B, Ladwig G, Tucker S. Evidence-based nursing care guidelines: Medical-surgical interventions. St. Louis, MO: Mosby Elsevier; 2007.

25. Mira JJ, Carrillo I, Guilabert M, Lorenzo S, Pérez-Pérez P, Silvestre C, et al. The Second Victim Phenomenon After a Clinical Error: The Design and Evaluation of a Website to Reduce Caregivers' Emotional Responses After a Clinical Error. J Med Internet Res. 2017;19(6):e203.

26. Cobos-Vargas A, Pérez-Pérez P, Núñez-Núñez M, Casado-Fernández E, Bueno-Cavanillas A. Second Victim Support at the Core of Severe Adverse Event Investigation. Int J Environ Res Public Health. 2022;19(24):16850.

27. Roesler R, Ward D, Short M. Supporting Staff Recovery and Reintegration After a Critical Incident Resulting in Infant Death. Adv Neonatal Care. 2009;9(4).

28. Edrees H, Connors C, Paine L, Norvell M, Taylor H, Wu AW. Implementing the RISE second victim support programme at the Johns Hopkins Hospital: a case study. BMJ Open. 2016;6(9):e011708.

29. Civil NM, Hoskins JD. Building a critical incident peer response team: A full theatre team welfare intervention. Anaesth Intensive Care. 2022;50(6):421–9.

30. Krzan KD, Merandi J, Morvay S, Mirtallo J. Implementation of a "second victim" program in a pediatric hospital. Am J Health Syst Pharm. 2015;72(7):563–7.

31. Merandi J, Liao N, Lewe D, Morvay S, Stewart B, Catt C, et al. Deployment of a Second Victim Peer Support Program: A Replication Study. Pediatr Qual Saf. 2017;2(4):e031.

32. Hinzmann D, Forster A, Koll-Krüsmann M, Schießl A, Schneider F, Sigl-Erkel T, et al. Calling for Help—Peer-Based Psychosocial Support for Medical Staff by Telephone—A Best Practice Example from Germany. Int J Environ Res Public Health. 2022;19(23):15453.

For peer review only

For peer review only - http://bmjopen.bmj.com/site/about/guidelines.xhtml

33. Bernburg M, Groneberg DA, Mache S. Mental Health Promotion Intervention for Nurses Working in German Psychiatric Hospital Departments: A Pilot Study. Issues Ment Health Nurs. 2019;40(8):706–11.

34. Rubin EW, Rassman A. cAIR: Implementation of peer response support for frontline health care workers facing the COVID-19 pandemic. Soc Work Health Care. 2021;60(2):177–86.

35. Schrøder K, Bovil T, Jørgensen JS, Abrahamsen C. Evaluation of'the Buddy Study', a peer support program for second victims in healthcare: a survey in two Danish hospital departments. BMC Health Serv Res. 2022;22(1):566.

36. Shapiro J, Galowitz P. Peer Support for Clinicians: A Programmatic Approach. Acad Med. 2016;91(9):1200–4.

37. Thompson M, Hunnicutt R, Broadhead M, Vining B, Aroke E. Implementation of a Certified Registered Nurse Anesthetist Second Victim Peer Support Program. J Perianesth Nurs. 2022;37(2):167–73.

38. Scott SD, Hirschinger LE, Cox KR, McCoig M, Hahn-Cover K, Epperly KM, et al. Caring for Our Own: Deploying a Systemwide Second Victim Rapid Response Team. Jt Comm J Qual Patient Saf. 2010;36(5):233–40.

39. Morales CL, Brown MM. Creating a Care for the Caregiver Program in a Ten-Hospital Health System. Crit Care Nurs Clin North Am. 2019;31(4):461–73.

40. Lane MA, Newman BM, Taylor MZ, O'Neill M, Ghetti C, Woltman RM, et al. Supporting Clinicians After Adverse Events: Development of a Clinician Peer Support Program. J Patient Saf. 2018;14(3):e56–60.

41. Foreman S. Developing a process to support perinatal nurses after a critical event. 2014;18(1):61–5.

42. El Hechi MW, Bohnen JD, Westfal M, Han K, Cauley C, Wright C, et al. Design and Impact of a Novel Surgery-Specific Second Victim Peer Support Program. J Am Coll Surg. 2020;230(6):926–33.

43. Rivera-Chiauzzi E, Smith H, Moore-Murray T, Lee C, Goffman D, Bernstein P, et al. Healing Our Own: A Randomized Trial to Assess Benefits of Peer Support. J Patient Saf. 2022;18(1):e308–14.

44. Peterson U, Bergström G, Samuelsson M, Åsberg M, Nygren Å. Reflecting peer-support groups in the prevention of stress and burnout: randomized controlled trial. J Adv Nurs. 2008;63(5):506–16.

45. Finney RE, Jacob A, Johnson J, Messner H, Pulos B, Sviggum H. Implementation of a Second Victim Peer Support Program in a Large Anesthesia Department. AANA. 2021;89(3):235–44.

46. Bryant R. Raising Awareness of Second Victim Phenomenon and SupportingYOU: A Quality and Safety Initiative. Graduate Theses, Dissertations, and Problem Reports. West Virginia: West Verginia University; 2022. 10169.

47. Mellins CA, Mayer LES, Glasofer DR, Devlin MJ, Albano AM, Nash SS, et al. Supporting the well-being of health care providers during the COVID-19 pandemic: The CopeColumbia response. Gen Hosp Psychiatry. 2020;67:62–9.

For peer review only

48. Merandi J, Winning AM, Liao N, Rogers E, Lewe D, Gerhardt CA. Implementation of a second victim program in the neonatal intensive care unit: An interim analysis of employee satisfaction. J Patient Saf Risk Manag. 2018;23(6):231–8.

49. Allen D, Spencer G, McEwan K, Catarino F, Evans R, Crooks S, Gilbert P. The Schwartz Centre Rounds: Supporting mental health workers with the emotional impact of their work. Int J Ment Health Nurs. 2020 Oct;29(5):942-952.

50. Schuster MA. Creating the Hematology/Oncology/Stem Cell Transplant Advancing Resiliency Team: A Nurse-Led Support Program for Hematology/Oncology/Stem Cell Transplant Staff. J Pediatr Oncol Nurs. 2021;38(5):331–41.

51. Calder-Sprackman S, Kumar T, Gerin-Lajoie C, Kilvert M, Sampsel K. Ice cream rounds: The adaptation, implementation, and evaluation of a peer-support wellness rounds in an emergency medicine resident training program. CJEM. 2018;20(5):777–80.

52. Graham P, Zerbi G, Norcross W, Montross-Thomas L, Lobbestael L, Davidson J. Testing of A Caregiver Support Team. EXPLORE. 2019;15(1):19–26.

53. Winning AM, Merandi JM, Lewe D, Stepney LMC, Liao NN, Fortney CA, et al. The emotional impact of errors or adverse events on healthcare providers in the NICU: The protective role of coworker support. J Adv Nurs. 2018;74(1):172–80.

54. Carbone R, Ferrari S, Callegarin S, Casotti F, Turina L, Artioli G, et al. Peer support between healthcare workers in hospital and out-of-hospital settings: a scoping review. Acta Biomed Atenei Parm. 2022;93(5):e2022308.

55. Pratt SD, Jachna BR. Care of the clinician after an adverse event. Int J Obstet Anesth. 2015;24(1):54–63.

56. Dukhanin V, Edrees HH, Connors CA, Kang E, Norvell M, Wu AW. Case: A Second Victim Support Program in Pediatrics: Successes and Challenges to Implementation. J Pediatr Nurs. 2018;41:54–9.

57. Liukka M, Steven A, Vizcaya Moreno MF, Sara-aho AM, Khakurel J, Pearson P, et al. Action after Adverse Events in Healthcare: An Integrative Literature Review. Int J Environ Res Public Health. 2020;17(13):4717.

58. Moudatsou M, Stavropoulou A, Philalithis A, Koukouli S. The Role of Empathy in Health and Social Care Professionals. Healthcare. 2020;8(1):26.

59. Arnetz JE, Zhdanova LS, Elsouhag D, Lichtenberg P, Luborsky MR, Arnetz BB. Organizational Climate Determinants of Resident Safety Culture in Nursing Homes. The Gerontologist. 2011;51(6):739–49.

60. Busch IM, Moretti F, Campagna I, Benoni R, Tardivo S, Wu AW, et al. Promoting the Psychological Well-Being of Healthcare Providers Facing the Burden of Adverse Events: A Systematic Review of Second Victim Support Resources.

61. Malik RF, Buljac-Samardžić M, Amajjar I, Hilders CGJM, Scheele F. Open organisational culture: what does it entail? Healthcare stakeholders reaching consensus by means of a Delphi technique. BMJ Open. 2021;11(9):e045515.

62. Aghaei H, Sadat Asadi Z, Mirzaei Aliabadi M, Ahmadinia H. The Relationships Among Occupational Safety Climate, Patient Safety Climate, and Safety Performance Based on Structural Equation Modeling. J Prev Med Pub Health. 2020;53(6):447–54.

For peer review only

63. Mira JJ, Lorenzo S, Carrillo I, Ferrús L, Silvestre C, Astier P, et al. Lessons learned for reducing the negative impact of adverse events on patients, health professionals and healthcare organizations. Int J Qual Health Care. 2017;29(4):450–60.

64. Rosak-Szyrocka J. Employee motivation in health care. Prod Eng Arch. 2015;6/1:21–5.

65. Kitsios F, Kamariotou M. Job satisfaction behind motivation: An empirical study in public health workers. Heliyon. 2021;7(4):e06857.

66. Dubois CA, Singh D. From staff-mix to skill-mix and beyond: towards a systemic approach to health workforce management. Hum Resour Health. 2009;7(1):87.

67. Boguslavsky V, Gutierrez R, Holschneider S. Effective Leadership for Quality Improvement in Health Care A Practical Guide. Published by USAID ASSIST Project.Chevy Chase. MD: University Research CO (LLC) URS; 2019.

68. Joint Commission. The essential role of leadership in developing a safety culture. Department of Corporate Communications; 2007.

69. Usman M, Ghani U, Cheng J, Farid T, Iqbal S. Does Participative Leadership Matters in Employees' Outcomes During COVID-19? Role of Leader Behavioral Integrity. Front Psychol. 2021;12:646442.

70. Fagerdal B, Lyng HB, Guise V, Anderson JE, Thornam PL, Wiig S. Exploring the role of leaders in enabling adaptive capacity in hospital teams – a multiple case study. BMC Health Serv Res. 2022;22(1):908.

71. Søvold LE, Naslund JA, Kousoulis AA, Saxena S, Qoronfleh MW, Grobler C, et al. Prioritizing the Mental Health and Well-Being of Healthcare Workers: An Urgent Global Public Health Priority. Front Public Health. 2021;9:679397.

72. MEASURE Evaluation. Fundamentals of Implementation Research. U.S. Agency for International Development; 2012.

73. Straus SE, Tetroe J, Graham ID, Zwarenstein M, Bhattacharyya O, Shepperd S. Monitoring use of knowledge and evaluating outcomes. Can Med Assoc J. 2010;182(2):E94–8.

74. Wade L, Williams N, Fitzpatrick E, Parker R, Hurley K. Organizational interventions and policies to support second victims in acute care settings: A scoping study. Can J Emerg Med. 2019;21:S112–3.

For peer review only

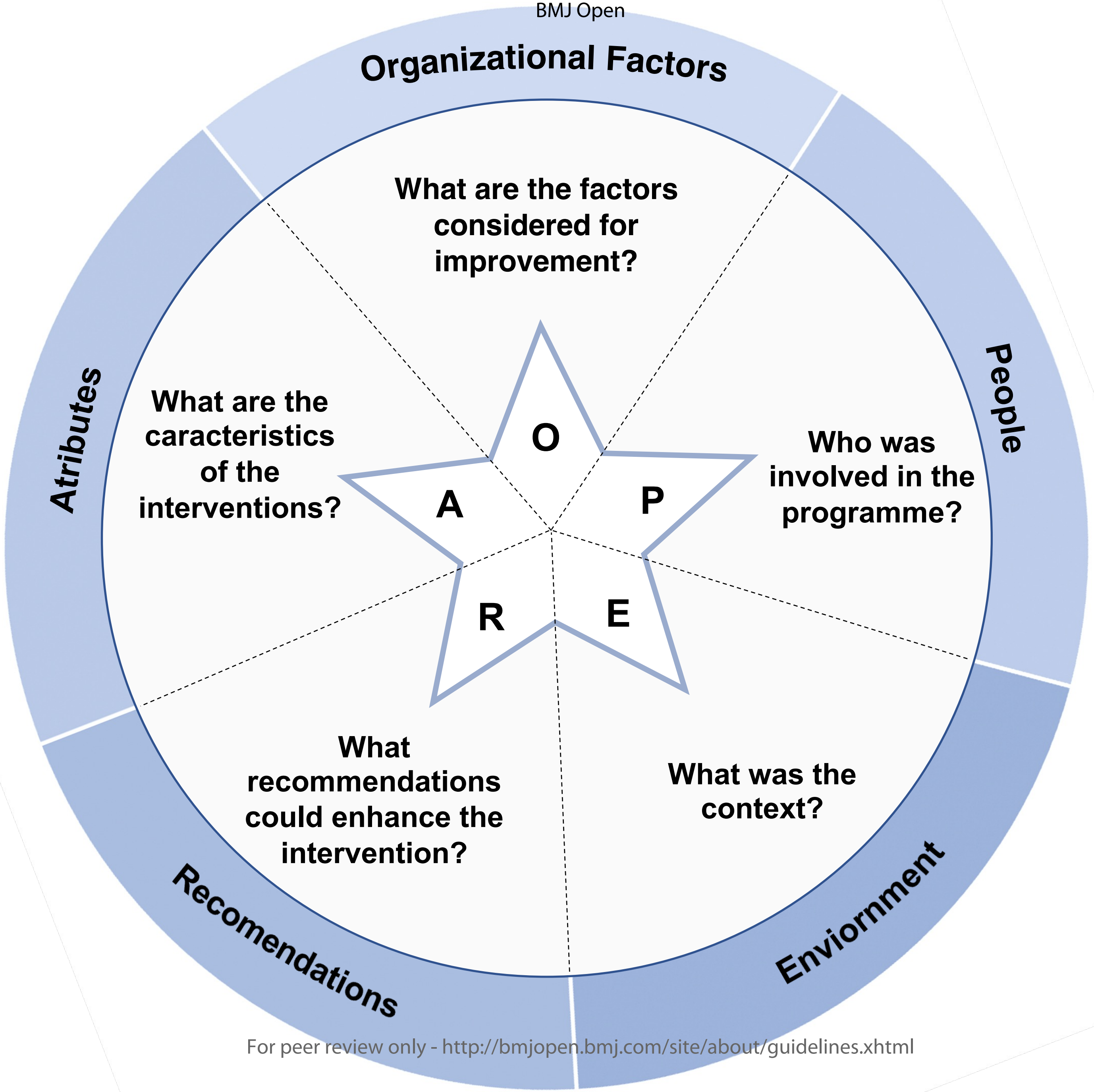
Organizational Factors
What are the factors considered for improvement?
People
Who was involved in the programme?
Enviornment
What was the context?
Recomendations
What recommendations could enhance the intervention?
Atributes
What are the caracteristics of the interventions?
O
P
E
R
A

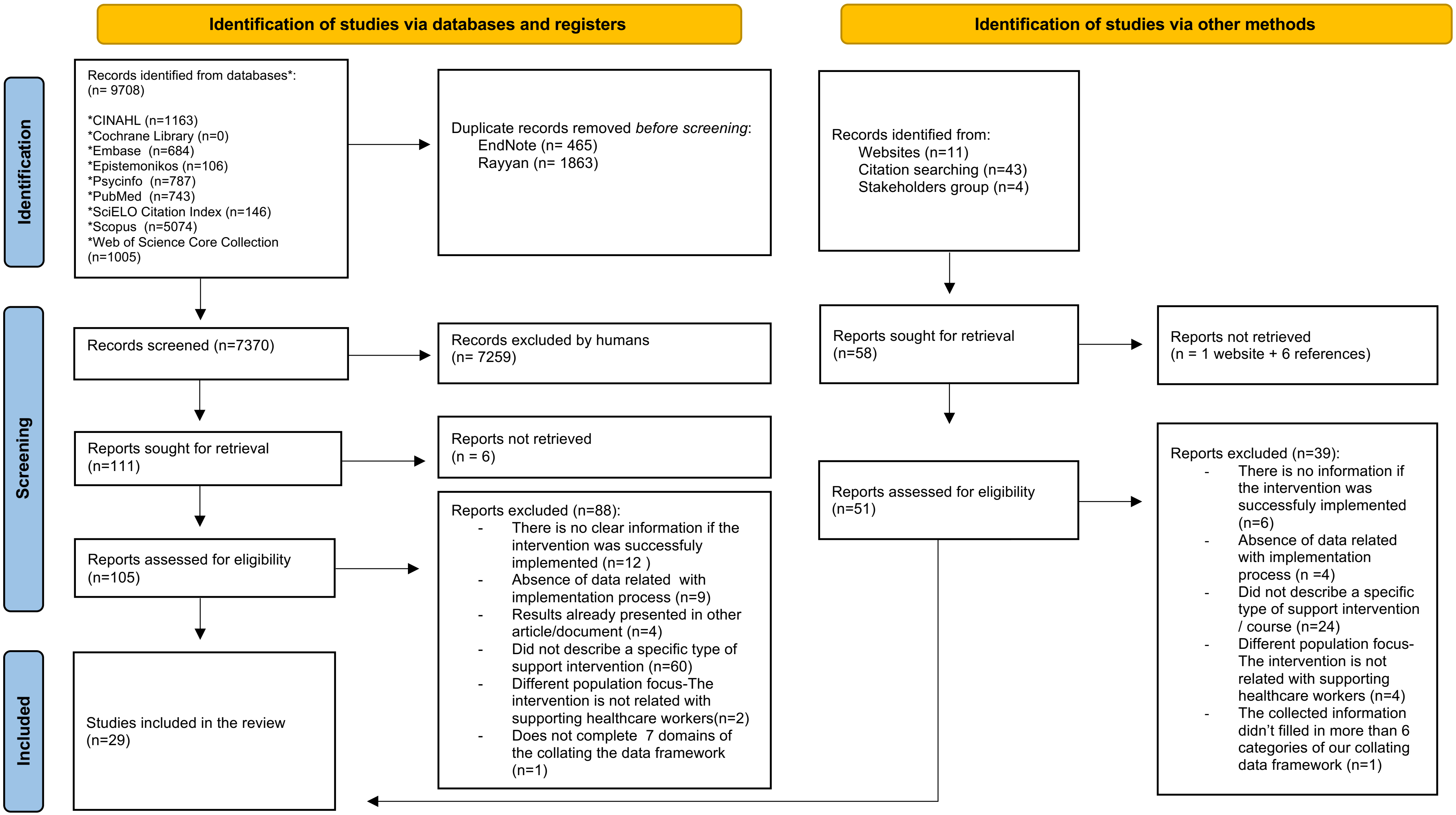
Identification of studies via databases and registers
Identification of studies via other methods
Identification
Screening
Included
Records identified from databases*:
(n= 9708)
*CINAHL (n=1163)
*Cochrane Library (n=0)
*Embase (n=684)
*Epistemonikos (n=106)
*Psycinfo (n=787)
*PubMed (n=743)
*SciELO Citation Index (n=146)
*Scopus (n=5074)
*Web of Science Core Collection
(n=1005)
Duplicate records removed *before screening*:
EndNote (n= 465)
Rayyan (n= 1863)
Records identified from:
Websites (n=11)
Citation searching (n=43)
Stakeholders group (n=4)
Records screened (n=7370)
Records excluded by humans
(n= 7259)
Reports sought for retrieval
(n=58)
Reports not retrieved
(n = 1 website + 6 references)
Reports sought for retrieval
(n=111)
Reports not retrieved
(n = 6)
Reports assessed for eligibility
(n=51)
Reports excluded (n=39):
- There is no information if the intervention was successfuly implemented (n=6)
- Absence of data related with implementation process (n =4)
- Did not describe a specific type of support intervention / course (n=24)
- Different population focus- The intervention is not related with supporting healthcare workers (n=4)
- The collected information didn't filled in more than 6 categories of our collating data framework (n=1)
Reports assessed for eligibility
(n=105)
Reports excluded (n=88):
- There is no clear information if the intervention was successfuly implemented (n=12)
- Absence of data related with implementation process (n=9)
- Results already presented in other article/document (n=4)
- Did not describe a specific type of support intervention (n=60)
- Different population focus-The intervention is not related with supporting healthcare workers(n=2)
- Does not complete 7 domains of the collating the data framework (n=1)
Studies included in the review
(n=29)

Complementary table 1 - Search strategies applied to the different electronic databases

**Applied search strategies :**			**Results**
Context	#1	“Health Services” OR “Health Facilities” OR “Healthcare” OR “Primary health care” OR “General Practice” OR “Family practice” OR “Ambulatory Care” OR “Nursing Care” OR “Family unit” OR "Hospitals"	
Content	#2	“Program evaluation” OR "Support program” OR “Peer support” OR “Support strategies” OR “Organizational factors” OR “Organizational culture”	
Population	#3	(“Health personnel” OR “Physicians” OR “Nurses” OR “Doctor” OR “Practitioner” OR “Medical students” OR “Medical residents” OR “Healthcare providers” OR “Healthcare worker” OR “Healthcare staff") AND (“Error” OR “Near miss” OR “Adverse Event” OR “Clinical Error” OR “Medical error” OR “Second victim” OR “Wounded caregiver” OR “Wounded healer” OR “Secondary trauma”)	
Web of Science Core collection<br>*All databases*<br><br>Last updated 2022/10/31	#1 AND #2 AND #3	Error OR Adverse Event OR Clinical Error OR Medical error OR Second victim OR Wounded caregiver OR Wounded healer OR Secondary trauma (Topic) and Health Services OR Health Facilities OR Healthcare OR Primary health care OR General Practice OR Family practice OR Ambulatory Care OR Nursing Care OR Family unit OR Hospitals (Topic) and Program evaluation OR Support program OR Peer support OR Support strategies OR Organizational factors OR Organizational culture (Topic) and Health personnel OR Physicians OR Nurses OR Doctor OR Practitioner OR Medical students OR Medical residents OR Healthcare providers OR Healthcare worker OR Healthcare staff (Topic) and Article or Review Article or Early Access or Editorial Material (Document Types)	1005 results
Complete search on Pubmed (Medline**)**<br><br>Last updated 2022/10/31	#1 AND #2 AND #3	((((("Health Services"[MeSH Terms]) OR ("Health Services"[Title/Abstract])) OR (("Health Facilities"[MeSH Terms]))) OR ("Health Facilities"[Title/Abstract])) OR ("healthcare"[Title/Abstract])) OR ("Primary health care"[MeSH Terms])) OR ("Primary health care"[Title/Abstract])) OR ("General Practice"[MeSH Terms])) OR ("General Practice"[Title/Abstract])) OR ("Family practice"[MeSH Terms])) OR ("Family practice"[Title/Abstract])) OR ("Ambulatory Care"[MeSH Terms])) OR ("Ambulatory Care"[Title/Abstract])) OR ("Nursing Care"[Title/Abstract])) OR ("Family unit"[Title/Abstract])) OR ("Hospitals"[Title/Abstract]))) AND (("Program evaluation"[Title/Abstract]) OR ("Support program"[Title/Abstract]) OR ("Peer support"[Title/Abstract]) OR ("Support strategies"[Title/Abstract]) OR ("Organizational factors"[Title/Abstract]) OR ("Organizational culture"[MeSH Terms]) OR ("Organizational culture"[Title/Abstract])))) AND (("Health personnel"[MeSH Terms]) OR ("Health personnel"[Title/Abstract]) OR ("Physicians"[Title/Abstract]) OR ("Nurses"[Title/Abstract]) OR ("Doctor"[Title/Abstract]) OR ("Practitioner"[Title/Abstract]) OR ("Medical students"[Title/Abstract]) OR ("Medical residents"[Title/Abstract]) OR ("Healthcare providers"[Title/Abstract]) OR ("Healthcare worker"[Title/Abstract]) OR ("Healthcare staff"[Title/Abstract])))) AND ((Error[Title/Abstract]) OR ((“Near miss”[Title/Abstract]) OR (“Adverse Event”[Title/Abstract]) OR (“Clinical Error”[Title/Abstract]) OR (“Medical error”[MeSH Terms]) OR (“Medical error”[Title/Abstract]) OR (“Second victim”[Title/Abstract]) OR (“Wounded caregiver”[Title/Abstract]) OR (“Wounded healer”[Title/Abstract]) OR (“Secondary trauma”[Title/Abstract])))	743 results

For peer review only

Complete search on PsycInfo<br><br>Last updated 2022/11/3	#1 AND #2 AND #3	(((health services) OR (health facilities) OR (healthcare) OR (primary health care) OR (general practice) OR (family practice) OR (ambulatory care) OR (nursing care) OR (family unit) OR (hospital)) AND ((program evaluation) OR (support programs) OR (peer support) OR (support strategies) OR (organizational factors) OR (organizational culture)) AND ((health personnel) OR (physician) OR (nurse) OR (doctors) OR (practitioner) OR (medical students) OR (medical residents) OR (healthcare providers) OR (healthcare workers) OR (healthcare staff)) AND ((errors) OR (near miss) OR (adverse events) OR (clinical errors) OR (medical errors) OR (second victim) OR (wounded healer) OR (secondary trauma))	787 results
SCOPUS<br><br>Last updated 2022/11/2	#1 AND #2 AND #3	(TITLE-ABS-KEY (health AND services) OR TITLE-ABS-KEY (health AND facilities) OR TITLE-ABS-KEY (healthcare) OR TITLE-ABS-KEY (primary AND health AND care) OR TITLE-ABS-KEY (general AND practice) OR TITLE-ABS-KEY (family AND practice) OR TITLE-ABS-KEY (ambulatory AND care) OR TITLE-ABS-KEY (nursing AND care) OR TITLE-ABS-KEY (family AND unit) OR TITLE-ABS-KEY (hospitals)) AND (TITLE-ABS-KEY (program AND evaluation) OR TITLE-ABS-KEY (support AND program) OR TITLE-ABS-KEY (peer AND support) OR TITLE-ABS-KEY (support AND strategies) OR TITLE-ABS-KEY (organizational AND factors) OR TITLE-ABS-KEY (organizational AND culture)) AND (TITLE-ABS-KEY (health AND personnel) OR TITLE-ABS-KEY (physicians) OR TITLE-ABS-KEY (nurses) OR TITLE-ABS-KEY (doctor) OR TITLE-ABS-KEY (practitioner) OR TITLE-ABS-KEY (medical AND students) OR TITLE-ABS-KEY (medical AND residents) OR TITLE-ABS-KEY (healthcare AND providers) OR TITLE-ABS-KEY (healthcare AND worker) OR TITLE-ABS-KEY (healthcare AND staff)) AND (TITLE-ABS-KEY (error) OR TITLE-ABS-KEY (near AND miss) OR TITLE-ABS-KEY (adverse AND event) OR TITLE-ABS-KEY (clinical AND error) OR TITLE-ABS-KEY (medical AND error) OR TITLE-ABS-KEY (second AND victim) OR TITLE-ABS-KEY (wounded AND caregiver) OR TITLE-ABS-KEY (wounded AND healer) OR TITLE-ABS-KEY (secondary AND trauma)) AND (LIMIT-TO (DOCTYPE , "ar") OR LIMIT-TO (DOCTYPE , "re") OR LIMIT-TO (DOCTYPE , "cp") OR LIMIT-TO (DOCTYPE , "cr"))	5074 results
Complete search on CINAHL<br><br>Last updated 2022/10/31	#1 AND #2 AND #3	TX ("health services" OR "health facilities" OR healthcare OR "primary health care" OR "general practice" OR "family practice" OR "ambulatory care" OR "nursing care" OR "family unit" OR hospital) AND TX ("program evaluation" OR "support programs" OR "peer support" OR "support strategies" OR "organizational factors" OR "organizational culture") AND TX ("health personnel" OR physician OR nurse OR doctors OR practitioner OR "medical students" OR "medical residents" OR "healthcare providers" OR "healthcare workers" OR "healthcare staff") AND TX (errors OR "near miss" OR "adverse events" OR "clinical errors" OR "medical errors" OR "second victim" OR "wounded healer" OR "secondary trauma")	1163 results
Complete search on Embase<br><br>Last updated 2022/10/31	#1 AND #2 AND #3	('health services':ab,ti OR 'health facilities':ab,ti OR 'healthcare':ab,ti OR 'primary health care':ab,ti OR 'general practice':ab,ti OR 'family practice':ab,ti OR 'ambulatory care':ab,ti OR 'nursing care':ab,ti OR 'family unit':ab,ti OR 'hospitals':ab,ti) AND ('program evaluation':ab,ti OR 'support program':ab,ti OR 'peer support':ab,ti OR 'support strategies':ab,ti OR 'organizational factors':ab,ti OR 'organizational culture':ab,ti) AND ('health personnel':ab,ti OR 'physicians':ab,ti OR 'nurses':ab,ti OR 'doctor':ab,ti OR 'practitioner':ab,ti OR 'medical students':ab,ti OR 'medical residents':ab,ti OR 'healthcare providers':ab,ti OR 'healthcare worker':ab,ti OR 'healthcare staff':ab,ti) AND (error:ab,ti OR 'adverse event':ab,ti OR 'clinical error':ab,ti OR 'medical error':ab,ti OR 'second victim':ab,ti OR 'wounded caregiver':ab,ti OR 'wounded healer':ab,ti OR 'secondary traumatic stress':ab,ti)	684 results
Scielo citation index through. Web of Science<br><br>Last updated: 2022/11/4	#1 AND #2 AND #3	("Health Services" OR "Health Facilities" OR "Healthcare" OR "Primary health care" OR "General Practice" OR "Family practice" OR "Ambulatory Care" OR "Nursing Care" OR "Family unit" OR "Hospitals") AND ("Program evaluation" OR "Support program" OR "Peer support" OR "Support strategies" OR "Organizational factors" OR "Organizational culture") AND ("Health personnel" OR "Physicians" OR "Nurses" OR "Doctor" OR "Practitioner" OR "Medical students" OR "Medical residents" OR "Healthcare providers" OR "Healthcare worker" OR "Healthcare staff" and "Error" OR "Near miss" OR "Adverse Event" OR "Clinical Error" OR "Medical error" OR "Second victim" OR "Wounded caregiver" OR "Wounded healer" OR "Secondary trauma")	146 results

For peer review only - http://bmjopen.bmj.com/site/about/guidelines.xhtml

Complete search on Epistemonikos<br><br>Last updated 2022/11/2	#1 AND #2 AND #3	(title ((((health services) OR (health facilities) OR (healthcare) OR (primary health care) OR (general practice) OR (family practice) OR (ambulatory care) OR (nursing care) OR (family unit) OR (hospital)) AND ((program evaluation) OR (support programs) OR (peer support) OR (support strategies) OR (organizational factors) OR (organizational culture)) AND ((health personnel) OR (physician) OR (nurse) OR (doctors) OR (practitioner) OR (medical students) OR (medical residents) OR (healthcare providers) OR (healthcare workers) OR (healthcare staff)) AND ((errors) OR (near miss) OR (adverse events) OR (clinical errors) OR (medical errors) OR (second victim) OR (wounded healer) OR (secondary trauma))) OR abstract:(((health services) OR (health facilities) OR (healthcare) OR (primary health care) OR (general practice) OR (family practice) OR (ambulatory care) OR (nursing care) OR (family unit) OR (hospital)) AND ((program evaluation) OR (support programs) OR (peer support) OR (support strategies) OR (organizational factors) OR (organizational culture)) AND ((health personnel) OR (physician) OR (nurse) OR (doctors) OR (practitioner) OR (medical students) OR (medical residents) OR (healthcare providers) OR (healthcare workers) OR (healthcare staff)) AND ((errors) OR (near miss) OR (adverse events) OR (clinical errors) OR (medical errors) OR (second victim) OR (wounded healer) OR (secondary trauma))))	106 results
Language		No language filter/restraint will be applied	
Period		No period filter/restraint will be applied	
Exclusion criteria		Article types not included: editorial , letter to the editor, cases series, case reports, narrative review, commentary	

For peer review only

Complementary table 2- Characteristics of the included studies

Authors (year of publication)	Type of study and level of evidence	Main aim of the study	Study design	Methods	Key findings	Quality assessment
Allen,Spencer, McEwan,Catarino , Evans, Crooks et al (2020)	Scientific article – Level VI[1]	To evaluate the experience of HCWs working in a mental health service after attending the Schwartz Rounds.	Mixed Method	Application of a quantitative evaluation form after the rounds and focus group and 6 years follow up.	Rounds were helpful, insightful and relevant to support HCWs in a non-blaming environment. The six-year follow-up revealed that the Rounds were still rated positively.	4*/5 of the MMAT criteria *there are no inconsistencies between results
Bryant (2022)	Graduate Theses, Dissertation s - Level VI[1]	To raise staff awareness on resilience, SVP and SupportingYOU intervention in a large academic children's hospital.	Quantitative descriptive	Application of preintervention and postintervention quantitative surveys,	Intervention increased the staff awareness on SVP, their perception on resilience and contribute for a proactive culture to manage critical incidents, by increasing the sense of feeling cared by the institution.	5*/5 of the MMAT criteria
Civil, Hoskins (2022)	Scientific article- Level VI[1]	To describe the design and implementation of a critical incident peer response team program at Waikato Hospital.	Quantitative descriptive	Application of postintervention quantitative survey.	Positive feedback from the attendants was given after the intervention. The program contributed to a supportive culture and enhanced teamwork.	2*/5 of the MMAT * 3 criteria were evaluated as "can't tell" : the sample is not representative of the target population, there is a risk of nonresponse bias; absence of statistical analysis
Edrees, Connors, Paine, Norvell, Taylor, Wu (2016)	Scientific article- Level VI[1]	To describe the development of RISE, initial evaluation of pilot programme and hospital-wide implementation at Johns Hopkins Hospital.	Mixed Method	Application of pre and post implementation quantitative surveys and focus group.	Although there were few calls in the first year of implementation, the rate of calls increased during the next years. Evaluation indicates the success of most encounters with callers and effectiveness of training to prepared the peer supporters to support second victim.	4*/5 of the MMAT criteria *there are no inconsistencies between results
Finney, Jacob, Johnson, Messner, Pulos, Sviggum (2021)	Scientific article Level VI[1]	To describe the implementation and evaluation of a SV program – Healing Emotional lives of Peers (HELP) in a Department of Anaesthesiology.	Mixed method	Application of post implementation quantitative surveys with open questions.	The program developed during 3 years was successfully implemented in the first 2 years of the program inception and is now a resource for other institutions in the region.	4*/5 of the MMAT criteria *no confounders accounted for in the design and analysis
Foreman (2014)	Paper Level VI[1]	To describe the development and implementation of a plan to help perinatal nurses to cope with stress after critical incidents and deaths in a family birth centre.	Qualitative	Qualitative description of the programme testing and the learning from its implementation.	Feedback on the use of the critical event plan has been positive. Nurses found the plan very useful after a tragedy on the unit related with the death of a new-born.	3*/ 5 of the MMAT criteria * 2 criteria were evaluated as "can't tell": qualitative data collection methods are not described; coherence between qualitative data sources, collection, analysis and interpretation is not clear;
Graham, Zerbi, Norcross, Montross-	Scientific article	To describe the Caregiver Support Team programme implementation in	Mixed-method	Application of baseline and post implementation (3 months follow up)	As an addition to the previous implemented Code Lavender intervention, the program was accepted and positively evaluated. Efforts will	4*/5 of the MMAT criteria *no confounders accounted for in the design and analysis

Thomas, Lobbestael , Davidson (2019)	Level VI[1]	an academic medical centre and evaluate its feasibility.		quantitative surveys . Self reported experiences of attendants.	be made to disseminate the program system-wide.	
Johnson, Simms-Ellis, Janes, Mills, Budworth, Atkinson ,Harrison (2020)	Scientific article<br><br>Level VI[1]	To evaluate a psychological resilience coaching intervention for National Health Services (NHS) trust sites and university premises.	Mixed-method	Application of pre and post implementation, follow up after the coaching phone call (10–20 days after the workshop) and 4–6 weeks after the workshop. Interviews application.	The program seems to be feasible and effective on improving general resilience of clinicians, by improving their knowledge and confidence in coping after adverse events.	5/5 of the MMAT criteria
Krzan, Merandi, Morvay, Mirtallo (2015)	Scientific article<br><br>Level VI[1]	To describe YOU Matter Support program implementation at Nationwide Children's Hospital.	Mixed-method	Application of pre and post (after 5 months) online surveys. Collection of peer encounters documentation of the Second Victim SharePoint site.	Most of the surveyed staff reported that the department benefited from implementation of the SV program. After the success of the pilot program, the hospital has decided to expand the YOU Matter program hospital- wide.	4*/5 of the MMAT criteria *no confounders accounted for in the design and analysis
Merandi, Liao , Lewe, Morvay, Stewart, Catt, Scott (2017)	Scientific article<br><br>Level VI[1]	To describe the replication of the ForYOU Matter program and expansion of the program to a large pediatric institution.	Quantitative descriptive	Description of the program implementation and of the collected data from electronic tracking system via SharePoint.	Application of the MUHC support model in the Nationwide Children's Hospital was validated and demonstrates that it's suitable and transferrable to other healthcare facilities and contexts.	3*/5 of the MMAT criteria<br>2 criteria was evaluated as "can't tell": no clear information if the risk of nonresponse bias was low: absence of statistical analysis
Lane, Newman, Taylor, O'Neill, Ghetti, Woltman, Waterman (2018)	Scientific article<br><br>Level VI[1]	To describe the Washington University School of Medicine Clinician Peer Support Program.	Quantitative descriptive	Description of the program development and its implementation.	Program was successfully implemented, however difficulties were found in sustaining the program over time, since peer supporters don't have protected time to be part of the program and therefore the programme activities have conflict with their operative schedules.	3*/5 of the MMAT criteria –<br>*2 criteria was evaluated as "can't tell": nonresponse bias was not applicable to this study; absence of statistical analysis
Mellins, Mayer, Glasofer, Devlin, Albano, Nash et al (2020)	Scientific article<br><br>Level VI[1]	To describe the application of CopeColombia programme in a Department of Psychiatry of a large urban medical centre's.	Mixed method	Description of the key issues/themes and facilitator responses emerged in the sessions (facilitator interventions);<br>post peer supporter group online survey (perceived impact of the program).	HCW emotional distress decreased after the program implementation. Peer Support Groups were the most used, valued and recommended. Sustainability of the program is critical due to financial constrains.	3*/5 of the MMAT criteria<br>*there are no inconsistencies between results;<br>1 criteria was evaluated as "can't tell": it's not clear if the study follows the traditional quality criteria of the qualitative methods involved
Merandi, Winning , Liao, Rogers, Lewe, Gerhardt (2018)	Scientific article<br><br>Level VI[1]	To assess healthcare providers satisfaction in the early implementation of a SV program in a group of neonatal intensive care units.	Mixed method	Application of pre and post implementation surveys with closed and open – ended questions. Thematic content analysis of the qualitative data from the survey.	This study suggest that peer support programs are likely to be viewed as positive and helpful for frontline HCW and managers. However, additional work should be done to assure the accessibility and effectiveness of the program.	4*/5 of the MMAT criteria *no confounders accounted for in the design and analysis

Rivera-Chiauzzi,Smith,Moore-Murray, Lee, Goffman, Bernstein, Chazotte (2022)	Scientific article<br><br>Level II[1]	To describe the development and evaluation a structured peer support program to address the needs of providers involved in obstetric adverse outcomes and to compare it with a routine support for HCW after the same type of events.	Pilot Randomized controlled trial	Application of needs assessment survey before program implementation. Surveys were applied at baseline, 3 months, and 6 months follow up.	Structured peer support program was successfully implemented with limited resources. The enhanced support group was significantly more likely to consider departmental leadership as one of the most helpful resources for support after adverse event. All participants refer were thriving at 6-month follow-up.	4*/5 of the MMAT criteria<br><br>*outcome assessors are not blinded to the intervention provided
Roesler, Ward, Short (2009)	Scientific article<br><br>Level VI[1]	To describe the recovery and reintegration of Neonatal Intensive Care Unit staff after a critical incident based on the Just Culture philosophy.	Qualitative: Single Case	Description of the protocol activation and application after the involvement of a severe adverse event.	The protocol was successfully applied and helped the affected HCW to thrive and return back to work after their involvement in the severe adverse event.	3*/5 of the MMAT criteria<br>*2 criteria were evaluated as "can't tell": qualitative data collection methods are not described ; it's not clear if there is coherence between qualitative data sources, collection, analysis and interpretation
Schrøder, Bovil,, Jørgensen, Abrahamsen (2022)	Scientific article<br><br>Level VI[1]	To evaluate the Buddy Study program by assessing HCW experiences with having the program in the department, attending the compulsory seminar, and using a buddy or being activated as a buddy.	Mixed method	Application of quantitative questionnaires to assess HCW experiences of attending the Buddy Study seminar and participating in the Buddy Study program using a buddy or being activated as a buddy, along with two open-ended questions and three individual interviews for more elaborate answers.	The buddy study program was evaluated positively. It allowed to acknowledge the SVP and strengthen a supportive organizational culture by creating buddy relationships to support HCW after stressful events during care.	4*/5 of the MMAT criteria *no confounders accounted for in the design and analysis
Shapiro, Galowitz (2016)	Scientific article<br><br>Level VI[1]	To describe the development and implementation of Brigham and Women's Hospital peer support program to support HCW after the impact of an emotional stressful event.	Quantitative descriptive	Description of the program and quantitative data collection of outreached calls and peer support sessions.	The peer support program has been implemented for 4 years and it's expected to be expanded hospital-wide. The program does not yet reach many clinicians that might be in need of support after stressful events.	3*/5 of the MMAT criteria<br>*3 criteria were evaluated as "can't tell": it's not clear if the sample is representative of the target population; risk of nonresponse bias low and statistical analysis are not applicable
Thompson, Hunnicutt, Broadhead, Vining, Aroke (2022)	Scientific article<br><br>Level VI[1]	To describe the implementation of SV support program in a large academic medical centre based on a quality improvement project.	Quantitative descriptive	Application the SVEST survey pre and post peer support program implementation.	Although the study didn't find statistically significant differences in pre and post implementation the program has received a positive feedback among leadership members and peer supporters.	4*/5 of the MMAT criteria<br>*2 criteria were evaluated as "can't tell": low response rate in post-implementation survey may have decreased generalizability of results which can affect the sample representative ; potential for response bias was identified in the study

El Hechi , Bohnen, Westfal, Han, Cauley, Wright, Schulz et al (2020)	Scientific article<br><br>Level VI[1]	To describe the design and implementation of a SV surgical peer support program and its 1 year impact.	Mixed method	Programme design: literature review and multidisciplinary expert group discussion; Evaluation of the impact of the programme: quantitative and qualitative surveys application – evaluation of the peer supporters and of the program.	The first surgery-specific peer support program in US was successfully implemented. After 1 year experience, the program is highly used and well received.	4/5 of the MMAT criteria<br>*inconsistencies between quantitative and qualitative are not addressed in the study
Mira, Carrillo, Guilabert, Lorenzo, Pérez-Pérez, Silvestre et al (2017)	Scientific article<br><br>Level VI[1]	To describe the development and assessment of Mitigating Impact in Second Victims (MISE) programme on the awareness and information focused on the SVP.	Quantitative descriptive	Description of accreditation process; quantitative survey applied to 26 experts to assess structure and content; Quantitative survey applied to frontline HCW; evaluation of knowledge gained from the online program after each online module.	The online program was positively assessed by the accreditation agency, by the experts and the HCWs. The programme increases knowledge about patient safety, SVP and how to act after a severe adverse event. The time demand is reasonable for complete the course.	5/5 of the MMAT criteria
Scott, Hirschinger, Cox, McCoig, Hahn-Cover, Epperly et al. (2010)	Scientific article<br><br>Level VI[1]	To describe the deployment of ForYOU Team programme , an institutional rapid response system (RRS) for second victims at University of Missouri Health Care (MUHC).	Mixed method	Interviews and quantitative surveys application by the MUHC faculty and staff to support the development of the programme.	After identifying the need for support the SV in MUHC , the programme was designed, developed and successfully implemented. The programme has been integrated in the healthcare in the scheduled activities of the team leaders and clinical teams.	4*/5 of the MMAT criteria<br>*inconsistencies between quantitative and qualitative are not addressed in the study
Schuster (2021)	Scientific article<br><br>Level VI[1]	To describe the implementation of the HART programme and assess its impact on the Hematology/Oncology/Stem Cell Transplant Unit.	Mixed method	Preintervention and midpoint survey (after 3 months of pilot program), and qualitative subjective information collection from daily coach documentation entries.	HART programme was successfully implemented in the department and with high level of utilization from the staff. After implementation of HART, mental, emotional and physical wellbeing of all staff members improved.	4*/5 of the MMAT criteria<br>*inconsistencies between quantitative and qualitative are not addressed in the study
Calder-Sprackman; Kumar; Gerin-Lajoie; Kilvert; Sampsel (2018)	Scientific article<br><br>Level VI[1]	To describe the implementation, adaptation and evaluation of the ice cream rounds intervention in an emergency medicine training programme.	Quantitative descriptive	Application of a pre-implementation survey for needs assessment and a post implementation for feedback after intervention.	This Canadian initiative increased the overall perception of support and companionship, decreased feelings of stress, anxiety and burnout and can have a positive impact on the clinical practice in emergency medicine residents.	3*/5 of the MMAT criteria<br>*2 criteria were evaluated as "can't tell": it's not clear if the sample is representative of the target population; absence of statistical analysis

Peterson, Bergström, Samuelsson, Åsberg, Nygren (2008)	Scientific article<br><br>Level II[1]	To evaluate the effect a reflecting peer-support group on self-reported health, burnout and on perceived changes in work conditions of multidisciplinary group of HCW in 3 hospitals of a county council in Sweden.	Randomized controlled trial.	Application of a baseline survey (7 months before the interview started), pre implementation questionnaire , immediately after the intervention ended (post intervention), 7 and 12 months after the intervention follow up.	The study showed positive intervention effects (after 12 months of the programme implementation) on overall health and decreased perceived work demands. A decrease in exhaustion, depression and anxiety symptoms was observed , as well as an increase in vitality.	5/5 of the MMAT criteria
Rubin, Rassman (2021)	Scientific article<br><br>Level VI[1]	To describe the social work led peer support model, COVID-19 Am I Resilient (cAIR), developed and implemented during the first wave of the COVID-19 pandemic in a large urban healthcare system.	Mixed method	Application of pre and post intervention surveys with quantitative and open ended questions.	The pilot programme cAIR was successfully implemented and provided support to normalize frontline HCW experience during Covid 19 pandemic, encouraged coworkers connections and developed coping skills . Participant engagement in cAIR were strong, however overall utilization of programme activities was low.	4*/5 of the MMAT criteria<br>*inconsistencies between quantitative and qualitative are not addressed in the study
Bernburg, Groneberg , Mache (2019)	Scientific article<br><br>Level II[1]	To develop, implement and evaluate the effectiveness of a mental health intervention programme on nurses' perceived job stress, perceptions of individual coping skills and the quality of patient relations in a group of psychiatric hospital departments.	Randomized controlled trial	Pre and post intervention surveys were applied using Perceived Stress Questionnaire; the Brief Resilient Coping Scale; the Self- Efficacy, Optimism and Pessimism Questionnaire; Emotion Regulation Skills Questionnaire and the German Quality of Relationship Inventory – at baseline , follow up after 3 months, 6 months and after 12 months.	After the intervention being successfully implemented, psychiatric nurses significantly increased their perception about job stress, emotion regulation skills, resilience, and self-efficacy; quality of patient-relationship were significantly higher ($p < 0.05$). The results indicate that self- care skills training may improve such as resilience and self-efficacy.	4*/5 of the MMAT criteria<br>*outcome assessors are not blinded to the intervention provided
Morales, Brown (2019)	Scientific article<br><br>Level VI[1]	To describe the development of the Care for the Caregiver Program in a 10 hospital system.	Qualitative	Description of the "Care for the Caregiver" programme and of practical scenarios after the programme was been activated.	Both described scenarios of support after stressful events indicate that the programme has been useful and well received by the clinicians.	4*/5 of the MMAT criteria<br>* 1 criteria was rated as can't tell : coherence between qualitative data sources, collection, analysis and interpretation is not applicable in this study
Cobos-Vargas, Pérez-Pérez, Núñez-Núñez, Casado-Fernández, Bueno-Cavanillas (2022)	Scientific article<br><br>Level VI[1]	To describe the Procedure for Serious Adverse Events (PSAE), integrated into a sentinel event operational procedure and to describe its accumulated results over two years of implementation in a university hospital.	Mixed method	Collection of quantitative data related with programme application, characteristics of involved HCWs and adverse events; HCWs qualitative feedback on programme improvement solutions and	The programme had a positive impact on patient safety culture and it increased the adverse event reporting in the hospital. The level of programme acceptance was high and HCWs valued to be individually interview after their involvement in the event and to contribute for future improvement actions.	4*/5 of the MMAT criteria<br>*inconsistencies between quantitative and qualitative are not addressed in the study

				their experience after attending the programme.		
Hinzmann, Forster, Koll-Krüsmann, Schießl , Schneider, Sigl-Erkel et al (2022)	Scientific article<br><br>Level VI[1]	To evaluate the burden, benefits, and mechanisms of action of a telephone support service for HCWs (PSU-HELPLINE).	Mixed methods	Application of two surveys: i) quantitative survey during the call(socio-demographic data, current stress ,relief from the counselling interview); ii) meta-questionnaire (to document the call and for quality assurance proposes) . Memory transcripts for thematical analysis were also collected.	The PSU-HELPLINE was mainly used for processing serious events and in phases of overload. The programme was well received by a specific region of Germany and callers consider it useful and supportive service.	4*/5 of the MMAT criteria<br>*inconsistencies between quantitative and qualitative are not addressed in the study

Complementary table 3 - Characteristics of the included interventions

Note: We have contacted the authors to complete the missing information, however some of the authors didn't reply to our contact.

Author (Year of publication)	Starting year of the intervention	Duration of the intervention	Country and Setting	Target population	Aim of the intervention	Main outcomes related with the intervention
Allen,Spencer , McEwan,Catarino, Evans, Crooks et al (2020)	Missing data	6 months	UK - Mental health service, inpatient acute psychiatric unit	Nursing care, healthcare assistance, occupational therapy, psychiatry, psychological assistance, domestic support building states, administration services, crisis resolution , home treatment team and perinatal mental health team	To understand experiences of emotional distress and reflecting on its impact and ways of coping	**i) Evaluation of Schwartz Rounds*:** From an average evaluation form response was 3.42 out of 4;<br>**ii) Experience of the participants after attending Schwartz Rounds:** In the Rounds, HCWs could express their emotions in an non-judgemental and empathic environment. Rounds were considered helpful, insightful and relevant;<br>**iii) Long-term follow-up of 6 years**: Rounds were still rated positively.<br>*Based on the following criteria: plan to attend the Rounds again, gained insight into thoughts and feelings, facilitator helped the discussion, the overview and presentation of the Rounds were helpful, it helped to improve the relation with colleagues in the work, the knowledge was useful for caring patients, the case was relevant to the clinical work
Bryant (2022)	2021	13 months	US - Intensive care unit, lactation services, child-life services, paediatrics medical surgical units	Staff members from lactation services and paediatric medical-surgical units. child life specialists, physicians, nurses, respiratory therapists, social workers, clinical associates, pharmacist and chaplains	To raise awareness of SVP and SupportingYOU. To increase resiliency by establishing a proactive culture to manage critical incidents prior they occur	**i) Staff perception on resiliency**: 100% of the surveyed staff reported an enhanced perception of resilience (p<0,001, CI 95%);<br>**ii) Staff awareness of SVP** significantly increased (p<0,001, CI 95%);<br>**iii) Staff awareness of SVP resources and of SupportingYOU**: it was limited since that most of participants were already aware of it;<br>**iv) Sense of preparedness in handling critical incidents**: there was an increased sense of managing emotional response after critical incidents among the participants (p<0,01, CI 95%);<br>**v) Feeling cared by the institution –** participants felt care for in the workplace (p<0,01, CI 95%).
Civil, Hoskins (2022)	2018	36 months	New Zealand – Department of Anaesthesia and Pain Medicine	Anaesthetists, surgeons, anaesthetist technicians, theatre nurse coordinator/charge nurse; theatre and recovery room nurses and midwifes	To provide peer-led group psychological first aid to full theatre team stressful events?	**i) Number of participants that attended the defuse:** over 200 members;<br>**ii) Number of interventions** : 28 defuse interventions have been delivered;<br>**iii) Participants feedback of the defuse** : Defuses were rated through neutral to very helpful, all surveyed participants would attend a defuse again in the future and would recommend to colleague or a friend.

Edrees, Connors, Paine, Norvell, Taylor, Wu (2016)	2011	52 months (from november 2011 to march 2016)	US- Johns Hopkins Hospital	All hospital staff	To provide timely psychological first aid and emotional support after critical incidents based on a call system	**i) Frequency of encounters:** 119 encounters from November 2011 and march 2016;<br>**ii) Caller interaction:** rated as excellent 66,7% or neutral (22,8%).
Finney, Jacob, Johnson, Messner, Pulos, Sviggum (2021)	2018	23 months (from July 2018 to June 2020)	US- Department of anaesthesiology at a large academic institution in the Midwest	Anaesthesiologist, certified nurse anaesthetics , residents, students registered nurse anaesthetics	To provide three-tiered support psychological first aid : first level given by peer and leadership at the local, second level provided by trained peer supporters; third level was provided by external services (from the institution or outside the institution)	**i) Experience after receiving peer support:** From 31 surveyed participants, 25 (80.0%) evaluated the received support as "extremely" or "very beneficial", and 28 (90.3%) referred that were "extremely" or "very satisfied" with their experience; 30 (96.8%) would recommend HELP programme to a colleague;<br>**ii) HELP Programme Activations for Peer Support:** 91 electronic activations were utilized to assist 179 HCWs (the most common events leading to HELP activation were intraoperative patient demise, cardiac arrest, and pediatric care);<br>**iii) Trained peer support self-assessments:** From 85 surveyed peer supporters, 81 (95.3%) felt satisfied with how the peer support encounter turned out; 19 (22.4%) felt that they needed additional training and experience, 80 (94.1%) felt comfortable with their knowledge and skills as a peer supporter.
Foreman (2014)	Missing data	Info missing	US- Family birth centre Wilcox Women's Pavillon at Legacy Good Samaritan Hospital	All perinatal nurses of the family birth caring for women and newborns in all phases of childbearing	To create a critical event plan and implement it in the centre for helping staff to organize and manage critical events	**i) Experience on the use of the Critical Event Plan:** Nurses found it very useful and helpful**.**
Graham, Zerbi, Norcross, Montross-Thomas, Lobbestael , Davidson (2019)	Missing data	3 months	US- 4 target units in an academic medical centre: telemetry unit, emergency department, neonatal ICU and medical and neurologic ICU	Staff and physicians employed in the units	To provide emotional first aid in the workplace after critical events	**i) Programme activation :** 38 activations of the programme;<br>**ii) Experience after the programme:** all surveyed staff found it helpful and would recommend it to other colleagues; HCWs referred an improvement in feeling cared-for and also noted the sense of safety at the workplace;<br>**iii) Quality of life assessment and job satisfaction**: No significant changes were demonstrated before and after the intervention in Quality of Life Scores or job satisfaction. One suicide was prevented;<br>**iv) Organizational changes :** Debriefings started to be requested after significant events affecting the entire department.
Johnson, Simms-Ellis, Janes, Mills, Budworth, Atkinson	2018	7 months	UK- National Health Services (NHS) trust sites and University of Leeds	HCWs or students that complete an education programme: midwives, paramedics , obstetrics and gynaecology trainee	To strengthen resilience and preparedness in dealing with adverse events : more flexible thinking, higher self-	**i) Experience after the programme:** workshop was useful, relevant and adequate in length**.** The participants highly valued the peer learning, the level of engagement and the format of the workshop delivery. The coaching call was critical to the consolidation of the knowledge and to

,Harrison (2020)				doctors , paediatric trainee doctors, paediatric consultant, physician associate students and sonography and mammography students.	esteem, better explanatory style	understand how to apply the acquired skills in practice. Knowledge about coping strategies led to a significant increase in self-perceived resilience, as well as confidence in coping with adverse events.
Krzan, Merandi, Morvay, Mirtallo (2015)	2013	5 months	US- pharmacy department at Nationwide Children's Hospital (NCH): one main inpatient pharmacy, inpatient pharmacy satellites, two outpatient pharmacies and home care pharmacy	Staff from all areas of pharmacy services	To provide three-tiered support psychological first aid (based on Susan's Scott model) to support HCWs involved in adverse drug events, patient-related injuries, and other traumatic work experiences	**i) Activation of the programme:** 3 respondents personally used the programme and 11 had recommend it;<br><br>**ii) Experience after the programme :** 85% of the pharmacy (95 of 112 respondents) refer that the department had benefit from the programme's implementation.
Merandi, Liao, Lewe, Morvay, Stewart, Catt, Scott (2017)	2012	60 months (5 years)	US- Nationwide Children's Hospital (NCH)	Staff from Nationwide Children's Hospital (NCH): all inpatient units as well as urgent cares, outpatient primary care clinics, and ambulatory specialty clinics	To provide three-tiered support psychological first aid (based on Susan's Scott model) to support HCWs involved in adverse drug events, patient-related injuries, and other traumatic work experiences	**i) Activation of the programme:** 21 group encounters were documented since November 2013;<br><br>**ii) Quantitative description of peer encounters:** 62% of peer encounters occurred in the emergency department, and 8% in pediatric intensive care unit and cardiothoracic intensive care unit ; nurses have the highest number of peer support encounters. Patient death and emotional stress were the most common reasons for peer encounter.
Lane, Newman, Taylor, O'Neill, Ghetti, Woltman, Waterman (2018)	2014	33 months	US- Two Hospitals affiliates of the Washington School of Medicine: Barnes -Jewish Hospital and St. Louis Children's Hospital	Doctors, junior doctors, physician associates, nurse practitioners, and registered nurse anaesthetists, trainees providing care in the inpatient and outpatient settings	To facilitate the support of clinicians who have been involved in an adverse event or another adverse outcome during medical care	**i) Programme activation:** 165 clinicians were referred to the programme;<br><br>**ii) Median number of interactions per month:** 4.8 referrals per month (Range 0-12).
Mellins, Mayer, Glasofer, Devlin, Albano, Nash et al (2020)	2020	3 months	US-Large urban , academic, tertiary care - Columbia University Irving Medical Centre (CUIMC)	All CUIMC clinical and non-clinical staff, residents and faculty students	To provide peer support after stressful situations and enhance resilience of HCWs	**i) Emotional distress assessment:** The average emotional distress had significantly decreased (p<0.05)**;**<br><br>**ii) Perceived helpfulness of the peer support group:** Perceived helpfulness was high (76% rating helpfulness as "quite a bit" or "extremely."); all respondents (with the exception of two) recommended the support group to a colleague.

Merandi, Winning , Liao, Rogers, Lewe, Gerhardt (2018)	2015	12 months	US-7 neonatal intensive care units (NICUs) from quaternary-care, paediatrics hospital	All staff from the 7 NICUs	To provide three-tiered support psychological first aid (based on Susan's Scott model) to support HCWs involved in adverse drug events, patient-related injuries, and other traumatic work experiences	**i) Attendance to the programme:** A small number of healthcare providers (n=6) used the program. Some HCWs (not quantified) did not find the programme accessible.<br><br>**ii) Satisfaction with the programme:** The majority of the participants (56.3%, n=9) reported moderate benefit of the programme. From 250 surveyed HCWs in the units, 73.2% indicated that the NICUs benefited at least a little from the SV peer support programme. The manager reported that the programme enhanced the team leadership.
Rivera-Chiauzzi,Smith,Moore-Murray, Lee, Goffman, Bernstein, Chazotte (2022)	2015	8 months	US- Department of obstetrics & Gynaecology and Women Health	HCWs and residents (physicians and nurses) who experienced an obstetric adverse outcome.	To provide peer support to HCWs and residents, who have experienced an obstetric adverse outcome, through phone calls	**i) Attendance to the programme :** Out of 34 programme activations, 23 participants were placed in the enhanced group and 19 were assigned in the routine group;<br><br>**ii) Use of support resources and perception of its helpfulness:** Peer support was the most common source of assistance (p<0.05); departmental leadership was considered one of the most helpful resources for the enhanced support group;<br><br>**iii) Identified barriers:** Time (P = 0.26) was reported as a constraint by participants in the enhanced group, and routine group reported privacy concerns (P = 0.39) and stigma (P = 0.12)**;**<br><br>**iv) Effect of the programme on the stage of recovery:** At 6-month follow-up, all participants in the enhanced group reported that they were thriving after the event;<br><br>**v) Duration of peer support interventions:** most participants required less than 3 months of support to recover from the event.
Roesler, Ward, Short (2009)	2006	No clear information (estimated time : approx. 6 months)	US- Neonatal Intensive Care Unit (NICU), Methodist Hospital of Indianapolis	Staff of the NICU	To provide an institutional incident response after a paediatric serious adverse event	**i) Staff and unit recovery:** The pharmacist technician and 5 out of 6 nurses affected by the incident were back to work after the adverse event (1 nurse didn't return to work for familiar reasons). None of the affected HCWs experienced job changes. Debriefing and healing sessions were essential to the staff unit overcome the situation;<br><br>**ii) Impact on work culture**- Reinforcement of the culture of disclosure after applying the protocol.
Schrøder, Bovil,, Jørgensen, Abrahamsen (2022)	2018	18 months	Denmark-2 departments at Odense University Hospital: Department of Obstetrics and Gynaecology (OB-GYN) and Svendborg	All midwives in the OB-GYN and all physicians at the IME	To provide one-on-one peer support to HCW following a stressful event, including adverse events	**i) Attendance to the programme:** Out of 156 respondents, 26 of them reported using one of their buddies during the study period**;**<br><br>**ii) Evaluation of the Buddy Study programme in the department:** The programme encouraged more attentiveness among participants towards one another following adverse events (n=82, 52.6%) ; have

			and Internal Medicine and Emergency Department (IME)			contributed to more inter-collegial talks about adverse events (n=57,36.5%) ; sense of more openness to talk with colleagues about feelings in the aftermath of adverse events (n=65, 41.7%) ; increased willingness to ask for leadership support (n=53, 34.0%);<br><br>**iii) Overall experience with the programme:** an open and compassionate culture was encouraged; increased attentiveness to the staff wellbeing ; increased sense of safety (n=91, 58.3%).
Shapiro, Galowitz (2016)	2012	47 months (January 2012 and December 2015)	US- Brigham and Women's Hospital (BWH)	HCWs of Brigham and Women's Hospital	To provide one-on-one peer support or group peer support (if a team is likely to be affected), after a stressful event, including adverse events	**i) Activation of the programme:** 220 outreach calls to one-on-one peer support;<br><br>**ii) Attendance to the group sessions:** 240 clinicians participated in multidisciplinary group peer support sessions.
Thompson, Hunnicutt, Broadhead, Vining, Aroke (2022)	May, 2020 through October, 2020	1 month (trial period)	US – Anaesthesia department of the large academic centre	Certified registered nurses of the anaesthesia department.	To provide a peer support program to decrease SV distress after stressful events	**i) Frequency of the encounters:** Over the course of one month, a total of 8 peer support encounters were reported;<br><br>**ii) HCWs' distress**: It wasn't possible to observe a significant change in distress level after one month period, however the study found a statistically significant relationship between increased distress and insufficient colleagues support.
El Hechi , Bohnen, Westfal, Han, Cauley, Wright, Schulz et al (2020)	Missing data	12 months	US-Department of surgery at a tertiary academic medical centre	Surgeons and surgical trainees from general surgery, transplantation, pediatric surgery, thoracic surgery, cardiac surgery, vascular surgery, and trauma/acute care surgery.	To provide a peer support - based on three-tiered support psychological first aid - to surgeons and surgical trainees dealing with intraoperative adverse events, catastrophic patient outcomes, and/or long- term litigation cases.	**i) Programme activation:** 47 outreach calls;<br><br>**ii) Evaluation of the programme impact:** The majority of surgical staff was satisfied with the following domains – programme's confidentiality (89%), safe/trusting environment (73%,), timeliness of the intervention (83%). It was also perceived a positive impact on the department's culture, as it raised awareness about the importance of supporting colleagues facing difficult situations at work, and fostered a general sense of "safety and support" in the workplace.
Mira, Carrillo, Guilabert, Lorenzo, Pérez-Pérez, Silvestre et al (2017)	2015	15 months	Spain – without a specific location (online programme)	HCWs from hospitals and primary care that access the webpage of the online programme	To provide an online preventive programme to mitigate the impact of severe adverse events in HCWs and raise awareness about the SV phenomenon.	**i) Knowledge improvement (main outcome):** After completing the informative and demonstrative package, users significantly improved their knowledge about patient safety terminology, impact and prevalence of adverse events, SV support strategies and recommended actions following a severe adverse event (*P*<0.001).

Scott, Hirschinger, Cox, McCoig, Hahn-Cover, Epperly et al. (2010)	2009	10 months	US-University of Missouri Healthcare	University of Missouri Health Care faculty and healthcare staff	To provide a peer support programme to facilitate the second victim's transition through the six stages of emotional recovery	**i) Frequency of the encounters**: 49 encounters with forYOU Team members ,13 of which involved referrals to external support; there were 6 team debriefings with an average of 15 HCWs each. The average duration of the encounter with forYOU Team members lasted 30 minutes, while debriefings extended to 77 minutes.
Schuster (2021)	End of 2019	6 months	US- Department of Hematology/Oncology /Stem Cell Transplant Unit- Boston Children's Hospital – 2 separate units: a 30-bed hematology/oncology unit and a 14-bed stem cell transplant unit. Both units are under one hematology/oncology/ bone marrow transplant department within the inpatient pediatric free-standing children's hospital.	Multidisciplinary staff caring for hematology/oncology patients and their families (registered nurses, advanced practice nurses, clinical assistants, physicians, dieticians, child life specialists, environmental services, food tray distributors, patient experience representatives, social workers, resource room staff, chaplains, pain service staff, supply restockers, and students on the floor).	To provide peer to peer support program to promote a safer, supportive and resilient workplace culture, improve staff wellbeing and decrease the frequency of contacts among colleagues for work-related support outside of working hours to cope with stressful situations	**i) Frequency of the programme interventions:** 98 HART shifts;<br><br>**ii) Outcomes related with HART application:** improvement of HCWs' well-being, the number of HCWs reaching out coworkers for support outside working hours decreased after implementing HART; number of breaks increased during the work shifts, the use of hospital resources increased after HART coaches recommend them;<br><br>**iii) Satisfaction with the work-related support after HART implementation:** 25.6% of participants reported to be extremely satisfied with the received support; 49.4% of HCWs felt more supported by leadership.<br><br>**iv) Interactions resolutions:** The majority of interactions were solved in real time (83.2%);<br><br>**v) Subjective Feedback:** The programme fostered a safe, supportive and open work environment. HCWs felt more connected to their colleagues in the workplace.
Calder-Sprackman; Kumar; Gerin-Lajoie; Kilvert; Sampsel (2018)	2014	24 months	Canada – Department of emergency medicine , university of Ottawa	Residents from the emergency department at the university of Ottawa	To provide a peer-support sessions to improve residents wellbeing and create a supportive and resilient workplace	**i) Perception of change after the support programme**: From a total of 20 surveyed HCWs, 95% referred that support and companionship among residents increased after the support the sessions; 58,8% gained an increased awareness of coping strategies to deal with challenges during residency ;<br><br>**ii) Impact of the Rounds on clinical practice:** Half of HCWs referred that the rounds helped them to reflect about their clinical practice, a quarter of HCWs expressed that the rounds had not significantly impacted their clinical practice. Nevertheless, they acknowledged the value of having this type of support available to them;

						**iii) Perception of stress, anxiety and burnout**: 20% of HCWs decreased sensation of burnout and 10% decrease their perceived stress and anxiety;<br><br>**iv) Programme recommendation:** 89% of respondents would recommend ice cream rounds to other colleagues
Peterson, Bergström, Samuelsson, Åsberg, Nygren (2008)	2002	2 months	Sweden- 3 hospitals of the county council	Physicians, registered nurses, nursing assistants, social workers, occupational therapists, physiotherapists, psychologists, dental nurses and hygienists, dentists, service staff, administrators, teachers and technician. participants scored above the 75th percentile on the exhaustion dimension of the Oldenburg Burnout Inventory	To provide a reflecting peer-support group to discuss and reflect on work-related stress and burnout; to help to find out alternative ways to handle perceived stressful situations on an individual level; to provide an opportunity for mutual support between colleagues, to share and compare experiences and learn from each other.	**i) Frequency of the encounters:** 8 peer-support groups with 5–8 participants in each group**;**<br><br>**ii) Perceived impact of the peer support group:** The space for reflection on 'real problems' was valued by the participants; increased knowledge about stress and coping strategies, increased sense of belonging/community; Increased self-confidence;the existence of structured group was appreciated, decreased stress symptoms and anger; behavioural change; sleep improvement;<br><br>**iii) Work-related symptoms and burnout:** Both groups showed an overall decrease from baseline to follow up after 12 months in exhaustion, disengagement, depression and anxiety measures;<br><br>**iv) General health and vitality:** both increased after the intervention;<br><br>**v) Change in work conditions**: statistical significant difference was found in participation at work and support at work after 12 month follow-up.
Rubin, Rassman (2021)	2020	"During spring"	US-large urban healthcare system: Clinical education and practice department in Swedish Health Services	Clinical and non-clinical staff (nurse educator, professional development specialist, programme manager, student intern) and disciplines (nursing, social work) within the Clinical Education and Practice department.	To provide informational and emotional support to help frontline staff thrive in the first wave of the Covid 19 Pandemic	**i) Programme utilization:** 13 out 71 HCWs participated in the programme;<br><br>**ii) Evaluation of programme's resources:** Approximately 85% of surveyed HCWs agreed or strongly agreed that there were adequate resources to support the HCWs during and after stressful events in the healthcare organization. All respondents agreed that assisting the cAIR video presentation was a valuable use of their time. The nature of the content and dedicated time for staff support stood out as the most helpful aspects of the video presentation. Most respondents improved their knowledge and/or skills with the cAIR resources.

For peer review only - http://bmjopen.bmj.com/site/about/guidelines.xhtml

Bernburg, Groneberg , Mache (2019)	Missing data	3 months	Germany - psychiatric hospital departments	Nurses full time working in psychiatric hospital department	To provide a mental health promotion intervention to develop self-care skills in psychiatric nurses	**i) Programme's attendance:** 44 nurses were part of the intervention group and 42 nurses of the control group;<br>**ii) Perceived job stress:** intervention group perceived lower levels of stress after attending the programme;<br>**iii) Relation with patients**: After the programme, significant improvements in nurses' relationship with their patients and lower levels of perceived conflicts were observed ($p < 0.05$);<br>**iv) Emotion regulation:** Large effects on emotion regulation skills were found in the first follow up (3 months) and medium effects sizes were found after 6 and 12 months;<br>**v) Resilience and self-efficacy:** statistically difference was found in these measures within groups (p<0.05)**;**<br>**vi) Final course evaluation:** Overall satisfaction with the training (1.39; 1-best score, 5-worse score); nurses displayed a strong level of motivation and interest in learning self-care techniques and applying them in their work. All participants verified that the training was worth attending (learning was meaningful and motivating for selfcare).
Morales, Brown (2019)	Missing data	No info	US- 10-hospital health system	Clinicians working in the 10-hospital system	To provide immediate emotional first aid after a serious adverse event and coach to HCWs on how to respond to patients and their families in a timely, empathetic, consistent, and patient-centred way.	**i) Process and outcome's results of two different scenarios have been described:**<br>**Scenario 1 (death after health condition deterioration of a patient affected all ICU nursing team) -** Process outcomes: programme activation was done by the nursing director; group sessions were applied to all nurses of the ICU , Employee Assistant Programme was provided to several nurses; Outcome results: after nurses had receive support from the programme, they described "feeling grateful for the opportunity to talk openly without judgment" about the event. Sessions also enabled for peer support among the nursing team members;<br>**Scenario 2 (intensivist involved in a stressful situation during intubation of a patient) -** Process outcomes: critical care unit director activated the programme; "Thinking of You" bag was delivered to the HCW in need. **Outcome results**: the intensivist expressed gratitude for both unit staff and members of the programme.
Cobos-Vargas, Pérez-Pérez, Núñez-Núñez, Casado-	2020	24 months	Spain- Clinico San Cecilio University Hospital	Hospital staff involved in any type of serious clinical incidents that caused or could have been caused (death or	To provide support to patients and their families, to HCWs and the healthcare institution after serious events, and to investigate and develop improvement	**i) Programme activations:** From 25 activations, 23 severe adverse events were investigated;<br>**ii) Frequency of the peer support encounters:** 1 to 17 HCWs per adverse event received trained peer support;

For peer review only - http://bmjopen.bmj.com/site/about/guidelines.xhtml

Fernández, Bueno-Cavanillas (2022)				serious harm of a patient)	actions, based on Susan's Scott model.	**iii) Time from the event until the activation of the programme:** ranged from 12h to one week;<br>**iv) Number of second victims identified:** 34.8% (n=47) were identified as second victims;<br>**v) Referral for third level of support** : 7 cases (14.9%) were referred, however 4 refused it;<br>**v) Participants experience after participating in the programme:** increased sense of safety in the workplace; participants valued the interview provided in the second level of support and being able to contribute for the improvement actions after the adverse event occurrence;<br>**vi) Feedback for programme improvement:** The programme must be disseminated for all HCWs to have access to it; leaders should be trained to be more aware of the problem; first level of approach is identified has a problem in the units.
Hinzmann, Forster, Koll-Krüsmann, Schießl , Schneider, Sigl-Erkel et al (2022)	2020	24 months	Germany- health and emergency services	Clinicians and managers from the health and emergency services	To stabilize and restore the ability to act in highly stressful situation including adverse events, preserving clinical teams health and work capacity – in case of need , to transfer HCWs and managers with for specific treatment , initial structures of standard psychotherapeutic care in a low-threshold and timely manner.	**i) Evaluation of the programme:** In 81.4% of the cases the programme provided strong and very strong support to the callers in coping with the burden after a stressful event;<br>**ii) Follow up call after the first call :** 52.9% of callers needed a follow-up appointment with the same supporter after the first call;<br>**iii) Qualitative feedback after the programme** : there was an increased understanding of one's own reactions; openness to listen and understand that emotions are welcomed in a safe environment; knowing where to turn for help; enhanced mutual understanding and peer validation, as well as self-awareness and self-reflexion; leadership involvement increased.

For peer review only

Preferred Reporting Items for Systematic reviews and Meta-Analyses extension for Scoping Reviews (PRISMA-ScR) Checklist

SECTION	ITEM	PRISMA-ScR CHECKLIST ITEM	REPORTED ON PAGE #
**TITLE**			
Title	1	Identify the report as a scoping review.	1
**ABSTRACT**			
Structured summary	2	Provide a structured summary that includes (as applicable): background, objectives, eligibility criteria, sources of evidence, charting methods, results, and conclusions that relate to the review questions and objectives.	2
**INTRODUCTION**			
Rationale	3	Describe the rationale for the review in the context of what is already known. Explain why the review questions/objectives lend themselves to a scoping review approach.	4
Objectives	4	Provide an explicit statement of the questions and objectives being addressed with reference to their key elements (e.g., population or participants, concepts, and context) or other relevant key elements used to conceptualize the review questions and/or objectives.	4
**METHODS**			
Protocol and registration	5	Indicate whether a review protocol exists; state if and where it can be accessed (e.g., a Web address); and if available, provide registration information, including the registration number.	4
Eligibility criteria	6	Specify characteristics of the sources of evidence used as eligibility criteria (e.g., years considered, language, and publication status), and provide a rationale.	Pages 5,6, Complementary table 1
Information sources*	7	Describe all information sources in the search (e.g., databases with dates of coverage and contact with authors to identify additional sources), as well as the date the most recent search was executed.	Page 5,Complementary table 1
Search	8	Present the full electronic search strategy for at least 1 database, including any limits used, such that it could be repeated.	Complementary table 1
Selection of sources of evidence†	9	State the process for selecting sources of evidence (i.e., screening and eligibility) included in the scoping review.	5,6
Data charting process‡	10	Describe the methods of charting data from the included sources of evidence (e.g., calibrated forms or forms that have been tested by the team before their use, and whether data charting was done independently or in duplicate) and any processes for obtaining and confirming data from investigators.	6,7
Data items	11	List and define all variables for which data were sought and any assumptions and simplifications made.	6

St. Michael's
Inspired Care.
Inspiring Science.

For peer review only - http://bmjopen.bmj.com/site/about/guidelines.xhtml

SECTION	ITEM	PRISMA-ScR CHECKLIST ITEM	REPORTED ON PAGE #
Critical appraisal of individual sources of evidence§	12	If done, provide a rationale for conducting a critical appraisal of included sources of evidence; describe the methods used and how this information was used in any data synthesis (if appropriate).	6
Synthesis of results	13	Describe the methods of handling and summarizing the data that were charted.	7
**RESULTS**			
Selection of sources of evidence	14	Give numbers of sources of evidence screened, assessed for eligibility, and included in the review, with reasons for exclusions at each stage, ideally using a flow diagram.	6,7
Characteristics of sources of evidence	15	For each source of evidence, present characteristics for which data were charted and provide the citations.	7
Critical appraisal within sources of evidence	16	If done, present data on critical appraisal of included sources of evidence (see item 12).	Page 7 and Complementary table 2
Results of individual sources of evidence	17	For each included source of evidence, present the relevant data that were charted that relate to the review questions and objectives.	7- 13
Synthesis of results	18	Summarize and/or present the charting results as they relate to the review questions and objectives.	Pages 7, 9-13 Complementary tables 2 and 3 ;
**DISCUSSION**			
Summary of evidence	19	Summarize the main results (including an overview of concepts, themes, and types of evidence available), link to the review questions and objectives, and consider the relevance to key groups.	14,15,16
Limitations	20	Discuss the limitations of the scoping review process.	15,16
Conclusions	21	Provide a general interpretation of the results with respect to the review questions and objectives, as well as potential implications and/or next steps.	16
**FUNDING**			
Funding	22	Describe sources of funding for the included sources of evidence, as well as sources of funding for the scoping review. Describe the role of the funders of the scoping review.	16

JBI = Joanna Briggs Institute; PRISMA-ScR = Preferred Reporting Items for Systematic reviews and Meta-Analyses extension for Scoping Reviews.
* Where *sources of evidence* (see second footnote) are compiled from, such as bibliographic databases, social media platforms, and Web sites.
† A more inclusive/heterogeneous term used to account for the different types of evidence or data sources (e.g., quantitative and/or qualitative research, expert opinion, and policy documents) that may be eligible in a scoping review as opposed to only studies. This is not to be confused with *information sources* (see first footnote).
‡ The frameworks by Arksey and O'Malley (6) and Levac and colleagues (7) and the JBI guidance (4, 5) refer to the process of data extraction in a scoping review as data charting.
§ The process of systematically examining research evidence to assess its validity, results, and relevance before using it to inform a decision. This term is used for items 12 and 19 instead of "risk of bias" (which is more applicable to systematic reviews of interventions) to include and acknowledge the various sources of evidence that may be used in a scoping review (e.g., quantitative and/or qualitative research, expert opinion, and policy document).

From: Tricco AC, Lillie E, Zarin W, O'Brien KK, Colquhoun H, Levac D, et al. PRISMA Extension for Scoping Reviews (PRISMAScR): Checklist and Explanation. Ann Intern Med. 2018;169:467–473. doi: 10.7326/M18-0850.

For peer review only - http://bmjopen.bmj.com/site/about/guidelines.xhtml

BMJ Open

Key factors for effective implementation of healthcare workers support interventions after patient safety incidents in health organisations - a scoping review

Journal:	*BMJ Open*
Manuscript ID	bmjopen-2023-078118.R1
Article Type:	Original research
Date Submitted by the Author:	19-Oct-2023
Complete List of Authors:	Guerra-Paiva, Sofia; NOVA University Lisbon NOVA National School of Public Health, Public Health Research Centre, Comprehensive Health Research Center, CHRC<br>Lobão, Maria João; NOVA University Lisbon NOVA National School of Public Health, Public Health Research Centre, Comprehensive Health Research Center, CHRC; Hospital de Cascais Dr Jose de Almeida, Internal Medicine Department,<br>Simões , Diogo ; NOVA University Lisbon NOVA National School of Public Health, Public Health Research Centre, Comprehensive Health Research Center, CHRC; Public Health Unit of ACES Almada-Seixal<br>Fernandes, Joana; NOVA University Lisbon NOVA National School of Public Health, Public Health Research Centre, Comprehensive Health Research Center, CHRC<br>Donato, Helena; Centro Hospitalar e Universitário de Coimbra, Coimbra – Portugal, Documentation and Scientific Information Service<br>Carrillo, Irene; Miguel Hernandez University of Elche, Health Psychology; FISABIO,<br>Mira, José ; Miguel Hernandez University of Elche, Health Psychology; Salud Alicante-Sant Joan Health District,<br>Sousa, Paulo; NOVA University Lisbon NOVA National School of Public Health, Public Health Research Centre, Comprehensive Health Research Center, CHRC
**Primary Subject Heading**:	Public health
Secondary Subject Heading:	Health services research, Health policy
Keywords:	PUBLIC HEALTH, HEALTH SERVICES ADMINISTRATION & MANAGEMENT, Quality in health care < HEALTH SERVICES ADMINISTRATION & MANAGEMENT, Health & safety < HEALTH SERVICES ADMINISTRATION & MANAGEMENT

SCHOLARONE™
Manuscripts

I, the Submitting Author has the right to grant and does grant on behalf of all authors of the Work (as defined in the below author licence), an exclusive licence and/or a non-exclusive licence for contributions from authors who are: i) UK Crown employees; ii) where BMJ has agreed a CC-BY licence shall apply, and/or iii) in accordance with the terms applicable for US Federal Government officers or employees acting as part of their official duties; on a worldwide, perpetual, irrevocable, royalty-free basis to BMJ Publishing Group Ltd ("BMJ") its licensees and where the relevant Journal is co-owned by BMJ to the co-owners of the Journal, to publish the Work in this journal and any other BMJ products and to exploit all rights, as set out in our licence.

The Submitting Author accepts and understands that any supply made under these terms is made by BMJ to the Submitting Author unless you are acting as an employee on behalf of your employer or a postgraduate student of an affiliated institution which is paying any applicable article publishing charge ("APC") for Open Access articles. Where the Submitting Author wishes to make the Work available on an Open Access basis (and intends to pay the relevant APC), the terms of reuse of such Open Access shall be governed by a Creative Commons licence – details of these licences and which Creative Commons licence will apply to this Work are set out in our licence referred to above.

Other than as permitted in any relevant BMJ Author's Self Archiving Policies, I confirm this Work has not been accepted for publication elsewhere, is not being considered for publication elsewhere and does not duplicate material already published. I confirm all authors consent to publication of this Work and authorise the granting of this licence.

For peer review only

Key factors for effective implementation of healthcare workers support interventions after patient safety incidents in health organisations - a scoping review

Sofia Guerra-Paiva[a], Maria João Lobão[a,b], Diogo Godinho Simões[a,c], Joana Fernandes[a], Helena Donato[d], Irene Carrillo[e], José Joaquin Mira[e,f], Paulo Sousa[a]

Corresponding author:

Sofia Guerra-Paiva[a]

https://orcid.org/0000-0002-4311-3799

Avenida Padre Cruz 1600-560 Lisboa

sg.paiva@ensp.unl.pt

a. NOVA National School of Public Health, Public Health Research Centre, Comprehensive Health Research Center, CHRC, NOVA University Lisbon, Lisbon, Portugal
b. Internal Medicine Department, Hospital de Cascais, Cascais, Portugal
c. Public Health Unit of ACES Almada-Seixal, Almada, Portugal
d. Documentation and Scientific Information Service, Centro Hospitalar e Universitário de Coimbra, Coimbra, Portugal
e. Department of Health Psychology, Universidad Miguel Hernández de Elche
f. Alicante-Sant Joan Healthcare District, 03013 Alacant, Spain

Word count: 6217

For peer review only

ABSTRACT:

Objectives: To map and frame the main factors present in support interventions successfully implemented in health organisations in order to provide timely and adequate response to healthcare workers (HCW) after patient safety incidents (PSIs).

Design: Scoping review guided by the six-stage approach proposed by Arksey and O'Malley and by PRISMA-ScR.

Data sources: CINAHL, Cochrane Library, Embase, Epistemonikos, PsycINFO, PubMed, SciELO Citation Index, Scopus, Web of Science Core Collection, reference lists of the eligible articles, websites and a consultation group.

Eligibility criteria for selecting studies: Empirical studies (original articles) were prioritised. We used the Mixed Methods Appraisal Tool (MMAT) Version 2018 to conduct a quality assessment of the eligible studies.

Data extraction and synthesis: A total of 9,766 records were retrieved. We assessed 156 articles for eligibility in the full-text screening. Of these, 29 articles met the eligibility criteria. The articles were independently screened by two authors. In the case of disagreement, a third author was involved. The collected data was organied according with the acronym OPERA (Organisational factors, People, Environment, Recommendations from other studies, Attributes of the support interventions). We used Endnote to import articles from the databases and Rayyan to support the screening of titles and abstracts.

Results: We found that the existence of an organisational culture based on principles of trust and non-judgement, active leadership involvement, multidisciplinary teams, and strong dissemination were crucial factors for the effectiveness of the programmes' implementation. Training should be provided for peer supporters and leadership to facilitate the response to HCW needs. Regular communication among the implementation team, existence of protected time, funding and continuous monitoring are useful elements to guarantee the success of the programme over time.

Conclusion: HCWs' well-being depends on an adequate implementation process, ensuring a complex group of interrelated factors to support them after PSIs.

Registration number: 10.17605/OSF.IO/RQAT6.

Keywords: support programs; health personnel; second victim; clinical error; patient safety

only

Strengths and limitations of this study:

- The inclusion and exclusion criteria were defined in accordance with a preliminary search strategy, guided by the population, concept and context (PCC), as recommended by the JBI for scoping reviews;
- We didn’t restrict language and period of time to avoid having selection bias and compromise the validity and reliability of the findings;
- The data collection was limited to five interrelated dimensions (Organisational factors, People, Environment, Recommendations from other studies, Attributes of the support interventions);
- We used the MMAT assessment tool to evaluate the quality of the included studies, however some of the criteria couldn’t be fully applied in some specific cases;
- We included five experts from different countries to complement the literature search with additional sources of information;

For peer review only

INTRODUCTION

It's estimated that 10,4% to half of the professionals working in health sector will experience at least once in their career the second victim phenomenon (SVP) (1,2) defined as "any healthcare worker (HCW), directly or indirectly involved in an unanticipated adverse patient event, unintentional healthcare error, or patient injury, and who becomes victimised in the sense that they are also negatively impacted" (3). These types of incidents, with an unintended or unexpected nature, can harm patients (first victims of an adverse event) or pose a risk to the system (near miss) (4,5).

HCWs play a crucial role in patient care and they can be seriously affected when a patient safety incident (PSI) happens. PSIs can impact HCW's quality of life (2,6,7), in particularly their physical and psychological well-being (8,9). A study published in 2020, shows that the most prevalent symptoms in HCWs after PSIs were troubling memories, anxiety/concern and anger toward themselves (9). Work satisfaction, confidence in their abilities (2) and work performance (7,10) can also be seriously impacted by this type of incidents. It can result in turnover intentions and absenteeism (11) and in the most severe cases can lead to suicide (12).

Instituitional support systems are increasingly being implemented in order to provide an immediate and empathic response to HCWs after stressful situations such as PSIs. Health organisations are recognising the importance of this support, given its important impact on the organisational culture (13), patient safety (PS) and quality of care (14–16) and also on the economic perspective (17). It is well-established that poor HCWs' well-being have a strong influence on the reoccurrence of PSIs (14). Therefore, prioritizing interventions that effectively support HCWs after stressful situations can prevent future healthcare incidents and increase patient safety.

The first reported support programmes were implemented in the United States in 2006 and since then, they have been gradually multiplying all over the world (18) In recent years there has been a growing number of publications describing the implementation of these types of programmes and practices with the overall aim of decreasing emotional and psychological distress in HCWs. A systematic review found that HCWs seek this type of support not only after being involved in PSIs, but also when facing other distressing situations (e.g. emotional distress, torpid evolution of a patient, personal crises, intraoperative mishaps) (9).

Although support interventions have demonstrated their benefits and utility, there is still limited research on finding what the common elements present in the development and implementation process of successful interventions are. A toolkit was introduced in 2010 to provide guidance on the implementation of programmes to support HCWs that have been negatively impacted by PSIs (19). The development of this toolkit was an important step in assisting with the implementation of healthcare support programmes and it can be adjusted to any type of healthcare organisation (19). However, no study has been published focused on reviewing the existing evidence to understand the main factors that contribute for an effective implementation of these types of support interventions.

Evidence shows that establishing a set of elements for implementing interventions does not ensure itself that it will be effectively introduced in daily usage (20,21). The success of interventions in health organisations highly depends on an adequate design, implementation and evaluation (22). One of the main aims of implementation science is to understand what are the factors that might affect the effectiveness and sustainability of the interventions and what is the necessary implementation process to produce the expected effects (20). In this sense, learning from previous experience can facilitate practical application and contribute to more effective interventions (23).

Study rationale:

In this study we set out **to map and frame the main factors that underlie an effective implementation of support interventions** in order to provide timely and adequate response to HCWs that are physically and/or emotionally affected by PSIs (known as second victims) or similar distressing situations. We have defined five interrelated dimensions guided by five main research questions, further described in this study, and we organised them in the acronym OPERA (Figure 1). This framework helped to inform the planning and design of the scoping review, as well as the execution. The defined five domains were inspired on health policy triangle (HPT) framework to guide effective implementation of health policies (24). However, HPT is a theoretical model and in order to overcome the research-to-practice gap, we have incorporated the

implementation science principles and Donabedian's structure–process–outcome quality-of-care model, more recently adapted by Yano (25).

Objectives:

We aim to understand what existing organisational factors, relevant actors, contextual factors, operational attributes are present in interventions that were successfully implemented in health organisations to support HCW's after PSIs or other similar stressful events. We also aim to identify what are the recommendations from the included interventions for improving the effectiveness of the programmes implementation in health organisations.

METHODS

This scoping review is conducted using the six-stage approach proposed by Arksey and O'Malley (26) and is guided by the Preferred Reporting Items for Systematic Reviews and Meta-Analysis Extension for Scoping Reviews to ensure the transparency of the results obtained (27) and follows The Joanna Briggs Institute Methodology for Scoping Reviews (28) .

All the methodological steps are described in further detail in the scoping review protocol published in a previous publication (29).

Stage 1 : Identifying the research question(s)

In this study we focus on the main research question :

- What are the key factors that contribute for an effective implementation of interventions to support HCWs after PSIs or other similar stressful situations in health organisations?

To answer the primary research question, five secondary questions were formulated based on the specific objectives and outcomes of interest of the study :

- What are the organisational factors that contribute for an effective implementation of these interventions?
- Who are the relevant actors that contribute for an effective implementation of these interventions?
- What are the contextual factors that contribute for an effective implementation of these interventions?
- What recommendations, as identified in previous studies, can be applied to effectively implement these interventions?
- What are the operational attributes that contribute for an effective implementation of these interventions?

Stage 2: Search strategy

A comprehensive search strategy using relevant electronic databases was developed with the support of a qualified research librarian. The search comprised Medical Subject Headings terms along with free-text keywords. We applied the search strategy in nine electronic databases and the last update was done in November 2022 (CINAHL, Cochrane Library, Embase, Epistemonikos, PsycINFO, PubMed, SciELO Citation Index, Scopus, Web of Science Core Collection). The applied search strategies in the electronic databases can be consulted in Supplementary Table 1. In addition to the database search, relevant websites were consulted and reference lists of the studies included in the full-text screening were screened to identify any other potential articles to include.

Stage 3: Study selection

We used Endnote to import articles from the different databases and we used Rayyan as tool to support the screening of titles and abstracts. The articles were independently and manually screened by two authors between April 2022 and February 2023. In the case of disagreement on article inclusion, a third author was involved to evaluate the paper independently and contribute to making a final decision.

We did not restrict the period of time or language of the included studies in order to reduce the selection bias and to undertake a comprehensive overview of the existing literature on a topic with still limited number of publications. Empirical studies (original articles) were prioritised along with systematic reviews and meta-analyses for collecting potential eligible studies. Grey literature (theses and other documents) were also considered eligible for the study.

The inclusion and exclusion criteria were defined in accordance with a preliminary search strategy, guided by the population, concept and context (PCC) (recommended by the JBI for scoping reviews (28)) and are further described in the published protocol of this study (29).

In this study we focused on identifying the highest number of programmes and other initiatives focused on supporting HCWs after PSI's. Therefore we included support interventions in health organisations in which HCWs are physically and/or emotionally affected by PSIs and other distressing situations. Based on the fact that there is still a lack of assistance to HCWs to cope in distressing situations, some support interventions are opening their scope of action (9).

We considered support interventions destinated to health professionals, residents, and other allied health professionals (such as technicians and supply workers) in a variety of healthcare settings, including urgent and acute care , primary care, ambulatory services, rehabilitation centres, long-term facilities, and outpatient surgery centres. The included support interventions were fully implemented and executed in health organisations and provided measurable results that assessed the attainment of a desired outcome.

Exclusion criteria

Editorials, letters to the editor, case series, case reports, narrative reviews and commentaries were excluded.

Stage 4: charting the data

A data extraction template was created to show the characteristics of the eligible studies (detailed information can be consulted in Supplementary Table 2).

Quality assessment

We used the Mixed Methods Appraisal Tool (MMAT) Version 2018 to conduct a quality assessment of the eligible studies (30). We believe that this appraisal will be important to enhance the quality and rigor of our study, ensuring greater transparency and validity of the data. The eligible studies were evaluated by two independent reviewers. A third reviewer was involved in cases of disagreement in the quality assessment.

Stage 5: collating, summarizing and reporting the results

The information from the eligible studies was collected and organised into different conceptual categories, as presented in the acronym OPERA (figure 1):

Organisational factors:

- *organisational structures* (e.g. infrastructures, resources, tools, equipment, units and staffing levels functional for managing and delivering services, leadership structure/authority and organisational culture);

- *organisational processes* (e.g. organisational actions, procedures, recruitment criteria, training, programme implementation, communication processes, quality of interactions and coordination during programme implementation and dissemination as well as the sustainability of the practice);

- *organisational outcomes* (e.g. implementation measures, process quality measures, utilisation measures, effectiveness measures that assess the attainment of an end state);

People - Relevant actors (individuals and organisations that actively participate in the development and implementation of the programme)

Environment - Contextual factors (type of healthcare setting and cultural context).

Recommendations described in the included studies (recommendations to improve the implementation process of the support interventions).

Operational attributes of the interventions (format/type of programme, accessibility, usability and confidentiality of the programme/intervention).

Stage 6: consultation exercise and stakeholder involvement

We invited a group of five experts working on SVP research from five different countries (Finland, Germany, Italy, Portugal and Spain) to complement the literature search with additional sources of information. All of them are members of the European Network Working on Second Victims.

Patient and public involvement and engagement

None

RESULTS:

A total of 9,708 records were retrieved from nine electronic databases, 43 articles were retrieved from the reference lists of the included articles, 11 from websites and 4 were collected from stakeholders' group inputs.

Based on the screening of titles and abstracts, 7,262 articles were excluded and 13 articles could not be retrieved after trying to contact the authors. A total of 156 articles were assessed for eligibility.

A third independent author was involved to solve 4 conflicts in authors' decision, leading to the inclusion of one article . In total, 127 articles were excluded after the screening, and 29 articles ultimately met the eligibility criteria. Detailed information about the data collection, screening process, duplicates removed and reasons for exclusion is exhibited in the flow chart (Supplementary Figure 1), in line with the original PRISMA statement (31)

Studies with levels of evidence IV and II (32) met the eligibility criteria. We have included the following types of studies: mixed methods (n = 15); quantitative descriptive (n = 8); qualitative (n = 3) ; randomised controlled trial (n = 2) (for further details about the included studies consult Supplementary Table 2). Bearing in mind that we only included empirical studies, we didn't include the first two screening questions in the MMAT evaluation (optional for MMAT) (30).

The characteristics of the included studies are outlined in Table 1.

Table 1- Characteristics of the included studies

**Categories**	**Subcategories**	**Number of articles**	**Total**
**Type of**	Level VI - Evidence from a single descriptive or qualitative study	26	29

For peer review only - http://bmjopen.bmj.com/site/about/guidelines.xhtml

**scientific article** [1]	Level II - Evidence obtained from Randomised Controlled Trial	3	
**Type of Study design**	Mixed Method	15	29
	Quantitative descriptive	8	
	Qualitative	3	
	Randomised Controlled Trial	3	
**Country where the study was developed**	Denmark	1	29
	Germany	3	
	New Zealand	1	
	Spain	2	
	Sweden	1	
	UK	2	
	United States	19	

1.This rating scale is based on Ackley, B. J., Swan, B. A., Ladwig, G., & Tucker, S. (2008). *Evidence-based nursing care guidelines: Medical-surgical interventions.* (p. 7). St. Louis, MO: Mosby Elsevier (32).

Most of the programmes included in this study have a multidisciplinary application and were focused on supporting HCWs after traumatic work experiences directly associated with PSIs. Several programmes were particularly focused on responding to severe adverse events (33–35).

Although one-to-one sessions were the most commonly provided support, some programmes also included group sessions. In our study we also included interventions focused on raising awareness of SVP and creating a supportive and proactive culture to manage critical incidents and enhance HCWs' well-being.

The included interventions are described in Supplementary Table 3.

In the following section we present the results based on the organisation of the acronym OPERA : Organisational factors (structure, process and outcome), People, Environment, Recommendations from the included studies and Attributes of the support programmes.

(Organisational factors)PERA

a) Organisational factors - Structure

Resources:

We found four main types of useful resources used in the interventions according to different applications. These resources are described in detailed in Table 2.

Table 2: Resources identified in the included HCW support programmes

**Marketing and dissemination materials**	Print marketing materials: posters (36,37); handouts such as brochures and flyers (38–40) identification badge of peer support for easy recognition and quick reference cards (38) .<br><br>Digital marketing material: promotional videos (41) website (39,41,42) ; emailbox (42–44).
**Selfcare and wellbeing related resources**	Packets with aromatherapy (45,46); chocolate (35,46,47); snacks (48,49); kind messages (35,46,48); self-care pockets with essential support resources and guidance for coping with normal grief responses (50), others contain a journal, a stress ball, and tissues (47); general mental and emotional wellness advices (51), and use existing resources (51).
**Functional resources for programme implementation**	Electronic mailbox (38,39,42–44,46,52–55) access to virtual zoom (42,43) and WebEx platform (42); dedicated mobile phone/pager/hotline/phone call system for peer supporter sessions (35,36,44,55–58); web-based collaborative administrative platform for sharing information and managing the program (39) such as Sharepoint (38,52); checklist of responsibilities for the development team (50); list of peer support schedules (40,50); peer encounter forms (44,52) secure database of outreach attempts (55).

**Educational resources (most of them are related with peer support training)**	Online training focused on psychological first aid (33,44) PowerPoint presentations with voice narrations (33) training scenarios (47,57,59); videos (33,39,60); "Do's" and "Don'ts" list, self-affirmations resources (55,60) and specific facilitator's guide (42) made available to peer supporters for guidance during the encounters with SV/HCWs; tutorial for peer support facilitation (49).

Posters, brochures and flyers were the most widely used marketing resources. The most recent studies have an investment in administrative support resources such as the use of SharePoint, a collaborative platform for programme management (38,39,52).

Infrastructures:

The acquisition of materials and human resources was, in most of the cases, on a voluntary basis. However, some studies mentioned that the intervention received specific funding for acquiring resources (45,46).

The existence of a specific room for sharing information and emotions in privacy was referred to by two studies (35,50).

Organisational culture

We identified the following factors associated with the organisational culture that are facilitators of the implementation of HCW support programmes after PSIs:

- openness of the health organisation to innovation (40);
- implementation of previous initiatives that have contributed to the creation of a proactive organisational culture to manage PSIs (36) and to support HCWs after PSIs /other stressful situations and promote their well-being (43,45,46,53);
- the existence of formalised structures directed at fostering a PS culture, based on a just culture approach (35,46,53), and at supporting HCWs and enhancing their well-being (35,52,53,59);
- active involvement of leadership members in initiatives that support SVs and HCWs' well-being (35,39,40,42,44,45,53,55);
- existence of established policies promoting a supportive organisational culture (such as the application of paid time off after a critical incident occurs) (35) and organisational accountability for employees' support and well-being after PSIs (51).

We also identified some potential organisational barriers to the implementation of programmes:

- a lack of staff and leadership awareness regarding the support programmes for HCWs (41);
- the organisational culture doesn't prioritise PS and HCW support and doesn't disclose wellness problems (48,54,58).

b) Organisational factors – Process

We found that most of the implemented programmes had developed a needs assessment (39,44,49,51,52,58) and/or conducted a literature review (33,39,55) prior to the design and creation of the programme. The needs assessment makes it possible to adapt the interventions to the needs of the clinical teams and adjust them to the institutional context and culture in accordance with the most recent literature.
The team was recruited using three different methods: direct nomination of the team members based on their ability to provide support in an empathic way (36,44,45,53,55); votes from the clinical team (46,55); and on a voluntary basis (39,44,58).
An advertising campaign for raising attention of all the staff that would benefit the programme and show how to activate the service was carried out in a large number of interventions (36,38–42,51).

We describe below some of the implemented communication strategies described in the included studies:

- Digital marketing: dissemination of the programme on computer screensavers (41) and digital communication through the institutional website (40–42).

- Internal communication: hospital magazine, newsletters or email (41,43,44,44).

- Networking: presentation of programme in divisional meetings (39,41,44,50,53,54) or in hospital-wide events and conferences (47,50).

For peer review only - http://bmjopen.bmj.com/site/about/guidelines.xhtml

- Involvement of the leadership members in the dissemination process (41,48) and some programmes have included unit-level champions (41).

- Previous staff training on the topic of SVP (39,41) including training for staff provides the first level of support after a PSI in the local (57).

Dissemination was also carried out for recruiting peer supporters to join the peer support programmes (39,52,53,55). Most of the peer supporters received specific training to prepare them for providing assistance to others (36,38–41,44–46,48,49,52–55,57,61).

Sustainability of the programmes:

After implementing the pilot intervention, several projects have effectively expanded the pilot intervention to other departments (52), other healthcare facilities (37,39) or hospital wide (38,51). The full integration of the programme in the departments underlies the inclusion of the programme in the scheduled activities and in the available services of the institution (57).

Leadership support was an important factor for the implementation of the programme and its sustainability (36,41,44). Other programmes nominated unit champions to ensure the implementation of the programme and its sustainability by promoting a more supportive culture within the unit (38,54,58).

Regular meetings were found to be important to maintain the cohesion of the team over time (36,45,47,57). Annual courses and the implementation of an interactive virtual platform were important for the expansion of the workforce working in these programmes (36,39). The high level of motivation and interest of the team (55) and retention of peer supporters were given particular consideration for programme sustainability and this was associated with work meaningfulness, staff satisfaction, commitment, a high level of resilience and a high level of confidence as a peer supporter (41).

Some of the programmes were implemented in healthcare organisations where some PS initiatives and "culture-shifting interventions" had already taken place (38,40,41,45). In other cases they were integrated in major projects developed by the organisations (47,53,56). Both situations were considered potential facilitators for maintaining the programmes over time.

Funding was also an important aspect to consider for the sustainability of several programmes (37,42,45,53,54).

c) Organisational factors - Outcomes

Most of the studies included in the analysis focused on collecting outcomes related with programme's utilisation and the evaluation conducted by both peer supporters and users (HCWs/second victims that attended to the programmes).

In the Table 3 we describe in further detail the outcomes evaluated in the included studies.

Table 3: Collected outcomes from the included interventions

**Outcomes related to support services utilisation**	Frequency of the HCWs who attended the programme (36,46,53,56,60); frequency of programme activation (34,36,38,39,44,45,47,48,51–55,57) , average duration of the encounters (57) number of programme dropouts (33); median number of interactions per month (54,57,58); frequency of peer support encounters (41,58); number of HCWs that need external support (57).
**Evaluation of the programme by the peer supporters perspective**	Overall peer support satisfaction with the training (33,34,44,51,60); perception of acquired knowledge, meaningfulness, motivation and interest to learn more and apply the learning (59); satisfaction about how encounter end out (39), need for additional training and experience (39), feeling able to provide support and being comfortable with their knowledge and skills as a peer supporter (39).
**Evaluation of the programme by the user perspective (HCWs involved in PSIs/SV)**	Overall satisfaction with the programme (33,36,37,39,45,48–52,60–62) knowledge/skills acquisition (33,43), usefulness of the contents (33), timeliness of the program (55) perceived helpfulness of the program (42,58); HCWs awareness of SVP phenomenon (40). qualitative experience after attending the programme (how much HCWs benefit from the programme) (33,36,37,39,45,48–52,60–62)

**Health-related outcomes**	Psychological and physical distress (44); emotional distress (42); perceived stress (56,59) anxiety and burnout (49,61) assessment of quality of life (46), perceptions of individual coping skills such us emotion regulation, self-efficacy and resilience (40,44,59,60).
**Work-related outcomes**	Job satisfaction (46) turnover intention and absenteeism (44) return to work (35) confidence in coping with adverse events (60).

O(People)ERA- Relevant actors

The establishment of a multidisciplinary team for the development and implementation of the support programmes was common to all the programmes. This team was predominantly composed by leadership members (hospital administrators and unit leaders), front-line workers, academics and experts in quality and safety (33,34,41,50,52,54). In some cases, it also included chaplains (50), social workers (50,52) and legal department members (54).

Most of the programmes' development and implementation were dependent on volunteer efforts. However, some programmes hired specific elements of the team, such as the programme directors and coordination members (40,53,56,57). Several studies highlighted the importance of these members in the programme activation process, particularly in matching the profile of peer supporters with HCWs' needs and in the contact with peer supporters and outreach people in need of support (47,53–55,58). One programme included contract freelancer work by psychotherapists to provide more specialised support (56) and other programmes remunerated peer supporters for their work (45,51,56).

Trained peer supporters are crucial for providing effective support for HCWs involved in PSIs. Specialised trainers from different types of backgrounds (such as psychology, nursing, quality improvement and PS, workplace wellness, legal services, executive sponsors, department representatives) provided workshops and seminars for peer supporters (46,52,55). In some cases, there was a specialist to facilitate monthly debriefing meetings for peer supporters to process their experiences and to receive assistance (45,46,52).

Most of the programmes also provided access to specialised external support that represents the third level of support in the case of programmes that follow the Scott Three-Tier Model (34,39,46,48,57). In other types of programmes, complementary support was provided by chaplains, social workers or Employee Assistance Programme (EAP) counsellors (35,39,50,52).

On a department level, unit leaders performed different types of essential functions by contributing to the development of the programme (45,55), participating in the recruitment of peer supporters (33,41,48,52), providing first-level support for HCWs in need (34), coordinating programme's components and mentoring peer support team members within the facility (57).

OP(Environment)RA – contextual factors

On an internal level, we found that most of the programmes were implemented in large and academic hospitals, characterised by an environment with multiple and complex divisions (34–36,38–40,42,44,53,54,57), and with a high level of specialisation (tertiary and quaternary care) (43,45,48,51,55). Some programmes were specifically implemented in stressful and busy environments, such as emergency medicine departments, intensive care units and psychiatric departments (38,40,48,49,59).

We found that in some cases the organisational environment was beneficial to the implementation of the programme, particularly when healthcare organisations were already working towards creating a more supportive environment for their staff and strengthening the safety culture (45–47,52,53,56).

We also found that previous occurrence of a very serious adverse event helped in recognizing the need to implement a programme to support staff in supporting them to cope after PSIs (35,41).

On an external level, several studies have mentioned that programme implementation was affected by the Covid-19 pandemic, such as the possibility of handling face-to-face encounters, and it also affected the data collection/monitoring process of the interventions (6,8,9,11,22,24,27).

OPE(Recommendations)A - Recommendations related with the implementation process and directly related with HCWs experience.

Different types of recommendations for improving the programmes were mentioned in the included studies with the ultimate goal of achieving a more effective intervention. They were identified from the user perspective (HCWs in need of support after being involved in a PSI) and also from the perspective of the implementation process (described in Table 4).

Table 4: Main recommendations referred to in the included studies from both the user and the implementation process perspectives

Recommendations related with the implementation process	Recommendations directly related with users' experience (Healthcare workers involved in PSIs/SV)
**Conditions to facilitate implementation process:**	**Conditions to facilitate users experience:**
To allocate protected time for teams to implement the programme and actively participate in the tasks and training (34,39);	To allocate protected time for HCWs to attend the programme's activities and the support sessions (37,49,60);
Administrative framework should be ensured to support programme implementation (57);	Participation in the process should be entirely voluntary and confidential (34,36,39,49,54,57,58,60);
To develop an institutional policy to guide the management of the critical event and support the affected HCWs and patients (35);	To invest in creating an organisational culture that addresses and acknowledges clinicians' vulnerability, while promoting a supportive environment after stressful incidents (53,57);
Funding was an important facilitator for program development and implementation (38,42,45);	To involve legal and risk management departments to address concerns about confidentiality or related issues (34,35,54);
To be formally recognised as an institutional program (34);	To ensure that SV have an adequate access to the programme, feel safe and not stigmatised when accessing resources (48);
To invest in telehealth solutions to support HCWs in the workplace (42);	**Procedures focused on user's experience:**
**Procedures related with the implementation process:**	Appropriate timing for programme's activation (ideally it should be immediately available to the HCWs after a stressful event (57)) and adequate duration of the support (36,60);
To invest in programme's dissemination and marketing for increasing HCWs' adherence to the programme (34,38,42,44,48,51,55–57);	All HCWs involved in a critical incident should be contacted to receive support (36,54);
To actively involve the target group in the development of the programme and to conduct a needs assessment helps fostering interest and adapt to the specific needs of the target population (49,61);	Active surveillance in the units should be done to identify potential SV (particularly in high risk environments) (57);
To integrate staff working in the unit in the programme's team, helps to understand the needs of the unit (45).	To increase program awareness for front-line staff and prepare them how to give first level of support (44,48,52);
To promote active involvement of leadership members facilitates the implementation of the programmes and contributes for staff engagement (33,35,36,39,47–49,51,53,57);	To train the leadership to support staff to cope with stressful situations and to direct them to support resources in case of need (34,45–47,60);
To promote training sessions and resources to increase managers awareness about the SVP and about the existing support programmes (47,48);	To enhance the level of education on staff resiliency in the services (40);
To create a multidisciplinary support team to facilitate a comprehensive programme's development and address different areas for support while leveraging a range of expertise (54);	To establish an interdisciplinary support team to open the scope of support according with the different staff's needs and background (38,41,52);
To set regular debriefings (in person or virtual meetings) to exchange experiences and to foster a culture of mutual support among the members of the programme's team (36,47);	To create a safe place for sessions (45,46,49,50);
Training should be provided to peer support training and role play is one of the most recommended formats (34,41,47);	Smaller groups are preferred for sharing experiences and support (37);
To develop a list of key phrases that peer supporters can use in their interactions with SVs (41);	To make resources available and close to the staff, to make them easy to reach (48,50,53);
To evaluate the impact of the programme and monitor its longer term effects and drive continuous improvement (41,42,44,45,47,51,52,59).	To identify barriers and facilitators for HCW to seek mental health support (e.g. stigma, career concerns, protected time) (42);
To use pre-existing structures , resources and adapt existing programmes to facilitate the development and implementation of the programme(38,39,47,51);	To provide channels to reach the programme even when the HCW was not involved in a PSI (55);
Having an electronic dashboard for sharing documentation and data collection (38);	To provide a holistic support (e.g. incorporating integrative therapy techniques; cognitive based therapy) (45,59).

OPER(Attributes) - Operational attributes of the programmes

For peer review only - http://bmjopen.bmj.com/site/about/guidelines.xhtml

We considered the main operational attributes of the programmes: accessibility and usability.

a) Accessibility:

The access of HCWs to the programmes was done by different channels to find the most convenient format for the users: phone call (36,38,41,52,56), email (38,43,52,59) or direct contact with peer supporters or with core team members (38,52,59).

Programmes can be activated by the following people:

- anyone that was involved in the stressful event (34,44,47,51,53,57);
- safety and risk management staff (47,54);
- peer supporters (54);
- nurse in charge (50);
- leadership members (34,35);
- programme directors (53–55).

In some cases, the entire clinical team is contacted by the implementation team or by leadership members with a view to integrating in the support programme after a PSI, however acceptance only depends on the individual choice of the HCWs (34,55,58,61).

In the case of programmes that have online resources, they could be accessed through a website (33,39,40).

Although most of the programmes were provided on a voluntary basis, some of them have mandatory activities for all staff in the departments, such as a seminar to promote a shared understanding of SVP and the need for peer support (51), or attendance at debriefing sessions to enhance the recovery of all staff in the unit (35).

Programmes based on the Scott Three-Tier Model establish the access to the programme according to different levels of HCW needs (34,36,57). The first level of support should be available immediately after the incident has happened and team members should be prepared to provide it (local-level support). For accessing the second level of support, anyone can activate peer support with a trained peer. For the third level of support, the HCW is referred to specialised support (57).

In some cases, programmes can be accessed seven days a week (38,40,42,44,52,56,57). In a support programme implemented in New Zealand, the phone number to reach the support team was added into the staff contact list and on-call phones within the unit to facilitate the activation process (36). The dissemination of schedules, timelines and contacts was a useful strategy employed to facilitate access to the programme (40,44).

The identified barriers to accessing the programmes included the lack of staff awareness about the programme (41,48) and getting the time to attend the interventions (37). Moreover, some HCWs resisted accessing support since they did not recognise the need for it or preferred to avoid dealing with the situation again by talking about it (35).

b) Usability :

To ensure the maximal attendance of staff, in several programmes the communication process was facilitated by peer supporters or the programme director after the activation of the programme. These actors have the responsibility of contacting the HCWs immediately after the incident to schedule a one-to-one peer support encounter (39,51,53–55,58). In several programmes, the schedule was negotiated with HCWs according to their needs (36,39,45,47,54,55,58), and in some cases the location (39,45) and format (in person, phone call or email) of the sessions were also negotiated (47,55).

In some cases, staffing relief at the workplace and protected time were provided to allow HCWs to attend the support programmes during their working time (39,43,45,49,50,61).

To facilitate initiation of the programme, a leadership member or the programme director matched the peer supporter profile with the HCWs' needs according to the different criteria, such as geographic proximity and the same medical speciality (39,47,55). In other situations, like in the Buddy Study Programme, the HCWs selected the peer supporter according to their preferences so they could contact if they needed support (51).

To facilitate the HCWs' participation in the programme, some programmes prioritised the virtual format (39,42,43,54) and limited the duration of sessions to 60 minutes (36,37,42,45,49).

A peer support intervention in Sweden defined the topics to approach in the sessions based on participants' suggestions and beliefs (61). This made it possible to attend to the needs of the participants and to promote their adherence to the interventions (61).

In online programmes like MISE we found that browsing, amount of time required to complete the activities and comprehension of the programme content were valued attributes to facilitate the usability of the programme (33).

DISCUSSION

The majority of the programmes included in this study are based on peer support. These types of programmes are founded on social support with the ultimate goals of increasing professional quality of life, decreasing emotional stress and ensuring patients' safety (63–65).

We found that these types of programmes should be voluntary, with easy access and widely disseminated in healthcare organisations. This will make it possible to provide immediate psychological first aid after a distressful event and to overcome obstacles related to a lack of awareness of SVP and stigmatisation associated with HCW vulnerability (38,41,65–67).

The association between just culture and empathic and non-judgemental responses to PSIs has been clear (18,41,68,69). This is identified as a core condition for an open communication, and to establish positive relationships between peer supporters and HCWs, thereby enhancing programme adherence (69,70). Namely, willingness to give support with empathy, leadership skills, reliability, being communicative, and not being judgmental are essential elements for effective peer support (64). Another study pointed out that the ability to understand others' feelings and experiences after a PSI can be beneficial to the support process, by improving emotional regulation and reinforcing the cooperation between HCWs and peer supporters (71). In our study we have identified that all these principles were mentioned to improve the effectiveness of the interventions and therefore it should be taken in consideration in peer supporters training when implementing the programme.

We found that specific training for peer supporters was provided in most of the programmes to prepare them to adequately provide psychological support according to HCWs' needs. Training is believed to be one of the key components to consider when implementing a support programme (38,41,65). According to implementation research, it's essential for an effective programme implementation (72).

Many of the included studies also mentioned the importance of setting regular multidisciplinary meetings to share important learning and experiences and to keep the team motivated over time. Rosak-Szyrocka (2015) points out that having a motivated team is very important to ensure their commitment to, and engagement in work (73). This study also points out that in hospitals, HCWs are particularly motivated by strong interpersonal relations and a positive atmosphere, as they foster cooperation and mutual support among the team members (74). Another study indicates that multidisciplinary teamwork is an essential element for improving outcomes at an organisational level (75).

We found that active participation by leaders in the initiatives can influence the effectiveness of programmes implementation and their sustainability. Helping to create a safe and resilient

For peer review only - http://bmjopen.bmj.com/site/about/guidelines.xhtml

environment can increase the programmes' acceptability among HCWs and their engagement in the activities (75–78). Leaders' participation was not only important in the implementation process, but also in the development of the programmes. In particular, it can contribute to adjust the programmes to the healthcare context, facilitate the acquisition of resources and recruitment of peer supporters.

We also found that leadership engagement in HCWs/SV support initiatives is very important for strengthening the organisational culture towards a non-punitive response to error. Leadership members are essential actors in helping to create stimulating and supportive environments in healthcare among teams (74,79). Boguslavsky, Gutierrez- and Holschneider (76), refers the importance of having leaders with empathic and communication skills, that are able to listen, empower and encourage others, in line with non-blame culture principles. Therefore, the involvement of leaders with these types of skills will benefit the support programme's implementation.

Moreover, we also found that the success of the implementation is also dependent on how closely aligned they are with HCWs' needs. Therefore, HCWs should be consulted and involved in the programme development process. Our results corroborate Søvold et al.'s findings, which highlight the importance of HCWs participating more in the decision-making process as well as in the development, implementation, testing and evaluation of the interventions, with the ultimate aim of improving their health, well-being and job satisfaction (80). Interventions that include the target population's perspectives from the first steps of development exhibit a higher level of adherence and adoption and are more sustainable over time (72).

However, HCWs are very often overloaded with duties that could be undertaken by other staff, and prevented from performing other necessary tasks that need their qualifications, with few opportunities to apply for training, develop their professionals skills and be available to participate in workplace initiatives (75). This contributes to less work satisfaction and more costs for the system (75). We found that working conditions are one of the main pillars for ensuring effective implementation and its sustainability. Providing protected time and staffing relief were identified as two of the main priorities for ensuring that, on the one hand, qualified HCWs could participate in the development and implementation of support interventions, and on the other, HCWs would be able to attend the support programmes if they needed support. Financial incentives play also an important role in keeping the support teams over time and to facilitate the programme implementation. Some evidence corroborates this findings namely in what concerns to HCWs retention (72,75).

Finally, it is agreed that monitoring programme outcomes over time is essential for assessing programme effectiveness, and evaluating its progress and impact on HCWs/SVs and in health organisations (81). This process should be continuous and facilitates the ongoing improvement of the programmes (22). However, there are a limited number of studies that follow up interventions over time. According to Wade et al. (2022), it's still not clear how much time would be necessary to monitor the impact of programmes on HCWs' skills and knowledge (82). Thus, we recommend that future programmes invest in monitoring their results over time and for longer periods.

In hospital settings, we have found that programmes frequently monitor before and after interventions, by collecting both qualitative and quantitative data. It's agreed that monitoring process should follow rigorous and feasible when assessing both types of measures (81).

Although hospital settings are increasingly investing in the monitoring process, the study of the impact of HCWs' support programmes it's still limited and unreported in non-hospital settings (83). This might be due to the insufficient emphasis on safety culture in non-hospital settings (83,84), which could be improved by increasing PS initiatives and awareness campaigns in these contexts.

The experience from support programmes directly and not directly associated with supporting HCWs after PSIs was particularly useful to identify barriers and challenges in the access and adherence to the programmes and sustainability concerns. One of the main topics of discussion is focused on programmes' confidentiality (41,85–87). Wade et al. (2022) highlighted the need to establish a consensus and build a body of evidence to evaluate these types of programmes in an ethical and confidential way that involves protecting the privacy and confidentiality of the attendees of these types of programmes (82). We also highlight that the legal framework of each country focused on

professional liability is often not cited in articles describing interventions, despite its influence on programmes and its impact on transitioning from a reactive safety culture to a generative safety culture that can influence the success of this type of programmes implementation (88).

Ultimately, we found that these types of programmes should be formalised and have defined structures in order to facilitate its sustainability and to overcome potential institutional barriers to the implementation of the programmes. Examples like RISE programme have demonstrated that the formal recognition of the support programme in all the large academic medical centre , the use of existing structures and involvement institutional stakeholders have not only strengthen its visibility within the institution, but also inspired other external health settings to implement their own support programmes (41).

In summary, we have organised the main findings in Supplementary Table 4, according with the acronym OPERA.

LIMITATIONS

In this study, we have found that long-term evaluation of programmes it’s still limited for the most part, and in some cases, evaluating the outcomes is not recognised as a priority for reasons of confidentiality and ethics. We recognise the need to strengthen the organisational culture towards a non-punitive response to error in order to overcome potential barriers to programme adherence and evaluation. It is very important to increase the follow-up time to understand the impact of these types of programmes and their effectiveness in the long term.

We evaluate all the included studies using the MMAT quality assessment tool since this tool is suitable for different types of methodologies, however some of the criteria couldn’t be applied in the descriptive studies. We suggest that this tool could be adapted in the future to these types of studies.

CONCLUSION

This is the first time that a study has focused on understanding the set of characteristics and elements necessary for a successful programme' implementation to respond to HCWs needs after PSIs, based on the fact that their success highly depends on an adequate implementation and evaluation process.

This study was inspired in the previous experience from other support programmes with the ultimate propose of guiding the implementation of HCWs support programmes in health organisations and contributing for future evidence-based practice.

In summary, we concluded that programmes should be easily accessible and voluntary for all HCWs in health organisations. Dissemination should be prioritised in order to give higher level of visibility to these programmes. The effectiveness of programme’ implementation is highly dependent on the organisational culture, the active involvement of leadership, and a multidisciplinary team. Training should be provided for both peer supporters and leadership members, to make it possible to respond to HCWs’ needs in a more prepared and satisfactory way. Regular communication among support teams should be maintained over time to keep teams motivated and increase their retention. Moreover, it's recommended to allocate dedicated time and staffing resources to engage in these types of interventions. Establishing formalised structures and securing funding sources it’s important for the programmes's sustainability. The use of existing resources can overcome potential institutional barriers. Finally, programmes should be monitored for their continuous improvement without compromising the confidentially of the data.

AKNOWLEGMENTS

This work was supported by ERNST – Cost Action 19113 (COST – European Cooperation in Science and Technology, www.cost.eu). We would like to particularly thank the members of stakeholder groups,

namely Prof. Reinhard Strametz, Dr Susanna Tella and Prof. Massimiliano Panella, for their contribution to this study as members of the consultation group.

FUNDING

The present publication was funded by Fundação Ciência e Tecnologia, IP national support through CHRC (UIDP/04923/2020) and under the grant (UI/BD/150875/2021).

ETHICS AND DISSEMINATION

This scoping review does not require ethical approval. Results and conclusions of the study will be presented in scientific conferences as well as in international forums and through other relevant dissemination channels.

AUTHOR CONTRIBUTIONS

SGP was involved in the writing of the article. SGP, MJL, PS and JJM contributed to the conceptualisation of the study. SGP, MJL, DGS participated in the pilot testing stage and defined the inclusion criteria. SGP, MJL, JDS, JF and IC were involved in the screening process of the eligible studies. HD contributed to the definition of a robust search strategy and participated in the preliminary search in relevant bibliographic databases, particularly in peer-reviewed journals and grey literature databases. PS, JJM and IC contributed to the literature review. PS and JJM also participated in the consultation group. All authors read and approved the manuscript.

COMPETING INTERESTS

This protocol is part of the PhD project of the corresponding author (SGP), which has been previously approved by the National School of Public Health (ENSP- NOVA). Some of the research group members are part of the European Researchers Network Working on Second Victims (ERNST) - COST Action 19113. All the authors have submitted the disclosure of interest form.

REFERENCES

1. Seys D, Wu AW, Gerven EV, Vleugels A, Euwema M, Panella M, et al. Health Care Professionals as Second Victims after Adverse Events: A Systematic Review. Eval Health Prof. 2013;36(2):135–62.
2. Waterman AD, Garbutt J, Hazel E, Dunagan WC, Levinson W, Fraser VJ, et al. The Emotional Impact of Medical Errors on Practicing Physicians in the United States and Canada. Jt Comm J Qual Patient Saf. 2007;33(8):467–76.
3. Vanhaecht K, Seys D, Russotto S, Strametz R, Mira J, Sigurgeirsdóttir S, et al. An Evidence and Consensus-Based Definition of Second Victim: A Strategic Topic in Healthcare Quality, Patient Safety, Person-Centeredness and Human Resource Management. Int J Environ Res Public Health. 2022;19:16869.
4. World Health Organization. Patient safety incident reporting and learning systems: technical report and guidance. Geneva: World Health Organization; 2020.
5. NHS England. Report a patient safety incident [Internet]. Redditch; 2022. Available from: https://www.england.nhs.uk/patient-safety/report-patient-safety-incident/
6. Rinaldi C, Leigheb F, Vanhaecht K, Donnarumma C, Panella M. Becoming a “second victim” in health care: Pathway of recovery after adverse event. Rev Calid Asist. 2016;31:11–9.
7. Vanhaecht K, Seys D, Schouten L, Bruyneel L, Coeckelberghs E, Panella M, et al. Duration of second victim symptoms in the aftermath of a patient safety incident and

For peer review only - http://bmjopen.bmj.com/site/about/guidelines.xhtml

association with the level of patient harm: a cross-sectional study in the Netherlands. BMJ Open. 2019;9:e029923.

8. Strid EN, Wåhlin C, Ros A, Kvarnström S. Health care workers' experiences of workplace incidents that posed a risk of patient and worker injury: a critical incident technique analysis. BMC Health Serv Res. 2021;21(1):511.

9. Busch IM, Moretti F, Purgato M, Barbui C, Wu AW, Rimondini M. Psychological and Psychosomatic Symptoms of Second Victims of Adverse Events: a Systematic Review and Meta-Analysis. J Patient Saf. 2020;16(2):e61–74.

10. Carrillo I, Tella S, Strametz R, Vanhaecht K, Panella M, Guerra-Paiva S, et al. Studies on the second victim phenomenon and other related topics in the pan-European environment: the experiences of ERNST Consortium members. Journal of Patient Safety and Risk Management. 2022;27(2):59–65.

11. Burlison JD, Quillivan RR, Scott SD, Johnson S, Hoffman JM. The Effects of the Second Victim Phenomenon on Work-Related Outcomes: Connecting Self-Reported Caregiver Distress to Turnover Intentions and Absenteeism. J Patient Saf. 2021;17(3):195–9.

12. Scott SD, McCoig MM. Care at the point of impact: Insights into the second-victim experience. Am Soc Healthc Risk Manag Am Hosp Assoc. 2016;35(4):6–13.

13. Quillivan RR, Burlison JD, Browne EK, Scott SD, Hoffman JM. Patient Safety Culture and the Second Victim Phenomenon: Connecting Culture to Staff Distress in Nurses. Jt Comm J Qual Patient Saf. 2016;42(8):377-AP2.

14. Hall LH, Johnson J, Watt I, Tsipa A, O'Connor DB. Healthcare Staff Wellbeing, Burnout, and Patient Safety: A Systematic Review. Harris F, editor. PLOS ONE. 2016;11(7):e0159015.

15. Schiess C, Schwappach D, Schwendimann R, Vanhaecht K, Burgstaller M, Senn B. A Transactional 'Second-Victim' Model-Experiences of Affected Healthcare Professionals in Acute-Somatic Inpatient Settings: A Qualitative Metasynthesis. J Patient Saf. 2021;17(8):e1001–18.

16. Schwappach D, Boluarte T. The emotional impact of medical error involvement on physicians: A call for leadership and organizational accountability. Swiss Med Wkly. 2008;138(1–2):9–15.

17. Moran D, Wu AW, Connors C, Chappidi MR, Sreedhara SK, Selter JH, et al. Cost-Benefit Analysis of a Support Program for Nursing Staff. J Patient Saf. 2020;16(4):e250–4.

18. Busch IM, Moretti F, Campagna I, Benoni R, Tardivo S, Wu AW, et al. Promoting the Psychological Well-Being of Healthcare Providers Facing the Burden of Adverse Events: A Systematic Review of Second Victim Support Resourcesmix. Int J Environ Res Public Healt. 2021;18(10):5080.

19. Pratt S, Kenny L, Scott SD, Wu AW. How to develop a second victim support program: a toolkit for health care organizations. Jt Comm J Qual Patient Saf. 2012;38(5):235–40.

20. Bauer MS, Kirchner J. Implementation science: What is it and why should I care? Psychiatry Res. 2020;283:112376.

21. Thoele K, Ferren M, Moffat L, Keen A, Newhouse R. Development and use of a toolkit to facilitate implementation of an evidence-based intervention: a descriptive case study. Implement Sci Commun. 2020;1(1):86.

22. Herrera-Sánchez IM, León-Pérez JM, León-Rubio JM. Steps to Ensure a Successful Implementation of Occupational Health and Safety Interventions at an Organizational Level. Front Psychol. 2017;8:2135.

23. Cook S, Mayers S, Goggins K, Schlundt D, Bonnet K, Williams N, et al.

For peer review only

For peer review only - http://bmjopen.bmj.com/site/about/guidelines.xhtml

Assessing research participant preferences for receiving study results. J Clin Transl Sci. 2019;4(3):243–9.
24. Buse K, Mays N, Walt G. The health policy framework:Context,process and actors. In: Making health policy. 1st edition. Maidenhead,Berkshire: McGraw- Hill Education:University Press; 2005.
25. Yano EM. The role of organizational research in implementing evidence-based practice: QUERI Series. Implement Sci. 2008;3(1):29.
26. Arksey H, O'Malley L. Scoping studies: towards a methodological framework. Int J Soc Res Methodol. 2005;8(1):19–32.
27. Tricco AC, Lillie E, Zarin W, O'Brien KK, Colquhoun H, Levac D, et al. PRISMA Extension for Scoping Reviews (PRISMA-ScR): Checklist and Explanation. Ann Intern Med. 2018;169(7):467–73.
28. The Joanna Briggs Institute. The Joanna Briggs Institute Reviewers' Manual 2015 Methodology for JBI Scoping Reviews. Australia: The Joanna Briggs Institute; 2015.
29. Guerra-Paiva S, Lobão MJ, Simões JD, Donato H, Carrillo I, Mira JJ, et al. Key factors for effective implementation of healthcare worker support interventions after patient safety incidents in health organisations: a protocol for a scoping review. BMJ Open. 2022;12(8):e061543.
30. Hong Q, Pluye P, Fàbregues S, Bartlett G, Boardman F, Cargo M, et al. Mixed Methods Appraisal Tool (MMAT), version 2018.Registration of Copyright (#1148552), Canadian Intellectual Property Office, Industry Canada.
31. Moher D, Liberati A, Tetzlaff J, Altman DG, for the PRISMA Group. Preferred reporting items for systematic reviews and meta-analyses: the PRISMA statement. BMJ. 2009;339:b2535.
32. Ackley B, Swan B, Ladwig G, Tucker S. Evidence-based nursing care guidelines: Medical-surgical interventions. St. Louis, MO: Mosby Elsevier; 2007.
33. Mira JJ, Carrillo I, Guilabert M, Lorenzo S, Pérez-Pérez P, Silvestre C, et al. The Second Victim Phenomenon After a Clinical Error: The Design and Evaluation of a Website to Reduce Caregivers' Emotional Responses After a Clinical Error. J Med Internet Res. 2017;19(6):e203.
34. Cobos-Vargas A, Pérez-Pérez P, Núñez-Núñez M, Casado-Fernández E, Bueno-Cavanillas A. Second Victim Support at the Core of Severe Adverse Event Investigation. Int J Environ Res Public Health. 2022;19(24):16850.
35. Roesler R, Ward D, Short M. Supporting Staff Recovery and Reintegration After a Critical Incident Resulting in Infant Death. Adv Neonatal Care. 2009;9(4):163–71.
36. Civil NM, Hoskins JD. Building a critical incident peer response team: A full theatre team welfare intervention. Anaesth Intensive Care. 2022;50(6):421–9.
37. Allen D, Spencer, G, McEwan K, Catarino F, Evans R, Crooks S, et al. The Schwartz Centre Rounds: Supporting mental health workers with the emotional impact of their work. Int J Ment Health Nurs. 2020;29:942–52.
38. Merandi J, Liao N, Lewe D, Morvay S, Stewart B, Catt C, et al. Deployment of a Second Victim Peer Support Program: A Replication Study. Pediatr Qual Saf. 2017;2(4):e031.
39. Finney RE, Jacob A, Johnson J, Messner H, Pulos B, Sviggum H. Implementation of a Second Victim Peer Support Program in a Large Anesthesia Department. AANA. 2021;89(3):235–44.
40. Bryant R. Raising Awareness of Second Victim Phenomenon and SupportingYOU: A Quality and Safety Initiative. Graduate Theses, Dissertations, and Problem Reports. West Virginia: West Verginia University; 2022. 10169.

For peer review only

41. Edrees H, Connors C, Paine L, Norvell M, Taylor H, Wu AW. Implementing the RISE second victim support programme at the Johns Hopkins Hospital: a case study. BMJ Open. 2016;6(9):e011708.

42. Mellins CA, Mayer LES, Glasofer DR, Devlin MJ, Albano AM, Nash SS, et al. Supporting the well-being of health care providers during the COVID-19 pandemic: The CopeColumbia response. Gen Hosp Psychiatry. 2020;67:62–9.

43. Rubin EW, Rassman A. cAIR: Implementation of peer response support for frontline health care workers facing the COVID-19 pandemic. Soc Work Health Care. 2021;60(2):177–86.

44. Thompson M, Hunnicutt R, Broadhead M, Vining B, Aroke E. Implementation of a Certified Registered Nurse Anesthetist Second Victim Peer Support Program. J Perianesth Nurs. 2022;37(2):167–73.

45. Schuster MA. Creating the Hematology/Oncology/Stem Cell Transplant Advancing Resiliency Team: A Nurse-Led Support Program for Hematology/Oncology/Stem Cell Transplant Staff. J Pediatr Oncol Nurs. 2021;38(5):331–41.

46. Graham P, Zerbi G, Norcross W, Montross-Thomas L, Lobbestael L, Davidson J. Testing of A Caregiver Support Team. EXPLORE. 2019 Jan;15(1):19–26.

47. Morales CL, Brown MM. Creating a Care for the Caregiver Program in a Ten-Hospital Health System. Crit Care Nurs Clin North Am. 2019;31(4):461–73.

48. Merandi J, Winning AM, Liao N, Rogers E, Lewe D, Gerhardt CA. Implementation of a second victim program in the neonatal intensive care unit: An interim analysis of employee satisfaction. J Patient Saf Risk Manag. 2018;23(6):231–8.

49. Calder-Sprackman S, Kumar T, Gerin-Lajoie C, Kilvert M, Sampsel K. Ice cream rounds: The adaptation, implementation, and evaluation of a peer-support wellness rounds in an emergency medicine resident training program. CJEM. 2018;20(5):777–80.

50. Foreman S. Developing a process to support perinatal nurses after a critical event. Nurs Womens Health. 2014;18(1):61–5.

51. Schrøder K, Bovil T, Jørgensen JS, Abrahamsen C. Evaluation of'the Buddy Study', a peer support program for second victims in healthcare: a survey in two Danish hospital departments. BMC Health Serv Res. 2022;22(1):566.

52. Krzan KD, Merandi J, Morvay S, Mirtallo J. Implementation of a "second victim" program in a pediatric hospital. Am J Health Syst Pharm. 2015;72(7):563–7.

53. Shapiro J, Galowitz P. Peer Support for Clinicians: A Programmatic Approach. Acad Med. 2016;91(9):1200–4.

54. Lane MA, Newman BM, Taylor MZ, O'Neill M, Ghetti C, Woltman RM, et al. Supporting Clinicians After Adverse Events: Development of a Clinician Peer Support Program. J Patient Saf. 2018;14(3):e56–60.

55. El Hechi MW, Bohnen JD, Westfal M, Han K, Cauley C, Wright C, et al. Design and Impact of a Novel Surgery-Specific Second Victim Peer Support Program. J Am Coll Surg. 2020;230(6):926–33.

56. Hinzmann D, Forster A, Koll-Krüsmann M, Schießl A, Schneider F, Sigl-Erkel T, et al. Calling for Help—Peer-Based Psychosocial Support for Medical Staff by Telephone—A Best Practice Example from Germany. Int J Environ Res Public Health. 2022;19(23):15453.

57. Scott SD, Hirschinger LE, Cox KR, McCoig M, Hahn-Cover K, Epperly KM, et al. Caring for Our Own: Deploying a Systemwide Second Victim Rapid Response Team. Jt Comm J Qual Patient Saf. 2010;36(5):233–40.

58. Rivera-Chiauzzi E, Smith H, Moore-Murray T, Lee C, Goffman D, Bernstein P,

For peer review only

For peer review only - http://bmjopen.bmj.com/site/about/guidelines.xhtml

et al. Healing Our Own: A Randomized Trial to Assess Benefits of Peer Support. J Patient Saf. 2022;18(1):e308–14.

59. Bernburg M, Groneberg DA, Mache S. Mental Health Promotion Intervention for Nurses Working in German Psychiatric Hospital Departments: A Pilot Study. Issues Ment Health Nurs. 2019;40(8):706–11.

60. Johnson J, Simms-Ellis R, Janes G, Mills T, Budworth L, Atkinson L, et al. Can we prepare healthcare professionals and students for involvement in stressful healthcare events? A mixed-methods evaluation of a resilience training intervention. BMC Health Serv Res. 2020;20(1094).

61. Peterson U, Bergström G, Samuelsson M, Åsberg M, Nygren Å. Reflecting peer-support groups in the prevention of stress and burnout: randomized controlled trial. J Adv Nurs. 2008;63(5):506–16.

62. Graham P, Zerbi G, Norcross W, Montross-Thomas L, Lobbestael L, Davidson J. Testing of A Caregiver Support Team. EXPLORE. 2019;15(1):19–26.

63. Winning AM, Merandi JM, Lewe D, Stepney LMC, Liao NN, Fortney CA, et al. The emotional impact of errors or adverse events on healthcare providers in the NICU: The protective role of coworker support. J Adv Nurs. 2018;74(1):172–80.

64. Carbone R, Ferrari S, Callegarin S, Casotti F, Turina L, Artioli G, et al. Peer support between healthcare workers in hospital and out-of-hospital settings: a scoping review. Acta Biomed Atenei Parm. 2022;93(5):e2022308.

65. Pratt SD, Jachna BR. Care of the clinician after an adverse event. Int J Obstet Anesth. 2015;24(1):54–63.

66. Dukhanin V, Edrees HH, Connors CA, Kang E, Norvell M, Wu AW. Case: A Second Victim Support Program in Pediatrics: Successes and Challenges to Implementation. J Pediatr Nurs. 2018;41:54–9.

67. Liukka M, Steven A, Vizcaya Moreno MF, Sara-aho AM, Khakurel J, Pearson P, et al. Action after Adverse Events in Healthcare: An Integrative Literature Review. Int J Environ Res Public Health. 2020;17(13):4717.

68. Arnetz JE, Zhdanova LS, Elsouhag D, Lichtenberg P, Luborsky MR, Arnetz BB. Organizational Climate Determinants of Resident Safety Culture in Nursing Homes. The Gerontologist. 2011;51(6):739–49.

69. Malik RF, Buljac-Samardžić M, Amajjar I, Hilders CGJM, Scheele F. Open organisational culture: what does it entail? Healthcare stakeholders reaching consensus by means of a Delphi technique. BMJ Open. 2021;11(9):e045515.

70. Aghaei H, Sadat Asadi Z, Mirzaei Aliabadi M, Ahmadinia H. The Relationships Among Occupational Safety Climate, Patient Safety Climate, and Safety Performance Based on Structural Equation Modeling. J Prev Med Pub Health. 2020;53(6):447–54.

71. Moudatsou M, Stavropoulou A, Philalithis A, Koukouli S. The Role of Empathy in Health and Social Care Professionals. Healthcare. 2020;8(1):26.

72. MEASURE Evaluation. Fundamentals of Implementation Research. U.S. Agency for International Development; 2012.

73. Rosak-Szyrocka J. Employee motivation in health care. Prod Eng Arch. 2015;6/1:21–5.

74. Kitsios F, Kamariotou M. Job satisfaction behind motivation: An empirical study in public health workers. Heliyon. 2021;7(4):e06857.

75. Dubois CA, Singh D. From staff-mix to skill-mix and beyond: towards a systemic approach to health workforce management. Hum Resour Health. 2009;7(1):87.

76. Boguslavsky V, Gutierrez R, Holschneider S. Effective Leadership for Quality Improvement in Health Care A Practical Guide. Published by USAID ASSIST Project.Chevy Chase. MD: University Research CO (LLC) URS; 2019.

For peer review only

For peer review only - http://bmjopen.bmj.com/site/about/guidelines.xhtml

77. Usman M, Ghani U, Cheng J, Farid T, Iqbal S. Does Participative Leadership Matters in Employees' Outcomes During COVID-19? Role of Leader Behavioral Integrity. Front Psychol. 2021;12:646442.
78. Fagerdal B, Lyng HB, Guise V, Anderson JE, Thornam PL, Wiig S. Exploring the role of leaders in enabling adaptive capacity in hospital teams – a multiple case study. BMC Health Serv Res. 2022;22(1):908.
79. Joint Commission. The essential role of leadership in developing a safety culture. Department of Corporate Communications; 2007.
80. Søvold LE, Naslund JA, Kousoulis AA, Saxena S, Qoronfleh MW, Grobler C, et al. Prioritizing the Mental Health and Well-Being of Healthcare Workers: An Urgent Global Public Health Priority. Front Public Health. 2021;9:679397.
81. Straus SE, Tetroe J, Graham ID, Zwarenstein M, Bhattacharyya O, Shepperd S. Monitoring use of knowledge and evaluating outcomes. Can Med Assoc J. 2010;182(2):E94–8.
82. Wade L, Williams N, Fitzpatrick E, Parker R, Hurley K. Organizational interventions and policies to support second victims in acute care settings: A scoping study. Can J Emerg Med. 2019;21:S112–3.
83. Mira JJ, Lorenzo S, Carrillo I, Ferrús L, Pérez-Pérez P, Iglesias F, et al. Interventions in health organisations to reduce the impact of adverse events in second and third victims. BMC Health Serv Res. 2015;15:341.
84. Lawati MHAl, Dennis S, Short SD, Abdulhadi NN. Patient safety and safety culture in primary health care: a systematic review. BMC Fam Pract. 2018;19(1):104.
85. Simms L, Ottman KE, Griffith JL, Knight MG, Norris L, Karakcheyeva V, et al. Psychosocial Peer Support to Address Mental Health and Burnout of Health Care Workers Affected by COVID-19: A Qualitative Evaluation. Int J Environ Res Public Health. 2023;20(5):4536.
86. Marr R, Goyal A, Quinn M, Chopra V. Support opportunities for second victims lessons learned: a qualitative study of the top 20 US News and World Report Honor Roll Hospitals. BMC Health Serv Res. 2021;21(1):1330.
87. Quadrado E, Tronchin D, Maia F. Strategies to support health professionals in the condition of second victim: scoping review. Rev Esc Enferm USP. 2021;55:e03669.
88. Mira JJ, Lorenzo S, Carrillo I, Ferrús L, Silvestre C, Astier P, et al. Lessons learned for reducing the negative impact of adverse events on patients, health professionals and healthcare organizations. Int J Qual Health Care. 2017;29(4):450–60.

For peer review only

Legend of the figures-

Figure 1 – OPERA- The five key domains to guide HCWs' support interventions after stressful events such as PSI

Supplementary Figure 1 – PRISMA flow chart of the selection process

For peer review only - http://bmjopen.bmj.com/site/about/guidelines.xhtml

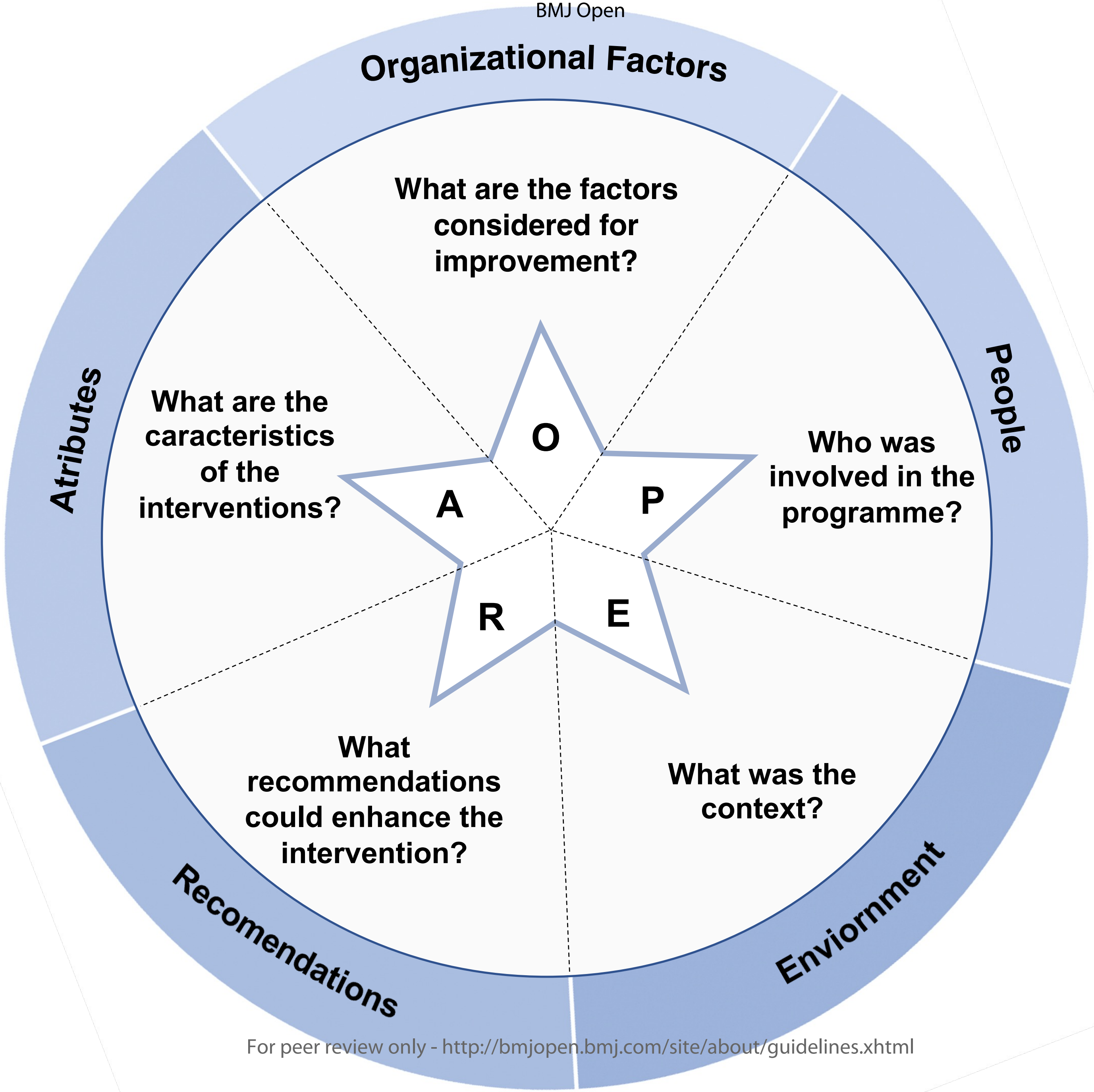
Organizational Factors
What are the factors considered for improvement?
People
Who was involved in the programme?
Enviornment
What was the context?
Recomendations
What recommendations could enhance the intervention?
Atributes
What are the caracteristics of the interventions?
O
P
E
R
A

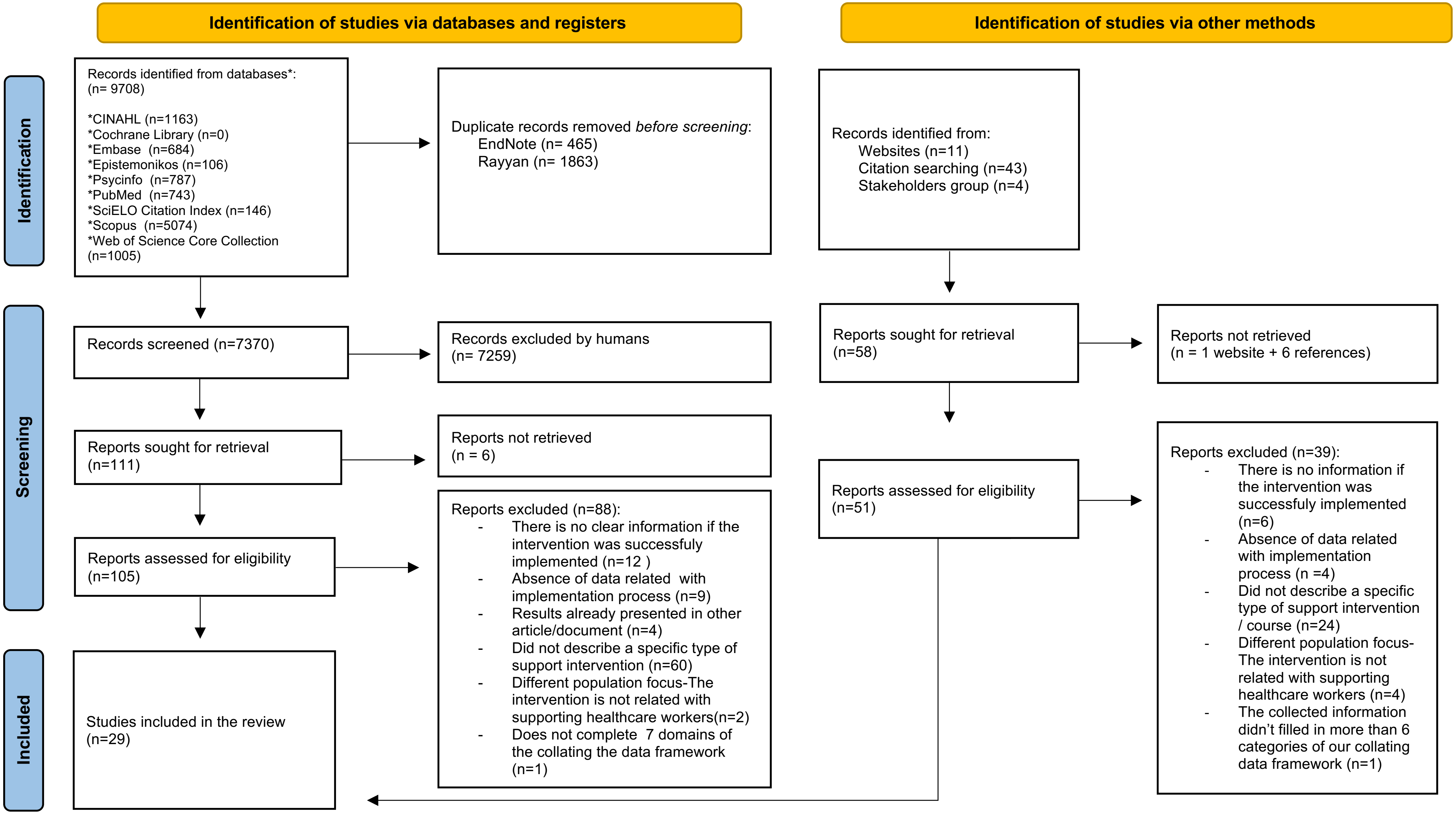
Identification of studies via databases and registers
Identification of studies via other methods
Identification
Screening
Included
Records identified from databases*: (n= 9708)
*CINAHL (n=1163)
*Cochrane Library (n=0)
*Embase (n=684)
*Epistemonikos (n=106)
*Psycinfo (n=787)
*PubMed (n=743)
*SciELO Citation Index (n=146)
*Scopus (n=5074)
*Web of Science Core Collection (n=1005)
Duplicate records removed *before screening*: EndNote (n= 465) Rayyan (n= 1863)
Records identified from: Websites (n=11) Citation searching (n=43) Stakeholders group (n=4)
Records screened (n=7370)
Records excluded by humans (n= 7259)
Reports sought for retrieval (n=58)
Reports not retrieved (n = 1 website + 6 references)
Reports sought for retrieval (n=111)
Reports not retrieved (n = 6)
Reports assessed for eligibility (n=105)
Reports excluded (n=88):
- There is no clear information if the intervention was successfuly implemented (n=12)
- Absence of data related with implementation process (n=9)
- Results already presented in other article/document (n=4)
- Did not describe a specific type of support intervention (n=60)
- Different population focus-The intervention is not related with supporting healthcare workers(n=2)
- Does not complete 7 domains of the collating the data framework (n=1)
Reports assessed for eligibility (n=51)
Reports excluded (n=39):
- There is no information if the intervention was successfuly implemented (n=6)
- Absence of data related with implementation process (n =4)
- Did not describe a specific type of support intervention / course (n=24)
- Different population focus- The intervention is not related with supporting healthcare workers (n=4)
- The collected information didn’t filled in more than 6 categories of our collating data framework (n=1)
Studies included in the review (n=29)

Supplementary Table 1 - Search strategies applied to the different electronic databases

**Applied search strategies :**			**Results**
Context	#1	"Health Services" OR "Health Facilities" OR "Healthcare" OR "Primary health care" OR "General Practice" OR "Family practice" OR "Ambulatory Care" OR "Nursing Care" OR "Family unit" OR "Hospitals"	
Content	#2	"Program evaluation" OR "Support program" OR "Peer support" OR "Support strategies" OR "Organizational factors" OR "Organizational culture"	
Population	#3	("Health personnel" OR "Physicians" OR "Nurses" OR "Doctor" OR "Practitioner" OR "Medical students" OR "Medical residents" OR "Healthcare providers" OR "Healthcare worker" OR "Healthcare staff") AND ("Error" OR "Near miss" OR "Adverse Event" OR "Clinical Error" OR "Medical error" OR "Second victim" OR "Wounded caregiver" OR "Wounded healer" OR "Secondary trauma")	
Web of Science Core collection *All databases* Last updated 2022/10/31	#1 AND #2 AND #3	Error OR Adverse Event OR Clinical Error OR Medical error OR Second victim OR Wounded caregiver OR Wounded healer OR Secondary trauma (Topic) and Health Services OR Health Facilities OR Healthcare OR Primary health care OR General Practice OR Family practice OR Ambulatory Care OR Nursing Care OR Family unit OR Hospitals (Topic) and Program evaluation OR Support program OR Peer support OR Support strategies OR Organizational factors OR Organizational culture (Topic) and Health personnel OR Physicians OR Nurses OR Doctor OR Practitioner OR Medical students OR Medical residents OR Healthcare providers OR Healthcare worker OR Healthcare staff (Topic) and Article or Review Article or Early Access or Editorial Material (Document Types)	1005 results
Complete search on Pubmed (Medline) Last updated 2022/10/31	#1 AND #2 AND #3	((((("Health Services"[MeSH Terms]) OR ("Health Services"[Title/Abstract])) OR (("Health Facilities"[MeSH Terms]))) OR ("Health Facilities"[Title/Abstract])) OR ("healthcare"[Title/Abstract])) OR ("Primary health care"[MeSH Terms])) OR ("Primary health care"[Title/Abstract])) OR ("General Practice"[MeSH Terms])) OR ("General Practice"[Title/Abstract])) OR ("Family practice"[MeSH Terms])) OR ("Family practice"[Title/Abstract])) OR ("Ambulatory Care"[MeSH Terms])) OR ("Ambulatory Care"[Title/Abstract])) OR ("Nursing Care"[Title/Abstract])) OR ("Family unit"[Title/Abstract])) OR ("Hospitals"[Title/Abstract]))) AND (("Program evaluation"[Title/Abstract]) OR ("Support program"[Title/Abstract]) OR ("Peer support"[Title/Abstract]) OR ("Support strategies"[Title/Abstract]) OR ("Organizational factors"[Title/Abstract]) OR ("Organizational culture"[MeSH Terms]) OR ("Organizational culture"[Title/Abstract])))) AND (("Health personnel"[MeSH Terms]) OR ("Health personnel"[Title/Abstract]) OR ("Physicians"[Title/Abstract]) OR ("Nurses"[Title/Abstract]) OR ("Doctor"[Title/Abstract]) OR ("Practitioner"[Title/Abstract]) OR ("Medical students"[Title/Abstract]) OR ("Medical residents"[Title/Abstract]) OR ("Healthcare providers"[Title/Abstract]) OR ("Healthcare worker"[Title/Abstract]) OR ("Healthcare staff"[Title/Abstract])))) AND ((Error[Title/Abstract]) OR (("Near miss"[Title/Abstract]) OR ("Adverse Event"[Title/Abstract]) OR ("Clinical Error"[Title/Abstract]) OR ("Medical error"[MeSH Terms]) OR ("Medical error"[Title/Abstract]) OR ("Second victim"[Title/Abstract]) OR ("Wounded caregiver"[Title/Abstract]) OR ("Wounded healer"[Title/Abstract]) OR ("Secondary trauma"[Title/Abstract])))	743 results

Complete search on PsycInfo<br><br>Last updated 2022/11/3	#1 AND #2 AND #3	(((health services) OR (health facilities) OR (healthcare) OR (primary health care) OR (general practice) OR (family practice) OR (ambulatory care) OR (nursing care) OR (family unit) OR (hospital)) AND ((program evaluation) OR (support programs) OR (peer support) OR (support strategies) OR (organizational factors) OR (organizational culture)) AND ((health personnel) OR (physician) OR (nurse) OR (doctors) OR (practitioner) OR (medical students) OR (medical residents) OR (healthcare providers) OR (healthcare workers) OR (healthcare staff)) AND ((errors) OR (near miss) OR (adverse events) OR (clinical errors) OR (medical errors) OR (second victim) OR (wounded healer) OR (secondary trauma))	787 results
SCOPUS<br><br>Last updated 2022/11/2	#1 AND #2 AND #3	(TITLE-ABS-KEY (health AND services) OR TITLE-ABS-KEY (health AND facilities) OR TITLE-ABS-KEY (healthcare) OR TITLE-ABS-KEY (primary AND health AND care) OR TITLE-ABS-KEY (general AND practice) OR TITLE-ABS-KEY (family AND practice) OR TITLE-ABS-KEY (ambulatory AND care) OR TITLE-ABS-KEY (nursing AND care) OR TITLE-ABS-KEY (family AND unit) OR TITLE-ABS-KEY (hospitals)) AND (TITLE-ABS-KEY (program AND evaluation) OR TITLE-ABS-KEY (support AND program) OR TITLE-ABS-KEY (peer AND support) OR TITLE-ABS-KEY (support AND strategies) OR TITLE-ABS-KEY (organizational AND factors) OR TITLE-ABS-KEY (organizational AND culture)) AND (TITLE-ABS-KEY (health AND personnel) OR TITLE-ABS-KEY (physicians) OR TITLE-ABS-KEY (nurses) OR TITLE-ABS-KEY (doctor) OR TITLE-ABS-KEY (practitioner) OR TITLE-ABS-KEY (medical AND students) OR TITLE-ABS- KEY (medical AND residents) OR TITLE-ABS-KEY (healthcare AND providers) OR TITLE-ABS-KEY (healthcare AND worker) OR TITLE- ABS-KEY (healthcare AND staff)) AND (TITLE-ABS-KEY (error) OR TITLE-ABS-KEY (near AND miss) OR TITLE-ABS-KEY (adverse AND event) OR TITLE-ABS-KEY (clinical AND error) OR TITLE-ABS-KEY (medical AND error) OR TITLE-ABS-KEY (second AND victim) OR TITLE-ABS-KEY (wounded AND caregiver) OR TITLE-ABS-KEY (wounded AND healer) OR TITLE-ABS-KEY (secondary AND trauma)) AND (LIMIT-TO (DOCTYPE , "ar") OR LIMIT-TO (DOCTYPE , "re") OR LIMIT-TO (DOCTYPE , "cp") OR LIMIT-TO (DOCTYPE , "cr"))	5074 results
Complete search on CINAHL<br><br>Last updated 2022/10/31	#1 AND #2 AND #3	TX ("health services" OR "health facilities" OR healthcare OR "primary health care" OR "general practice" OR "family practice" OR "ambulatory care" OR "nursing care" OR "family unit" OR hospital) AND TX ("program evaluation" OR "support programs" OR "peer support" OR "support strategies" OR "organizational factors" OR "organizational culture") AND TX ("health personnel" OR physician OR nurse OR doctors OR practitioner OR "medical students" OR "medical residents" OR "healthcare providers" OR "healthcare workers" OR "healthcare staff") AND TX (errors OR "near miss" OR "adverse events" OR "clinical errors" OR "medical errors" OR "second victim" OR "wounded healer" OR "secondary trauma")	1163 results
Complete search on Embase<br><br>Last updated 2022/10/31	#1 AND #2 AND #3	('health services':ab,ti OR 'health facilities':ab,ti OR 'healthcare':ab,ti OR 'primary health care':ab,ti OR 'general practice':ab,ti OR 'family practice':ab,ti OR 'ambulatory care':ab,ti OR 'nursing care':ab,ti OR 'family unit':ab,ti OR 'hospitals':ab,ti) AND ('program evaluation':ab,ti OR 'support program':ab,ti OR 'peer support':ab,ti OR 'support strategies':ab,ti OR 'organizational factors':ab,ti OR 'organizational culture':ab,ti) AND ('health personnel':ab,ti OR 'physicians':ab,ti OR 'nurses':ab,ti OR 'doctor':ab,ti OR 'practitioner':ab,ti OR 'medical students':ab,ti OR 'medical residents':ab,ti OR 'healthcare providers':ab,ti OR 'healthcare worker':ab,ti OR 'healthcare staff':ab,ti) AND (error:ab,ti OR 'adverse event':ab,ti OR 'clinical error':ab,ti OR 'medical error':ab,ti OR 'second victim':ab,ti OR 'wounded caregiver':ab,ti OR 'wounded healer':ab,ti OR 'secondary traumatic stress':ab,ti)	684 results
Scielo citation index through. Web of Science<br><br>Last updated: 2022/11/4	#1 AND #2 AND #3	("Health Services" OR "Health Facilities" OR "Healthcare" OR "Primary health care" OR "General Practice" OR "Family practice" OR "Ambulatory Care" OR "Nursing Care" OR "Family unit" OR "Hospitals") AND ("Program evaluation" OR "Support program" OR "Peer support" OR "Support strategies" OR "Organizational factors" OR "Organizational culture") AND ("Health personnel" OR "Physicians" OR "Nurses" OR "Doctor" OR "Practitioner" OR "Medical students" OR "Medical residents" OR "Healthcare providers" OR "Healthcare worker" OR "Healthcare staff" and "Error" OR "Near miss" OR "Adverse Event" OR "Clinical Error" OR "Medical error" OR "Second victim" OR "Wounded caregiver" OR "Wounded healer" OR "Secondary trauma")	146 results

For peer review only

Complete search on Epistemonikos<br><br>Last updated 2022/11/2	#1 AND #2 AND #3	(title ((((health services) OR (health facilities) OR (healthcare) OR (primary health care) OR (general practice) OR (family practice) OR (ambulatory care) OR (nursing care) OR (family unit) OR (hospital)) AND ((program evaluation) OR (support programs) OR (peer support) OR (support strategies) OR (organizational factors) OR (organizational culture)) AND ((health personnel) OR (physician) OR (nurse) OR (doctors) OR (practitioner) OR (medical students) OR (medical residents) OR (healthcare providers) OR (healthcare workers) OR (healthcare staff)) AND ((errors) OR (near miss) OR (adverse events) OR (clinical errors) OR (medical errors) OR (second victim) OR (wounded healer) OR (secondary trauma))) OR abstract:(((health services) OR (health facilities) OR (healthcare) OR (primary health care) OR (general practice) OR (family practice) OR (ambulatory care) OR (nursing care) OR (family unit) OR (hospital)) AND ((program evaluation) OR (support programs) OR (peer support) OR (support strategies) OR (organizational factors) OR (organizational culture)) AND ((health personnel) OR (physician) OR (nurse) OR (doctors) OR (practitioner) OR (medical students) OR (medical residents) OR (healthcare providers) OR (healthcare workers) OR (healthcare staff)) AND ((errors) OR (near miss) OR (adverse events) OR (clinical errors) OR (medical errors) OR (second victim) OR (wounded healer) OR (secondary trauma))))	106 results
Language		No language filter/restraint will be applied	
Period		No period filter/restraint will be applied	
Exclusion criteria		Article types not included: editorial , letter to the editor, cases series, case reports, narrative review, commentary	

For peer review only

Supplementary Table 2- Characteristics of the included studies

Authors (year of publication)	Type of study and level of evidence	Main aim of the study	Study design	Methods	Key findings	Quality assessment
Allen,Spencer, McEwan,Catarino , Evans, Crooks et al (2020)	Scientific article – Level VI[1]	To evaluate the experience of HCWs working in a mental health service after attending the Schwartz Rounds.	Mixed Method	Application of a quantitative evaluation form after the rounds and focus group and 6 years follow up.	Rounds were helpful, insightful and relevant to support HCWs in a non-blaming environment. The six-year follow-up revealed that the Rounds were still rated positively.	4*/5 of the MMAT criteria *there are no inconsistencies between results
Bryant (2022)	Graduate Theses, Dissertation s - Level VI[1]	To raise staff awareness on resilience, SVP and SupportingYOU intervention in a large academic children's hospital.	Quantitative descriptive	Application of preintervention and postintervention quantitative surveys,	Intervention increased the staff awareness on SVP, their perception on resilience and contribute for a proactive culture to manage critical incidents, by increasing the sense of feeling cared by the institution.	5*/5 of the MMAT criteria
Civil, Hoskins (2022)	Scientific article- Level VI[1]	To describe the design and implementation of a critical incident peer response team program at Waikato Hospital.	Quantitative descriptive	Application of postintervention quantitative survey.	Positive feedback from the attendants was given after the intervention. The program contributed to a supportive culture and enhanced teamwork.	2*/5 of the MMAT * 3 criteria were evaluated as "can't tell" : the sample is not representative of the target population, there is a risk of nonresponse bias; absence of statistical analysis
Edrees, Connors, Paine, Norvell, Taylor, Wu (2016)	Scientific article- Level VI[1]	To describe the development of RISE, initial evaluation of pilot programme and hospital-wide implementation at Johns Hopkins Hospital.	Mixed Method	Application of pre and post implementation quantitative surveys and focus group.	Although there were few calls in the first year of implementation, the rate of calls increased during the next years. Evaluation indicates the success of most encounters with callers and effectiveness of training to prepared the peer supporters to support second victim.	4*/5 of the MMAT criteria *there are no inconsistencies between results
Finney, Jacob, Johnson, Messner, Pulos, Sviggum (2021)	Scientific article Level VI[1]	To describe the implementation and evaluation of a SV program – Healing Emotional lives of Peers (HELP) in a Department of Anaesthesiology.	Mixed method	Application of post implementation quantitative surveys with open questions.	The program developed during 3 years was successfully implemented in the first 2 years of the program inception and is now a resource for other institutions in the region.	4*/5 of the MMAT criteria *no confounders accounted for in the design and analysis
Foreman (2014)	Paper Level VI[1]	To describe the development and implementation of a plan to help perinatal nurses to cope with stress after critical incidents and deaths in a family birth centre.	Qualitative	Qualitative description of the programme testing and the learning from its implementation.	Feedback on the use of the critical event plan has been positive. Nurses found the plan very useful after a tragedy on the unit related with the death of a new-born.	3*/ 5 of the MMAT criteria * 2 criteria were evaluated as "can't tell": qualitative data collection methods are not described; coherence between qualitative data sources, collection, analysis and interpretation is not clear;
Graham, Zerbi, Norcross, Montross-	Scientific article	To describe the Caregiver Support Team programme implementation in	Mixed-method	Application of baseline and post implementation (3 months follow up)	As an addition to the previous implemented Code Lavender intervention, the program was accepted and positively evaluated. Efforts will	4*/5 of the MMAT criteria *no confounders accounted for in the design and analysis

Thomas, Lobbestael , Davidson (2019)	Level VI[1]	an academic medical centre and evaluate its feasibility.		quantitative surveys . Self reported experiences of attendants.	be made to disseminate the program system-wide.	
Johnson, Simms-Ellis, Janes, Mills, Budworth, Atkinson ,Harrison (2020)	Scientific article Level VI[1]	To evaluate a psychological resilience coaching intervention for National Health Services (NHS) trust sites and university premises.	Mixed-method	Application of pre and post implementation, follow up after the coaching phone call (10–20 days after the workshop) and 4–6 weeks after the workshop. Interviews application.	The program seems to be feasible and effective on improving general resilience of clinicians, by improving their knowledge and confidence in coping after adverse events.	5/5 of the MMAT criteria
Krzan, Merandi, Morvay, Mirtallo (2015)	Scientific article Level VI[1]	To describe YOU Matter Support program implementation at Nationwide Children's Hospital.	Mixed-method	Application of pre and post (after 5 months) online surveys. Collection of peer encounters documentation of the Second Victim SharePoint site.	Most of the surveyed staff reported that the department benefited from implementation of the SV program. After the success of the pilot program, the hospital has decided to expand the YOU Matter program hospital- wide.	4*/5 of the MMAT criteria *no confounders accounted for in the design and analysis
Merandi, Liao , Lewe, Morvay, Stewart, Catt, Scott (2017)	Scientific article Level VI[1]	To describe the replication of the ForYOU Matter program and expansion of the program to a large pediatric institution.	Quantitative descriptive	Description of the program implementation and of the collected data from electronic tracking system via SharePoint.	Application of the MUHC support model in the Nationwide Children's Hospital was validated and demonstrates that it's suitable and transferrable to other healthcare facilities and contexts.	3*/5 of the MMAT criteria 2 criteria was evaluated as "can't tell": no clear information if the risk of nonresponse bias was low: absence of statistical analysis
Lane, Newman, Taylor, O'Neill, Ghetti, Woltman, Waterman (2018)	Scientific article Level VI[1]	To describe the Washington University School of Medicine Clinician Peer Support Program.	Quantitative descriptive	Description of the program development and its implementation.	Program was successfully implemented, however difficulties were found in sustaining the program over time, since peer supporters don't have protected time to be part of the program and therefore the programme activities have conflict with their operative schedules.	3*/5 of the MMAT criteria – *2 criteria was evaluated as "can't tell": nonresponse bias was not applicable to this study; absence of statistical analysis
Mellins, Mayer, Glasofer, Devlin, Albano, Nash et al (2020)	Scientific article Level VI[1]	To describe the application of CopeColombia programme in a Department of Psychiatry of a large urban medical centre's.	Mixed method	Description of the key issues/themes and facilitator responses emerged in the sessions (facilitator interventions); post peer supporter group online survey (perceived impact of the program).	HCW emotional distress decreased after the program implementation. Peer Support Groups were the most used, valued and recommended. Sustainability of the program is critical due to financial constrains.	3*/5 of the MMAT criteria *there are no inconsistencies between results; 1 criteria was evaluated as "can't tell": it's not clear if the study follows the traditional quality criteria of the qualitative methods involved
Merandi, Winning , Liao, Rogers, Lewe, Gerhardt (2018)	Scientific article Level VI[1]	To assess healthcare providers satisfaction in the early implementation of a SV program in a group of neonatal intensive care units.	Mixed method	Application of pre and post implementation surveys with closed and open – ended questions. Thematic content analysis of the qualitative data from the survey.	This study suggest that peer support programs are likely to be viewed as positive and helpful for frontline HCW and managers. However, additional work should be done to assure the accessibility and effectiveness of the program.	4*/5 of the MMAT criteria *no confounders accounted for in the design and analysis

For peer review only

Rivera-Chiauzzi,Smith,Moore-Murray, Lee, Goffman, Bernstein, Chazotte (2022)	Scientific article<br><br>Level II[1]	To describe the development and evaluation a structured peer support program to address the needs of providers involved in obstetric adverse outcomes and to compare it with a routine support for HCW after the same type of events.	Pilot Randomized controlled trial	Application of needs assessment survey before program implementation. Surveys were applied at baseline, 3 months, and 6 months follow up.	Structured peer support program was successfully implemented with limited resources. The enhanced support group was significantly more likely to consider departmental leadership as one of the most helpful resources for support after adverse event. All participants refer were thriving at 6-month follow-up.	4*/5 of the MMAT criteria<br><br>*outcome assessors are not blinded to the intervention provided
Roesler, Ward, Short (2009)	Scientific article<br><br>Level VI[1]	To describe the recovery and reintegration of Neonatal Intensive Care Unit staff after a critical incident based on the Just Culture philosophy.	Qualitative: Single Case	Description of the protocol activation and application after the involvement of a severe adverse event.	The protocol was successfully applied and helped the affected HCW to thrive and return back to work after their involvement in the severe adverse event.	3*/5 of the MMAT criteria<br>*2 criteria were evaluated as “can’t tell”: qualitative data collection methods are not described ; it’s not clear if there is coherence between qualitative data sources, collection, analysis and interpretation
Schrøder, Bovil,, Jørgensen, Abrahamsen (2022)	Scientific article<br><br>Level VI[1]	To evaluate the Buddy Study program by assessing HCW experiences with having the program in the department, attending the compulsory seminar, and using a buddy or being activated as a buddy.	Mixed method	Application of quantitative questionnaires to assess HCW experiences of attending the Buddy Study seminar and participating in the Buddy Study program using a buddy or being activated as a buddy, along with two open-ended questions and three individual interviews for more elaborate answers.	The buddy study program was evaluated positively. It allowed to acknowledge the SVP and strengthen a supportive organizational culture by creating buddy relationships to support HCW after stressful events during care.	4*/5 of the MMAT criteria *no confounders accounted for in the design and analysis
Shapiro, Galowitz (2016)	Scientific article<br><br>Level VI[1]	To describe the development and implementation of Brigham and Women’s Hospital peer support program to support HCW after the impact of an emotional stressful event.	Quantitative descriptive	Description of the program and quantitative data collection of outreached calls and peer support sessions.	The peer support program has been implemented for 4 years and it’s expected to be expanded hospital-wide. The program does not yet reach many clinicians that might be in need of support after stressful events.	3*/5 of the MMAT criteria<br>*3 criteria were evaluated as “can’t tell”: it’s not clear if the sample is representative of the target population; risk of nonresponse bias low and statistical analysis are not applicable
Thompson, Hunnicutt, Broadhead, Vining, Aroke (2022)	Scientific article<br><br>Level VI[1]	To describe the implementation of SV support program in a large academic medical centre based on a quality improvement project.	Quantitative descriptive	Application the SVEST survey pre and post peer support program implementation.	Although the study didn’t find statistically significant differences in pre and post implementation the program has received a positive feedback among leadership members and peer supporters.	4*/5 of the MMAT criteria<br>*2 criteria were evaluated as “can’t tell”: low response rate in post-implementation survey may have decreased generalizability of results which can affect the sample representative ; potential for response bias was identified in the study

El Hechi , Bohnen, Westfal, Han, Cauley, Wright, Schulz et al (2020)	Scientific article<br><br>Level VI[1]	To describe the design and implementation of a SV surgical peer support program and its 1 year impact.	Mixed method	Programme design: literature review and multidisciplinary expert group discussion**;** Evaluation of the impact of the programme: quantitative and qualitative surveys application – evaluation of the peer supporters and of the program.	The first surgery-specific peer support program in US was successfully implemented. After 1 year experience, the program is highly used and well received.	4/5 of the MMAT criteria<br>*inconsistencies between quantitative and qualitative are not addressed in the study
Mira, Carrillo, Guilabert, Lorenzo, Pérez-Pérez, Silvestre et al (2017)	Scientific article<br><br>Level VI[1]	To describe the development and assessment of Mitigating Impact in Second Victims (MISE) programme on the awareness and information focused on the SVP.	Quantitative descriptive	Description of accreditation process; quantitative survey applied to 26 experts to assess structure and content; Quantitative survey applied to frontline HCW; evaluation of knowledge gained from the online program after each online module.	The online program was positively assessed by the accreditation agency, by the experts and the HCWs. The programme increases knowledge about patient safety, SVP and how to act after a severe adverse event. The time demand is reasonable for complete the course.	5/5 of the MMAT criteria
Scott, Hirschinger, Cox, McCoig, Hahn-Cover, Epperly et al. (2010)	Scientific article<br><br>Level VI[1]	To describe the deployment of ForYOU Team programme , an institutional rapid response system (RRS) for second victims at University of Missouri Health Care (MUHC).	Mixed method	Interviews and quantitative surveys application by the MUHC faculty and staff to support the development of the programme.	After identifying the need for support the SV in MUHC , the programme was designed, developed and successfully implemented. The programme has been integrated in the healthcare in the scheduled activities of the team leaders and clinical teams.	4*/5 of the MMAT criteria<br>*inconsistencies between quantitative and qualitative are not addressed in the study
Schuster (2021)	Scientific article<br><br>Level VI[1]	To describe the implementation of the HART programme and assess its impact on the Hematology/Oncology/Stem Cell Transplant Unit.	Mixed method	Preintervention and midpoint survey (after 3 months of pilot program), and qualitative subjective information collection from daily coach documentation entries.	HART programme was successfully implemented in the department and with high level of utilization from the staff. After implementation of HART, mental, emotional and physical wellbeing of all staff members improved.	4*/5 of the MMAT criteria<br>*inconsistencies between quantitative and qualitative are not addressed in the study
Calder-Sprackman; Kumar; Gerin-Lajoie; Kilvert; Sampsel (2018)	Scientific article<br><br>Level VI[1]	To describe the implementation, adaptation and evaluation of the ice cream rounds intervention in an emergency medicine training programme.	Quantitative descriptive	Application of a pre-implementation survey for needs assessment and a post implementation for feedback after intervention.	This Canadian initiative increased the overall perception of support and companionship, decreased feelings of stress, anxiety and burnout and can have a positive impact on the clinical practice in emergency medicine residents.	3*/5 of the MMAT criteria<br>*2 criteria were evaluated as "can't tell": it's not clear if the sample is representative of the target population; absence of statistical analysis

For peer review only

Peterson, Bergström, Samuelsson, Åsberg, Nygren (2008)	Scientific article<br><br>Level II[1]	To evaluate the effect a reflecting peer-support group on self-reported health, burnout and on perceived changes in work conditions of multidisciplinary group of HCW in 3 hospitals of a county council in Sweden.	Randomized controlled trial.	Application of a baseline survey (7 months before the interview started), pre implementation questionnaire , immediately after the intervention ended (post intervention), 7 and 12 months after the intervention follow up.	The study showed positive intervention effects (after 12 months of the programme implementation) on overall health and decreased perceived work demands. A decrease in exhaustion, depression and anxiety symptoms was observed , as well as an increase in vitality.	5/5 of the MMAT criteria
Rubin, Rassman (2021)	Scientific article<br><br>Level VI[1]	To describe the social work led peer support model, COVID-19 Am I Resilient (cAIR), developed and implemented during the first wave of the COVID-19 pandemic in a large urban healthcare system.	Mixed method	Application of pre and post intervention surveys with quantitative and open ended questions.	The pilot programme cAIR was successfully implemented and provided support to normalize frontline HCW experience during Covid 19 pandemic, encouraged coworkers connections and developed coping skills . Participant engagement in cAIR were strong, however overall utilization of programme activities was low.	4*/5 of the MMAT criteria<br>*inconsistencies between quantitative and qualitative are not addressed in the study
Bernburg, Groneberg , Mache (2019)	Scientific article<br><br>Level II[1]	To develop, implement and evaluate the effectiveness of a mental health intervention programme on nurses' perceived job stress, perceptions of individual coping skills and the quality of patient relations in a group of psychiatric hospital departments.	Randomized controlled trial	Pre and post intervention surveys were applied using Perceived Stress Questionnaire; the Brief Resilient Coping Scale; the Self- Efficacy, Optimism and Pessimism Questionnaire; Emotion Regulation Skills Questionnaire and the German Quality of Relationship Inventory – at baseline , follow up after 3 months, 6 months and after 12 months.	After the intervention being successfully implemented, psychiatric nurses significantly increased their perception about job stress, emotion regulation skills, resilience, and self-efficacy; quality of patient-relationship were significantly higher ($p < 0.05$). The results indicate that self- care skills training may improve such as resilience and self-efficacy.	4*/5 of the MMAT criteria<br>*outcome assessors are not blinded to the intervention provided
Morales, Brown (2019)	Scientific article<br><br>Level VI[1]	To describe the development of the Care for the Caregiver Program in a 10 hospital system.	Qualitative	Description of the "Care for the Caregiver" programme and of practical scenarios after the programme was been activated.	Both described scenarios of support after stressful events indicate that the programme has been useful and well received by the clinicians.	4*/5 of the MMAT criteria<br>* 1 criteria was rated as can't tell : coherence between qualitative data sources, collection, analysis and interpretation is not applicable in this study
Cobos-Vargas, Pérez-Pérez, Núñez-Núñez, Casado-Fernández, Bueno-Cavanillas (2022)	Scientific article<br><br>Level VI[1]	To describe the Procedure for Serious Adverse Events (PSAE), integrated into a sentinel event operational procedure and to describe its accumulated results over two years of implementation in a university hospital.	Mixed method	Collection of quantitative data related with programme application, characteristics of involved HCWs and adverse events; HCWs qualitative feedback on programme improvement solutions and	The programme had a positive impact on patient safety culture and it increased the adverse event reporting in the hospital. The level of programme acceptance was high and HCWs valued to be individually interview after their involvement in the event and to contribute for future improvement actions.	4*/5 of the MMAT criteria<br>*inconsistencies between quantitative and qualitative are not addressed in the study

For peer review only

				their experience after attending the programme.		
Hinzmann, Forster, Koll-Krüsmann, Schießl , Schneider, Sigl-Erkel et al (2022)	Scientific article<br><br>Level VI[1]	To evaluate the burden, benefits, and mechanisms of action of a telephone support service for HCWs (PSU-HELPLINE).	Mixed methods	Application of two surveys: i) quantitative survey during the call(socio-demographic data, current stress ,relief from the counselling interview); ii) meta-questionnaire (to document the call and for quality assurance proposes) . Memory transcripts for thematical analysis were also collected.	The PSU-HELPLINE was mainly used for processing serious events and in phases of overload. The programme was well received by a specific region of Germany and callers consider it useful and supportive service.	4*/5 of the MMAT criteria<br>*inconsistencies between quantitative and qualitative are not addressed in the study

For peer review only

Supplementary Table 3 - Characteristics of the included interventions

Note: We have contacted the authors to complete the missing information, however some of the authors didn't reply to our contact.

**Author (Year of publication)**	**Starting year of the intervention**	**Duration of the intervention**	**Country and Setting**	**Target population**	**Aim of the intervention**	**Main outcomes related with the intervention**
Allen,Spencer , McEwan,Catarino, Evans, Crooks et al (2020)	Missing data	6 months	UK - Mental health service, inpatient acute psychiatric unit	Nursing care, healthcare assistance, occupational therapy, psychiatry, psychological assistance, domestic support building states, administration services, crisis resolution , home treatment team and perinatal mental health team	To understand experiences of emotional distress and reflecting on its impact and ways of coping	**i) Evaluation of Schwartz Rounds*:** From an average evaluation form response was 3.42 out of 4;<br>**ii) Experience of the participants after attending Schwartz Rounds:** In the Rounds, HCWs could express their emotions in an non-judgemental and empathic environment. Rounds were considered helpful, insightful and relevant;<br>**iii) Long-term follow-up of 6 years**: Rounds were still rated positively.<br>*Based on the following criteria: plan to attend the Rounds again, gained insight into thoughts and feelings, facilitator helped the discussion, the overview and presentation of the Rounds were helpful, it helped to improve the relation with colleagues in the work, the knowledge was useful for caring patients, the case was relevant to the clinical work
Bryant (2022)	2021	13 months	US - Intensive care unit, lactation services, child-life services, paediatrics medical surgical units	Staff members from lactation services and paediatric medical-surgical units. child life specialists, physicians, nurses, respiratory therapists, social workers, clinical associates, pharmacist and chaplains	To raise awareness of SVP and SupportingYOU. To increase resiliency by establishing a proactive culture to manage critical incidents prior they occur	**i) Staff perception on resiliency**: 100% of the surveyed staff reported an enhanced perception of resilience (p<0,001, CI 95%);<br>**ii) Staff awareness of SVP** significantly increased (p<0,001, CI 95%);<br>**iii) Staff awareness of SVP resources and of SupportingYOU**: it was limited since that most of participants were already aware of it;<br>**iv) Sense of preparedness in handling critical incidents**: there was an increased sense of managing emotional response after critical incidents among the participants (p<0,01, CI 95%);<br>**v) Feeling cared by the institution –** participants felt care for in the workplace (p<0,01, CI 95%).
Civil, Hoskins (2022)	2018	36 months	New Zealand – Department of Anaesthesia and Pain Medicine	Anaesthetists, surgeons, anaesthetist technicians, theatre nurse coordinator/charge nurse; theatre and recovery room nurses and midwifes	To provide peer-led group psychological first aid to full theatre team stressful events?	**i) Number of participants that attended the defuse:** over 200 members;<br>**ii) Number of interventions** : 28 defuse interventions have been delivered;<br>**iii) Participants feedback of the defuse** : Defuses were rated through neutral to very helpful, all surveyed participants would attend a defuse again in the future and would recommend to colleague or a friend.

For peer review only

Edrees, Connors, Paine, Norvell, Taylor, Wu (2016)	2011	52 months (from november 2011 to march 2016)	US- Johns Hopkins Hospital	All hospital staff	To provide timely psychological first aid and emotional support after critical incidents based on a call system	**i) Frequency of encounters:** 119 encounters from November 2011 and march 2016;<br><br>**ii) Caller interaction:** rated as excellent 66,7% or neutral (22,8%).
Finney, Jacob, Johnson, Messner, Pulos, Sviggum (2021)	2018	23 months (from July 2018 to June 2020)	US- Department of anaesthesiology at a large academic institution in the Midwest	Anaesthesiologist, certified nurse anaesthetics , residents, students registered nurse anaesthetics	To provide three-tiered support psychological first aid : first level given by peer and leadership at the local, second level provided by trained peer supporters; third level was provided by external services (from the instituition or outside the instituition)	**i) Experience after receiving peer support:** From 31 surveyed participants, 25 (80.0%) evaluated the received support as "extremely" or "very beneficial", and 28 (90.3%) referred that were "extremely" or "very satisfied" with their experience; 30 (96.8%) would recommend HELP programme to a colleague;<br><br>**ii) HELP Programme Activations for Peer Support:** 91 electronic activations were utilized to assist 179 HCWs (the most common events leading to HELP activation were intraoperative patient demise, cardiac arrest, and pediatric care);<br><br>**iii) Trained peer support self-assessments:** From 85 surveyed peer supporters, 81 (95.3%) felt satisfied with how the peer support encounter turned out; 19 (22.4%) felt that they needed additional training and experience, 80 (94.1%) felt comfortable with their knowledge and skills as a peer supporter.
Foreman (2014)	Missing data	Info missing	US- Family birth centre Wilcox Women's Pavillon at Legacy Good Samaritan Hospital	All perinatal nurses of the family birth caring for women and newborns in all phases of childbearing	To create a critical event plan and implement it in the centre for helping staff to organize and manage critical events	**i) Experience on the use of the Critical Event Plan:** Nurses found it very useful and helpful**.**
Graham, Zerbi, Norcross, Montross-Thomas, Lobbestael , Davidson (2019)	Missing data	3 months	US- 4 target units in an academic medical centre: telemetry unit, emergency department, neonatal ICU and medical and neurologic ICU	Staff and physicians employed in the units	To provide emotional first aid in the workplace after critical events	**i) Programme activation :** 38 activations of the programme;<br><br>**ii) Experience after the programme:** all surveyed staff found it helpful and would recommend it to other colleagues; HCWs referred an improvement in feeling cared-for and also noted the sense of safety at the workplace;<br><br>**iii) Quality of life assessment and job satisfaction**: No significant changes were demonstrated before and after the intervention in Quality ofLife Scores or job satisfaction. One suicide was prevented;<br><br>**iv) Organizational changes :** Debriefings started to be requested after significant events affecting the entire department.
Johnson, Simms-Ellis, Janes, Mills, Budworth, Atkinson	2018	7 months	UK- National Health Services (NHS) trust sites and University of Leeds	HCWs or students that complete an education programme: midwives, paramedics , obstetrics and gynaecology trainee	To strengthen resilience and preparedness in dealing with adverse events : more flexible thinking, higher self-	**i) Experience after the programme:** workshop was useful, relevant and adequate in length**.** The participants highly valued the peer learning, the level of engagement and the format of the workshop delivery. The coaching call was critical to the consolidation of the knowledge and to

For peer review only

,Harrison (2020)				doctors , paediatric trainee doctors, paediatric consultant, physician associate students and sonography and mammography students.	esteem, better explanatory style	understand how to apply the acquired skills in practice. Knowledge about coping strategies led to a significant increase in self-perceived resilience, as well as confidence in coping with adverse events.
Krzan, Merandi, Morvay, Mirtallo (2015)	2013	5 months	US- pharmacy department at Nationwide Children's Hospital (NCH): one main inpatient pharmacy, inpatient pharmacy satellites, two outpatient pharmacies and home care pharmacy	Staff from all areas of pharmacy services	To provide three-tiered support psychological first aid (based on Susan's Scott model) to support HCWs involved in adverse drug events, patient-related injuries, and other traumatic work experiences	**i) Activation of the programme:** 3 respondents personally used the programme and 11 had recommend it;<br><br>**ii) Experience after the programme :** 85% of the pharmacy (95 of 112 respondents) refer that the department had benefit from the programme's implementation.
Merandi, Liao, Lewe, Morvay, Stewart, Catt, Scott (2017)	2012	60 months (5 years)	US- Nationwide Children's Hospital (NCH)	Staff from Nationwide Children's Hospital (NCH): all inpatient units as well as urgent cares, outpatient primary care clinics, and ambulatory specialty clinics	To provide three-tiered support psychological first aid (based on Susan's Scott model) to support HCWs involved in adverse drug events, patient-related injuries, and other traumatic work experiences	**i) Activation of the programme:** 21 group encounters were documented since November 2013;<br><br>**ii) Quantitative description of peer encounters:** 62% of peer encounters occurred in the emergency department, and 8% in pediatric intensive care unit and cardiothoracic intensive care unit ; nurses have the highest number of peer support encounters. Patient death and emotional stress were the most common reasons for peer encounter.
Lane, Newman, Taylor, O'Neill, Ghetti, Woltman, Waterman (2018)	2014	33 months	US- Two Hospitals affiliates of the Washington School of Medicine:<br>Barnes -Jewish Hospital and St. Louis Children's Hospital	Doctors, junior doctors, physician associates, nurse practitioners, and registered nurse anaesthetists, trainees providing care in the inpatient and outpatient settings	To facilitate the support of clinicians who have been involved in an adverse event or another adverse outcome during medical care	**i) Programme activation:** 165 clinicians were referred to the programme;<br><br>**ii) Median number of interactions per month:** 4.8 referrals per month (Range 0-12).
Mellins, Mayer, Glasofer, Devlin, Albano, Nash et al (2020)	2020	3 months	US-Large urban , academic, tertiary care - Columbia University Irving Medical Centre (CUIMC)	All CUIMC clinical and non-clinical staff, residents and faculty students	To provide peer support after stressful situations and enhance resilience of HCWs	**i) Emotional distress assessment:** The average emotional distress had significantly decreased (*p*<0.05);<br><br>**ii) Perceived helpfulness of the peer support group:** Perceived helpfulness was high (76% rating helpfulness as "quite a bit" or "extremely."); all respondents (with the exception of two) recommended the support group to a colleague.

Merandi, Winning , Liao, Rogers, Lewe, Gerhardt (2018)	2015	12 months	US-7 neonatal intensive care units (NICUs) from quaternary-care, paediatrics hospital	All staff from the 7 NICUs	To provide three-tiered support psychological first aid (based on Susan's Scott model) to support HCWs involved in adverse drug events, patient-related injuries, and other traumatic work experiences	**i) Attendance to the programme:** A small number of healthcare providers (n=6) used the program. Some HCWs (not quantified) did not find the programme accessible.<br>**ii) Satisfaction with the programme:** The majority of the participants (56.3%, n=9) reported moderate benefit of the programme. From 250 surveyed HCWs in the units, 73.2% indicated that the NICUs benefited at least a little from the SV peer support programme. The manager reported that the programme enhanced the team leadership.
Rivera-Chiauzzi,Smith,Moore-Murray, Lee, Goffman, Bernstein, Chazotte (2022)	2015	8 months	US- Department of obstetrics & Gynaecology and Women Health	HCWs and residents (physicians and nurses) who experienced an obstetric adverse outcome.	To provide peer support to HCWs and residents, who have experienced an obstetric adverse outcome, through phone calls	**i) Attendance to the programme :** Out of 34 programme activations, 23 participants were placed in the enhanced group and 19 were assigned in the routine group;<br>**ii) Use of support resources and perception of its helpfulness:** Peer support was the most common source of assistance (*p*<0.05); departmental leadership was considered one of the most helpful resources for the enhanced support group;<br>**iii) Identified barriers:** Time (*P* = 0.26) was reported as a constraint by participants in the enhanced group, and routine group reported privacy concerns (*P* = 0.39) and stigma (*P* = 0.12)**;**<br>**iv) Effect of the programme on the stage of recovery:** At 6-month follow-up, all participants in the enhanced group reported that they were thriving after the event;<br>**v) Duration of peer support interventions:** most participants required less than 3 months of support to recover from the event.
Roesler, Ward, Short (2009)	2006	No clear information (estimated time : approx. 6 months)	US- Neonatal Intensive Care Unit (NICU), Methodist Hospital of Indianapolis	Staff of the NICU	To provide an institutional incident response after a paediatric serious adverse event	**i) Staff and unit recovery:** The pharmacist technician and 5 out of 6 nurses affected by the incident were back to work after the adverse event (1 nurse didn't return to work for familiar reasons). None of the affected HCWs experienced job changes. Debriefing and healing sessions were essential to the staff unit overcome the situation;<br>**ii) Impact on work culture**- Reinforcement of the culture of disclosure after applying the protocol.
Schrøder, Bovil,, Jørgensen, Abrahamsen (2022)	2018	18 months	Denmark-2 departments at Odense University Hospital: Department of Obstetrics and Gynaecology (OB-GYN) and Svendborg	All midwives in the OB-GYN and all physicians at the IME	To provide one-on-one peer support to HCW following a stressful event, including adverse events	**i) Attendance to the programme:** Out of 156 respondents, 26 of them reported using one of their buddies during the study period**;**<br>**ii) Evaluation of the Buddy Study programme in the department:** The programme encouraged more attentiveness among participants towards one another following adverse events (n=82, 52.6%) ; have

For peer review only

			and Internal Medicine and Emergency Department (IME)			contributed to more inter-collegial talks about adverse events (n=57,36.5%) ; sense of more openness to talk with colleagues about feelings in the aftermath of adverse events (n=65, 41.7%) ; increased willingness to ask for leadership support (n=53, 34.0%);<br>**iii) Overall experience with the programme:** an open and compassionate culture was encouraged; increased attentiveness to the staff wellbeing ; increased sense of safety (n=91, 58.3%).
Shapiro, Galowitz (2016)	2012	47 months (January 2012 and December 2015)	US- Brigham and Women's Hospital (BWH)	HCWs of Brigham and Women's Hospital	To provide one-on-one peer support or group peer support (if a team is likely to be affected), after a stressful event, including adverse events	**i) Activation of the programme:** 220 outreach calls to one-on-one peer support;<br>**ii) Attendance to the group sessions:** 240 clinicians participated in multidisciplinary group peer support sessions.
Thompson, Hunnicutt, Broadhead, Vining, Aroke (2022)	May, 2020 through October, 2020	1 month (trial period)	US – Anaesthesia department of the large academic centre	Certified registered nurses of the anaesthesia department.	To provide a peer support program to decrease SV distress after stressful events	**i) Frequency of the encounters:** Over the course of one month, a total of 8 peer support encounters were reported;<br>**ii) HCWs' distress**: It wasn't possible to observe a significant change in distress level after one month period, however the study found a statistically significant relationship between increased distress and insufficient colleagues support.
El Hechi , Bohnen, Westfal, Han, Cauley, Wright, Schulz et al (2020)	Missing data	12 months	US-Department of surgery at a tertiary academic medical centre	Surgeons and surgical trainees from general surgery, transplantation, pediatric surgery, thoracic surgery, cardiac surgery, vascular surgery, and trauma/acute care surgery.	To provide a peer support - based on three-tiered support psychological first aid - to surgeons and surgical trainees dealing with intraoperative adverse events, catastrophic patient outcomes, and/or long- term litigation cases.	**i) Programme activation:** 47 outreach calls;<br>**ii) Evaluation of the programme impact:** The majority of surgical staff was satisfied with the following domains – programme's confidentiality (89%), safe/trusting environment (73%,), timeliness of the intervention (83%). It was also perceived a positive impact on the department's culture, as it raised awareness about the importance of supporting colleagues facing difficult situations at work, and fostered a general sense of "safety and support" in the workplace.
Mira, Carrillo, Guilabert, Lorenzo, Pérez-Pérez, Silvestre et al (2017)	2015	15 months	Spain – without a specific location (online programme)	HCWs from hospitals and primary care that access the webpage of the online programme	To provide an online preventive programme to mitigate the impact of severe adverse events in HCWs and raise awareness about the SV phenomenon.	**i) Knowledge improvement (main outcome):** After completing the informative and demonstrative package, users significantly improved their knowledge about patient safety terminology, impact and prevalence of adverse events, SV support strategies and recommended actions following a severe adverse event (*P*<0.001).

For peer review only

Scott, Hirschinger, Cox, McCoig, Hahn-Cover, Epperly et al. (2010)	2009	10 months	US-University of Missouri Healthcare	University of Missouri Health Care faculty and healthcare staff	To provide a peer support programme to facilitate the second victim's transition through the six stages of emotional recovery	**i) Frequency of the encounters**: 49 encounters with forYOU Team members ,13 of which involved referrals to external support; there were 6 team debriefings with an average of 15 HCWs each. The average duration of the encounter with forYOU Team members lasted 30 minutes, while debriefings extended to 77 minutes.
Schuster (2021)	End of 2019	6 months	US- Department of Hematology/Oncology /Stem Cell Transplant Unit- Boston Children's Hospital – 2 separate units: a 30-bed hematology/oncology unit and a 14-bed stem cell transplant unit. Both units are under one hematology/oncology/ bone marrow transplant department within the inpatient pediatric free-standing children's hospital.	Multidisciplinary staff caring for hematology/oncology patients and their families (registered nurses, advanced practice nurses, clinical assistants, physicians, dieticians, child life specialists, environmental services, food tray distributors, patient experience representatives, social workers, resource room staff, chaplains, pain service staff, supply restockers, and students on the floor).	To provide peer to peer support program to promote a safer, supportive and resilient workplace culture, improve staff wellbeing and decrease the frequency of contacts among colleagues for work-related support outside of working hours to cope with stressful situations	**i) Frequency of the programme interventions:** 98 HART shifts;<br>**ii) Outcomes related with HART application:** improvement of HCWs' well-being, the number of HCWs reaching out coworkers for support outside working hours decreased after implementing HART; number of breaks increased during the work shifts, the use of hospital resources increased after HART coaches recommend them;<br>**iii) Satisfaction with the work-related support after HART implementation:** 25.6% of participants reported to be extremely satisfied with the received support; 49.4% of HCWs felt more supported by leadership.<br>**iv) Interactions resolutions:** The majority of interactions were solved in real time (83.2%);<br>**v) Subjective Feedback:** The programme fostered a safe, supportive and open work environment. HCWs felt more connected to their colleagues in the workplace.
Calder-Sprackman; Kumar; Gerin-Lajoie; Kilvert; Sampsel (2018)	2014	24 months	Canada – Department of emergency medicine , university of Ottawa	Residents from the emergency department at the university of Ottawa	To provide a peer-support sessions to improve residents wellbeing and create a supportive and resilient workplace	**i) Perception of change after the support programme**: From a total of 20 surveyed HCWs, 95% referred that support and companionship among residents increased after the support the sessions; 58,8% gained an increased awareness of coping strategies to deal with challenges during residency ;<br>**ii) Impact of the Rounds on clinical practice:** Half of HCWs referred that the rounds helped them to reflect about their clinical practice, a quarter of HCWs expressed that the rounds had not significantly impacted their clinical practice. Nevertheless, they acknowledged the value of having this type of support available to them;

						**iii) Perception of stress, anxiety and burnout**: 20% of HCWs decreased sensation of burnout and 10% decrease their perceived stress and anxiety;<br><br>**iv) Programme recommendation:** 89% of respondents would recommend ice cream rounds to other colleagues
Peterson, Bergström, Samuelsson, Åsberg, Nygren (2008)	2002	2 months	Sweden- 3 hospitals of the county council	Physicians, registered nurses, nursing assistants, social workers, occupational therapists, physiotherapists, psychologists, dental nurses and hygienists, dentists, service staff, administrators, teachers and technician. participants scored above the 75th percentile on the exhaustion dimension of the Oldenburg Burnout Inventory	To provide a reflecting peer-support group to discuss and reflect on work-related stress and burnout; to help to find out alternative ways to handle perceived stressful situations on an individual level; to provide an opportunity for mutual support between colleagues, to share and compare experiences and learn from each other.	**i) Frequency of the encounters:** 8 peer-support groups with 5–8 participants in each group**;**<br><br>**ii) Perceived impact of the peer support group:** The space for reflection on 'real problems' was valued by the participants; increased knowledge about stress and coping strategies, increased sense of belonging/community; Increased self-confidence;the existence of structured group was appreciated, decreased stress symptoms and anger; behavioural change; sleep improvement;<br><br>**iii) Work-related symptoms and burnout:** Both groups showed an overall decrease from baseline to follow up after 12 months in exhaustion, disengagement, depression and anxiety measures;<br><br>**iv) General health and vitality:** both increased after the intervention;<br><br>**v) Change in work conditions**: statistical significant difference was found in participation at work and support at work after 12 month follow-up.
Rubin, Rassman (2021)	2020	"During spring"	US-large urban healthcare system: Clinical education and practice department in Swedish Health Services	Clinical and non-clinical staff (nurse educator, professional development specialist, programme manager, student intern) and disciplines (nursing, social work) within the Clinical Education and Practice department.	To provide informational and emotional support to help frontline staff thrive in the first wave of the Covid 19 Pandemic	**i) Programme utilization:** 13 out 71 HCWs participated in the programme;<br><br>**ii) Evaluation of programme's resources:** Approximately 85% of surveyed HCWs agreed or strongly agreed that there were adequate resources to support the HCWs during and after stressful events in the healthcare organization. All respondents agreed that assisting the cAIR video presentation was a valuable use of their time. The nature of the content and dedicated time for staff support stood out as the most helpful aspects of the video presentation. Most respondents improved their knowledge and/or skills with the cAIR resources.

For peer review only

Bernburg, Groneberg , Mache (2019)	Missing data	3 months	Germany - psychiatric hospital departments	Nurses full time working in psychiatric hospital department	To provide a mental health promotion intervention to develop self-care skills in psychiatric nurses	**i) Programme's attendance:** 44 nurses were part of the intervention group and 42 nurses of the control group;<br>**ii) Perceived job stress:** intervention group perceived lower levels of stress after attending the programme;<br>**iii) Relation with patients**: After the programme, significant improvements in nurses' relationship with their patients and lower levels of perceived conflicts were observed ($p < 0.05$);<br>**iv) Emotion regulation:** Large effects on emotion regulation skills were found in the first follow up (3 months) and medium effects sizes were found after 6 and 12 months;<br>**v) Resilience and self-efficacy:** statistically difference was found in these measures within groups (p<0.05)**;**<br>**vi) Final course evaluation:** Overall satisfaction with the training (1.39; 1-best score, 5-worse score); nurses displayed a strong level of motivation and interest in learning self-care techniques and applying them in their work. All participants verified that the training was worth attending (learning was meaningful and motivating for selfcare).
Morales, Brown (2019)	Missing data	No info	US- 10-hospital health system	Clinicians working in the 10-hospital system	To provide immediate emotional first aid after a serious adverse event and coach to HCWs on how to respond to patients and their families in a timely, empathetic, consistent, and patient-centred way.	**i) Process and outcome's results of two different scenarios have been described:**<br>**Scenario 1 (death after health condition deterioration of a patient affected all ICU nursing team) -** Process outcomes: programme activation was done by the nursing director; group sessions were applied to all nurses of the ICU , Employee Assistant Programme was provided to several nurses; Outcome results: after nurses had receive support from the programme, they described "feeling grateful for the opportunity to talk openly without judgment" about the event. Sessions also enabled for peer support among the nursing team members;<br>**Scenario 2 (intensivist involved in a stressful situation during intubation of a patient) -** Process outcomes: critical care unit director activated the programme; "Thinking of You" bag was delivered to the HCW in need. **Outcome results**: the intensivist expressed gratitude for both unit staff and members of the programme.
Cobos-Vargas, Pérez-Pérez, Núñez-Núñez, Casado-	2020	24 months	Spain- Clinico San Cecilio University Hospital	Hospital staff involved in any type of serious clinical incidents that caused or could have been caused (death or	To provide support to patients and their families, to HCWs and the healthcare institution after serious events, and to investigate and develop improvement	**i) Programme activations:** From 25 activations, 23 severe adverse events were investigated;<br>**ii) Frequency of the peer support encounters:** 1 to 17 HCWs per adverse event received trained peer support;

For peer review only

Fernández, Bueno-Cavanillas (2022)				serious harm of a patient)	actions, based on Susan's Scott model.	**iii) Time from the event until the activation of the programme:** ranged from 12h to one week;<br>**iv) Number of second victims identified:** 34.8% (n=47) were identified as second victims;<br>**v) Referral for third level of support** : 7 cases (14.9%) were referred, however 4 refused it;<br>**vi) Participants experience after participating in the programme:**increased sense of safety in the workplace; participants valued the interview provided in the second level of support and being able to contribute for the improvement actions after the adverse event occurrence;<br>**vii)Feedback for programme improvement:** The programme must be disseminated for all HCWs to have access to it; leaders should be trained to be more aware of the problem; first level of approach is identified has a problem in the units.
Hinzmann, Forster, Koll-Krüsmann, Schießl , Schneider, Sigl-Erkel et al (2022)	2020	24 months	Germany- health and emergency services	Clinicians and managers from the health and emergency services	To stabilize and restore the ability to act in highly stressful situation including adverse events, preserving clinical teams health and work capacity – in case of need , to transfer HCWs and managers with for specific treatment , initial structures of standard psychotherapeutic care in a low-threshold and timely manner.	**i) Evaluation of the programme:** In 81.4% of the cases the programme provided strong and very strong support to the callers in coping with the burden after a stressful event;<br>**ii) Follow up call after the first call :** 52.9% of callers needed a follow-up appointment with the same supporter after the first call;<br>**iii) Qualitative feedback after the programme** : there was an increased understanding of one's own reactions; openness to listen and understand that emotions are welcomed in a safe environment; knowing where to turn for help; enhanced mutual understanding and peer validation, as well as self-awareness and self-reflexion; leadership involvement increased.

For peer review only

Supplementary Table 4 – Main findings of the study according with the acronym OPERA

Main outcomes of interest	Main findings of the study
**O**rganisational factors<br>**P**eople<br>**E**nvironment<br>**R**ecommendations from previous studies on an organisational level<br>**A**ttributes of the interventions	Dissemination of the programme should be prioritised.
	Communication processes should be facilitated between HCWs and supporters.
	Specific training to peer supporters should be provided.
	Communication between the support team members should be regularly maintained.
	It's essential to create a multidisciplinary team with empathic skills.
	Leadership members should be actively engaged in both implementation and development of the programmes.
	A supportive and open organisational culture will benefit the programme implementation.
	To provide protected time and relief in staffing to participate in the programme (either for the programme's implementation team and for HCWs/SV that seek support) it's important for programme's sustainability.
	Evaluation of the support programme should be regular and overt time.
	Funding is important for programme sustainability.
	Programmes should be formally recognised in the instituition and have designated structures.
	Programmes should be easily accessible and on voluntary basis.
	Confidentiality should be ensured to facilitate HCWs' adherence and overcome potential barriers to participate in the programme.
	Programme's characteristics should be align with HCWs' needs.

Preferred Reporting Items for Systematic reviews and Meta-Analyses extension for Scoping Reviews (PRISMA-ScR) Checklist

SECTION	ITEM	PRISMA-ScR CHECKLIST ITEM	REPORTED ON PAGE #
**TITLE**			
Title	1	Identify the report as a scoping review.	1
**ABSTRACT**			
Structured summary	2	Provide a structured summary that includes (as applicable): background, objectives, eligibility criteria, sources of evidence, charting methods, results, and conclusions that relate to the review questions and objectives.	2
**INTRODUCTION**			
Rationale	3	Describe the rationale for the review in the context of what is already known. Explain why the review questions/objectives lend themselves to a scoping review approach.	4
Objectives	4	Provide an explicit statement of the questions and objectives being addressed with reference to their key elements (e.g., population or participants, concepts, and context) or other relevant key elements used to conceptualize the review questions and/or objectives.	4
**METHODS**			
Protocol and registration	5	Indicate whether a review protocol exists; state if and where it can be accessed (e.g., a Web address); and if available, provide registration information, including the registration number.	4
Eligibility criteria	6	Specify characteristics of the sources of evidence used as eligibility criteria (e.g., years considered, language, and publication status), and provide a rationale.	Pages 5,6, Complementary table 1
Information sources*	7	Describe all information sources in the search (e.g., databases with dates of coverage and contact with authors to identify additional sources), as well as the date the most recent search was executed.	Page 5,Complementary table 1
Search	8	Present the full electronic search strategy for at least 1 database, including any limits used, such that it could be repeated.	Complementary table 1
Selection of sources of evidence†	9	State the process for selecting sources of evidence (i.e., screening and eligibility) included in the scoping review.	5,6
Data charting process‡	10	Describe the methods of charting data from the included sources of evidence (e.g., calibrated forms or forms that have been tested by the team before their use, and whether data charting was done independently or in duplicate) and any processes for obtaining and confirming data from investigators.	6,7
Data items	11	List and define all variables for which data were sought and any assumptions and simplifications made.	6

For peer review only

SECTION	ITEM	PRISMA-ScR CHECKLIST ITEM	REPORTED ON PAGE #
Critical appraisal of individual sources of evidence§	12	If done, provide a rationale for conducting a critical appraisal of included sources of evidence; describe the methods used and how this information was used in any data synthesis (if appropriate).	6
Synthesis of results	13	Describe the methods of handling and summarizing the data that were charted.	7
**RESULTS**			
Selection of sources of evidence	14	Give numbers of sources of evidence screened, assessed for eligibility, and included in the review, with reasons for exclusions at each stage, ideally using a flow diagram.	6,7
Characteristics of sources of evidence	15	For each source of evidence, present characteristics for which data were charted and provide the citations.	7
Critical appraisal within sources of evidence	16	If done, present data on critical appraisal of included sources of evidence (see item 12).	Page 7 and Complementary table 2
Results of individual sources of evidence	17	For each included source of evidence, present the relevant data that were charted that relate to the review questions and objectives.	7- 13
Synthesis of results	18	Summarize and/or present the charting results as they relate to the review questions and objectives.	Pages 7, 9-13 Complementary tables 2 and 3 ;
**DISCUSSION**			
Summary of evidence	19	Summarize the main results (including an overview of concepts, themes, and types of evidence available), link to the review questions and objectives, and consider the relevance to key groups.	14,15,16
Limitations	20	Discuss the limitations of the scoping review process.	15,16
Conclusions	21	Provide a general interpretation of the results with respect to the review questions and objectives, as well as potential implications and/or next steps.	16
**FUNDING**			
Funding	22	Describe sources of funding for the included sources of evidence, as well as sources of funding for the scoping review. Describe the role of the funders of the scoping review.	16

JBI = Joanna Briggs Institute; PRISMA-ScR = Preferred Reporting Items for Systematic reviews and Meta-Analyses extension for Scoping Reviews.
* Where *sources of evidence* (see second footnote) are compiled from, such as bibliographic databases, social media platforms, and Web sites.
† A more inclusive/heterogeneous term used to account for the different types of evidence or data sources (e.g., quantitative and/or qualitative research, expert opinion, and policy documents) that may be eligible in a scoping review as opposed to only studies. This is not to be confused with *information sources* (see first footnote).
‡ The frameworks by Arksey and O'Malley (6) and Levac and colleagues (7) and the JBI guidance (4, 5) refer to the process of data extraction in a scoping review as data charting.
§ The process of systematically examining research evidence to assess its validity, results, and relevance before using it to inform a decision. This term is used for items 12 and 19 instead of "risk of bias" (which is more applicable to systematic reviews of interventions) to include and acknowledge the various sources of evidence that may be used in a scoping review (e.g., quantitative and/or qualitative research, expert opinion, and policy document).

From: Tricco AC, Lillie E, Zarin W, O'Brien KK, Colquhoun H, Levac D, et al. PRISMA Extension for Scoping Reviews (PRISMAScR): Checklist and Explanation. Ann Intern Med. 2018;169:467–473. doi: 10.7326/M18-0850.

For peer review only - http://bmjopen.bmj.com/site/about/guidelines.xhtml

BMJ Open

Key factors for effective implementation of healthcare workers support interventions after patient safety incidents in health organisations - a scoping review

Journal:	*BMJ Open*
Manuscript ID	bmjopen-2023-078118.R2
Article Type:	Original research
Date Submitted by the Author:	29-Nov-2023
Complete List of Authors:	Guerra-Paiva, Sofia; NOVA University Lisbon NOVA National School of Public Health, Public Health Research Centre, Comprehensive Health Research Center, CHRC<br>Lobão, Maria João; NOVA University Lisbon NOVA National School of Public Health, Public Health Research Centre, Comprehensive Health Research Center, CHRC; Hospital de Cascais Dr Jose de Almeida, Internal Medicine Department,<br>Simões , Diogo ; NOVA University Lisbon NOVA National School of Public Health, Public Health Research Centre, Comprehensive Health Research Center, CHRC; Public Health Unit of ACES Almada-Seixal<br>Fernandes, Joana; NOVA University Lisbon NOVA National School of Public Health, Public Health Research Centre, Comprehensive Health Research Center, CHRC<br>Donato, Helena; Centro Hospitalar e Universitário de Coimbra, Coimbra – Portugal, Documentation and Scientific Information Service<br>Carrillo, Irene; Miguel Hernandez University of Elche, Health Psychology; FISABIO,<br>Mira, José ; Miguel Hernandez University of Elche, Health Psychology; Salud Alicante-Sant Joan Health District,<br>Sousa, Paulo; NOVA University Lisbon NOVA National School of Public Health, Public Health Research Centre, Comprehensive Health Research Center, CHRC
**Primary Subject Heading**:	Public health
Secondary Subject Heading:	Health services research, Health policy
Keywords:	PUBLIC HEALTH, HEALTH SERVICES ADMINISTRATION & MANAGEMENT, Quality in health care < HEALTH SERVICES ADMINISTRATION & MANAGEMENT, Health & safety < HEALTH SERVICES ADMINISTRATION & MANAGEMENT

SCHOLARONE™
Manuscripts

I, the Submitting Author has the right to grant and does grant on behalf of all authors of the Work (as defined in the below author licence), an exclusive licence and/or a non-exclusive licence for contributions from authors who are: i) UK Crown employees; ii) where BMJ has agreed a CC-BY licence shall apply, and/or iii) in accordance with the terms applicable for US Federal Government officers or employees acting as part of their official duties; on a worldwide, perpetual, irrevocable, royalty-free basis to BMJ Publishing Group Ltd ("BMJ") its licensees and where the relevant Journal is co-owned by BMJ to the co-owners of the Journal, to publish the Work in this journal and any other BMJ products and to exploit all rights, as set out in our licence.

The Submitting Author accepts and understands that any supply made under these terms is made by BMJ to the Submitting Author unless you are acting as an employee on behalf of your employer or a postgraduate student of an affiliated institution which is paying any applicable article publishing charge ("APC") for Open Access articles. Where the Submitting Author wishes to make the Work available on an Open Access basis (and intends to pay the relevant APC), the terms of reuse of such Open Access shall be governed by a Creative Commons licence – details of these licences and which Creative Commons licence will apply to this Work are set out in our licence referred to above.

Other than as permitted in any relevant BMJ Author's Self Archiving Policies, I confirm this Work has not been accepted for publication elsewhere, is not being considered for publication elsewhere and does not duplicate material already published. I confirm all authors consent to publication of this Work and authorise the granting of this licence.

For peer review only

Key factors for effective implementation of healthcare workers support interventions after patient safety incidents in health organisations - a scoping review

Sofia Guerra-Paiva[a], Maria João Lobão[a,b], Diogo Godinho Simões[a,c], Joana Fernandes[a], Helena Donato[d], Irene Carrillo[e], José Joaquin Mira[e,f], Paulo Sousa[a]

Corresponding author:

Sofia Guerra-Paiva[a]

https://orcid.org/0000-0002-4311-3799

Avenida Padre Cruz 1600-560 Lisboa

sg.paiva@ensp.unl.pt

a. NOVA National School of Public Health, Public Health Research Centre, Comprehensive Health Research Center, CHRC, NOVA University Lisbon, Lisbon, Portugal
b. Internal Medicine Department, Hospital de Cascais, Cascais, Portugal
c. Public Health Unit of ACES Almada-Seixal, Almada, Portugal
d. Documentation and Scientific Information Service, Centro Hospitalar e Universitário de Coimbra, Coimbra, Portugal
e. Department of Health Psychology, Universidad Miguel Hernández de Elche
f. Alicante-Sant Joan Healthcare District, 03013 Alacant, Spain

Word count: 6212

For peer review only

ABSTRACT:

Objectives: To map and frame the main factors present in support interventions successfully implemented in health organisations in order to provide timely and adequate response to healthcare workers (HCW) after patient safety incidents (PSIs).

Design: Scoping review guided by the six-stage approach proposed by Arksey and O'Malley and by PRISMA-ScR.

Data sources: CINAHL, Cochrane Library, Embase, Epistemonikos, PsycINFO, PubMed, SciELO Citation Index, Scopus, Web of Science Core Collection, reference lists of the eligible articles, websites and a consultation group.

Eligibility criteria for selecting studies: Empirical studies (original articles) were prioritised. We used the Mixed Methods Appraisal Tool (MMAT) Version 2018 to conduct a quality assessment of the eligible studies.

Data extraction and synthesis: A total of 9,766 records were retrieved (last update in November 2022). We assessed 156 articles for eligibility in the full-text screening. Of these, 29 articles met the eligibility criteria. The articles were independently screened by two authors. In the case of disagreement, a third author was involved. The collected data was organized according to the acronym OPERA (Organisational factors, People, Environment, Recommendations from other studies, Attributes of the support interventions). We used Endnote to import articles from the databases and Rayyan to support the screening of titles and abstracts.

Results: We found that the existence of multidisciplinary teams, leadership engagement, an organisational culture based on principles of trust and non-judgement and strong dissemination of the support programmes' were crucial factors for the effectiveness of its implementation. Training should be provided for peer supporters and leaders to facilitate the response to HCW needs. Regular communication among the implementation team, existence of protected time, funding and continuous monitoring are useful elements to the sustainability of the programme.

Conclusion: HCWs' well-being depends on an adequate implementation process, ensuring a complex group of interrelated factors to support them after PSIs.

Registration number: 10.17605/OSF.IO/RQAT6.

Keywords: support programs; health personnel; second victim; clinical error; patient safety

only

Strengths and limitations of this study:

- The inclusion and exclusion criteria were defined in accordance with a preliminary search strategy, guided by the population, concept and context (PCC), as recommended by the JBI for scoping reviews;
- We didn’t restrict language and period of time to avoid having selection bias and compromise the validity and reliability of the findings;
- The data collection was limited to five interrelated dimensions (Organisational factors, People, Environment, Recommendations from other studies, Attributes of the support interventions);
- We used the MMAT assessment tool to evaluate the quality of the included studies, however some of the criteria couldn’t be fully applied in some specific cases;
- We included five experts from different countries to complement the literature search with additional sources of information;

For peer review only

For peer review only - http://bmjopen.bmj.com/site/about/guidelines.xhtml

INTRODUCTION

It's estimated that 10,4% to 50% of the professionals working in healthcare sector will experience at least once in their career the second victim phenomenon (SVP) (1,2) defined as "any healthcare worker (HCW), directly or indirectly involved in an unanticipated adverse patient event, unintentional healthcare error, or patient injury, and who becomes victimised in the sense that they are also negatively impacted" (3). These types of incidents, with an unintended or unexpected nature, can harm patients (first victims of an adverse event) or pose a risk to the system (near miss) (4,5).

HCWs play a crucial role in patient care and they can be seriously affected when a patient safety incident (PSI) happens. PSIs can impact HCWs' quality of life (2,6,7), in particular their physical and psychological well-being (8,9). A study published in 2020, shows that the most prevalent symptoms in HCWs after PSIs were troubling memories, anxiety/concern and anger toward themselves (9). Work satisfaction, confidence in their abilities (2) and work performance (7,10) can also be seriously impacted by these types of incidents. It can result in turnover intentions and absenteeism (11) and in the most severe cases can lead to suicide (12).

Institutional support systems are increasingly being implemented in order to provide an immediate and empathic response to HCWs after stressful situations such as PSIs. Health organisations are recognising the importance of this type of support, due to its important impact on the organisational culture (13), patient safety (PS) and quality of care (14–16) and also on the economic perspective (17). It is well-established that poor HCWs' well-being has a strong influence on the reoccurrence of PSIs (14). Therefore, prioritizing interventions that effectively support HCWs after stressful situations can prevent future healthcare incidents and improve PS.

The first reported support programmes were implemented in the United States in 2006 and since then, they have been gradually multiplying all over the world (18). In recent years there has been a growing number of publications describing the implementation of these types of programmes and practices with the overall aim of decreasing emotional and psychological distress in HCWs. A systematic review found that HCWs seek support not only after being involved in PSIs, but also when facing other distressing situations (e.g. emotional distress, torpid evolution of a patient, personal crises, intraoperative mishaps) (9). Based on the fact that there is still a lack of assistance to HCWs to cope in distressing situations, some support interventions are opening their scope of action (9).

Although support interventions have demonstrated their benefits and utility, there is still limited research on finding what the common elements present in the development and implementation process of successful interventions are. A toolkit was introduced in 2010 to provide guidance on the implementation of programmes to support HCWs that have been negatively impacted by PSIs (19). The development of this toolkit was an important step in assisting with the implementation of support programmes and it can be adjusted to any type of healthcare organisation (19). However, no study has been published focusing on reviewing the existing evidence to understand the main factors that contribute for an effective implementation of these types of support interventions.

Evidence shows that establishing a set of elements for implementing interventions does not ensure its effective introduction into daily usage (20,21). The success of interventions in health organisations highly depends on an adequate design, implementation and evaluation (22). One of the main aims of implementation science is to understand what are the factors that might affect the effectiveness and sustainability of the interventions and what is the necessary implementation process to produce the expected effects (20). In this sense, learning from previous experience can facilitate practical application and contribute to more effective interventions (23).

Study rationale:

In this study we set out **to map and frame the main factors that underlie an effective implementation of support interventions** in order to provide timely and adequate response to HCWs that are physically and/or emotionally affected by PSIs (known as second victims) or similar distressing situations. We have defined five interrelated dimensions guided by five main research questions, further described in this study, and we organised them in the acronym OPERA (Figure 1). This framework helped to inform the planning and design of the scoping review, as well as the execution. The defined five domains were inspired on the

health policy triangle (HPT) framework to guide effective implementation of health policies (24). However, HPT is a theoretical model and in order to overcome the research-to-practice gap, we have incorporated the implementation science principles and Donabedian's structure–process–outcome quality of care model, more recently adapted by Yano (25).

Objectives:

We aim to understand what existing organisational factors, relevant actors, contextual factors, operational attributes are present in interventions that were successfully implemented in health organisations to support HCWs after PSIs or other similar stressful events. We also aim to identify what are the recommendations from the included interventions for improving the effectiveness of the programmes implementation in health organisations.

METHODS

This scoping review is conducted using the six-stage approach proposed by Arksey and O'Malley (26) and is guided by the Preferred Reporting Items for Systematic Reviews and Meta-Analysis Extension for Scoping Reviews to ensure the transparency of the results obtained (27) and follows The Joanna Briggs Institute Methodology (JBI) for Scoping Reviews (28) .

All the methodological steps are described in further detail in the scoping review protocol published in a previous publication (29).

Stage 1 : Identifying the research question(s)

In this study we focus on the main research question:

- What are the key factors that contribute to an effective implementation of interventions to support HCWs after PSIs or other similar stressful situations in health organisations?

To answer the primary research question, five secondary questions were formulated based on the specific objectives and outcomes of interest of the study :

- What are the organisational factors that contribute to an effective implementation of these interventions?
- Who are the relevant actors that contribute to an effective implementation of these interventions?
- What are the contextual factors that contribute to an effective implementation of these interventions?
- What recommendations, as identified in previous studies, can be applied to effectively implement these interventions?
- What are the operational attributes that contribute to an effective implementation of these interventions?

Stage 2: Search strategy

A comprehensive search strategy using relevant electronic databases was developed with the support of a qualified research librarian. The search comprised Medical Subject Headings terms along with free-text keywords. We applied the search strategy in nine electronic databases and the last update was done in November 2022 (CINAHL, Cochrane Library, Embase, Epistemonikos, PsycINFO, PubMed, SciELO Citation Index, Scopus, Web of Science Core Collection). The applied search strategies in the electronic databases can be consulted in Supplementary Table 1. In addition to the database search, relevant websites were consulted and reference lists of the studies included in the full-text screening were screened to identify any other potential articles to include.

For peer review only - http://bmjopen.bmj.com/site/about/guidelines.xhtml

Stage 3: Study selection

We used Endnote to import articles from the different databases and we used Rayyan as a tool to facilitate the screening of titles and abstracts. The articles were independently and manually screened by two authors between April 2022 and February 2023. In the case of disagreement on article inclusion, a third author was involved to evaluate the paper independently and contribute to making a final decision.

We did not restrict the period of time or language of the included studies in order to reduce the selection bias and to undertake a comprehensive overview of the existing literature on a topic with still limited number of publications. Empirical studies (original articles) were prioritised along with systematic reviews and meta-analyses for collecting potential eligible studies. Grey literature (theses and other documents) was also considered eligible for the study.

The inclusion and exclusion criteria were defined in accordance with a preliminary search strategy, guided by the population, concept and context (PCC) framework (recommended by the JBI for scoping reviews (28)) and are further described in the published protocol of this study (29).

Based on the PCC framework, we defined the following criteria:

Population: Support interventions in health organisations in which HCWs are physically and/or emotionally affected by PSIs and other distressing situations. We considered support interventions destinated to health professionals, residents, and other allied health professionals (such as technicians and supply workers).

Concept: Support interventions that were fully implemented and executed in health organisations and provided measurable results that assessed the achievement of desired outcomes.

Context: Support interventions from a variety of healthcare contexts, including those in high-income, middle-income and low-income countries (e.g. primary care, urgent and acute care , ambulatory services, long-term facilities).

Exclusion criteria

Editorials, letters to the editor, case series, case reports, narrative reviews and commentaries were excluded.

Stage 4: charting the data

A data extraction template was created to show the characteristics of the eligible studies (detailed information can be consulted in Supplementary Table 2).

Quality assessment

We used the Mixed Methods Appraisal Tool (MMAT) Version 2018 to conduct a quality assessment of the eligible studies (30). We believe that this appraisal will be important to enhance the quality and rigor of our study, ensuring greater transparency and validity of the data. The eligible studies were evaluated by two independent reviewers. A third reviewer was involved in cases of disagreement in the quality assessment.

Stage 5: collating, summarizing and reporting the results

The information from the eligible studies was collected and organised into different conceptual categories, as presented in the acronym OPERA (figure 1):

<u>Organisational factors:</u>

- *organisational structures* (e.g. infrastructures, resources, tools, equipment, units and staffing levels functional for managing and delivering services, leadership structure/authority and organisational culture);

- *organisational processes* (e.g. organisational actions, procedures, recruitment criteria, training, programme implementation, communication processes, quality of interactions and coordination during programme implementation and dissemination as well as the sustainability of the practice);

- *organisational outcomes* (e.g. implementation measures, process quality measures, utilisation measures, effectiveness measures that assess the attainment of an end state);

People - Relevant actors (individuals and organisations that actively participate in the development and implementation of the programme)

Environment - Contextual factors (type of healthcare setting and cultural context).

Recommendations described in the included studies (recommendations to improve the implementation process of the support interventions).

Operational attributes of the interventions (format/type of programme, accessibility, usability and confidentiality of the programme/intervention).

Stage 6: consultation exercise and stakeholder involvement

We invited a group of five experts working on SVP research from five different countries (Finland, Germany, Italy, Portugal and Spain) to complement the literature search with additional sources of information. All of them are members of The European Researchers' Network Working on Second Victims (ERNST).

Patient and public involvement and engagement

None

RESULTS:

A total of 9,708 records were retrieved from nine electronic databases, 43 articles were retrieved from the reference lists of the included articles, 11 from websites and 4 were collected from stakeholders' group inputs.

Based on the screening of titles and abstracts, 7,262 articles were excluded and 13 articles could not be retrieved after trying to contact the authors. A total of 156 articles were assessed for eligibility.

A third independent author was involved to solve 4 conflicts in authors' decision, leading to the inclusion of one article. In total, 127 articles were excluded after the screening, and 29 articles ultimately met the eligibility criteria. Detailed information about the data collection, screening process, duplicates removed and reasons for exclusion is exhibited in the flow chart (Supplementary Figure 1), in line with the original PRISMA statement (31).

Studies with levels of evidence II and VI (32) met the eligibility criteria. We have included the following types of studies: mixed methods (n = 15); quantitative descriptive (n = 8); qualitative (n = 3); randomised controlled trial (n = 2) (for further details about the included studies consult Supplementary Table 2). Bearing in mind that we only included empirical studies, we didn't include the first two screening questions in the MMAT evaluation (optional for MMAT) (30).

The characteristics of the included studies are outlined in Table 1.

Table 1- Characteristics of the included studies

Categories	Subcategories	Number of articles	Total
Type of	Level II - Evidence obtained from Randomised Controlled Trial	3	29

For peer review only - http://bmjopen.bmj.com/site/about/guidelines.xhtml

**scientific article** [1]	Level VI - Evidence from a single descriptive or qualitative study	26	
**Type of Study design**	Mixed Method	15	29
	Quantitative descriptive	8	
	Qualitative	3	
	Randomised Controlled Trial	3	
**Country where the study was developed**	Denmark	1	29
	Germany	3	
	New Zealand	1	
	Spain	2	
	Sweden	1	
	UK	2	
	United States	19	

1.This rating scale is based on Ackley, B. J., Swan, B. A., Ladwig, G., & Tucker, S. (2008). *Evidence-based nursing care guidelines: Medical-surgical interventions.* (p. 7). St. Louis, MO: Mosby Elsevier (32).

Most of the programmes included in this study have a multidisciplinary application and were focused on supporting HCWs after traumatic work experiences directly associated with PSIs. Several programmes were particularly focused on responding to severe adverse events (33–35).

Although one-to-one sessions were the most commonly provided support, some programmes also included group sessions. We also included interventions focused on raising awareness of SVP and creating a supportive and proactive culture to manage critical incidents and enhance HCWs' well-being.

The included interventions are described in Supplementary Table 3.

In the following section we present the results based on the organisation of the acronym OPERA: Organisational factors (structure, process and outcome), People, Environment, Recommendations from the included studies and Attributes of the support programmes.

(Organisational factors)PERA

a) Organisational factors - Structure

Resources:

We found four main types of useful resources used in the interventions according to different applications. (36)These resources are described in Table 2.

Table 2: Resources identified in the included HCWs' support programmes

**Marketing and dissemination materials**	Print marketing materials: posters (37,38); handouts such as brochures and flyers (39–41) identification badge of peer support for easy recognition and quick reference cards (39) .<br><br>Digital marketing material: promotional videos (42) website (40,42,43) ; emailbox (43–45).
**Selfcare and wellbeing related resources**	Packets with aromatherapy (36,46); chocolate (35,36,47); snacks (48,49); kind messages (35,36,48); self-care pockets with essential support resources and guidance for coping with normal grief responses (36), others contain a journal, a stress ball, and tissues (47); general mental and emotional wellness advices (50), and use existing resources (50).
**Functional resources for programme implementation**	Electronic mailbox (36,39,40,43–45,51–54);access to virtual zoom (43,44) and WebEx platform (43); dedicated mobile phone/pager/hotline/phone call system for peer supporter sessions (35,37,45,54–57); web-based collaborative administrative platform for sharing information and managing the program (40) such as Sharepoint (39,51); checklist of responsibilities for the development team (36); list of peer support schedules (41,58); peer encounter forms (45,51) secure database of outreach attempts (54).

**Educational resources (most of them are related with peer support training)**	Online training focused on psychological first aid (33,45) PowerPoint presentations with voice narrations (33) training scenarios (47,56,59); videos (33,40,60); "Do's" and "Don'ts" list, self-affirmations resources (54,60) and specific facilitator's guide (43) made available to peer supporters for guidance during the encounters with SV/HCWs; tutorial for peer support facilitation (49).

Posters, brochures and flyers were the most widely used marketing resources. The most recent studies have an investment in administrative support resources such as the use of SharePoint, a collaborative platform for programme management (39,40,51).

Infrastructures:

The acquisition of materials and human resources was, in most of the cases, on a voluntary basis. However, some studies mentioned that the intervention received specific funding for acquiring resources (36,46).

The existence of a specific room for sharing information and emotions in privacy was referred to by two studies (35,58).

Organisational culture

We identified the following factors associated with the organisational culture that are facilitators of the implementation of HCW support programmes after PSIs:

- openness of the health organisation to innovation (41);
- implementation of previous initiatives that have contributed to the creation of a proactive organisational culture to manage PSIs (37) and to support HCWs after PSIs /other stressful situations and promote their well-being (36,44,46,52);
- the existence of formalised structures directed at fostering a PS culture, based on a just culture approach (35,36,52), and at supporting HCWs and enhancing their well-being (35,51,52,59)
- active involvement of leadership members in initiatives that support SVs and HCWs' well-being (35,40,41,43,45,46,52,54);
- existence of established policies promoting a supportive organisational culture (such as the application of paid time off after a critical incident occurs) (35) and organisational accountability for employees' support and well-being after PSIs (50).

We also identified some potential organisational barriers to the implementation of programmes:

- a lack of staff and leadership awareness regarding the support programmes for HCWs (42);
- the organisational culture doesn't prioritise PS and HCW support and doesn't disclose wellness problems (48,53,57).

b) Organisational factors – Process

Most of the implemented programmes had developed a needs assessment (40,45,49–51,57) and/or conducted a literature review (33,40,54) prior to the design and creation of the programme. The needs assessment makes it possible to adapt the interventions to the needs of the clinical teams and adjust them to the institutional context and culture in accordance with the most recent literature.

The team was recruited using three different methods: direct nomination of the team members based on their ability to provide support in an empathic way (37,45,46,52,54); votes from the clinical team (36,54); and on a voluntary basis (40,45,57).

An advertising campaign for raising attention of all the staff that would benefit the programme and show how to activate the service was carried out in a large number of interventions (37,39–43,50).

We describe below some of the implemented communication strategies described in the included studies:

- Digital marketing: dissemination of the programme on computer screensavers (42) and digital communication through the institutional website (41–43).

- Internal communication: hospital magazine, newsletters or email (42,44,45,45).

- Networking: presentation of programme in divisional meetings (40,42,45,52,53,58) or in hospital-wide events and conferences (47,58).

- Involvement of the leadership members in the dissemination process (42,48) and some programmes have included unit-level champions (42).

- Previous staff training on the topic of SVP (40,42) including training for staff provides the first level of support after a PSI in the local (56).

Dissemination was also carried out for recruiting peer supporters to join the peer support programmes (40,51,52,54). Most of the peer supporters received specific training to prepare them for providing assistance to others (36,37,39–42,45,46,48,49,51–54,56,61).

Sustainability of the programmes:

After implementing the pilot intervention, several projects have effectively expanded the pilot intervention to other departments (51), other healthcare facilities (38,40) or hospital wide (39,50). The full integration of the programme in the departments underlies the inclusion of the programme in the scheduled activities and in the available services of the institution (56).

Leadership support was an important factor for the implementation of the programme and its sustainability (37,42,45). Other programmes nominated unit champions to ensure the implementation of the programme and its sustainability by promoting a more supportive culture within the unit (39,53,57).

Regular meetings were found to be important to maintain the cohesion of the team over time (37,46,47,56). Annual courses and the implementation of an interactive virtual platform were important for the expansion of the workforce working in these programmes (37,40). The high level of motivation and interest of the team (54) and retention of peer supporters were given particular consideration for programme sustainability and this was associated with work meaningfulness, staff satisfaction, commitment, a high level of resilience and a high level of confidence as a peer supporter (42).

Some of the programmes were implemented in healthcare organisations where some PS initiatives and "culture-shifting interventions" had already taken place (39,41,42,46). In other cases they were integrated in major projects developed by the organisations (47,52,55). Both situations were considered potential facilitators for maintaining the programmes over time.

Funding was also an important aspect to consider for the sustainability of several programmes (38,43,46,52,53).

c) Organisational factors - Outcomes

Most of the studies included in the analysis focused on collecting outcomes related with programme's utilisation and the evaluation conducted by both peer supporters and users (HCWs/second victims that attended to the programmes).

In the Table 3 we describe in further detail the outcomes evaluated in the included studies.

Table 3: Collected outcomes from the included interventions

**Outcomes related to support services utilisation**	Frequency of the HCWs who attended the programme (36,37,52,55,60); frequency of programme activation (34,37,39,40,45–48,50–54,56); average duration of the encounters (56); number of programme dropouts (33); median number of interactions per month (53,56,57); frequency of peer support encounters (42,57); number of HCWs that need external support (56).
**Evaluation of the programme by the peer supporters perspective**	Overall peer support satisfaction with the training (33,34,45,50,60); perception of acquired knowledge, meaningfulness, motivation and interest to learn more and apply the learning (59); satisfaction about how encounter end out (40); need for additional training and experience (40); feeling able to provide support and being comfortable with their knowledge and skills as a peer supporter (40).

**Evaluation of the programme by the user perspective (HCWs involved in PSIs/SV)**	Overall satisfaction with the programme (33,36–38,40,46,48–51,58,60,61); knowledge/skills acquisition (33,44); usefulness of the contents (33); timeliness of the program (54); perceived helpfulness of the program (43,57); HCWs awareness of SVP phenomenon (41); qualitative experience after attending the programme (how much HCWs benefit from the programme) (33,36–38,40,46,48–51,58,60,61).
**Health-related outcomes**	Psychological and physical distress (45); emotional distress (43); perceived stress (55,59) anxiety and burnout (49,61); assessment of quality of life (36); perceptions of individual coping skills such us emotion regulation, self-efficacy and resilience (41,45,59,60).
**Work-related outcomes**	Job satisfaction (36); turnover intention and absenteeism (45); return to work (35); confidence in coping with adverse events (60).

O(People)ERA- Relevant actors

The establishment of a multidisciplinary team for the development and implementation of the support programmes was common to all the programmes. This team was predominantly composed by leadership members (hospital administrators and unit leaders), front-line workers, academics and experts in quality and safety (33,34,42,51,53,58). In some cases, it also included chaplains (58), social workers (51,58) and legal department members (53).

Most of the programmes' development and implementation were dependent on volunteer efforts. However, some programmes hired specific elements of the team, such as the programme directors and coordination members (41,52,55,56). Several studies highlighted the importance of these members in the programme activation process, particularly in matching the profile of peer supporters with HCWs' needs and in the contact with peer supporters and outreach people in need of support (47,52–54,57). One programme included contract freelancer work by psychotherapists to provide more specialised support (55) and other programmes remunerated peer supporters for their work (46,50,55).

Trained peer supporters are crucial for providing effective support for HCWs involved in PSIs. Specialised trainers from different types of backgrounds (such as psychology, nursing, quality improvement and PS, workplace wellness, legal services, executive sponsors, department representatives) provided workshops and seminars for peer supporters (36,51,54). In some cases, there was a specialist to facilitate monthly debriefing meetings for peer supporters to process their experiences and to receive assistance (36,46,51).

Most of the programmes also provided access to specialised external support that represents the third level of support in the case of programmes that follow the Scott Three-Tier Model (34,36,40,48,56). In other types of programmes, complementary support was provided by chaplains, social workers or Employee Assistance Programme (EAP) counsellors (35,40,51,58).

On a department level, unit leaders performed different types of essential functions by contributing to the development of the programme (46,54), participating in the recruitment of peer supporters (33,42,48,51), providing first-level support for HCWs in need (34), coordinating programme's components and mentoring peer support team members within the facility (56).

OP(Environment)RA – contextual factors

On an internal level, we found that most of the programmes were implemented in large and academic hospitals, characterised by an environment with multiple and complex divisions (34,35,37,39–41,43,45,52,53,56), and with a high level of specialisation (tertiary and quaternary care) (44,46,48,50,54). Some programmes were specifically implemented in stressful and busy environments, such as emergency medicine departments, intensive care units and psychiatric departments (39,41,48,49,59).

We found that in some cases the organisational environment was beneficial to the implementation of the programme, particularly when healthcare organisations were already working towards creating a more supportive environment for their staff and strengthening the safety culture (36,46,47,51,52,55).

We also found that previous occurrence of a very serious adverse event helped in recognizing the need to implement a programme to support staff in supporting them to cope after PSIs (35,42).

For peer review only - http://bmjopen.bmj.com/site/about/guidelines.xhtml

On an external level, several studies have mentioned that programme implementation was affected by the Covid-19 pandemic, such as the possibility of handling face-to-face encounters, and it also affected the data collection/monitoring process of the interventions (6,8,9,11,22,24,27).

OPE(Recommendations)A - Recommendations related with the implementation process and directly related with HCWs experience.

Different types of recommendations for improving the programmes were mentioned in the included studies with the ultimate goal of achieving a more effective intervention. They were identified from the user perspective (HCWs in need of support after being involved in a PSI) and also from the perspective of the implementation process (described in Table 4).

Table 4: Main recommendations referred to in the included studies from both the user and the implementation process perspectives

Recommendations related with the implementation process	Recommendations directly related with users' experience (Healthcare workers involved in PSIs/SV)
**Conditions to facilitate implementation process:**	**Conditions to facilitate users experience:**
To allocate protected time for teams to implement the programme and actively participate in the tasks and training (34,40);	To allocate protected time for HCWs to attend the programme's activities and the support sessions (38,49,60);
Administrative framework should be ensured to support programme implementation (56);	Participation in the process should be entirely voluntary and confidential (34,37,40,49,53,56,57,60);
To develop an institutional policy to guide the management of the critical event and support the affected HCWs and patients (35);	To invest in creating an organisational culture that addresses and acknowledges clinicians' vulnerability, while promoting a supportive environment after stressful incidents (52,56);
Funding was an important facilitator for program development and implementation (39,43,46);	To involve legal and risk management departments to address concerns about confidentiality or related issues (34,35,53);
To be formally recognised as an institutional program (34);	To ensure that SV have an adequate access to the programme, feel safe and not stigmatised when accessing resources (48);
To invest in telehealth solutions to support HCWs in the workplace (43);	
**Procedures related with the implementation process:**	**Procedures focused on user's experience:**
To invest in programme's dissemination and marketing for increasing HCWs' adherence to the programme (34,39,43,45,48,50,54–56);	Appropriate timing for programme's activation (ideally it should be immediately available to the HCWs after a stressful event (56)) and adequate duration of the support (37,60);
To actively involve the target group in the development of the programme and to conduct a needs assessment helps fostering interest and adapt to the specific needs of the target population (49,61);	All HCWs involved in a critical incident should be contacted to receive support (37,53);
To integrate staff working in the unit in the programme's team, helps to understand the needs of the unit (46).	Active surveillance in the units should be done to identify potential SV (particularly in high risk environments) (56);
To promote active involvement of leadership members facilitates the implementation of the programmes and contributes for staff engagement (33,35,37,40,47–50,52,56);	To increase program awareness for front-line staff and prepare them how to give first level of support (45,48,51);
To promote training sessions and resources to increase managers awareness about the SVP and about the existing support programmes (47,48);	To train the leadership to support staff to cope with stressful situations and to direct them to support resources in case of need (34,36,46,47,60);
To create a multidisciplinary support team to facilitate a comprehensive programme's development and address different areas for support while leveraging a range of expertise (53);	To enhance the level of education on staff resiliency in the services (41);
To set regular debriefings (in person or virtual meetings) to exchange experiences and to foster a culture of mutual support among the members of the programme's team (37,47);	To establish an interdisciplinary support team to open the scope of support according to the different staff's needs and background (39,42,51);
Training should be provided to peer support training and role play is one of the most recommended formats (34,42,47);	To create a safe place for sessions (36,46,49,58);
To develop a list of key phrases that peer supporters can use in their interactions with SVs (42);	Smaller groups are preferred for sharing experiences and support (38);
To evaluate the impact of the programme and monitor its longer term effects and drive continuous improvement (42,43,45–47,50,51,59).	To make resources available and close to the staff, to make them easy to reach (48,52,58);
	To identify barriers and facilitators for HCW to seek mental health support (e.g. stigma, career concerns, protected time) (43);
	To provide channels to reach the programme even when the HCW was not involved in a PSI (54);

To use pre-existing structures , resources and adapt existing programmes to facilitate the development and implementation of the programme(39,40,47,50);<br><br>Having an electronic dashboard for sharing documentation and data collection (39);	To provide a holistic support (e.g. incorporating integrative therapy techniques; cognitive based therapy) (46,59).

OPER(Attributes) - Operational attributes of the programmes

a) Accessibility:

The access of HCWs to the programmes was done by different channels to find the most convenient format for the users: phone call (37,39,42,51,55), email (39,44,51,59) or direct contact with peer supporters or with core team members (39,51,59).

Programmes can be activated by the following people:

- anyone that was involved in the stressful event (34,45,47,50,52,56);
- safety and risk management staff (47,53);
- peer supporters (53);
- nurse in charge (58);
- leadership members (34,35);
- programme directors (52–54).

In some cases, the entire clinical team is contacted by the implementation team or by leadership members with a view to integrating in the support programme after a PSI, however acceptance only depends on the individual choice of the HCWs (34,54,57,61).

In the case of programmes that have online resources, they could be accessed through a website (33,40,41).

Although most of the programmes were provided on a voluntary basis, some of them have mandatory activities for all staff in the departments, such as a seminar to promote a shared understanding of SVP and the need for peer support (50), or attendance at debriefing sessions to enhance the recovery of all staff in the unit (35).

Programmes based on the Scott Three-Tier Model establish the access to the programme according to different levels of HCW needs (34,37,56). The first level of support should be available immediately after the incident has happened and team members should be prepared to provide it (local-level support). For accessing the second level of support, anyone can activate peer support with a trained peer. For the third level of support, the HCW is referred to specialised support (56).

In some cases, programmes can be accessed seven days a week (39,41,43,45,51,55,56). In a support programme implemented in New Zealand, the phone number to reach the support team was added into the staff contact list and on-call phones within the unit to facilitate the activation process (37). The dissemination of schedules, timelines and contacts was a useful strategy employed to facilitate access to the programme (41,45).

Lack of staff awareness about the programme (42,48) and difficulty finding time to attend the interventions were the main barriers to accessing the programmes (38). Moreover, some HCWs resisted accessing support since they did not recognise the need for it or preferred to avoid dealing with the situation again by talking about it (35).

b) Usability :

For peer review only - http://bmjopen.bmj.com/site/about/guidelines.xhtml

To ensure the maximal attendance of staff, in several programmes the communication process was facilitated by peer supporters or the programme director after the activation of the programme. These actors have the responsibility of contacting the HCWs immediately after the incident to schedule a one-to-one peer support encounter (40,50,52–54,57). In several programmes, the schedule was negotiated with HCWs according to their needs (37,40,46,47,53,54,57), and in some cases the location (40,46) and format (in person, phone call or email) of the sessions were also negotiated (47,54).

In some cases, staffing relief at the workplace and protected time were provided to allow HCWs to attend the support programmes during their working time (40,44,46,49,58,61).

To facilitate initiation of the programme, a leadership member or the programme director matched the peer supporter profile with the HCWs' needs according to the different criteria, such as geographic proximity and the same medical speciality (40,47,54). In other situations, like in the Buddy Study Programme, the HCWs selected the peer supporter according to their preferences so they could contact if they needed support (50).

To facilitate the HCWs' participation in the programme, some programmes prioritised the virtual format (40,43,44,53) and limited the duration of sessions to 60 minutes (37,38,43,46,49).

A peer support intervention in Sweden defined the topics to approach in the sessions based on participants' suggestions and beliefs (61). This made it possible to attend to the needs of the participants and to promote their adherence to the interventions (61).

In online programmes like MISE we found that browsing, amount of time required to complete the activities and comprehension of the programme content were valued attributes to facilitate the usability of the programme (33).

DISCUSSION

In this study we focused on identifying the highest number of reported programmes and other initiatives to support HCWs after PSIs. The majority of the included programmes are based on peer support. These types of programmes are founded on social support with the ultimate goals of increasing professional quality of life, decreasing emotional stress and ensuring patients' safety (62–64).

We found that these types of programmes should be voluntary, with easy access and widely disseminated in healthcare organisations. This will make it possible to provide immediate psychological first aid after a distressful event and to overcome obstacles related to a lack of awareness of SVP and stigmatisation associated with HCW vulnerability (39,42,64–66).

The association between just culture and empathic and non-judgemental responses to PSIs has been clear (18,42,67,68). This is identified as a core condition for an open communication, and to establish positive relationships between peer supporters and HCWs, thereby enhancing programme adherence (68,69). Namely, willingness to give support with empathy, leadership skills, reliability, being communicative, and not being judgmental are essential elements for effective peer support (63). Another study pointed out that the ability to understand others' feelings and experiences after a PSI can be beneficial to the support process, by improving emotional regulation and reinforcing the cooperation between HCWs and peer supporters (70). In our study we have identified that all these principles were mentioned to improve the effectiveness of the interventions and, therefore it should be taken in consideration in peer supporters training when implementing the programme.

We found that specific training for peer supporters was provided in most of the programmes to prepare them to adequately provide psychological support according to HCWs' needs. Training is believed to be one of the key components to consider when implementing a support programme (39,42,64). According to implementation research, it's essential for an effective programme implementation (71).

Many of the included studies also mentioned the importance of setting regular multidisciplinary meetings to share important learning and experiences and to keep the team motivated over time. Rosak-Szyrocka (2015) points out that having a motivated team is very important to ensure their commitment to, and engagement in work (72). This study also points out that in hospitals, HCWs are

particularly motivated by strong interpersonal relations and a positive atmosphere, as they foster cooperation and mutual support among the team members (73). Another study indicates that multidisciplinary teamwork is an essential element for improving outcomes at an organisational level (74).

We found that active participation by leaders in the initiatives can influence the effectiveness of programmes implementation and their sustainability. Helping to create a safe and resilient environment can increase the programmes' acceptability among HCWs and their engagement in the activities (74–77). Leaders' participation was not only important in the implementation process, but also in the development of the programmes. In particular, it can contribute to adjust the programmes to the healthcare context, facilitate the acquisition of resources and recruitment of peer supporters.

We also found that leadership engagement in HCWs/SVs support initiatives is very important for strengthening the organisational culture towards a non-punitive response to error. Leadership members are essential actors in helping to create stimulating and supportive environments in healthcare among teams (73,78). Boguslavsky, Gutierrez- and Holschneider (75), refers the importance of having leaders with empathic and communication skills, that are able to listen, empower and encourage others, in line with non-blame culture principles. Therefore, the involvement of leaders with these types of skills will benefit the support programme's implementation.

Moreover, we also found that the success of the implementation is also dependent on how closely aligned it is with HCWs' needs. Therefore, HCWs should be consulted and involved in the programme development process. Our results corroborate Søvold et al.'s findings, which highlight the importance of HCWs participating more in the decision-making process as well as in the development, implementation, testing and evaluation of the interventions, with the ultimate aim of improving their health, well-being and job satisfaction (79). Interventions that include the target population's perspectives from the first steps of development exhibit a higher level of adherence and adoption and are more sustainable over time (71).

However, HCWs are very often overloaded with duties that could be undertaken by other staff, and prevented from performing other necessary tasks that need their qualifications, with few opportunities to apply for training, develop their professionals skills and be available to participate in workplace initiatives (74). This contributes to less work satisfaction and more costs for the system (74). We found that working conditions are one of the main pillars for ensuring effective implementation and its sustainability. Providing protected time and staffing relief were identified as two of the main priorities for ensuring that, on the one hand, qualified HCWs could participate in the development and implementation of support interventions, and on the other, HCWs would be able to attend the support programmes if they needed support. Financial incentives play also an important role in keeping the support teams over time and to facilitate the programme implementation. Some evidence corroborates these findings, namely in what concerns to HCWs retention (71,74).

Finally, it is agreed that monitoring programme outcomes over time is essential for assessing programme effectiveness, and evaluating its progress and impact on HCWs/SVs and in health organisations (80). This process should be continuous and facilitates the ongoing improvement of the programmes (22). However, there are a limited number of studies that follow up interventions over time. According to Wade et al. (2022), it's still not clear how much time would be necessary to monitor the impact of programmes on HCWs' skills and knowledge (81). Thus, we recommend that future programmes invest in monitoring their results over time and for longer periods.

In hospital settings, we have found that programmes frequently monitor before and after interventions, by collecting both qualitative and quantitative data. It's agreed that monitoring process should follow rigorous and feasible when assessing both types of measures (80).

Although hospital settings are increasingly investing in the monitoring process, the study of the impact of HCWs' support programmes it's still limited and unreported in non-hospital settings (82). This might be due to the insufficient emphasis on safety culture in non-hospital settings (82,83), which could be improved by increasing PS initiatives and awareness campaigns in these contexts.

For peer review only - http://bmjopen.bmj.com/site/about/guidelines.xhtml

The experience from support programmes directly and not directly associated with supporting HCWs after PSIs was particularly useful to identify barriers and challenges in the access and adherence to the programmes and sustainability concerns. One of the main topics of discussion is focused on programmes' confidentiality (42,84–86). Wade et al. (2022) highlighted the need to establish a consensus and build a body of evidence to evaluate these types of programmes in an ethical and confidential way that involves protecting the privacy and confidentiality of the attendees of these types of programmes (81). We also highlight that the legal framework of each country can influence the success of the programmes' implementation. Professional liability is often not cited in articles describing interventions, despite its influence on the programmes and its impact on transitioning from a reactive safety culture to a generative safety culture (87).

Ultimately, we found that these types of programmes should be formalised and have defined structures to facilitate its sustainability and to overcome potential institutional barriers to the implementation of the programmes. Examples like RISE programme have demonstrated that the formal recognition of the support programme in all the large academic medical centre , the use of existing structures and involvement institutional stakeholders have not only strengthen its visibility within the institution, but also inspired other external health settings to implement their own support programmes (42).

In summary, we have organised the main findings in Supplementary Table 4, organised according to the acronym OPERA.

LIMITATIONS

In this study, we have found that long-term evaluation of programmes it's still limited for the most part, and in some cases, evaluating the outcomes is not recognised as a priority for reasons of confidentiality and ethics. We recognise the need to strengthen the organisational culture towards a non-punitive response to error in order to overcome potential barriers to programme adherence and evaluation. It is very important to increase the follow-up time to understand the impact of these types of programmes and their effectiveness in the long term.

We evaluate all the included studies using the MMAT quality assessment tool since this tool is suitable for different types of methodologies, however some of the criteria couldn't be applied in the descriptive studies. We suggest that this tool could be adapted in the future to these types of studies.

CONCLUSION

This is the first time that a study has focused on understanding the set of characteristics and elements necessary for a successful programme' implementation to respond to HCWs needs after PSIs, based on the fact that their success highly depends on an adequate implementation and evaluation process.

This study was inspired in the previous experience from other support programmes with the ultimate propose of guiding the implementation of HCWs support programmes in health organisations and contributing for future evidence-based practice.

In summary, we concluded that programmes should be easily accessible and voluntary for all HCWs in health organisations. Dissemination should be prioritised in order to give higher level of visibility to these programmes. The effectiveness of programmes' implementation is highly dependent on the organisational culture, the active involvement of leadership, and a multidisciplinary team. Training should be provided for both peer supporters and leadership members, to make it possible to respond to HCWs' needs in a more prepared and satisfactory way. Regular communication among support teams should be maintained over time to keep teams motivated and increase their retention. Moreover, it's recommended to allocate dedicated time and staffing resources to engage in these types of interventions. Establishing formalised structures and securing funding sources it's important for the programmes's sustainability. The use of existing resources can overcome potential institutional barriers. Finally, programmes should be monitored for their continuous improvement without compromising the confidentially of the data.

For peer review only - http://bmjopen.bmj.com/site/about/guidelines.xhtml

AKNOWLEGMENTS

This work was supported by ERNST – Cost Action 19113 (COST – European Cooperation in Science and Technology, www.cost.eu). We would like to particularly thank the members of stakeholder groups, namely Prof. Reinhard Strametz, Dr Susanna Tella and Prof. Massimiliano Panella, for their contribution to this study as members of the consultation group.

FUNDING

The present publication was funded by Fundação Ciência e Tecnologia, IP national support through CHRC (UIDP/04923/2020) and under the grant (UI/BD/150875/2021).

ETHICS AND DISSEMINATION

This scoping review does not require ethical approval. Results and conclusions of the study will be presented in scientific conferences as well as in international forums and through other relevant dissemination channels.

AUTHOR CONTRIBUTIONS

SGP was involved in the writing of the article. SGP, MJL, PS and JJM contributed to the conceptualisation of the study. SGP, MJL, DGS participated in the pilot testing stage and defined the inclusion criteria. SGP, MJL, JDS, JF and IC were involved in the screening process of the eligible studies. HD contributed to the definition of a robust search strategy and participated in the preliminary search in relevant bibliographic databases, particularly in peer-reviewed journals and grey literature databases. PS, JJM and IC contributed to the literature review. PS and JJM also participated in the consultation group. All authors read and approved the manuscript.

COMPETING INTERESTS

This protocol is part of the PhD project of the corresponding author (SGP), which has been previously approved by the National School of Public Health (ENSP-NOVA). Some of the research group members are part of the ERNST - COST Action 19113. All the authors have submitted the disclosure of interest form.

DATA SHARING STATEMENT

All data relevant to the study are included in the article or uploaded as supplementary information.

REFERENCES

1. Seys D, Wu AW, Gerven EV, Vleugels A, Euwema M, Panella M, et al. Health Care Professionals as Second Victims after Adverse Events: A Systematic Review. Eval Health Prof. 2013;36(2):135–62.
2. Waterman AD, Garbutt J, Hazel E, Dunagan WC, Levinson W, Fraser VJ, et al. The Emotional Impact of Medical Errors on Practicing Physicians in the United States and Canada. Jt Comm J Qual Patient Saf. 2007;33(8):467–76.
3. Vanhaecht K, Seys D, Russotto S, Strametz R, Mira J, Sigurgeirsdóttir S, et al. An Evidence and Consensus-Based Definition of Second Victim: A Strategic Topic in Healthcare Quality, Patient Safety, Person-Centeredness and Human Resource Management. Int J Environ Res Public Health. 2022;19:16869.
4. World Health Organization. Patient safety incident reporting and learning systems: technical report and guidance. Geneva: World Health Organization; 2020.
5. NHS England. Report a patient safety incident [Internet]. Redditch; 2022. Available from: https://www.england.nhs.uk/patient-safety/report-patient-safety-incident/
6. Rinaldi C, Leigheb F, Vanhaecht K, Donnarumma C, Panella M. Becoming a "second victim" in health care: Pathway of recovery after adverse event. Rev Calid Asist. 2016;31:11–9.

For peer review only

7. Vanhaecht K, Seys D, Schouten L, Bruyneel L, Coeckelberghs E, Panella M, et al. Duration of second victim symptoms in the aftermath of a patient safety incident and association with the level of patient harm: a cross-sectional study in the Netherlands. BMJ Open. 2019;9:e029923.
8. Strid EN, Wåhlin C, Ros A, Kvarnström S. Health care workers' experiences of workplace incidents that posed a risk of patient and worker injury: a critical incident technique analysis. BMC Health Serv Res. 2021;21(1):511.
9. Busch IM, Moretti F, Purgato M, Barbui C, Wu AW, Rimondini M. Psychological and Psychosomatic Symptoms of Second Victims of Adverse Events: a Systematic Review and Meta-Analysis. J Patient Saf. 2020;16(2):e61–74.
10. Carrillo I, Tella S, Strametz R, Vanhaecht K, Panella M, Guerra-Paiva S, et al. Studies on the second victim phenomenon and other related topics in the pan-European environment: the experiences of ERNST Consortium members. Journal of Patient Safety and Risk Management. 2022;27(2):59–65.
11. Burlison JD, Quillivan RR, Scott SD, Johnson S, Hoffman JM. The Effects of the Second Victim Phenomenon on Work-Related Outcomes: Connecting Self-Reported Caregiver Distress to Turnover Intentions and Absenteeism. J Patient Saf. 2021;17(3):195–9.
12. Scott SD, McCoig MM. Care at the point of impact: Insights into the second-victim experience. Am Soc Healthc Risk Manag Am Hosp Assoc. 2016;35(4):6–13.
13. Quillivan RR, Burlison JD, Browne EK, Scott SD, Hoffman JM. Patient Safety Culture and the Second Victim Phenomenon: Connecting Culture to Staff Distress in Nurses. Jt Comm J Qual Patient Saf. 2016;42(8):377-AP2.
14. Hall LH, Johnson J, Watt I, Tsipa A, O'Connor DB. Healthcare Staff Wellbeing, Burnout, and Patient Safety: A Systematic Review. Harris F, editor. PLOS ONE. 2016;11(7):e0159015.
15. Schiess C, Schwappach D, Schwendimann R, Vanhaecht K, Burgstaller M, Senn B. A Transactional 'Second-Victim' Model-Experiences of Affected Healthcare Professionals in Acute-Somatic Inpatient Settings: A Qualitative Metasynthesis. J Patient Saf. 2021;17(8):e1001–18.
16. Schwappach D, Boluarte T. The emotional impact of medical error involvement on physicians: A call for leadership and organizational accountability. Swiss Med Wkly. 2008;138(1–2):9–15.
17. Moran D, Wu AW, Connors C, Chappidi MR, Sreedhara SK, Selter JH, et al. Cost-Benefit Analysis of a Support Program for Nursing Staff. J Patient Saf. 2020;16(4):e250–4.
18. Busch IM, Moretti F, Campagna I, Benoni R, Tardivo S, Wu AW, et al. Promoting the Psychological Well-Being of Healthcare Providers Facing the Burden of Adverse Events: A Systematic Review of Second Victim Support Resourcesmix. Int J Environ Res Public Healt. 2021;18(10):5080.
19. Pratt S, Kenny L, Scott SD, Wu AW. How to develop a second victim support program: a toolkit for health care organizations. Jt Comm J Qual Patient Saf. 2012;38(5):235–40.
20. Bauer MS, Kirchner J. Implementation science: What is it and why should I care? Psychiatry Res. 2020;283:112376.
21. Thoele K, Ferren M, Moffat L, Keen A, Newhouse R. Development and use of a toolkit to facilitate implementation of an evidence-based intervention: a descriptive case study. Implement Sci Commun. 2020;1(1):86.
22. Herrera-Sánchez IM, León-Pérez JM, León-Rubio JM. Steps to Ensure a Successful Implementation of Occupational Health and Safety Interventions at an Organizational Level. Front Psychol. 2017;8:2135.
23. Cook S, Mayers S, Goggins K, Schlundt D, Bonnet K, Williams N, et al. Assessing research participant preferences for receiving study results. J Clin Transl Sci. 2019;4(3):243–9.
24. Buse K, Mays N, Walt G. The health policy framework:Context,process and actors. In: Making health policy. 1st edition. Maidenhead,Berkshire: McGraw- Hill Education:University Press; 2005.
25. Yano EM. The role of organizational research in implementing evidence-based practice: QUERI Series. Implement Sci. 2008;3(1):29.
26. Arksey H, O'Malley L. Scoping studies: towards a methodological framework. Int J Soc Res Methodol. 2005;8(1):19–32.
27. Tricco AC, Lillie E, Zarin W, O'Brien KK, Colquhoun H, Levac D, et al. PRISMA Extension for Scoping Reviews (PRISMA-ScR): Checklist and Explanation. Ann Intern Med. 2018;169(7):467–73.

For peer review only

28. The Joanna Briggs Institute. The Joanna Briggs Institute Reviewers' Manual 2015 Methodology for JBI Scoping Reviews. Australia: The Joanna Briggs Institute; 2015.
29. Guerra-Paiva S, Lobão MJ, Simões JD, Donato H, Carrillo I, Mira JJ, et al. Key factors for effective implementation of healthcare worker support interventions after patient safety incidents in health organisations: a protocol for a scoping review. BMJ Open. 2022;12(8):e061543.
30. Hong Q, Pluye P, Fàbregues S, Bartlett G, Boardman F, Cargo M, et al. Mixed Methods Appraisal Tool (MMAT), version 2018.Registration of Copyright (#1148552), Canadian Intellectual Property Office, Industry Canada.
31. Moher D, Liberati A, Tetzlaff J, Altman DG, for the PRISMA Group. Preferred reporting items for systematic reviews and meta-analyses: the PRISMA statement. BMJ. 2009;339:b2535.
32. Ackley B, Swan B, Ladwig G, Tucker S. Evidence-based nursing care guidelines: Medical-surgical interventions. St. Louis, MO: Mosby Elsevier; 2007.
33. Mira JJ, Carrillo I, Guilabert M, Lorenzo S, Pérez-Pérez P, Silvestre C, et al. The Second Victim Phenomenon After a Clinical Error: The Design and Evaluation of a Website to Reduce Caregivers' Emotional Responses After a Clinical Error. J Med Internet Res. 2017;19(6):e203.
34. Cobos-Vargas A, Pérez-Pérez P, Núñez-Núñez M, Casado-Fernández E, Bueno-Cavanillas A. Second Victim Support at the Core of Severe Adverse Event Investigation. Int J Environ Res Public Health. 2022;19(24):16850.
35. Roesler R, Ward D, Short M. Supporting Staff Recovery and Reintegration After a Critical Incident Resulting in Infant Death. Adv Neonatal Care. 2009;9(4):163–71.
36. Graham P, Zerbi G, Norcross W, Montross-Thomas L, Lobbestael L, Davidson J. Testing of A Caregiver Support Team. EXPLORE. 2019;15(1):19–26.
37. Civil NM, Hoskins JD. Building a critical incident peer response team: A full theatre team welfare intervention. Anaesth Intensive Care. 2022;50(6):421–9.
38. Allen D, Spencer, G, McEwan K, Catarino F, Evans R, Crooks S, et al. The Schwartz Centre Rounds: Supporting mental health workers with the emotional impact of their work. Int J Ment Health Nurs. 2020;29:942–52.
39. Merandi J, Liao N, Lewe D, Morvay S, Stewart B, Catt C, et al. Deployment of a Second Victim Peer Support Program: A Replication Study. Pediatr Qual Saf. 2017;2(4):e031.
40. Finney RE, Jacob A, Johnson J, Messner H, Pulos B, Sviggum H. Implementation of a Second Victim Peer Support Program in a Large Anesthesia Department. AANA. 2021;89(3):235–44.
41. Bryant R. Raising Awareness of Second Victim Phenomenon and SupportingYOU: A Quality and Safety Initiative. Graduate Theses, Dissertations, and Problem Reports. West Virginia: West Verginia University; 2022. 10169.
42. Edrees H, Connors C, Paine L, Norvell M, Taylor H, Wu AW. Implementing the RISE second victim support programme at the Johns Hopkins Hospital: a case study. BMJ Open. 2016;6(9):e011708.
43. Mellins CA, Mayer LES, Glasofer DR, Devlin MJ, Albano AM, Nash SS, et al. Supporting the well-being of health care providers during the COVID-19 pandemic: The CopeColumbia response. Gen Hosp Psychiatry. 2020;67:62–9.
44. Rubin EW, Rassman A. cAIR: Implementation of peer response support for frontline health care workers facing the COVID-19 pandemic. Soc Work Health Care. 2021;60(2):177–86.
45. Thompson M, Hunnicutt R, Broadhead M, Vining B, Aroke E. Implementation of a Certified Registered Nurse Anesthetist Second Victim Peer Support Program. J Perianesth Nurs. 2022;37(2):167–73.
46. Schuster MA. Creating the Hematology/Oncology/Stem Cell Transplant Advancing Resiliency Team: A Nurse-Led Support Program for Hematology/Oncology/Stem Cell Transplant Staff. J Pediatr Oncol Nurs. 2021;38(5):331–41.
47. Morales CL, Brown MM. Creating a Care for the Caregiver Program in a Ten-Hospital Health System. Crit Care Nurs Clin North Am. 2019;31(4):461–73.
48. Merandi J, Winning AM, Liao N, Rogers E, Lewe D, Gerhardt CA. Implementation of a second victim program in the neonatal intensive care unit: An interim analysis of employee satisfaction. J Patient Saf Risk Manag. 2018;23(6):231–8.
49. Calder-Sprackman S, Kumar T, Gerin-Lajoie C, Kilvert M, Sampsel K. Ice cream rounds: The adaptation, implementation, and evaluation of a peer-support wellness rounds in an emergency medicine resident training program. CJEM. 2018;20(5):777–80.

For peer review only - http://bmjopen.bmj.com/site/about/guidelines.xhtml

50. Schrøder K, Bovil T, Jørgensen JS, Abrahamsen C. Evaluation of'the Buddy Study', a peer support program for second victims in healthcare: a survey in two Danish hospital departments. BMC Health Serv Res. 2022;22(1):566.
51. Krzan KD, Merandi J, Morvay S, Mirtallo J. Implementation of a "second victim" program in a pediatric hospital. Am J Health Syst Pharm. 2015;72(7):563–7.
52. Shapiro J, Galowitz P. Peer Support for Clinicians: A Programmatic Approach. Acad Med. 2016;91(9):1200–4.
53. Lane MA, Newman BM, Taylor MZ, O'Neill M, Ghetti C, Woltman RM, et al. Supporting Clinicians After Adverse Events: Development of a Clinician Peer Support Program. J Patient Saf. 2018;14(3):e56–60.
54. El Hechi MW, Bohnen JD, Westfal M, Han K, Cauley C, Wright C, et al. Design and Impact of a Novel Surgery-Specific Second Victim Peer Support Program. J Am Coll Surg. 2020;230(6):926–33.
55. Hinzmann D, Forster A, Koll-Krüsmann M, Schießl A, Schneider F, Sigl-Erkel T, et al. Calling for Help—Peer-Based Psychosocial Support for Medical Staff by Telephone—A Best Practice Example from Germany. Int J Environ Res Public Health. 2022;19(23):15453.
56. Scott SD, Hirschinger LE, Cox KR, McCoig M, Hahn-Cover K, Epperly KM, et al. Caring for Our Own: Deploying a Systemwide Second Victim Rapid Response Team. Jt Comm J Qual Patient Saf. 2010;36(5):233–40.
57. Rivera-Chiauzzi E, Smith H, Moore-Murray T, Lee C, Goffman D, Bernstein P, et al. Healing Our Own: A Randomized Trial to Assess Benefits of Peer Support. J Patient Saf. 2022;18(1):e308–14.
58. Foreman S. Developing a process to support perinatal nurses after a critical event. Nurs Womens Health. 2014;18(1):61–5.
59. Bernburg M, Groneberg DA, Mache S. Mental Health Promotion Intervention for Nurses Working in German Psychiatric Hospital Departments: A Pilot Study. Issues Ment Health Nurs. 2019;40(8):706–11.
60. Johnson J, Simms-Ellis R, Janes G, Mills T, Budworth L, Atkinson L, et al. Can we prepare healthcare professionals and students for involvement in stressful healthcare events? A mixed-methods evaluation of a resilience training intervention. BMC Health Serv Res. 2020;20(1094).
61. Peterson U, Bergström G, Samuelsson M, Åsberg M, Nygren Å. Reflecting peer-support groups in the prevention of stress and burnout: randomized controlled trial. J Adv Nurs. 2008;63(5):506–16.
62. Winning AM, Merandi JM, Lewe D, Stepney LMC, Liao NN, Fortney CA, et al. The emotional impact of errors or adverse events on healthcare providers in the NICU: The protective role of coworker support. J Adv Nurs. 2018;74(1):172–80.
63. Carbone R, Ferrari S, Callegarin S, Casotti F, Turina L, Artioli G, et al. Peer support between healthcare workers in hospital and out-of-hospital settings: a scoping review. Acta Biomed Atenei Parm. 2022;93(5):e2022308.
64. Pratt SD, Jachna BR. Care of the clinician after an adverse event. Int J Obstet Anesth. 2015;24(1):54–63.
65. Dukhanin V, Edrees HH, Connors CA, Kang E, Norvell M, Wu AW. Case: A Second Victim Support Program in Pediatrics: Successes and Challenges to Implementation. J Pediatr Nurs. 2018;41:54–9.
66. Liukka M, Steven A, Vizcaya Moreno MF, Sara-aho AM, Khakurel J, Pearson P, et al. Action after Adverse Events in Healthcare: An Integrative Literature Review. Int J Environ Res Public Health. 2020;17(13):4717.
67. Arnetz JE, Zhdanova LS, Elsouhag D, Lichtenberg P, Luborsky MR, Arnetz BB. Organizational Climate Determinants of Resident Safety Culture in Nursing Homes. The Gerontologist. 2011;51(6):739–49.
68. Malik RF, Buljac-Samardžić M, Amajjar I, Hilders CGJM, Scheele F. Open organisational culture: what does it entail? Healthcare stakeholders reaching consensus by means of a Delphi technique. BMJ Open. 2021;11(9):e045515.
69. Aghaei H, Sadat Asadi Z, Mirzaei Aliabadi M, Ahmadinia H. The Relationships Among Occupational Safety Climate, Patient Safety Climate, and Safety Performance Based on Structural Equation Modeling. J Prev Med Pub Health. 2020;53(6):447–54.

For peer review only

70. Moudatsou M, Stavropoulou A, Philalithis A, Koukouli S. The Role of Empathy in Health and Social Care Professionals. Healthcare. 2020;8(1):26.
71. MEASURE Evaluation. Fundamentals of Implementation Research. U.S. Agency for International Development; 2012.
72. Rosak-Szyrocka J. Employee motivation in health care. Prod Eng Arch. 2015;6/1:21–5.
73. Kitsios F, Kamariotou M. Job satisfaction behind motivation: An empirical study in public health workers. Heliyon. 2021;7(4):e06857.
74. Dubois CA, Singh D. From staff-mix to skill-mix and beyond: towards a systemic approach to health workforce management. Hum Resour Health. 2009;7(1):87.
75. Boguslavsky V, Gutierrez R, Holschneider S. Effective Leadership for Quality Improvement in Health Care A Practical Guide. Published by USAID ASSIST Project.Chevy Chase. MD: University Research CO (LLC) URS; 2019.
76. Usman M, Ghani U, Cheng J, Farid T, Iqbal S. Does Participative Leadership Matters in Employees' Outcomes During COVID-19? Role of Leader Behavioral Integrity. Front Psychol. 2021;12:646442.
77. Fagerdal B, Lyng HB, Guise V, Anderson JE, Thornam PL, Wiig S. Exploring the role of leaders in enabling adaptive capacity in hospital teams – a multiple case study. BMC Health Serv Res. 2022;22(1):908.
78. Joint Commission. The essential role of leadership in developing a safety culture. Department of Corporate Communications; 2007.
79. Søvold LE, Naslund JA, Kousoulis AA, Saxena S, Qoronfleh MW, Grobler C, et al. Prioritizing the Mental Health and Well-Being of Healthcare Workers: An Urgent Global Public Health Priority. Front Public Health. 2021;9:679397.
80. Straus SE, Tetroe J, Graham ID, Zwarenstein M, Bhattacharyya O, Shepperd S. Monitoring use of knowledge and evaluating outcomes. Can Med Assoc J. 2010;182(2):E94–8.
81. Wade L, Williams N, Fitzpatrick E, Parker R, Hurley K. Organizational interventions and policies to support second victims in acute care settings: A scoping study. Can J Emerg Med. 2019;21:S112–3.
82. Mira JJ, Lorenzo S, Carrillo I, Ferrús L, Pérez-Pérez P, Iglesias F, et al. Interventions in health organisations to reduce the impact of adverse events in second and third victims. BMC Health Serv Res. 2015;15:341.
83. Lawati MHAl, Dennis S, Short SD, Abdulhadi NN. Patient safety and safety culture in primary health care: a systematic review. BMC Fam Pract. 2018;19(1):104.
84. Simms L, Ottman KE, Griffith JL, Knight MG, Norris L, Karakcheyeva V, et al. Psychosocial Peer Support to Address Mental Health and Burnout of Health Care Workers Affected by COVID-19: A Qualitative Evaluation. Int J Environ Res Public Health. 2023;20(5):4536.
85. Marr R, Goyal A, Quinn M, Chopra V. Support opportunities for second victims lessons learned: a qualitative study of the top 20 US News and World Report Honor Roll Hospitals. BMC Health Serv Res. 2021;21(1):1330.
86. Quadrado E, Tronchin D, Maia F. Strategies to support health professionals in the condition of second victim: scoping review. Rev Esc Enferm USP. 2021;55:e03669.
87. Mira JJ, Lorenzo S, Carrillo I, Ferrús L, Silvestre C, Astier P, et al. Lessons learned for reducing the negative impact of adverse events on patients, health professionals and healthcare organizations. Int J Qual Health Care. 2017;29(4):450–60.

For peer review only

Legend of the figures-

Figure 1 – OPERA- The five key domains to guide HCWs' support interventions after stressful events such as PSI

Supplementary Figure 1 – PRISMA flow chart of the selection process

For peer review only

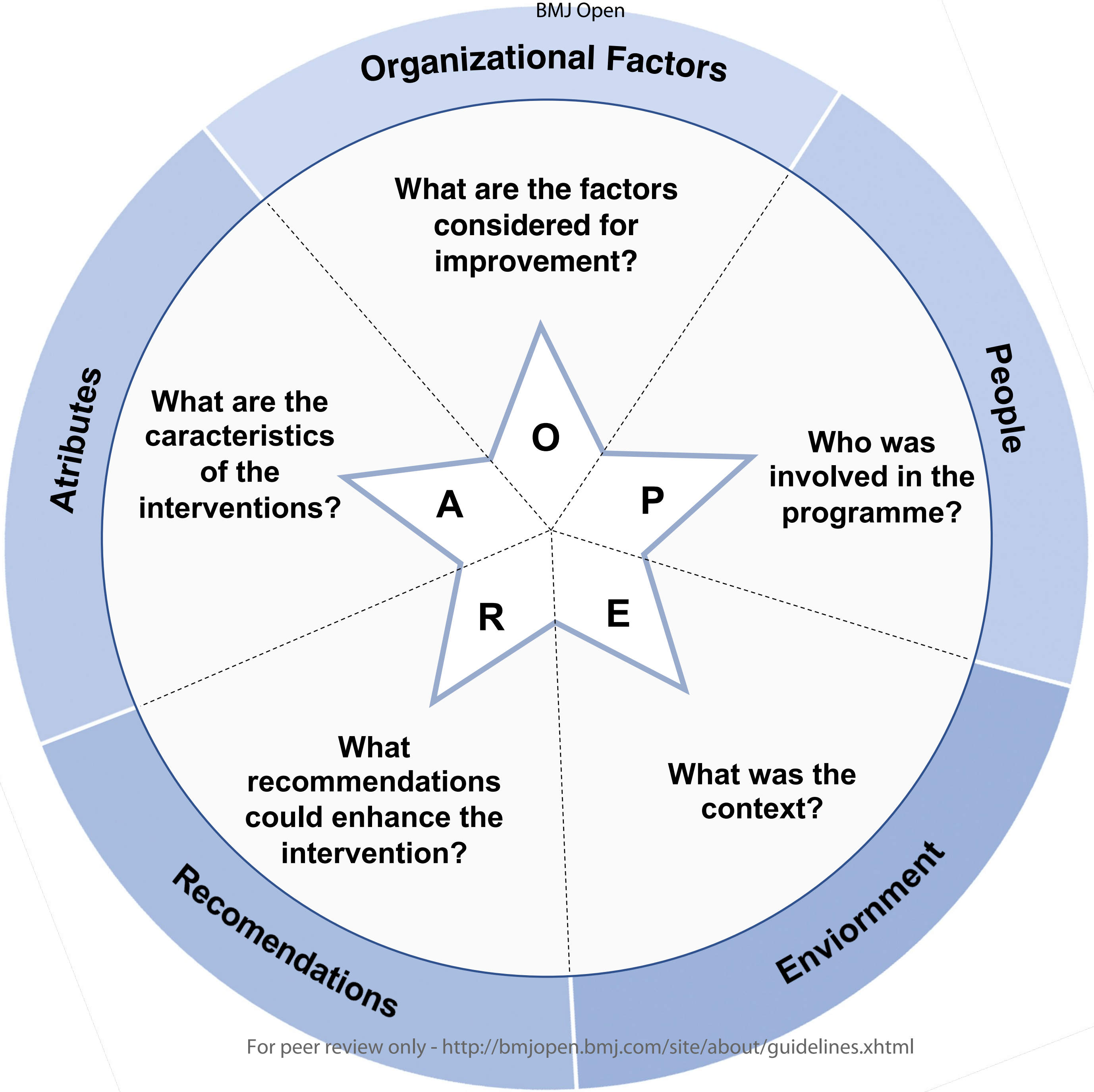
Organizational Factors
People
Enviornment
Recomendations
Atributes
What are the factors considered for improvement?
Who was involved in the programme?
What was the context?
What recommendations could enhance the intervention?
What are the caracteristics of the interventions?
O
P
E
R
A

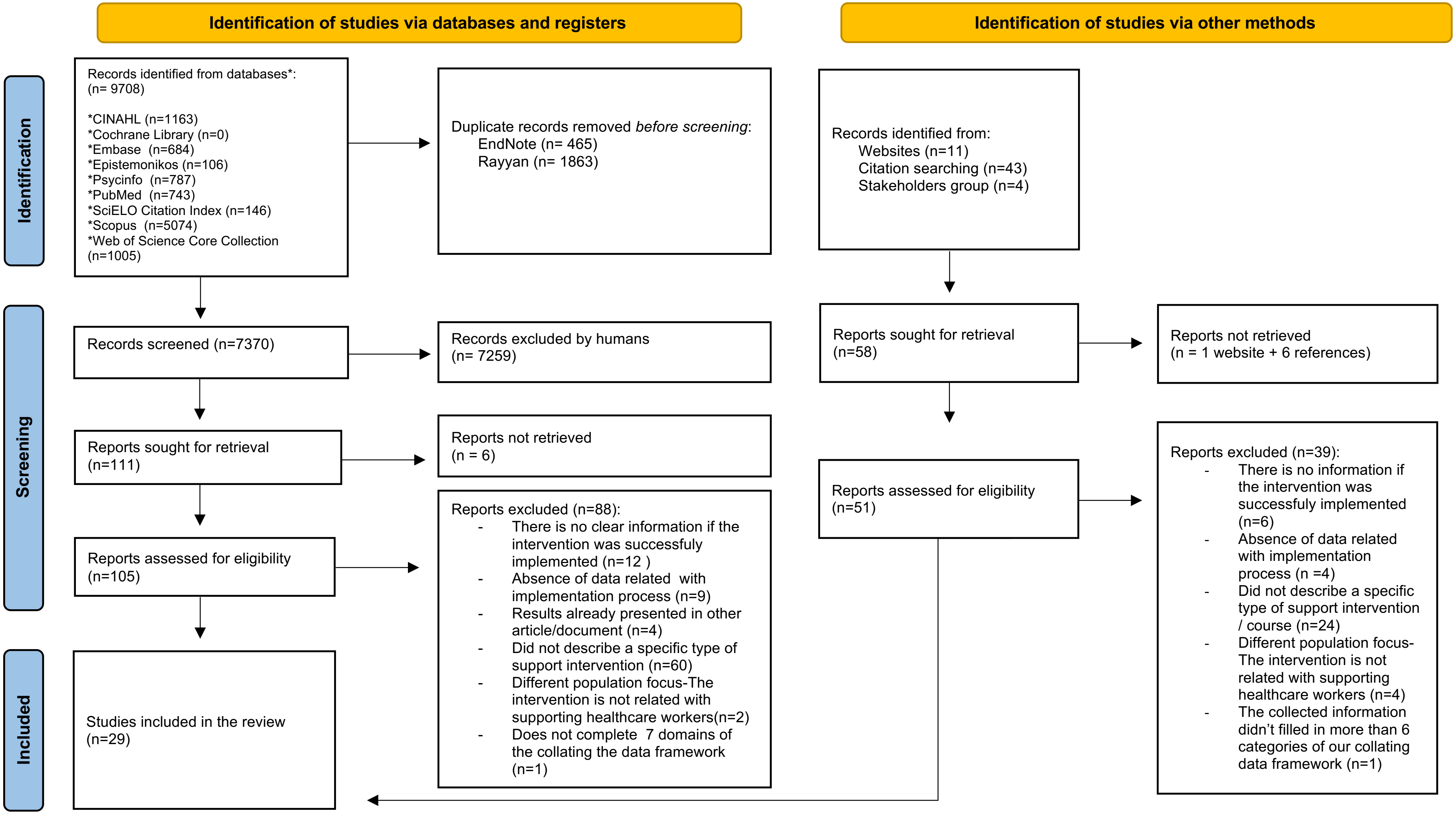
Identification of studies via databases and registers
Identification of studies via other methods
Identification
Screening
Included
Records identified from databases*:
(n= 9708)
*CINAHL (n=1163)
*Cochrane Library (n=0)
*Embase (n=684)
*Epistemonikos (n=106)
*Psycinfo (n=787)
*PubMed (n=743)
*SciELO Citation Index (n=146)
*Scopus (n=5074)
*Web of Science Core Collection
(n=1005)
Duplicate records removed *before screening*:
EndNote (n= 465)
Rayyan (n= 1863)
Records identified from:
Websites (n=11)
Citation searching (n=43)
Stakeholders group (n=4)
Records screened (n=7370)
Records excluded by humans
(n= 7259)
Reports sought for retrieval
(n=58)
Reports not retrieved
(n = 1 website + 6 references)
Reports sought for retrieval
(n=111)
Reports not retrieved
(n = 6)
Reports assessed for eligibility
(n=51)
Reports excluded (n=39):
- There is no information if the intervention was successfuly implemented (n=6)
- Absence of data related with implementation process (n =4)
- Did not describe a specific type of support intervention / course (n=24)
- Different population focus- The intervention is not related with supporting healthcare workers (n=4)
- The collected information didn't filled in more than 6 categories of our collating data framework (n=1)
Reports assessed for eligibility
(n=105)
Reports excluded (n=88):
- There is no clear information if the intervention was successfuly implemented (n=12)
- Absence of data related with implementation process (n=9)
- Results already presented in other article/document (n=4)
- Did not describe a specific type of support intervention (n=60)
- Different population focus-The intervention is not related with supporting healthcare workers(n=2)
- Does not complete 7 domains of the collating the data framework (n=1)
Studies included in the review
(n=29)

Supplementary Table 1 - Search strategies applied to the different electronic databases

**Applied search strategies :**			**Results**
Context	#1	“Health Services” OR “Health Facilities” OR “Healthcare” OR “Primary health care” OR “General Practice” OR “Family practice” OR “Ambulatory Care” OR “Nursing Care” OR “Family unit” OR "Hospitals"	
Content	#2	“Program evaluation” OR "Support program” OR “Peer support” OR “Support strategies” OR “Organizational factors” OR “Organizational culture”	
Population	#3	(“Health personnel” OR “Physicians” OR “Nurses” OR “Doctor” OR “Practitioner” OR “Medical students” OR “Medical residents” OR “Healthcare providers” OR “Healthcare worker” OR “Healthcare staff") AND (“Error” OR “Near miss” OR “Adverse Event” OR “Clinical Error” OR “Medical error” OR “Second victim” OR “Wounded caregiver” OR “Wounded healer” OR “Secondary trauma”)	
Web of Science Core collection<br>*All databases*<br><br>Last updated 2022/10/31	#1 AND #2 AND #3	Error OR Adverse Event OR Clinical Error OR Medical error OR Second victim OR Wounded caregiver OR Wounded healer OR Secondary trauma (Topic) and Health Services OR Health Facilities OR Healthcare OR Primary health care OR General Practice OR Family practice OR Ambulatory Care OR Nursing Care OR Family unit OR Hospitals (Topic) and Program evaluation OR Support program OR Peer support OR Support strategies OR Organizational factors OR Organizational culture (Topic) and Health personnel OR Physicians OR Nurses OR Doctor OR Practitioner OR Medical students OR Medical residents OR Healthcare providers OR Healthcare worker OR Healthcare staff (Topic) and Article or Review Article or Early Access or Editorial Material (Document Types)	1005 results
Complete search on Pubmed (Medline)<br><br>Last updated 2022/10/31	#1 AND #2 AND #3	((((("Health Services"[MeSH Terms]) OR ("Health Services"[Title/Abstract])) OR (("Health Facilities"[MeSH Terms]))) OR ("Health Facilities"[Title/Abstract])) OR ("healthcare"[Title/Abstract])) OR ("Primary health care"[MeSH Terms])) OR ("Primary health care"[Title/Abstract])) OR ("General Practice"[MeSH Terms])) OR ("General Practice"[Title/Abstract])) OR ("Family practice"[MeSH Terms])) OR ("Family practice"[Title/Abstract])) OR ("Ambulatory Care"[MeSH Terms])) OR ("Ambulatory Care"[Title/Abstract])) OR ("Nursing Care"[Title/Abstract])) OR ("Family unit"[Title/Abstract])) OR ("Hospitals"[Title/Abstract]))) AND (("Program evaluation"[Title/Abstract]) OR ("Support program"[Title/Abstract]) OR ("Peer support"[Title/Abstract]) OR ("Support strategies"[Title/Abstract]) OR ("Organizational factors"[Title/Abstract]) OR ("Organizational culture"[MeSH Terms]) OR ("Organizational culture"[Title/Abstract])))) AND (("Health personnel"[MeSH Terms]) OR ("Health personnel"[Title/Abstract]) OR ("Physicians"[Title/Abstract]) OR ("Nurses"[Title/Abstract]) OR ("Doctor"[Title/Abstract]) OR ("Practitioner"[Title/Abstract]) OR ("Medical students"[Title/Abstract]) OR ("Medical residents"[Title/Abstract]) OR ("Healthcare providers"[Title/Abstract]) OR ("Healthcare worker"[Title/Abstract]) OR ("Healthcare staff"[Title/Abstract])))) AND ((Error[Title/Abstract]) OR ((“Near miss”[Title/Abstract]) OR (“Adverse Event”[Title/Abstract]) OR (“Clinical Error”[Title/Abstract]) OR (“Medical error”[MeSH Terms]) OR (“Medical error”[Title/Abstract]) OR (“Second victim”[Title/Abstract]) OR (“Wounded caregiver”[Title/Abstract]) OR (“Wounded healer”[Title/Abstract]) OR (“Secondary trauma”[Title/Abstract])))	743 results

For peer review only

Complete search on PsycInfo<br><br>Last updated 2022/11/3	#1 AND #2 AND #3	(((health services) OR (health facilities) OR (healthcare) OR (primary health care) OR (general practice) OR (family practice) OR (ambulatory care) OR (nursing care) OR (family unit) OR (hospital)) AND ((program evaluation) OR (support programs) OR (peer support) OR (support strategies) OR (organizational factors) OR (organizational culture)) AND ((health personnel) OR (physician) OR (nurse) OR (doctors) OR (practitioner) OR (medical students) OR (medical residents) OR (healthcare providers) OR (healthcare workers) OR (healthcare staff)) AND ((errors) OR (near miss) OR (adverse events) OR (clinical errors) OR (medical errors) OR (second victim) OR (wounded healer) OR (secondary trauma))	787 results
SCOPUS<br><br>Last updated 2022/11/2	#1 AND #2 AND #3	(TITLE-ABS-KEY (health AND services) OR TITLE-ABS-KEY (health AND facilities) OR TITLE-ABS-KEY (healthcare) OR TITLE-ABS-KEY (primary AND health AND care) OR TITLE-ABS-KEY (general AND practice) OR TITLE-ABS-KEY (family AND practice) OR TITLE-ABS-KEY (ambulatory AND care) OR TITLE-ABS-KEY (nursing AND care) OR TITLE-ABS-KEY (family AND unit) OR TITLE-ABS-KEY (hospitals)) AND (TITLE-ABS-KEY (program AND evaluation) OR TITLE-ABS-KEY (support AND program) OR TITLE-ABS-KEY (peer AND support) OR TITLE-ABS-KEY (support AND strategies) OR TITLE-ABS-KEY (organizational AND factors) OR TITLE-ABS-KEY (organizational AND culture)) AND (TITLE-ABS-KEY (health AND personnel) OR TITLE-ABS-KEY (physicians) OR TITLE-ABS-KEY (nurses) OR TITLE-ABS-KEY (doctor) OR TITLE-ABS-KEY (practitioner) OR TITLE-ABS-KEY (medical AND students) OR TITLE-ABS- KEY (medical AND residents) OR TITLE-ABS-KEY (healthcare AND providers) OR TITLE-ABS-KEY (healthcare AND worker) OR TITLE- ABS-KEY (healthcare AND staff)) AND (TITLE-ABS-KEY (error) OR TITLE-ABS-KEY (near AND miss) OR TITLE-ABS-KEY (adverse AND event) OR TITLE-ABS-KEY (clinical AND error) OR TITLE-ABS-KEY (medical AND error) OR TITLE-ABS-KEY (second AND victim) OR TITLE-ABS-KEY (wounded AND caregiver) OR TITLE-ABS-KEY (wounded AND healer) OR TITLE-ABS-KEY (secondary AND trauma)) AND (LIMIT-TO (DOCTYPE , "ar") OR LIMIT-TO (DOCTYPE , "re") OR LIMIT-TO (DOCTYPE , "cp") OR LIMIT-TO (DOCTYPE , "cr"))	5074 results
Complete search on CINAHL<br><br>Last updated 2022/10/31	#1 AND #2 AND #3	TX ("health services" OR "health facilities" OR healthcare OR "primary health care" OR "general practice" OR "family practice" OR "ambulatory care" OR "nursing care" OR "family unit" OR hospital) AND TX ("program evaluation" OR "support programs" OR "peer support" OR "support strategies" OR "organizational factors" OR "organizational culture") AND TX ("health personnel" OR physician OR nurse OR doctors OR practitioner OR "medical students" OR "medical residents" OR "healthcare providers" OR "healthcare workers" OR "healthcare staff") AND TX (errors OR "near miss" OR "adverse events" OR "clinical errors" OR "medical errors" OR "second victim" OR "wounded healer" OR "secondary trauma")	1163 results
Complete search on Embase<br><br>Last updated 2022/10/31	#1 AND #2 AND #3	('health services':ab,ti OR 'health facilities':ab,ti OR 'healthcare':ab,ti OR 'primary health care':ab,ti OR 'general practice':ab,ti OR 'family practice':ab,ti OR 'ambulatory care':ab,ti OR 'nursing care':ab,ti OR 'family unit':ab,ti OR 'hospitals':ab,ti) AND ('program evaluation':ab,ti OR 'support program':ab,ti OR 'peer support':ab,ti OR 'support strategies':ab,ti OR 'organizational factors':ab,ti OR 'organizational culture':ab,ti) AND ('health personnel':ab,ti OR 'physicians':ab,ti OR 'nurses':ab,ti OR 'doctor':ab,ti OR 'practitioner':ab,ti OR 'medical students':ab,ti OR 'medical residents':ab,ti OR 'healthcare providers':ab,ti OR 'healthcare worker':ab,ti OR 'healthcare staff':ab,ti) AND (error:ab,ti OR 'adverse event':ab,ti OR 'clinical error':ab,ti OR 'medical error':ab,ti OR 'second victim':ab,ti OR 'wounded caregiver':ab,ti OR 'wounded healer':ab,ti OR 'secondary traumatic stress':ab,ti)	684 results
Scielo citation index through. Web of Science<br><br>Last updated: 2022/11/4	#1 AND #2 AND #3	("Health Services" OR "Health Facilities" OR "Healthcare" OR "Primary health care" OR "General Practice" OR "Family practice" OR "Ambulatory Care" OR "Nursing Care" OR "Family unit" OR "Hospitals") AND ("Program evaluation" OR "Support program" OR "Peer support" OR "Support strategies" OR "Organizational factors" OR "Organizational culture") AND ("Health personnel" OR "Physicians" OR "Nurses" OR "Doctor" OR "Practitioner" OR "Medical students" OR "Medical residents" OR "Healthcare providers" OR "Healthcare worker" OR "Healthcare staff" and "Error" OR "Near miss" OR "Adverse Event" OR "Clinical Error" OR "Medical error" OR "Second victim" OR "Wounded caregiver" OR "Wounded healer" OR "Secondary trauma")	146 results

For peer review only

Complete search on Epistemonikos<br><br>Last updated 2022/11/2	#1 AND #2 AND #3	(title ((((health services) OR (health facilities) OR (healthcare) OR (primary health care) OR (general practice) OR (family practice) OR (ambulatory care) OR (nursing care) OR (family unit) OR (hospital)) AND ((program evaluation) OR (support programs) OR (peer support) OR (support strategies) OR (organizational factors) OR (organizational culture)) AND ((health personnel) OR (physician) OR (nurse) OR (doctors) OR (practitioner) OR (medical students) OR (medical residents) OR (healthcare providers) OR (healthcare workers) OR (healthcare staff)) AND ((errors) OR (near miss) OR (adverse events) OR (clinical errors) OR (medical errors) OR (second victim) OR (wounded healer) OR (secondary trauma))) OR abstract:(((health services) OR (health facilities) OR (healthcare) OR (primary health care) OR (general practice) OR (family practice) OR (ambulatory care) OR (nursing care) OR (family unit) OR (hospital)) AND ((program evaluation) OR (support programs) OR (peer support) OR (support strategies) OR (organizational factors) OR (organizational culture)) AND ((health personnel) OR (physician) OR (nurse) OR (doctors) OR (practitioner) OR (medical students) OR (medical residents) OR (healthcare providers) OR (healthcare workers) OR (healthcare staff)) AND ((errors) OR (near miss) OR (adverse events) OR (clinical errors) OR (medical errors) OR (second victim) OR (wounded healer) OR (secondary trauma))))	106 results
Language		No language filter/restraint will be applied	
Period		No period filter/restraint will be applied	
Exclusion criteria		Article types not included: editorial , letter to the editor, cases series, case reports, narrative review, commentary	

For peer review only

Supplementary Table 2- Characteristics of the included studies

**Authors (year of publication)**	**Type of study and level of evidence**	**Main aim of the study**	**Study design**	**Methods**	**Key findings**	**Quality assessment**
Allen,Spencer, McEwan,Catarino , Evans, Crooks et al (2020)	Scientific article – Level VI[1]	To evaluate the experience of HCWs working in a mental health service after attending the Schwartz Rounds.	Mixed Method	Application of a quantitative evaluation form after the rounds and focus group and 6 years follow up.	Rounds were helpful, insightful and relevant to support HCWs in a non-blaming environment. The six-year follow-up revealed that the Rounds were still rated positively.	4*/5 of the MMAT criteria *there are no inconsistencies between results
Bryant (2022)	Graduate Theses, Dissertation s - Level VI[1]	To raise staff awareness on resilience, SVP and SupportingYOU intervention in a large academic children's hospital.	Quantitative descriptive	Application of preintervention and postintervention quantitative surveys,	Intervention increased the staff awareness on SVP, their perception on resilience and contribute for a proactive culture to manage critical incidents, by increasing the sense of feeling cared by the institution.	5*/5 of the MMAT criteria
Civil, Hoskins (2022)	Scientific article- Level VI[1]	To describe the design and implementation of a critical incident peer response team program at Waikato Hospital.	Quantitative descriptive	Application of postintervention quantitative survey.	Positive feedback from the attendants was given after the intervention. The program contributed to a supportive culture and enhanced teamwork.	2*/5 of the MMAT * 3 criteria were evaluated as "can't tell" : the sample is not representative of the target population, there is a risk of nonresponse bias; absence of statistical analysis
Edrees, Connors, Paine, Norvell, Taylor, Wu (2016)	Scientific article- Level VI[1]	To describe the development of RISE, initial evaluation of pilot programme and hospital-wide implementation at Johns Hopkins Hospital.	Mixed Method	Application of pre and post implementation quantitative surveys and focus group.	Although there were few calls in the first year of implementation, the rate of calls increased during the next years. Evaluation indicates the success of most encounters with callers and effectiveness of training to prepared the peer supporters to support second victim.	4*/5 of the MMAT criteria *there are no inconsistencies between results
Finney, Jacob, Johnson, Messner, Pulos, Sviggum (2021)	Scientific article Level VI[1]	To describe the implementation and evaluation of a SV program – Healing Emotional lives of Peers (HELP) in a Department of Anaesthesiology.	Mixed method	Application of post implementation quantitative surveys with open questions.	The program developed during 3 years was successfully implemented in the first 2 years of the program inception and is now a resource for other institutions in the region.	4*/5 of the MMAT criteria *no confounders accounted for in the design and analysis
Foreman (2014)	Paper Level VI[1]	To describe the development and implementation of a plan to help perinatal nurses to cope with stress after critical incidents and deaths in a family birth centre.	Qualitative	Qualitative description of the programme testing and the learning from its implementation.	Feedback on the use of the critical event plan has been positive. Nurses found the plan very useful after a tragedy on the unit related with the death of a new-born.	3*/ 5 of the MMAT criteria * 2 criteria were evaluated as "can't tell": qualitative data collection methods are not described; coherence between qualitative data sources, collection, analysis and interpretation is not clear;
Graham, Zerbi, Norcross, Montross-	Scientific article	To describe the Caregiver Support Team programme implementation in	Mixed-method	Application of baseline and post implementation (3 months follow up)	As an addition to the previous implemented Code Lavender intervention, the program was accepted and positively evaluated. Efforts will	4*/5 of the MMAT criteria *no confounders accounted for in the design and analysis

Thomas, Lobbestael , Davidson (2019)	Level VI[1]	an academic medical centre and evaluate its feasibility.		quantitative surveys . Self reported experiences of attendants.	be made to disseminate the program system-wide.	
Johnson, Simms-Ellis, Janes, Mills, Budworth, Atkinson ,Harrison (2020)	Scientific article<br>Level VI[1]	To evaluate a psychological resilience coaching intervention for National Health Services (NHS) trust sites and university premises.	Mixed-method	Application of pre and post implementation, follow up after the coaching phone call (10–20 days after the workshop) and 4–6 weeks after the workshop. Interviews application.	The program seems to be feasible and effective on improving general resilience of clinicians, by improving their knowledge and confidence in coping after adverse events.	5/5 of the MMAT criteria
Krzan, Merandi, Morvay, Mirtallo (2015)	Scientific article<br>Level VI[1]	To describe YOU Matter Support program implementation at Nationwide Children's Hospital.	Mixed-method	Application of pre and post (after 5 months) online surveys. Collection of peer encounters documentation of the Second Victim SharePoint site.	Most of the surveyed staff reported that the department benefited from implementation of the SV program. After the success of the pilot program, the hospital has decided to expand the YOU Matter program hospital- wide.	4*/5 of the MMAT criteria *no confounders accounted for in the design and analysis
Merandi, Liao , Lewe, Morvay, Stewart, Catt, Scott (2017)	Scientific article<br>Level VI[1]	To describe the replication of the ForYOU Matter program and expansion of the program to a large pediatric institution.	Quantitative descriptive	Description of the program implementation and of the collected data from electronic tracking system via SharePoint.	Application of the MUHC support model in the Nationwide Children's Hospital was validated and demonstrates that it's suitable and transferrable to other healthcare facilities and contexts.	3*/5 of the MMAT criteria<br>2 criteria was evaluated as "can't tell": no clear information if the risk of nonresponse bias was low: absence of statistical analysis
Lane, Newman, Taylor, O'Neill, Ghetti, Woltman, Waterman (2018)	Scientific article<br>Level VI[1]	To describe the Washington University School of Medicine Clinician Peer Support Program.	Quantitative descriptive	Description of the program development and its implementation.	Program was successfully implemented, however difficulties were found in sustaining the program over time, since peer supporters don't have protected time to be part of the program and therefore the programme activities have conflict with their operative schedules.	3*/5 of the MMAT criteria –<br>*2 criteria was evaluated as "can't tell": nonresponse bias was not applicable to this study; absence of statistical analysis
Mellins, Mayer, Glasofer, Devlin, Albano, Nash et al (2020)	Scientific article<br>Level VI[1]	To describe the application of CopeColombia programme in a Department of Psychiatry of a large urban medical centre's.	Mixed method	Description of the key issues/themes and facilitator responses emerged in the sessions (facilitator interventions);<br>post peer supporter group online survey (perceived impact of the program).	HCW emotional distress decreased after the program implementation. Peer Support Groups were the most used, valued and recommended. Sustainability of the program is critical due to financial constrains.	3*/5 of the MMAT criteria<br>*there are no inconsistencies between results;<br>1 criteria was evaluated as "can't tell": it's not clear if the study follows the traditional quality criteria of the qualitative methods involved
Merandi, Winning , Liao, Rogers, Lewe, Gerhardt (2018)	Scientific article<br>Level VI[1]	To assess healthcare providers satisfaction in the early implementation of a SV program in a group of neonatal intensive care units.	Mixed method	Application of pre and post implementation surveys with closed and open – ended questions. Thematic content analysis of the qualitative data from the survey.	This study suggest that peer support programs are likely to be viewed as positive and helpful for frontline HCW and managers. However, additional work should be done to assure the accessibility and effectiveness of the program.	4*/5 of the MMAT criteria *no confounders accounted for in the design and analysis

For peer review only

Rivera-Chiauzzi,Smith,Moore-Murray, Lee, Goffman, Bernstein, Chazotte (2022)	Scientific article<br><br>Level II[1]	To describe the development and evaluation a structured peer support program to address the needs of providers involved in obstetric adverse outcomes and to compare it with a routine support for HCW after the same type of events.	Pilot Randomized controlled trial	Application of needs assessment survey before program implementation. Surveys were applied at baseline, 3 months, and 6 months follow up.	Structured peer support program was successfully implemented with limited resources. The enhanced support group was significantly more likely to consider departmental leadership as one of the most helpful resources for support after adverse event. All participants refer were thriving at 6-month follow-up.	4*/5 of the MMAT criteria<br><br>*outcome assessors are not blinded to the intervention provided
Roesler, Ward, Short (2009)	Scientific article<br><br>Level VI[1]	To describe the recovery and reintegration of Neonatal Intensive Care Unit staff after a critical incident based on the Just Culture philosophy.	Qualitative: Single Case	Description of the protocol activation and application after the involvement of a severe adverse event.	The protocol was successfully applied and helped the affected HCW to thrive and return back to work after their involvement in the severe adverse event.	3*/5 of the MMAT criteria<br>*2 criteria were evaluated as "can't tell": qualitative data collection methods are not described ; it's not clear if there is coherence between qualitative data sources, collection, analysis and interpretation
Schrøder, Bovil,, Jørgensen, Abrahamsen (2022)	Scientific article<br><br>Level VI[1]	To evaluate the Buddy Study program by assessing HCW experiences with having the program in the department, attending the compulsory seminar, and using a buddy or being activated as a buddy.	Mixed method	Application of quantitative questionnaires to assess HCW experiences of attending the Buddy Study seminar and participating in the Buddy Study program using a buddy or being activated as a buddy, along with two open-ended questions and three individual interviews for more elaborate answers.	The buddy study program was evaluated positively. It allowed to acknowledge the SVP and strengthen a supportive organizational culture by creating buddy relationships to support HCW after stressful events during care.	4*/5 of the MMAT criteria *no confounders accounted for in the design and analysis
Shapiro, Galowitz (2016)	Scientific article<br><br>Level VI[1]	To describe the development and implementation of Brigham and Women's Hospital peer support program to support HCW after the impact of an emotional stressful event.	Quantitative descriptive	Description of the program and quantitative data collection of outreached calls and peer support sessions.	The peer support program has been implemented for 4 years and it's expected to be expanded hospital-wide. The program does not yet reach many clinicians that might be in need of support after stressful events.	3*/5 of the MMAT criteria<br>*3 criteria were evaluated as "can't tell": it's not clear if the sample is representative of the target population; risk of nonresponse bias low and statistical analysis are not applicable
Thompson, Hunnicutt, Broadhead, Vining, Aroke (2022)	Scientific article<br><br>Level VI[1]	To describe the implementation of SV support program in a large academic medical centre based on a quality improvement project.	Quantitative descriptive	Application the SVEST survey pre and post peer support program implementation.	Although the study didn't find statistically significant differences in pre and post implementation the program has received a positive feedback among leadership members and peer supporters.	4*/5 of the MMAT criteria<br>*2 criteria were evaluated as "can't tell": low response rate in post-implementation survey may have decreased generalizability of results which can affect the sample representative ; potential for response bias was identified in the study

El Hechi , Bohnen, Westfal, Han, Cauley, Wright, Schulz et al (2020)	Scientific article<br><br>Level VI[1]	To describe the design and implementation of a SV surgical peer support program and its 1 year impact.	Mixed method	Programme design: literature review and multidisciplinary expert group discussion**;** Evaluation of the impact of the programme: quantitative and qualitative surveys application – evaluation of the peer supporters and of the program.	The first surgery-specific peer support program in US was successfully implemented. After 1 year experience, the program is highly used and well received.	4/5 of the MMAT criteria<br>*inconsistencies between quantitative and qualitative are not addressed in the study
Mira, Carrillo, Guilabert, Lorenzo, Pérez-Pérez, Silvestre et al (2017)	Scientific article<br><br>Level VI[1]	To describe the development and assessment of Mitigating Impact in Second Victims (MISE) programme on the awareness and information focused on the SVP.	Quantitative descriptive	Description of accreditation process; quantitative survey applied to 26 experts to assess structure and content; Quantitative survey applied to frontline HCW; evaluation of knowledge gained from the online program after each online module.	The online program was positively assessed by the accreditation agency, by the experts and the HCWs. The programme increases knowledge about patient safety, SVP and how to act after a severe adverse event. The time demand is reasonable for complete the course.	5/5 of the MMAT criteria
Scott, Hirschinger, Cox, McCoig, Hahn-Cover, Epperly et al. (2010)	Scientific article<br><br>Level VI[1]	To describe the deployment of ForYOU Team programme , an institutional rapid response system (RRS) for second victims at University of Missouri Health Care (MUHC).	Mixed method	Interviews and quantitative surveys application by the MUHC faculty and staff to support the development of the programme.	After identifying the need for support the SV in MUHC , the programme was designed, developed and successfully implemented. The programme has been integrated in the healthcare in the scheduled activities of the team leaders and clinical teams.	4*/5 of the MMAT criteria<br>*inconsistencies between quantitative and qualitative are not addressed in the study
Schuster (2021)	Scientific article<br><br>Level VI[1]	To describe the implementation of the HART programme and assess its impact on the Hematology/Oncology/Stem Cell Transplant Unit.	Mixed method	Preintervention and midpoint survey (after 3 months of pilot program), and qualitative subjective information collection from daily coach documentation entries.	HART programme was successfully implemented in the department and with high level of utilization from the staff. After implementation of HART, mental, emotional and physical wellbeing of all staff members improved.	4*/5 of the MMAT criteria<br>*inconsistencies between quantitative and qualitative are not addressed in the study
Calder-Sprackman; Kumar; Gerin-Lajoie; Kilvert; Sampsel (2018)	Scientific article<br><br>Level VI[1]	To describe the implementation, adaptation and evaluation of the ice cream rounds intervention in an emergency medicine training programme.	Quantitative descriptive	Application of a pre-implementation survey for needs assessment and a post implementation for feedback after intervention.	This Canadian initiative increased the overall perception of support and companionship, decreased feelings of stress, anxiety and burnout and can have a positive impact on the clinical practice in emergency medicine residents.	3*/5 of the MMAT criteria<br>*2 criteria were evaluated as "can't tell": it's not clear if the sample is representative of the target population; absence of statistical analysis

For peer review only

Peterson, Bergström, Samuelsson, Åsberg, Nygren (2008)	Scientific article<br><br>Level II[1]	To evaluate the effect a reflecting peer-support group on self-reported health, burnout and on perceived changes in work conditions of multidisciplinary group of HCW in 3 hospitals of a county council in Sweden.	Randomized controlled trial.	Application of a baseline survey (7 months before the interview started), pre implementation questionnaire , immediately after the intervention ended (post intervention), 7 and 12 months after the intervention follow up.	The study showed positive intervention effects (after 12 months of the programme implementation) on overall health and decreased perceived work demands. A decrease in exhaustion, depression and anxiety symptoms was observed , as well as an increase in vitality.	5/5 of the MMAT criteria
Rubin, Rassman (2021)	Scientific article<br><br>Level VI[1]	To describe the social work led peer support model, COVID-19 Am I Resilient (cAIR), developed and implemented during the first wave of the COVID-19 pandemic in a large urban healthcare system.	Mixed method	Application of pre and post intervention surveys with quantitative and open ended questions.	The pilot programme cAIR was successfully implemented and provided support to normalize frontline HCW experience during Covid 19 pandemic, encouraged coworkers connections and developed coping skills . Participant engagement in cAIR were strong, however overall utilization of programme activities was low.	4*/5 of the MMAT criteria<br>*inconsistencies between quantitative and qualitative are not addressed in the study
Bernburg, Groneberg , Mache (2019)	Scientific article<br><br>Level II[1]	To develop, implement and evaluate the effectiveness of a mental health intervention programme on nurses' perceived job stress, perceptions of individual coping skills and the quality of patient relations in a group of psychiatric hospital departments.	Randomized controlled trial	Pre and post intervention surveys were applied using Perceived Stress Questionnaire; the Brief Resilient Coping Scale; the Self- Efficacy, Optimism and Pessimism Questionnaire; Emotion Regulation Skills Questionnaire and the German Quality of Relationship Inventory – at baseline , follow up after 3 months, 6 months and after 12 months.	After the intervention being successfully implemented, psychiatric nurses significantly increased their perception about job stress, emotion regulation skills, resilience, and self-efficacy; quality of patient-relationship were significantly higher ($p < 0.05$). The results indicate that self- care skills training may improve such as resilience and self-efficacy.	4*/5 of the MMAT criteria<br>*outcome assessors are not blinded to the intervention provided
Morales, Brown (2019)	Scientific article<br><br>Level VI[1]	To describe the development of the Care for the Caregiver Program in a 10 hospital system.	Qualitative	Description of the "Care for the Caregiver" programme and of practical scenarios after the programme was been activated.	Both described scenarios of support after stressful events indicate that the programme has been useful and well received by the clinicians.	4*/5 of the MMAT criteria<br>* 1 criteria was rated as can't tell : coherence between qualitative data sources, collection, analysis and interpretation is not applicable in this study
Cobos-Vargas, Pérez-Pérez, Núñez-Núñez, Casado-Fernández, Bueno-Cavanillas (2022)	Scientific article<br><br>Level VI[1]	To describe the Procedure for Serious Adverse Events (PSAE), integrated into a sentinel event operational procedure and to describe its accumulated results over two years of implementation in a university hospital.	Mixed method	Collection of quantitative data related with programme application, characteristics of involved HCWs and adverse events; HCWs qualitative feedback on programme improvement solutions and	The programme had a positive impact on patient safety culture and it increased the adverse event reporting in the hospital. The level of programme acceptance was high and HCWs valued to be individually interview after their involvement in the event and to contribute for future improvement actions.	4*/5 of the MMAT criteria<br>*inconsistencies between quantitative and qualitative are not addressed in the study

For peer review only

				their experience after attending the programme.		
Hinzmann, Forster, Koll-Krüsmann, Schießl , Schneider, Sigl-Erkel et al (2022)	Scientific article<br><br>Level VI[1]	To evaluate the burden, benefits, and mechanisms of action of a telephone support service for HCWs (PSU-HELPLINE).	Mixed methods	Application of two surveys: i) quantitative survey during the call(socio-demographic data, current stress ,relief from the counselling interview); ii) meta-questionnaire (to document the call and for quality assurance proposes) . Memory transcripts for thematical analysis were also collected.	The PSU-HELPLINE was mainly used for processing serious events and in phases of overload. The programme was well received by a specific region of Germany and callers consider it useful and supportive service.	4*/5 of the MMAT criteria<br>*inconsistencies between quantitative and qualitative are not addressed in the study

For peer review only

Supplementary Table 3 - Characteristics of the included interventions

Note: We have contacted the authors to complete the missing information, however some of the authors didn't reply to our contact.

Author (Year of publication)	Starting year of the intervention	Duration of the intervention	Country and Setting	Target population	Aim of the intervention	Main outcomes related with the intervention
Allen,Spencer , McEwan,Catarino, Evans, Crooks et al (2020)	Missing data	6 months	UK - Mental health service, inpatient acute psychiatric unit	Nursing care, healthcare assistance, occupational therapy, psychiatry, psychological assistance, domestic support building states, administration services, crisis resolution , home treatment team and perinatal mental health team	To understand experiences of emotional distress and reflecting on its impact and ways of coping	**i) Evaluation of Schwartz Rounds*:** From an average evaluation form response was 3.42 out of 4;<br>**ii) Experience of the participants after attending Schwartz Rounds:** In the Rounds, HCWs could express their emotions in an non-judgemental and empathic environment. Rounds were considered helpful, insightful and relevant;<br>**iii) Long-term follow-up of 6 years**: Rounds were still rated positively.<br>*Based on the following criteria: plan to attend the Rounds again, gained insight into thoughts and feelings, facilitator helped the discussion, the overview and presentation of the Rounds were helpful, it helped to improve the relation with colleagues in the work, the knowledge was useful for caring patients, the case was relevant to the clinical work
Bryant (2022)	2021	13 months	US - Intensive care unit, lactation services, child-life services, paediatrics medical surgical units	Staff members from lactation services and paediatric medical-surgical units. child life specialists, physicians, nurses, respiratory therapists, social workers, clinical associates, pharmacist and chaplains	To raise awareness of SVP and SupportingYOU. To increase resiliency by establishing a proactive culture to manage critical incidents prior they occur	**i) Staff perception on resiliency**: 100% of the surveyed staff reported an enhanced perception of resilience (p<0,001, CI 95%);<br>**ii) Staff awareness of SVP** significantly increased (p<0,001, CI 95%);<br>**iii) Staff awareness of SVP resources and of SupportingYOU**: it was limited since that most of participants were already aware of it;<br>**iv) Sense of preparedness in handling critical incidents**: there was an increased sense of managing emotional response after critical incidents among the participants (p<0,01, CI 95%);<br>**v) Feeling cared by the institution –** participants felt care for in the workplace (p<0,01, CI 95%).
Civil, Hoskins (2022)	2018	36 months	New Zealand – Department of Anaesthesia and Pain Medicine	Anaesthetists, surgeons, anaesthetist technicians, theatre nurse coordinator/charge nurse; theatre and recovery room nurses and midwifes	To provide peer-led group psychological first aid to full theatre team stressful events?	**i) Number of participants that attended the defuse:** over 200 members;<br>**ii) Number of interventions** : 28 defuse interventions have been delivered;<br>**iii) Participants feedback of the defuse** : Defuses were rated through neutral to very helpful, all surveyed participants would attend a defuse again in the future and would recommend to colleague or a friend.

For peer review only

Edrees, Connors, Paine, Norvell, Taylor, Wu (2016)	2011	52 months (from november 2011 to march 2016)	US- Johns Hopkins Hospital	All hospital staff	To provide timely psychological first aid and emotional support after critical incidents based on a call system	**i) Frequency of encounters:** 119 encounters from November 2011 and march 2016;<br><br>**ii) Caller interaction:** rated as excellent 66,7% or neutral (22,8%).
Finney, Jacob, Johnson, Messner, Pulos, Sviggum (2021)	2018	23 months (from July 2018 to June 2020)	US- Department of anaesthesiology at a large academic institution in the Midwest	Anaesthesiologist, certified nurse anaesthetics , residents, students registered nurse anaesthetics	To provide three-tiered support psychological first aid : first level given by peer and leadership at the local, second level provided by trained peer supporters; third level was provided by external services (from the instituition or outside the instituition)	**i) Experience after receiving peer support:** From 31 surveyed participants, 25 (80.0%) evaluated the received support as "extremely" or "very beneficial", and 28 (90.3%) referred that were "extremely" or "very satisfied" with their experience; 30 (96.8%) would recommend HELP programme to a colleague;<br><br>**ii) HELP Programme Activations for Peer Support:** 91 electronic activations were utilized to assist 179 HCWs (the most common events leading to HELP activation were intraoperative patient demise, cardiac arrest, and pediatric care);<br><br>**iii) Trained peer support self-assessments:** From 85 surveyed peer supporters, 81 (95.3%) felt satisfied with how the peer support encounter turned out; 19 (22.4%) felt that they needed additional training and experience, 80 (94.1%) felt comfortable with their knowledge and skills as a peer supporter.
Foreman (2014)	Missing data	Info missing	US- Family birth centre Wilcox Women's Pavillon at Legacy Good Samaritan Hospital	All perinatal nurses of the family birth caring for women and newborns in all phases of childbearing	To create a critical event plan and implement it in the centre for helping staff to organize and manage critical events	**i) Experience on the use of the Critical Event Plan:** Nurses found it very useful and helpful**.**
Graham, Zerbi, Norcross, Montross-Thomas, Lobbestael , Davidson (2019)	Missing data	3 months	US- 4 target units in an academic medical centre: telemetry unit, emergency department, neonatal ICU and medical and neurologic ICU	Staff and physicians employed in the units	To provide emotional first aid in the workplace after critical events	**i) Programme activation :** 38 activations of the programme;<br><br>**ii) Experience after the programme:** all surveyed staff found it helpful and would recommend it to other colleagues; HCWs referred an improvement in feeling cared-for and also noted the sense of safety at the workplace;<br><br>**iii) Quality of life assessment and job satisfaction**: No significant changes were demonstrated before and after the intervention in Quality ofLife Scores or job satisfaction. One suicide was prevented;<br><br>**iv) Organizational changes :** Debriefings started to be requested after significant events affecting the entire department.
Johnson, Simms-Ellis, Janes, Mills, Budworth, Atkinson	2018	7 months	UK- National Health Services (NHS) trust sites and University of Leeds	HCWs or students that complete an education programme: midwives, paramedics , obstetrics and gynaecology trainee	To strengthen resilience and preparedness in dealing with adverse events : more flexible thinking, higher self-	**i) Experience after the programme:** workshop was useful, relevant and adequate in length**.** The participants highly valued the peer learning, the level of engagement and the format of the workshop delivery. The coaching call was critical to the consolidation of the knowledge and to

,Harrison (2020)				doctors , paediatric trainee doctors, paediatric consultant, physician associate students and sonography and mammography students.	esteem, better explanatory style	understand how to apply the acquired skills in practice. Knowledge about coping strategies led to a significant increase in self-perceived resilience, as well as confidence in coping with adverse events.
Krzan, Merandi, Morvay, Mirtallo (2015)	2013	5 months	US- pharmacy department at Nationwide Children's Hospital (NCH): one main inpatient pharmacy, inpatient pharmacy satellites, two outpatient pharmacies and home care pharmacy	Staff from all areas of pharmacy services	To provide three-tiered support psychological first aid (based on Susan's Scott model) to support HCWs involved in adverse drug events, patient-related injuries, and other traumatic work experiences	**i) Activation of the programme:** 3 respondents personally used the programme and 11 had recommend it;<br><br>**ii) Experience after the programme :** 85% of the pharmacy (95 of 112 respondents) refer that the department had benefit from the programme's implementation.
Merandi, Liao, Lewe, Morvay, Stewart, Catt, Scott (2017)	2012	60 months (5 years)	US- Nationwide Children's Hospital (NCH)	Staff from Nationwide Children's Hospital (NCH): all inpatient units as well as urgent cares, outpatient primary care clinics, and ambulatory specialty clinics	To provide three-tiered support psychological first aid (based on Susan's Scott model) to support HCWs involved in adverse drug events, patient-related injuries, and other traumatic work experiences	**i) Activation of the programme:** 21 group encounters were documented since November 2013;<br><br>**ii) Quantitative description of peer encounters:** 62% of peer encounters occurred in the emergency department, and 8% in pediatric intensive care unit and cardiothoracic intensive care unit ; nurses have the highest number of peer support encounters. Patient death and emotional stress were the most common reasons for peer encounter.
Lane, Newman, Taylor, O'Neill, Ghetti, Woltman, Waterman (2018)	2014	33 months	US- Two Hospitals affiliates of the Washington School of Medicine:<br>Barnes -Jewish Hospital and St. Louis Children's Hospital	Doctors, junior doctors, physician associates, nurse practitioners, and registered nurse anaesthetists, trainees providing care in the inpatient and outpatient settings	To facilitate the support of clinicians who have been involved in an adverse event or another adverse outcome during medical care	**i) Programme activation:** 165 clinicians were referred to the programme;<br><br>**ii) Median number of interactions per month:** 4.8 referrals per month (Range 0-12).
Mellins, Mayer, Glasofer, Devlin, Albano, Nash et al (2020)	2020	3 months	US-Large urban , academic, tertiary care - Columbia University Irving Medical Centre (CUIMC)	All CUIMC clinical and non-clinical staff, residents and faculty students	To provide peer support after stressful situations and enhance resilience of HCWs	**i) Emotional distress assessment:** The average emotional distress had significantly decreased (*p*<0.05)**;**<br><br>**ii) Perceived helpfulness of the peer support group:** Perceived helpfulness was high (76% rating helpfulness as "quite a bit" or "extremely."); all respondents (with the exception of two) recommended the support group to a colleague.

Merandi, Winning , Liao, Rogers, Lewe, Gerhardt (2018)	2015	12 months	US-7 neonatal intensive care units (NICUs) from quaternary-care, paediatrics hospital	All staff from the 7 NICUs	To provide three-tiered support psychological first aid (based on Susan's Scott model) to support HCWs involved in adverse drug events, patient-related injuries, and other traumatic work experiences	**i) Attendance to the programme:** A small number of healthcare providers (n=6) used the program. Some HCWs (not quantified) did not find the programme accessible. **ii) Satisfaction with the programme:** The majority of the participants (56.3%, n=9) reported moderate benefit of the programme. From 250 surveyed HCWs in the units, 73.2% indicated that the NICUs benefited at least a little from the SV peer support programme. The manager reported that the programme enhanced the team leadership.
Rivera-Chiauzzi,Smith,Moore-Murray, Lee, Goffman, Bernstein, Chazotte (2022)	2015	8 months	US- Department of obstetrics & Gynaecology and Women Health	HCWs and residents (physicians and nurses) who experienced an obstetric adverse outcome.	To provide peer support to HCWs and residents, who have experienced an obstetric adverse outcome, through phone calls	**i) Attendance to the programme :** Out of 34 programme activations, 23 participants were placed in the enhanced group and 19 were assigned in the routine group; **ii) Use of support resources and perception of its helpfulness:** Peer support was the most common source of assistance (*p*<0.05); departmental leadership was considered one of the most helpful resources for the enhanced support group; **iii) Identified barriers:** Time (*P* = 0.26) was reported as a constraint by participants in the enhanced group, and routine group reported privacy concerns (*P* = 0.39) and stigma (*P* = 0.12)**;** **iv) Effect of the programme on the stage of recovery:** At 6-month follow-up, all participants in the enhanced group reported that they were thriving after the event; **v) Duration of peer support interventions:** most participants required less than 3 months of support to recover from the event.
Roesler, Ward, Short (2009)	2006	No clear information (estimated time : approx. 6 months)	US- Neonatal Intensive Care Unit (NICU), Methodist Hospital of Indianapolis	Staff of the NICU	To provide an institutional incident response after a paediatric serious adverse event	**i) Staff and unit recovery:** The pharmacist technician and 5 out of 6 nurses affected by the incident were back to work after the adverse event (1 nurse didn't return to work for familiar reasons). None of the affected HCWs experienced job changes. Debriefing and healing sessions were essential to the staff unit overcome the situation; **ii) Impact on work culture**- Reinforcement of the culture of disclosure after applying the protocol.
Schrøder, Bovil,, Jørgensen, Abrahamsen (2022)	2018	18 months	Denmark-2 departments at Odense University Hospital: Department of Obstetrics and Gynaecology (OB-GYN) and Svendborg	All midwives in the OB-GYN and all physicians at the IME	To provide one-on-one peer support to HCW following a stressful event, including adverse events	**i) Attendance to the programme:** Out of 156 respondents, 26 of them reported using one of their buddies during the study period**;** **ii) Evaluation of the Buddy Study programme in the department:** The programme encouraged more attentiveness among participants towards one another following adverse events (n=82, 52.6%) ; have

For peer review only

			and Internal Medicine and Emergency Department (IME)			contributed to more inter-collegial talks about adverse events (n=57,36.5%) ; sense of more openness to talk with colleagues about feelings in the aftermath of adverse events (n=65, 41.7%) ; increased willingness to ask for leadership support (n=53, 34.0%);<br>**iii) Overall experience with the programme:** an open and compassionate culture was encouraged; increased attentiveness to the staff wellbeing ; increased sense of safety (n=91, 58.3%).
Shapiro, Galowitz (2016)	2012	47 months (January 2012 and December 2015)	US- Brigham and Women's Hospital (BWH)	HCWs of Brigham and Women's Hospital	To provide one-on-one peer support or group peer support (if a team is likely to be affected), after a stressful event, including adverse events	**i) Activation of the programme:** 220 outreach calls to one-on-one peer support;<br>**ii) Attendance to the group sessions:** 240 clinicians participated in multidisciplinary group peer support sessions.
Thompson, Hunnicutt, Broadhead, Vining, Aroke (2022)	May, 2020 through October, 2020	1 month (trial period)	US – Anaesthesia department of the large academic centre	Certified registered nurses of the anaesthesia department.	To provide a peer support program to decrease SV distress after stressful events	**i) Frequency of the encounters:** Over the course of one month, a total of 8 peer support encounters were reported;<br>**ii) HCWs' distress**: It wasn't possible to observe a significant change in distress level after one month period, however the study found a statistically significant relationship between increased distress and insufficient colleagues support.
El Hechi , Bohnen, Westfal, Han, Cauley, Wright, Schulz et al (2020)	Missing data	12 months	US-Department of surgery at a tertiary academic medical centre	Surgeons and surgical trainees from general surgery, transplantation, pediatric surgery, thoracic surgery, cardiac surgery, vascular surgery, and trauma/acute care surgery.	To provide a peer support - based on three-tiered support psychological first aid - to surgeons and surgical trainees dealing with intraoperative adverse events, catastrophic patient outcomes, and/or long- term litigation cases.	**i) Programme activation:** 47 outreach calls;<br>**ii) Evaluation of the programme impact:** The majority of surgical staff was satisfied with the following domains – programme's confidentiality (89%), safe/trusting environment (73%,), timeliness of the intervention (83%). It was also perceived a positive impact on the department's culture, as it raised awareness about the importance of supporting colleagues facing difficult situations at work, and fostered a general sense of "safety and support" in the workplace.
Mira, Carrillo, Guilabert, Lorenzo, Pérez-Pérez, Silvestre et al (2017)	2015	15 months	Spain – without a specific location (online programme)	HCWs from hospitals and primary care that access the webpage of the online programme	To provide an online preventive programme to mitigate the impact of severe adverse events in HCWs and raise awareness about the SV phenomenon.	**i) Knowledge improvement (main outcome):** After completing the informative and demonstrative package, users significantly improved their knowledge about patient safety terminology, impact and prevalence of adverse events, SV support strategies and recommended actions following a severe adverse event (P<0.001).

For peer review only

Scott, Hirschinger, Cox, McCoig, Hahn-Cover, Epperly et al. (2010)	2009	10 months	US-University of Missouri Healthcare	University of Missouri Health Care faculty and healthcare staff	To provide a peer support programme to facilitate the second victim's transition through the six stages of emotional recovery	**i) Frequency of the encounters**: 49 encounters with forYOU Team members ,13 of which involved referrals to external support; there were 6 team debriefings with an average of 15 HCWs each. The average duration of the encounter with forYOU Team members lasted 30 minutes, while debriefings extended to 77 minutes.
Schuster (2021)	End of 2019	6 months	US- Department of Hematology/Oncology /Stem Cell Transplant Unit- Boston Children's Hospital – 2 separate units: a 30-bed hematology/oncology unit and a 14-bed stem cell transplant unit. Both units are under one hematology/oncology/ bone marrow transplant department within the inpatient pediatric free-standing children's hospital.	Multidisciplinary staff caring for hematology/oncology patients and their families (registered nurses, advanced practice nurses, clinical assistants, physicians, dieticians, child life specialists, environmental services, food tray distributors, patient experience representatives, social workers, resource room staff, chaplains, pain service staff, supply restockers, and students on the floor).	To provide peer to peer support program to promote a safer, supportive and resilient workplace culture, improve staff wellbeing and decrease the frequency of contacts among colleagues for work-related support outside of working hours to cope with stressful situations	**i) Frequency of the programme interventions:** 98 HART shifts;<br><br>**ii) Outcomes related with HART application:** improvement of HCWs' well-being, the number of HCWs reaching out coworkers for support outside working hours decreased after implementing HART; number of breaks increased during the work shifts, the use of hospital resources increased after HART coaches recommend them;<br><br>**iii) Satisfaction with the work-related support after HART implementation:** 25.6% of participants reported to be extremely satisfied with the received support; 49.4% of HCWs felt more supported by leadership.<br><br>**iv) Interactions resolutions:** The majority of interactions were solved in real time (83.2%);<br><br>**v) Subjective Feedback:** The programme fostered a safe, supportive and open work environment. HCWs felt more connected to their colleagues in the workplace.
Calder-Sprackman; Kumar; Gerin-Lajoie; Kilvert; Sampsel (2018)	2014	24 months	Canada – Department of emergency medicine , university of Ottawa	Residents from the emergency department at the university of Ottawa	To provide a peer-support sessions to improve residents wellbeing and create a supportive and resilient workplace	**i) Perception of change after the support programme**: From a total of 20 surveyed HCWs, 95% referred that support and companionship among residents increased after the support the sessions; 58,8% gained an increased awareness of coping strategies to deal with challenges during residency ;<br><br>**ii) Impact of the Rounds on clinical practice:** Half of HCWs referred that the rounds helped them to reflect about their clinical practice, a quarter of HCWs expressed that the rounds had not significantly impacted their clinical practice. Nevertheless, they acknowledged the value of having this type of support available to them;

						**iii) Perception of stress, anxiety and burnout**: 20% of HCWs decreased sensation of burnout and 10% decrease their perceived stress and anxiety;<br><br>**iv) Programme recommendation:** 89% of respondents would recommend ice cream rounds to other colleagues
Peterson, Bergström, Samuelsson, Åsberg, Nygren (2008)	2002	2 months	Sweden- 3 hospitals of the county council	Physicians, registered nurses, nursing assistants, social workers, occupational therapists, physiotherapists, psychologists, dental nurses and hygienists, dentists, service staff, administrators, teachers and technician. participants scored above the 75th percentile on the exhaustion dimension of the Oldenburg Burnout Inventory	To provide a reflecting peer-support group to discuss and reflect on work-related stress and burnout; to help to find out alternative ways to handle perceived stressful situations on an individual level; to provide an opportunity for mutual support between colleagues, to share and compare experiences and learn from each other.	**i) Frequency of the encounters:** 8 peer-support groups with 5–8 participants in each group**;**<br><br>**ii) Perceived impact of the peer support group:** The space for reflection on 'real problems' was valued by the participants; increased knowledge about stress and coping strategies, increased sense of belonging/community; Increased self-confidence;the existence of structured group was appreciated, decreased stress symptoms and anger; behavioural change; sleep improvement;<br><br>**iii) Work-related symptoms and burnout:** Both groups showed an overall decrease from baseline to follow up after 12 months in exhaustion, disengagement, depression and anxiety measures;<br><br>**iv) General health and vitality:** both increased after the intervention;<br><br>**v) Change in work conditions**: statistical significant difference was found in participation at work and support at work after 12 month follow-up.
Rubin, Rassman (2021)	2020	"During spring"	US-large urban healthcare system: Clinical education and practice department in Swedish Health Services	Clinical and non-clinical staff (nurse educator, professional development specialist, programme manager, student intern) and disciplines (nursing, social work) within the Clinical Education and Practice department.	To provide informational and emotional support to help frontline staff thrive in the first wave of the Covid 19 Pandemic	**i) Programme utilization:** 13 out 71 HCWs participated in the programme;<br><br>**ii) Evaluation of programme's resources:** Approximately 85% of surveyed HCWs agreed or strongly agreed that there were adequate resources to support the HCWs during and after stressful events in the healthcare organization. All respondents agreed that assisting the cAIR video presentation was a valuable use of their time. The nature of the content and dedicated time for staff support stood out as the most helpful aspects of the video presentation. Most respondents improved their knowledge and/or skills with the cAIR resources.

For peer review only

Bernburg, Groneberg , Mache (2019)	Missing data	3 months	Germany - psychiatric hospital departments	Nurses full time working in psychiatric hospital department	To provide a mental health promotion intervention to develop self-care skills in psychiatric nurses	**i) Programme's attendance:** 44 nurses were part of the intervention group and 42 nurses of the control group;<br><br>**ii) Perceived job stress:** intervention group perceived lower levels of stress after attending the programme;<br><br>**iii) Relation with patients**: After the programme, significant improvements in nurses' relationship with their patients and lower levels of perceived conflicts were observed ($p < 0.05$);<br><br>**iv) Emotion regulation:** Large effects on emotion regulation skills were found in the first follow up (3 months) and medium effects sizes were found after 6 and 12 months;<br><br>**v) Resilience and self-efficacy:** statistically difference was found in these measures within groups ($p<0.05$)**;**<br><br>**vi) Final course evaluation:** Overall satisfaction with the training (1.39; 1-best score, 5-worse score); nurses displayed a strong level of motivation and interest in learning self-care techniques and applying them in their work. All participants verified that the training was worth attending (learning was meaningful and motivating for selfcare).
Morales, Brown (2019)	Missing data	No info	US- 10-hospital health system	Clinicians working in the 10-hospital system	To provide immediate emotional first aid after a serious adverse event and coach to HCWs on how to respond to patients and their families in a timely, empathetic, consistent, and patient-centred way.	**i) Process and outcome's results of two different scenarios have been described:**<br><br>**Scenario 1 (death after health condition deterioration of a patient affected all ICU nursing team) -** Process outcomes: programme activation was done by the nursing director; group sessions were applied to all nurses of the ICU , Employee Assistant Programme was provided to several nurses; Outcome results: after nurses had receive support from the programme, they described "feeling grateful for the opportunity to talk openly without judgment" about the event. Sessions also enabled for peer support among the nursing team members;<br><br>**Scenario 2 (intensivist involved in a stressful situation during intubation of a patient) -** Process outcomes: critical care unit director activated the programme; "Thinking of You" bag was delivered to the HCW in need. **Outcome results**: the intensivist expressed gratitude for both unit staff and members of the programme.
Cobos-Vargas, Pérez-Pérez, Núñez-Núñez, Casado-	2020	24 months	Spain- Clinico San Cecilio University Hospital	Hospital staff involved in any type of serious clinical incidents that caused or could have been caused (death or	To provide support to patients and their families, to HCWs and the healthcare institution after serious events, and to investigate and develop improvement	**i) Programme activations:** From 25 activations, 23 severe adverse events were investigated;<br><br>**ii) Frequency of the peer support encounters:** 1 to 17 HCWs per adverse event received trained peer support;

For peer review only

Fernández, Bueno-Cavanillas (2022)				serious harm of a patient)	actions, based on Susan's Scott model.	**iii) Time from the event until the activation of the programme:** ranged from 12h to one week;<br>**iv) Number of second victims identified:** 34.8% (n=47) were identified as second victims;<br>**v) Referral for third level of support** : 7 cases (14.9%) were referred, however 4 refused it;<br>**vi) Participants experience after participating in the programme:**increased sense of safety in the workplace; participants valued the interview provided in the second level of support and being able to contribute for the improvement actions after the adverse event occurrence;<br>**vii)Feedback for programme improvement:** The programme must be disseminated for all HCWs to have access to it; leaders should be trained to be more aware of the problem; first level of approach is identified has a problem in the units.
Hinzmann, Forster, Koll-Krüsmann, Schießl , Schneider, Sigl-Erkel et al (2022)	2020	24 months	Germany- health and emergency services	Clinicians and managers from the health and emergency services	To stabilize and restore the ability to act in highly stressful situation including adverse events, preserving clinical teams health and work capacity – in case of need , to transfer HCWs and managers with for specific treatment , initial structures of standard psychotherapeutic care in a low-threshold and timely manner.	**i) Evaluation of the programme:** In 81.4% of the cases the programme provided strong and very strong support to the callers in coping with the burden after a stressful event;<br>**ii) Follow up call after the first call :** 52.9% of callers needed a follow-up appointment with the same supporter after the first call;<br>**iii) Qualitative feedback after the programme** : there was an increased understanding of one's own reactions; openness to listen and understand that emotions are welcomed in a safe environment; knowing where to turn for help; enhanced mutual understanding and peer validation, as well as self-awareness and self-reflexion; leadership involvement increased.

For peer review only

Supplementary Table 4 – Main findings of the study according with the acronym OPERA

Main outcomes of interest	Main findings of the study
**O**rganisational factors<br>**P**eople<br>**E**nvironment<br>**R**ecommendations from previous studies on an organisational level<br>**A**ttributes of the interventions	Dissemination of the programme should be prioritised.
	Communication processes should be facilitated between HCWs and supporters.
	Specific training to peer supporters should be provided.
	Communication between the support team members should be regularly maintained.
	It's essential to create a multidisciplinary team with empathic skills.
	Leadership members should be actively engaged in both implementation and development of the programmes.
	A supportive and open organisational culture will benefit the programme implementation.
	To provide protected time and relief in staffing to participate in the programme (either for the programme's implementation team and for HCWs/SV that seek support) it's important for programme's sustainability.
	Evaluation of the support programme should be regular and overt time.
	Funding is important for programme sustainability.
	Programmes should be formally recognised in the instituition and have designated structures.
	Programmes should be easily accessible and on voluntary basis.
	Confidentiality should be ensured to facilitate HCWs' adherence and overcome potential barriers to participate in the programme.
	Programme's characteristics should be align with HCWs' needs.

Preferred Reporting Items for Systematic reviews and Meta-Analyses extension for Scoping Reviews (PRISMA-ScR) Checklist

SECTION	ITEM	PRISMA-ScR CHECKLIST ITEM	REPORTED ON PAGE #
**TITLE**			
Title	1	Identify the report as a scoping review.	1
**ABSTRACT**			
Structured summary	2	Provide a structured summary that includes (as applicable): background, objectives, eligibility criteria, sources of evidence, charting methods, results, and conclusions that relate to the review questions and objectives.	2
**INTRODUCTION**			
Rationale	3	Describe the rationale for the review in the context of what is already known. Explain why the review questions/objectives lend themselves to a scoping review approach.	4
Objectives	4	Provide an explicit statement of the questions and objectives being addressed with reference to their key elements (e.g., population or participants, concepts, and context) or other relevant key elements used to conceptualize the review questions and/or objectives.	4
**METHODS**			
Protocol and registration	5	Indicate whether a review protocol exists; state if and where it can be accessed (e.g., a Web address); and if available, provide registration information, including the registration number.	4
Eligibility criteria	6	Specify characteristics of the sources of evidence used as eligibility criteria (e.g., years considered, language, and publication status), and provide a rationale.	Pages 5,6, Complementary table 1
Information sources*	7	Describe all information sources in the search (e.g., databases with dates of coverage and contact with authors to identify additional sources), as well as the date the most recent search was executed.	Page 5,Complementary table 1
Search	8	Present the full electronic search strategy for at least 1 database, including any limits used, such that it could be repeated.	Complementary table 1
Selection of sources of evidence†	9	State the process for selecting sources of evidence (i.e., screening and eligibility) included in the scoping review.	5,6
Data charting process‡	10	Describe the methods of charting data from the included sources of evidence (e.g., calibrated forms or forms that have been tested by the team before their use, and whether data charting was done independently or in duplicate) and any processes for obtaining and confirming data from investigators.	6,7
Data items	11	List and define all variables for which data were sought and any assumptions and simplifications made.	6

For peer review only

SECTION	ITEM	PRISMA-ScR CHECKLIST ITEM	REPORTED ON PAGE #
Critical appraisal of individual sources of evidence§	12	If done, provide a rationale for conducting a critical appraisal of included sources of evidence; describe the methods used and how this information was used in any data synthesis (if appropriate).	6
Synthesis of results	13	Describe the methods of handling and summarizing the data that were charted.	7
**RESULTS**			
Selection of sources of evidence	14	Give numbers of sources of evidence screened, assessed for eligibility, and included in the review, with reasons for exclusions at each stage, ideally using a flow diagram.	6,7
Characteristics of sources of evidence	15	For each source of evidence, present characteristics for which data were charted and provide the citations.	7
Critical appraisal within sources of evidence	16	If done, present data on critical appraisal of included sources of evidence (see item 12).	Page 7 and Complementary table 2
Results of individual sources of evidence	17	For each included source of evidence, present the relevant data that were charted that relate to the review questions and objectives.	7- 13
Synthesis of results	18	Summarize and/or present the charting results as they relate to the review questions and objectives.	Pages 7, 9-13 Complementary tables 2 and 3 ;
**DISCUSSION**			
Summary of evidence	19	Summarize the main results (including an overview of concepts, themes, and types of evidence available), link to the review questions and objectives, and consider the relevance to key groups.	14,15,16
Limitations	20	Discuss the limitations of the scoping review process.	15,16
Conclusions	21	Provide a general interpretation of the results with respect to the review questions and objectives, as well as potential implications and/or next steps.	16
**FUNDING**			
Funding	22	Describe sources of funding for the included sources of evidence, as well as sources of funding for the scoping review. Describe the role of the funders of the scoping review.	16

JBI = Joanna Briggs Institute; PRISMA-ScR = Preferred Reporting Items for Systematic reviews and Meta-Analyses extension for Scoping Reviews.
* Where *sources of evidence* (see second footnote) are compiled from, such as bibliographic databases, social media platforms, and Web sites.
† A more inclusive/heterogeneous term used to account for the different types of evidence or data sources (e.g., quantitative and/or qualitative research, expert opinion, and policy documents) that may be eligible in a scoping review as opposed to only studies. This is not to be confused with *information sources* (see first footnote).
‡ The frameworks by Arksey and O'Malley (6) and Levac and colleagues (7) and the JBI guidance (4, 5) refer to the process of data extraction in a scoping review as data charting.
§ The process of systematically examining research evidence to assess its validity, results, and relevance before using it to inform a decision. This term is used for items 12 and 19 instead of "risk of bias" (which is more applicable to systematic reviews of interventions) to include and acknowledge the various sources of evidence that may be used in a scoping review (e.g., quantitative and/or qualitative research, expert opinion, and policy document).

From: Tricco AC, Lillie E, Zarin W, O'Brien KK, Colquhoun H, Levac D, et al. PRISMA Extension for Scoping Reviews (PRISMAScR): Checklist and Explanation. Ann Intern Med. 2018;169:467–473. doi: 10.7326/M18-0850.

For peer review only - http://bmjopen.bmj.com/site/about/guidelines.xhtml
